# RNA-Seq-Based Breast Cancer Subtypes Classification Using Machine Learning Approaches

**DOI:** 10.1155/2020/4737969

**Published:** 2020-10-29

**Authors:** Zhezhou Yu, Zhuo Wang, Xiangchun Yu, Zhe Zhang

**Affiliations:** ^1^College of Computer Science and Technology, Jilin University, Changchun, China; ^2^School of Information Engineering, Jiangxi University of Science and Technology, Ganzhou, China

## Abstract

**Background:**

Breast invasive carcinoma (BRCA) is not a single disease as each subtype has a distinct morphology structure. Although several computational methods have been proposed to conduct breast cancer subtype identification, the specific interaction mechanisms of genes involved in the subtypes are still incomplete. To identify and explore the corresponding interaction mechanisms of genes for each subtype of breast cancer can impose an important impact on the personalized treatment for different patients.

**Methods:**

We integrate the biological importance of genes from the gene regulatory networks to the differential expression analysis and then obtain the weighted differentially expressed genes (weighted DEGs). A gene with a high weight means it regulates more target genes and thus holds more biological importance. Besides, we constructed gene coexpression networks for control and experiment groups, and the significantly differentially interacting structures encouraged us to design the corresponding Gene Ontology (GO) enrichment based on gene coexpression networks (GOEGCN). The GOEGCN considers the two-side distinction analysis between gene coexpression networks for control and experiment groups. The method allows us to study how the modulated coexpressed gene couples impact biological functions at a GO level.

**Results:**

We modeled the binary classification with weighted DEGs for each subtype. The binary classifier could make a good prediction for an unseen sample, and the experimental results validated the effectiveness of our proposed approaches. The novel enriched GO terms based on GOEGCN for control and experiment groups of each subtype explain the specific biological function changes according to the two-side distinction of coexpression network structures to some extent.

**Conclusion:**

The weighted DEGs contain biological importance derived from the gene regulatory network. Based on the weighted DEGs, five binary classifiers were learned and showed good performance concerning the “Sensitivity,” “Specificity,” “Accuracy,” “*F*1,” and “AUC” metrics. The GOEGCN with weighted DEGs for control and experiment groups presented a novel GO enrichment analysis results and the novel enriched GO terms would further unveil the changes of specific biological functions among all the BRCA subtypes to some extent. The R code in this research is available at https://github.com/yxchspring/GOEGCN_BRCA_Subtypes.

## 1. Introduction

The breast invasive carcinoma (BRCA) is regarded as a heterogeneous disease which is difficult to define under the definition of the conventional histopathology [1]. BRCA spans multiple subtypes, each with dissimilar morphology structures and clinical upshots [2]. It is generally accepted that BRCA covers five kinds of intrinsic subtypes at the molecular level, namely, Basal-like, Her2 overexpression (Her2), Luminal A (LumA), Luminal B (LumB), and Normal-like [2]. Sørlie et al. developed a “molecular portrait” method to classify the tumors into five subtypes (i.e., Basal-like, Her2, LumA, LumB, and Normal-like) according to the distinctive gene expression patterns [3]. Hu et al. chose 306 genes with significant differential expression to split cancer into the same five subtypes [4]. Parker et al. also found the same five intrinsic subtypes by utilizing 50 genes (PAM50) and it showed great value for clinical prognosis and prediction [5]. This division can be mapped to the subtypes defined by IHC markers (i.e., ER, PR, and Her2), except for the Normal-like which shares similar IHC description with LumA [2]. However, the existence of the Normal-like subtype is disbelieved by some researchers, owing to the indetermination of its clinical significance. Therefore, several studies only focused on the four kinds of BRCA subtypes other than the Normal-like [1, 6]. Other studies adopted the unsupervised methods to cluster the tumor samples into the different groups and each group represented an individual subtype [7, 8]. In addition, many researcher have proposed different machine learning approaches to carry out cancer subtyping and classification. Dass et al. [9] proposed an improved decision tree for lung cancer subtypes. More importantly, the decision rules discovered in this research can provide reference guidelines for diagnosis and drug development of lung cancer subtypes. Flynn et al. [10] have studied several machine learning approaches, including KNN, random forest, and SVM, using gene expression data to determine the molecular subtypes of cancer. Hijazi and Chan [11] proposed a classification framework for cancer subtypes based on gene expression data. This work studied several different machine learning methods including decision trees, random forests, and SVM for subtype classification. Bazila Banu et al. [12] focused on the performance of the Naive Bayes classifiers in breast cancer classification. Kharya and Soni [13] proposed a weighted Naive Bayes approach for breast cancer classification.

Function enrichment analysis was conducted to acquire the enriched GO terms based on the corresponding DEGs for each subtype [1, 7]. In this research, we focus on the identification of the five subtypes of BRCA (i.e., Basal-like, Her2, LumA, LumB, and Normal-like) using gene expression values based on RNA-Seq data. For the DEGs selection step, we conducted statistical analysis between each subtype compared with the remaining subtypes similar to [14]. Different from the abovementioned method, we strive to utilize the biological importance information of the genes. Hua et al. [15] proposed to construct the miRNA-mRNA dysregulated network to identify breast cancer subtypes based on miRNA expression. Xu et al. [7] proposed the gene regulatory networks named miRNA-TF-mRNA which could reveal the interaction relationship between molecules. Inspired by this method, we integrated the regulatory information to the selection of DEGs. That is to say, we selected the top M genes with high weights after we get the DEGs. The weighted DEGs utilize the interaction information derived from the gene regulatory network and thus reveal the biological importance related to the corresponding regulatory mechanism for different subtypes. Machine learning-based approaches are being applied to conduct feature selection [16], protein-protein interactions prediction [17, 18], and cancer classification [16, 19, 20] and show powerful performance in bioinformatics. In order to validate the effectiveness and discrimination of the weighted DEGs, we constructed a machine learning-based classification model for each subtype, and a binary classifier between control (e.g., non-Basal-like) and experiment (e.g., Basal-like) groups is learned to separate the different groups of data into the actual classes.

We believe that the different groups will certainly hold distinct molecular interaction mechanisms, so we constructed the gene coexpression networks with weighted DEGs based on Pearson correlation coefficients (PCC) for control and experiment groups, respectively. There is a lot of work [21–23] that can use Pearson correlation coefficients to build a coexpression network based on high-throughput FPKM data from TCGA database. In addition, in order to meet the requirements of the normal distribution, we performed some data preprocessing operations, such as log2 transformation. The significantly differential structures not only demonstrate the different interaction relationship among coexpressed gene couples for control and experiment groups, but also encourage us to propose a novel enrichment analysis approach called GO enrichment based on gene coexpression network (GOEGCN).

For GO enrichment analysis, we consider the two-side distinction between gene coexpression networks for control and experiment groups [24]. This means that the coexpressed gene couples which appear in the experiment group instead of the control group can imply that their coexpression is activated (similar to the upregulated expression), and conversely, the ones appearing in control instead of experiment group infer that their coexpression is inhibited (similar to the downregulated expression). This analysis method is different from previous studies [1, 7, 14]. Finally, we recalculated the *p* values using the hypergeometric test [25] and obtained the final enriched GO terms list for each subtype after reordering those GO terms according to the adjusted *p* values.

## 2. Materials and Methods

### 2.1. Data

**Figure 1:**
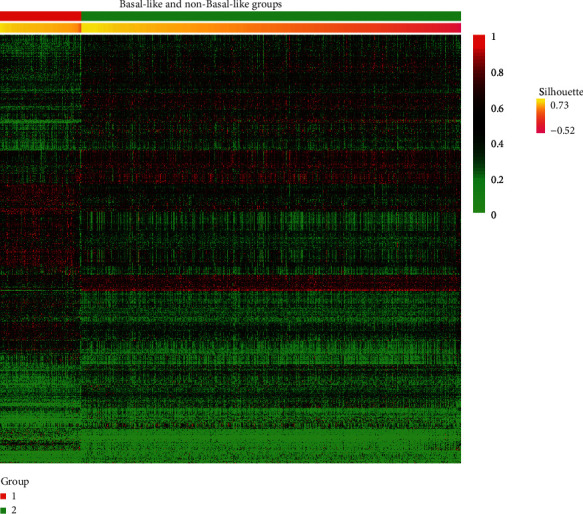
Heatmap for Basal-like and non-Basal-like groups. The left group 1 represents the Basal-like group and the right group 2 denotes the non-Basal-like group.

The RNA-Seq-based gene expression data of BRCA was downloaded from the TCGA database. The FPKM values were adopted in this research. When the tumor data of BRCA are obtained, we filtered out the genes whose mean values are less than 0.2 and variations are less than 2 across the tumor samples. We divided all the tumor samples into five subtypes, Basal-like, Her2, LumA, LumB, and Normal-like, according to the description of BRCA clinical data. The specific tumor sample size for each subtype is demonstrated in Table 1. Five binary classifiers for each subtype were learned to validate the effectiveness and discrimination of the weighted DEGs and make a prediction for the unseen sample. The gene expression profiles for Basal-like (experiment) and non-Basal-like (control) groups are shown in Figure 1. The heatmaps for the other four kinds of subtypes are presented in Figures S1–S4. The data was normalized using log2 transformation and inputted into the binary classifier for each subtype.

### 2.2. Statistical Analysis

The counts data of control and experiment groups were inputted into the voom [26] and limma [27] package to get the DEGs for each subtype. The genes with absolute log fold change ≥0.5 and adjusted *p* value ≤0.01 were kept and regarded as the initial DEGs. In order to explore the biological importance of the DEGs, we utilized the gene regulatory networks proposed by Xu et al. to retrieve the genes with higher weights, i.e., genes that participate in the regulation of more target genes. Let *S*={*g*_1_, *g*_2_, *g*_*N*_} denote the gene set, where *N* is the total number of genes, and the weights of *g*_*i*_ and *g*_*j*_ with *g*_*i*_⟶*g*_*j*_ are calculated with the following equation:

(1) $$\begin{array}{c} W\left(g_{i}\right)=\frac{1-d}{N}+d\sum_{g_{j}\in T\left(g_{i}\right)}\frac{W\left(g_{j}\right)}{L\left(g_{j}\right)}, \end{array}$$

where *d* (0 *<* *d* *<* 1) denotes the damping factor, *T*(*g*_*j*_) is the target genes set that *g*_*i*_ regulates, and *L*(*g*_*j*_) is the total number of regulator genes which regulate *g*_*j*_. Then the top 1,000 genes with high weights were selected from the miRNA-TF-mRNA gene regulatory networks [7]. The reasons why we select the 1,000 genes with high weights are as follows. Firstly, the required quantity for constructing machine learning classifiers can be satisfied to a certain extent. Too many genes will result in the overfitting issue, when the sample size is too small. Besides, we think that choosing the specific quantity is a more direct method. The weighted DEGs for classification were obtained by taking the intersection between the initial DEGs and the top 1,000 mRNA with high weights (ranks). The number of weighted DEGs for classification of each BRCA subtype is illustrated in Table 2 and detailed gene information is shown in Supplementary S1 in Supplementary materials When we conducted the GO enrichment analysis, the top 3,000 genes with high weights were chosen to perform the intersection with the initial DEGs and then we would get the final weighted DEGs for GO enrichment analysis.  S2 in Supplementary Materials File presents the detailed information of weighted DEGs of each BRCA subtype for GO enrichment analysis.

### 2.3. Construct Gene Coexpression Network with Weighted DEGs

**Figure 2:**
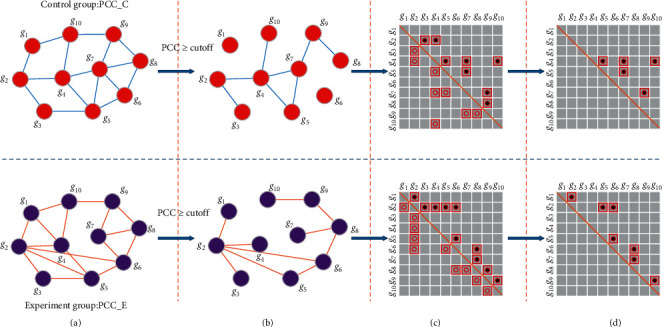
Flowchart to discover the interaction networks structures for control and experiment groups. (a) Construct the gene coexpression networks by PCC. The bold edges denote the higher PCC, and the thin edges represent the lower PCC. (b) Conduct the pruning operation and remove the edges whose PCC values are less than the cutoff. (c) The symmetric matrix forms compared with step (b). (d) Remove the shared network structures between control and experiment groups, and just focus on the specific structures of the upper triangular matrix from control and experiment groups owing to the symmetry.

In order to explore the different interaction structures between the control and experiment groups for each subtype, the gene coexpression networks were constructed by PCC with weighted DEGs for the experiment (e.g., Basal-like) and control (e.g., non-Basal-like) groups. The two gene coexpression networks will carry different interaction information and distinct network structures. When we acquired the gene coexpression networks for control and experiment groups, the edges with low values of PCC were pruned and the edges with higher PCC were retained. We set PCC ≥ 0 : 3 as the threshold in this research. Besides, we utilized the symmetric matrix forms to represent the pruned gene coexpression networks with weighted DEGs. Finally, we removed the shared network structures between the control and experiment groups and then just focused on the differential structures of the upper triangular matrix. The detailed information is illustrated in Figure 2.

### 2.4. GO Enrichment Analysis

Based on the discovery of the significantly differential interaction network structures, we were driven to design a novel GO enrichment analysis called GOEGCN. For the GO enrichment analysis, we considered the two-side distinction analysis between gene coexpression networks for control and experiment groups [24]. Firstly, the GO terms [28–30] with adjusted *p* value ≤0.05 were collected utilizing the weighted DEGs for each subtype. Secondly, the distribution of coexpressed gene couples in the upper triangular matrix was regarded as the background for control and experiment groups, respectively. The subupper triangular matrix of the “geneID” for each GO term was further obtained by scanning the background (upper triangular matrix) of control and experiment groups, respectively. Finally, the hypergeometric test [25] was used to recalculate the *p* values, and the terms whose adjusted *p* values were not greater than 0.05 were retained. After collecting and reordering the results, the novel enriched GO terms were acquired for the control and experiment groups of each subtype, respectively.

## 3. Results

**Figure 3:**
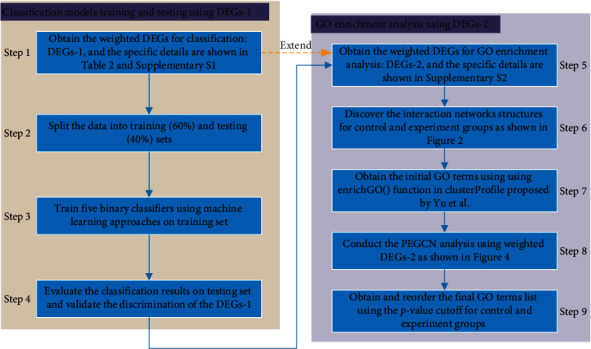
Framework of our proposed algorithm.

The algorithm framework we propose mainly includes two steps, Firstly, the initial GO terms based on the final DEGs are obtained by using Yu's method. Secondly, compared with the DEGs, the difference between coexpression network structures can well show the difference between the control and experiment groups, as shown in Figure 2. Based on this finding, we propose a new GOEGCN method which adopts the hypergeometric test to explore the differences between coexpression network structures, thereby further obtaining the final GO enrichment analysis results. Finally, it is noted that the GOEGCN method still obtains the new GO enrichment results based on the initial GO terms. However, because our proposed method can better show the difference between the control and the experiment groups, the GO enrichment results obtained are more reasonable. The whole process of our proposed algorithm is illustrated in Figure 3.

### 3.1. BRCA Subtypes Classification Using Weighted DEGs

Five kinds of binary classification models were learned on the control (regarded as the negative class) and experiment (regarded as the positive class) groups with weighted DEGs for each subtype. Each binary classifier can make a prediction for an unseen sample (tissue). Three kinds of well-known machine learning approaches (i.e., Naive Bayes, Random Forest, and svmRadial [31]) were applied to train the model, and the sampling method SMOTE [32] was adopted to deal with the imbalanced sample size. The final classification results using 5-fold cross-validation with 100 repeats were used to measure the robustness of our proposed method. Among them, “Sensitivity,” “Specificity,” “Accuracy,” “*F*1,” and “AUC” metrics were used to evaluate the performance of the binary classifiers. Finally, a very important point is that these classification results are to a certain extent to test the validity of the DEGs. So the classification approaches are only a verification one, and the GO enrichment analysis between control and experiment groups is our goal.

**Figure 4:**
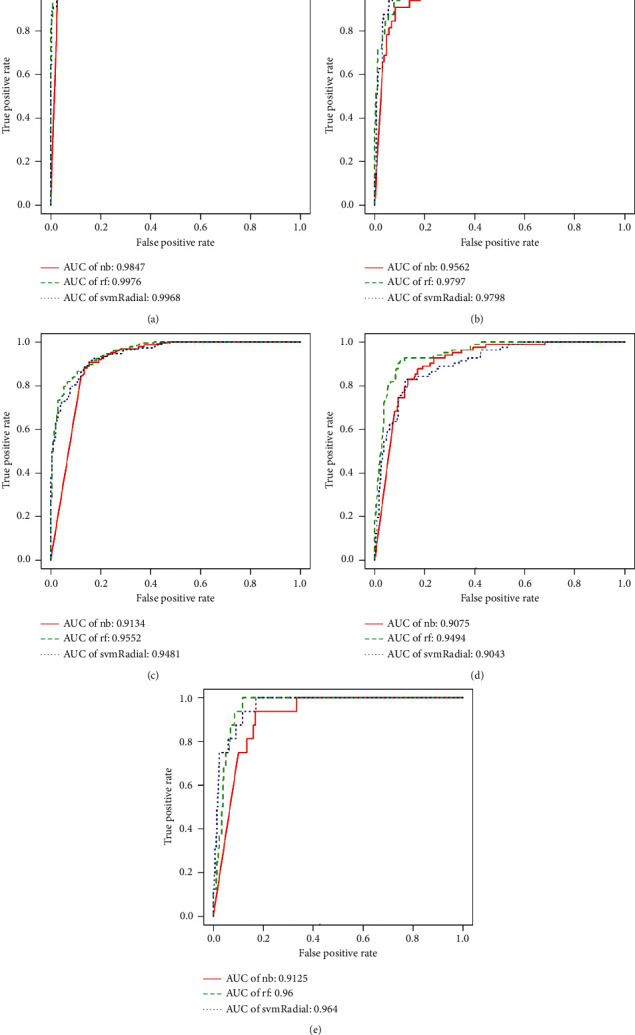
ROC curves of each subtype using three kinds of machine learning approaches. The Area Under Curve (AUC) is used to assess the performance of the binary classifier. (a) The ROC curves of Basal-like using three kinds of machine learning approaches (i.e., nb, rf, and svmRadial). (b) The ROC curves of Her2 using three kinds of machine learning approaches. (c) The ROC curves of LumA using three kinds of machine learning approaches. (d) The ROC curves of LumB using three kinds of machine learning approaches. (e) The ROC curves of Normal-like using three kinds of machine learning approaches.

The specific classification results are reported in Table 3 and the corresponding confusion matrix is shown in Table 4. Three kinds of approaches including “Naive Bayes (nb),” “Random Forest (rf),” and “svmRadial” (SVM with radial basis kernel) were adopted to train the models. Among them, the ROC curves of each subtype of the three kinds of machine learning approaches are shown in Figure 4. It was worth noting that the high “sensitivity” for the positive class (e.g., the Basal-like) showed that the binary classifier could give a good prediction for the class with a smaller sample size. At the same time, the other four metrics (specificity, accuracy, *F*1, and AUC) all illustrated good performances. But for the “Normal-like” one, only the “Naive Bayes” and “Random Forest” gave good “sensitivity” values and the “*F*1” values were relatively low for all the machine learning approaches. The possible explanations are that (1) the “Normal-like” subtype shares a similar IHC status with the “LumA” [2] and (2) some studies reveal that the clinical significance of “Normal-like” subtype is still undetermined and even have a suspect of the existence concerning this kind of subtype [33].

### 3.2. GO Enrichment Analysis for the BRCA Subtypes Using GOEGCN with Weighted DEGs

**Figure 5:**
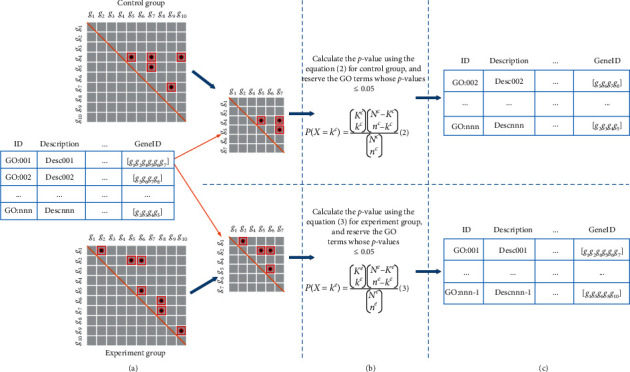
Flowchart for conducting the GOEGCN analysis using weighted DEGs. (a) First of all, the initial enriched GO terms are obtained using GO enrichment analysis. Then a sub symmetric coexpression matrix of “geneID” from each GO term for control or experiment group is constructed, and the interaction network structures of original symmetric coexpression matrix for control or experiment group are regarded as the background. (b) Adopt equations (2) and (3) to recalculate the *p* values for control and experiment groups, respectively. (c) Collect and reorder the results of enriched GO terms which are recalculated and form the final enriched GO terms list for control and experiment groups, respectively.

The weighted DEGs for conducting GO enrichment analysis are described in File S2 in Supplementary Materials. The original method to conduct the differential expression analysis for each subtype is firstly to find DEGs between each subtype and normal data and then use the GO terms [28–30] to discover the corresponding significant GO terms. In this paper, we propose conducting two gene coexpression networks by PCC for control (e.g., non-Basal-like) and experiment (e.g., Basal-like) groups. We have validated that the gene coexpression network structures are significantly different. This discovery drives us to propose the GOEGCN method. This method has two advantages. (1) It can differentiate structures attached to the experiment and control groups. (2) More importantly, the changes in interaction information between control and experiment groups can be explored. In other words, the interaction information attached to experiment group but not to control group reveals that some biological functions are activated; vice versa, the corresponding biological functions are inhibited. The specific process for conducting the GOEGCN analysis using weighted DEGs is shown in Figure 5. Equations (2) and (3) are adopted to recalculate the *p* values for control and experiment groups, respectively.

(2) $$\begin{array}{c} P\left(X=k^{c}\right)=\frac{\left(\begin{array}{c} K^{c}\\ k^{c} \end{array}\right)\left(\begin{array}{c} N^{c}-K^{c}\\ n^{c}-k^{c} \end{array}\right)}{\left(\begin{array}{c} N^{c}\\ n^{c} \end{array}\right)}, \end{array}$$

where *N*^*c*^ denotes the scatter of background (upper triangular matrix of gene coexpression network) for the control group, and *n*^*c*^ represents the scatter of subupper triangular matrix for each GO term. *K*^*c*^ denotes the scatter of inhibited coexpressed gene couples in background for the control group, and *k*^*c*^ represents then scatter of inhibited coexpressed gene couples in subupper triangular matrix.

(3) $$\begin{array}{c} P\left(X=k^{e}\right)=\frac{\left(\begin{array}{c} K^{e}\\ k^{e} \end{array}\right)\left(\begin{array}{c} N^{e}-K^{e}\\ n^{e}-k^{e} \end{array}\right)}{\left(\begin{array}{c} N^{e}\\ n^{e} \end{array}\right)}, \end{array}$$

where *N*^*e*^ denotes the scatter of background (upper triangular matrix of gene coexpression network) for the experiment group, and *n*^*e*^ represents the scatter of subupper triangular matrix for each GO term. *K*^*e*^ denotes the scatter of activated coexpressed gene couples in background for experiment group, and *k*^*c*^ represents the scatter of activated coexpressed gene couples in subupper triangular matrix.

We conducted pathway enrichment analysis using the PEGCN with weighted DEGs for the control and experiment groups of each subtype, and the top 10 enriched pathways for Basal-like are shown in Table 5. The detailed enriched pathway results for all subtypes are shown in Files S3–S7 in Supplementary Materials.

## 4. Discussion

Although several computational approaches have been proposed to identify the subtypes of BRCA, no comprehensive explanation was given on the molecular regulatory mechanisms of the distinguished subtypes. To well explore the interaction network for each subtype will contribute to providing personalized treatments for different patients. In this research, the weighted DEGs that carry the regulatory information derived from the gene regulatory networks are adopted to conduct the classification tasks between different groups of subtypes. Based on the weighted DEGs, we aim to explore the interaction mechanisms for each BRCA subtype using gene expression values based on RNA-Seq data.

The heatmap figures (e.g., Figure 1) can show the differential gene expression profiles based on the weighted DEGs. Also, the machine learning-based approaches are adopted to train the binary classifier for each subtype. Three kinds of approaches, “Naive Bayes (nb),” “Random Forest (rf),” and “svmRadial,” were adopted to train the models and five kinds of metrics, “sensitivity,” “specificity,” “Accuracy,” “*F*1,” and “AUC,” were adopted to evaluate the performance of the five binary classifiers. The high metric values verify the robustness and effectiveness of our proposed method.

We also explored the interaction mechanisms derived from the gene coexpression networks of control and experiment groups, and the significantly differential structures of gene coexpression networks validate the different interaction relationships among coexpressed gene couples. More importantly, this discovery inspires us to further investigate the biological function changes using the proposed GOEGCN method. The novel enriched GO terms are obtained according to whether the interaction mechanisms of coexpressed gene couples are activated or inhibited. The two-side enriched GO terms will provide more information for GO enrichment analysis.

The specific analysis of enriched GO terms for each subtype using GOEGCN is as follows.

1. For Basal-like subtype, this kind of subtype has low or no expression for the hormone receptors and Her2 receptor (i.e., ER-PR-Her2-), while it holds the high expression of basal markers and high expression of genes related to proliferation [2]. The basal markers comprise keratins 5, keratins 6, keratins 14, keratins 17, and the epidermal growth factor receptor (EGFR) [2, 34]. Their expression profiles are similar to the ones of basal epithelial cells and the ones of normal breast myoepithelial cells [2, 34]. Besides, the low BRCA1 expression, as well as TP53 mutation, tends to exist in the Basal-like tumors with basal cytokeratin expression [2, 3, 35].
2. As shown in Table 5 and File S3 in Supplementary Materials, the enriched GO terms for the control group are based on the coexpressed gene couples which are inhibited in the corresponding gene coexpression network. The enriched GO terms for the experiment group are based on the coexpressed gene couples which are activated in the corresponding gene coexpression network. The common group holds the enriched GO terms which are shared between the control and experiment groups. The enriched GO terms of the control group are related to the “epithelium,” “cell adhesion,” “epithelial cell proliferation,” “epithelial cell migration,” etc. The ones of the experiment group are concentrated in “immune response,” “protein signal transduction,” “growth factor,” “cell proliferation,” “catabolic process,” “cell cycle” etc., and one possible reason is that the Basal-like subtype is likely to belong to Grade 3 tumor, so the immune response will work during this phase. The “lymphocyte,” “inflammatory,” “cell proliferation,” “immune response,” etc. are discovered in the common group. These enriched GO terms are consistent with the high expression of basal markers and high expression of genes related to proliferation to some extent.
3. The Her2 overexpression subtype has low or no expression for hormone receptors and high expression of the Her2 receptor (i.e., ER-PR-Her2+). The Her2 is responsible for encoding the Her2 receptor [33]. This subtype is also characterized by overexpression of genes related to Her2 amplicon (e.g., GRB7 [34] and PGAP3 [36]). This subtype has a high proliferation rate, 75% of Her2 has high histological grade and nuclear grade, and 40%–80% of Her2 has TP53 mutation resided in this subtype [2, 33, 37]. The Her2 is likely to belong to Grade 3 tumor [2] and carries a poor prognosis [3, 8, 38]. The more aggressive behaviors in biological and clinical areas are also one of features of this subtype [33].
4. As shown in File S4 in Supplementary Materials, the enriched GO terms for control group are related to “chromosome segregation,” “cell cycle phase transition,” “nuclear division,” “cell cycle,” “epithelial cell proliferation,” “steroid hormone,” etc.; the enriched GO terms for experiment group are related to “calcium ion,” “leukocyte migration,” “lymphocyte differentiation,” “endothelial cell proliferation,” etc.; and there are no shared enriched GO terms in common group. These enriched GO terms are associated with the low expression of hormone receptors and high expression of Her2 receptor to some extent.
5. The LumA subtype is ER or PR positive and Her2 negative, while the LumB subtype is ER or PR positive and Her2 positive [2]. Compared with the LumB subtype, ER-related genes have higher expression and proliferative genes have lower expression in LumA [2, 33]. The expression of luminal epithelial cytokeratins (CK8 and CK18) and the ER1 luminal associated markers, as well as the genes related to ER activation (e.g., BCL2, LIV1, FOXA1, XBP1, GATA3, CCND1, erbB3, and erbB4 [8, 34, 39]) is the main characteristic of LumA [2, 33].
6. As shown in File S5 in Supplementary Materials, the enriched GO terms of control group are associated with “gland development,” “epithelium development,” “steroid hormone,” “branching structure,” “T cell differentiation,” “immune response,” “cell cycle,” etc., the ones for the experiment group are involved in “acid chemical,” “epithelial cell proliferation,” “ERK1 and ERK2 cascade,” “calcium ion,” “peptidyl-tyrosine modification,” “epithelial cell migration,” etc., and the ones for the common group are “response to acid chemical,” “neuron projection development,” “metabolic process,” “response to peptide,” “protein kinase B,” etc.
7. Compared with LumA, LumB tends to have a worse prognosis and more aggressive phenotypes as well as higher histological grade [33]. Besides, the proliferation-related genes (e.g., v-MYB, GGH, LAPTMB4, NSEP1, and CCNE1) have an increased expression, and the genes of growth receptor signaling [40] also present an increased expression in LumB [33].
8. As shown in File S6 in Supplementary Materials, the enriched GO terms of the control group are associated with “gland development,” “epithelial cell development,” “gland epithelium development,” “ERK1 and ERK2 cascade,” “cell cycle,” “phosphorylation,” etc., and the ones for experiment group are involved in “extracellular matrix,” “growth factor,” “phospholipase activity,” “cell growth,” “cell adhesion,” “angiogenesis,” etc., and the common group are “epithelial cell proliferation,” “steroid hormone,” “branching epithelium,” “muscle cell proliferation,” etc. These enriched GO terms are consistent with the increased expression of proliferation-related genes and growth receptor signaling to some extent.
9. The Normal-like subtype is ER negative and/or PR negative with a low level of Ki-67 protein. Few studies have been conducted to this subtype and its specific clinical significance is still undetermined [33]. The adipose tissue expression is one of its characteristics, and this subtype has the intermediate prognosis between Basal-like subtype and luminal subtypes.

As shown in File S7 in Supplementary Materials, the enriched GO terms of the control group are associated with “morphogenesis of an epithelium,” “vasculature development,” “angiogenesis,” “gland morphogenesis,” “steroid hormone,” “cell adhesion,” “leukocyte migration,” “lymphocyte activation,” etc., the ones for the experiment group are involved in “cellular protein localization,” “biosynthetic process,” “phosphatidylinositol metabolic,” “chromatin assembly,” “nucleosome assembly,” etc., and the one for the common group is “kidney epithelium development.”

Although our proposed approaches show good performance, we also admit that some limitations still exist.

1. Only the single-omics mRNA data (i.e., gene expression data) was used to train the binary classifiers for each subtype. This main purpose is to ensure the consistency with the subsequent enrichment analysis which is only available for the mRNA molecules. In spite of this, our binary classifiers still perform well.
2. The interaction networks between control and experiment groups are derived from the gene coexpression networks. However, the effective fusion between the gene coexpression networks and gene regulatory networks will show powerful interaction information, and this will be our follow-up work.

## 5. Conclusion

In this paper, we proposed attaching the biological importance of regulatory information to the differential expression analysis. Based on the weighted DEGs, the binary classifier for each subtype was learned. The experimental results validated the effectiveness of the weighted DEGs and each binary classifier for each subtype could make a good prediction for an unseen sample. More importantly, we constructed the gene coexpression networks for control and experiment groups using weighted DEGs, respectively, and we further explored the interaction mechanisms between these two groups. The significantly differential structures drove us to develop the GOEGCN to conduct GO enrichment analysis based on whether the coexpressed gene couples were activated or inhibited. The novel *p* values were recalculated using the hypergeometric test and after reordering the adjusted *p* values, the novel enriched GO terms were acquired for control and experiment groups, respectively. The novel enriched GO terms could give some explanation for the biological function changes of each BRCA subtype to some degree. In the future, we will explore the effective fusion between gene coexpression networks and gene regulatory networks. Based on the novel network structures, we will further investigate the specific interaction mechanisms and reveal the detailed changes of biological functions across BRCA subtypes.

## Figures and Tables

**Table 1:** The tumor sample number for each subtype of BRCA.

Subtypes	Basal-like	Her2	LumA	LumB	Normal-like
Number	192	82	564	207	40

**Table 2:** The number of weighted DEGs of each BRCA subtype for classification.

Subtypes	Basal-like	Her2	LumA	LumB	Normal-like
Weighted DEGs	376	157	249	206	249

**Table 3:** RNA-Seq-based BRCA subtypes classification using 5-fold cross-validation with 100 repeats. The first column denotes the five kinds of subtypes, and we built a binary classifier for each subtype by splitting the data into control and experiment groups. The sample size of two groups was imbalanced, so the “SMOTE” sampling method in the second column was utilized to lessen the interference of imbalanced data. The “LumA” subtype was an exception because it had sufficient samples. The third column denotes the five kinds of metrics used in this experiment, and the remaining columns are the three kinds of machine learning approaches adopted in this research, where the “svmRadial” represents the svm with radial basis kernel.

Subtypes	Sampling	Metrics	nb	rf	svmRadial
Basal-like	SMOTE	Sensitivity	**0.9737**	0.9605	**0.9737**
		Specificity	0.9580	**0.9916**	0.9720
		Accuracy	0.9607	**0.9861**	0.9723
		*F*1	0.8970	**0.9605**	0.9250
		AUC	0.9847	**0.9976**	0.9968

Her2	SMOTE	Sensitivity	**0.9063**	0.7813	0.8750
		Specificity	0.8853	**0.9601**	0.9526
		Accuracy	0.8868	**0.9469**	**0.9469**
		*F*1	0.5421	0.6849	**0.7089**
		AUC	0.9562	0.9797	**0.9798**

LumA	None	Sensitivity	**0.9067**	0.8667	**0.9067**
		Specificity	0.8173	**0.8846**	0.8462
		Accuracy	0.8637	0.8753	**0.8776**
		*F*1	0.8737	0.8784	**0.8850**
		AUC	0.9134	**0.9952**	0.9481

LumB	SMOTE	Sensitivity	**0.8415**	0.8171	0.5488
		Specificity	0.8376	0.9288	**0.9544**
		Accuracy	0.8383	**0.9076**	0.8776
		*F*1	0.6635	**0.7701**	0.6294
		AUC	0.9075	**0.9494**	0.9043

Normal-like	SMOTE	Sensitivity	**0.8125**	0.7500	0.5000
		Specificity	0.8517	0.9498	**0.9833**
		Accuracy	0.8502	0.9424	**0.9654**
		*F*1	0.9163	0.9695	**0.9821**
		AUC	0.9125	0.9600	**0.9640**

**Table 4:** The confusion matrix of the classification results corresponding to Table 3, where “P” represents a certain subtype (e.g., Basal-like) and “N” represents the remaining four subtypes (e.g., Her2, LumA, LumB, and Normal-like). The columns of the confusion matrix represent the reference (i.e., true) class labels, and the rows represent the prediction class labels.

Subtypes	Nb			rf			svmRadial		
Basal-like	Pred\Ref	P	N	Pred\Ref	P	N	Pred\Ref	P	N
	P	74	15	P	73	3	P	74	10
	N	2	342	N	3	354	N	2	347

Her2	Pred\Ref	P	N	Pred\Ref	P	N	Pred\Ref	P	N
	P	29	46	P	25	16	P	28	19
	N	3	355	N	7	385	N	4	382

LumA	Pred\Ref	P	N	Pred\Ref	P	N	Pred\Ref	P	N
	P	204	38	P	195	24	P	204	32
	N	21	170	N	30	184	N	21	176

LumB	Pred\Ref	P	N	Pred\Ref	P	N	Pred\Ref	P	N
	P	69	57	P	67	25	P	45	16
	N	13	294	N	15	326	N	37	335

Normal-like	Pred\Ref	P	N	Pred\Ref	P	N	Pred\Ref	P	N
	P	356	3	P	397	4	P	411	8
	N	62	13	N	21	12	N	7	8

**Table 5:** Top 10 enriched GO terms for Basal-like subtype of BRCA, where the “*p*.adjust” means the *p* values are adjusted by the BH approach.

Basal-like	Top 10 enriched GO terms	*p* adjust
Control group	Morphogenesis of an epithelium	0
	Response to lipopolysaccharide	0
	Response to molecule of bacterial origin	0
	Positive regulation of cell adhesion	0
	Regulation of cell-cell adhesion	0
	Gliogenesis	0
	Peptidyl-tyrosine phosphorylation	0
	Peptidyl-tyrosine modification	0
	Wnt signaling pathway	0

Experiment group	Adaptive immune response based on somatic recombination of immune receptors built from immunoglobulin superfamily domains	0
	Phospholipase C-activating G protein-coupled receptor signaling pathway	2.18*E* − 09
	Protein-DNA complex subunit organization	1.33*E* − 08
	Regulation of cellular response to growth factor stimulus	4.06*E* − 08
	RNA catabolic process	1.33*E* − 07
	Regulation of gene silencing by miRNA	1.81*E* − 07
	Skeletal system morphogenesis	8.43*E* − 07
	Regulation of gene silencing	9.87*E* − 07
	Regulation of interferon-gamma production	1.21*E* − 06

Common	Extracellular structure organization	0
	Lymphocyte differentiation	0
	Regulation of inflammatory response	2.99*E* − 12
	I-kappaB kinase/NF-kappaB signaling	6.03*E* − 12
	T cell activation	1.03*E* − 11
	B cell activation	7.85*E* − 11
	Positive regulation of response to external stimulus	6.35*E* − 10
	Ribonucleoprotein complex biogenesis	1.33*E* − 08
	Formation of primary germ layer	1.29*E* − 07

## Data Availability

The RNA-Seq-based processed data used to support the findings of this study have been deposited in the Github repository (https://github.com/yxchspring/GOEGCN_BRCA_Subtypes/tree/master/Data), and the original data can be accessed in the open TCGA database (https://www.cancer.gov/tcga).

## Supplementary Materials

Supplementary Materials Figure S1: heatmap for Her2 and non Her2 groups. The left group 1 represents the Her2 group and the right group 2 denotes the non-Her2 group. Figure S2: heatmap for LumA and non-LumA groups. The left group 1 represents the LumA group and the right group 2 denotes the non-LumA group. Figure S3: heatmap for LumB and non-LumB groups. The left group 1 represents the LumB group and the right group 2 denotes the non-LumB group. Figure S4: heatmap for Normal-like and non-Normal-like groups. The left group 1 represents the Normal-like group and the right group 2 denotes the non-Normal-like group. S1 File: the detailed information of weighted DEGs for classification. S2 File: the detailed information of weighted DEGs for GO enrichment analysis. S3 File: the detailed enriched GO terms results for Basal-like subtype. S4 File: the detailed enriched GO terms results for Her2 subtype. S5 File: the detailed enriched GO terms results for LumA subtype. S6 File: the detailed enriched GO terms results for LumB subtype. S7 File**:** the detailed enriched GO terms results for Normal-like subtype.
