# Supplementary material for: RNA-Seq-Based Breast Cancer Subtypes Classification Using Machine Learning Approaches
Source: Comput Intell Neurosci. 2020 Oct 29;2020:4737969. doi: 10.1155/2020/4737969 (PMC7644310; doi:10.1155/2020/4737969)
Supplement: Supplementary Materials — Figure S1: heatmap for Her2 and non Her2 groups. The left group 1 represents the Her2 group and the right group 2 denotes the non-Her2 group. Figure S2: heatmap for LumA and non-LumA groups. The left group 1 represents the LumA group and the right group 2 denotes the non-LumA group. Figure S3: heatmap for LumB and non-LumB groups. The left group 1 represents the LumB group and the right group 2 denotes the non-LumB group. Figure S4: heatmap for Normal-like and non-Normal-like groups. The left group 1 represents the Normal-like group and the right group 2 denotes the non-Normal-like group. S1 File: the detailed information of weighted DEGs for classification. S2 File: the detailed information of weighted DEGs for GO enrichment analysis. S3 File: the detailed enriched GO terms results for Basal-like subtype. S4 File: the detailed enriched GO terms results for Her2 subtype. S5 File: the detailed enriched GO terms results for LumA subtype. S6 File: the detailed enriched GO terms results for LumB subtype. S7 File: the detailed enriched GO terms results for Normal-like subtype. [file 4737969.f1.zip › supplementary materials/S1 File.docx]

**S1 File:** The detailed information of weighted DEGs for classification.

| **No.** | **Basal-like** | **Her2** | **LumA** | **LumB** | **Normal-like** |
| --- | --- | --- | --- | --- | --- |
| 1 | FOXA1 | ERBB2 | CENPA | ESPL1 | TP63 |
| 2 | BCL11A | RARG | CCNB2 | CCNE2 | NGFR |
| 3 | GATA3 | MMP1 | MYBL2 | DSN1 | PDGFA |
| 4 | CCNE1 | IGF1R | ORC6 | CENPE | OXTR |
| 5 | AR | HDAC5 | KIF2C | AURKA | EDN3 |
| 6 | IL12RB2 | RRM2 | CDC20 | ZWINT | FGF2 |
| 7 | CENPA | DNMT3B | CCNA2 | DHFR | EGR2 |
| 8 | YBX1 | CBX2 | CDC45 | CDC6 | EDNRB |
| 9 | AURKB | CDC6 | PLK1 | SGO1 | KIT |
| 10 | CDC20 | PMAIP1 | AURKA | BRCA1 | S1PR1 |
| 11 | CDCA8 | SMAD4 | ORC1 | CENPU | LMO2 |
| 12 | GAL | IL6ST | CDCA8 | BUB1B | EGFR |
| 13 | CDC25A | BCL2 | UBE2C | KNTC1 | ETS2 |
| 14 | ORC1 | TRH | CDC25A | CCNB1 | BCL11A |
| 15 | TAF4B | MED13L | SGO1 | KIF18A | CAV1 |
| 16 | SKP2 | MYC | BIRC5 | TOP2A | EDN1 |
| 17 | IL6ST | MED24 | FOXM1 | MAD2L1 | SAA1 |
| 18 | NDC80 | MED1 | BUB1 | EDN3 | SNAI2 |
| 19 | RARA | STAT5B | CDCA5 | CDK1 | RARB |
| 20 | RNF8 | ESR1 | AURKB | BIRC5 | FLI1 |
| 21 | E2F3 | IRS1 | NDC80 | LMNB1 | PTGS2 |
| 22 | CCKBR | DKK1 | CHEK1 | NUF2 | FOXO1 |
| 23 | CHEK1 | KAT2A | RRM2 | UBE2C | JAK3 |
| 24 | KIF2C | STAT6 | KIF18A | POLR2K | EGR3 |
| 25 | SKP1 | IVL | E2F2 | CCNA2 | MAFF |
| 26 | ORC6 | TLE1 | BUB1B | PCNA | CD40 |
| 27 | CEBPB | AURKA | MAD2L1 | E2F1 | ANXA1 |
| 28 | MED30 | AKT1 | IL6ST | ETS2 | EBF1 |
| 29 | BHLHE40 | HIF1A | CCNE1 | SGO2 | A2M |
| 30 | MAGOH | ADCY9 | CDK1 | ANXA1 | PRKCA |
| 31 | CBX2 | ORC6 | CENPE | MCM8 | CXCL1 |
| 32 | HMGA1 | CDC45 | ESR1 | WWTR1 | RUNX3 |
| 33 | KLF5 | CDK8 | EZH2 | RRM2 | PARP1 |
| 34 | E2F2 | MYCN | NUF2 | PTPN1 | EGR1 |
| 35 | CDC45 | EPO | CCNB1 | RBL1 | IL6 |
| 36 | ODC1 | EDN3 | E2F1 | CCNB2 | TLR2 |
| 37 | PLK1 | CXCL8 | BCL2 | BUB1 | FOSL1 |
| 38 | CCNB2 | SMARCD3 | LMNB1 | MYBL2 | CYR61 |
| 39 | FOXM1 | IKBKB | SKP2 | CDCA5 | LPL |
| 40 | CDT1 | IRS2 | PGR | EGFR | MEF2C |
| 41 | PIM1 | ZWINT | CDC6 | BCL11A | PENK |
| 42 | ESR1 | MYBL2 | ESPL1 | PLK1 | FOSB |
| 43 | MYB | TAC1 | CENPF | TCF7L2 | MAF |
| 44 | CDKN2A | SGO1 | CDT1 | PTGS2 | MTA3 |
| 45 | TGFB3 | TGFBR1 | GATA3 | CDC45 | ALB |
| 46 | EZH2 | ADCY3 | MYB | ESR1 | CCND3 |
| 47 | NUF2 | FOXO4 | DNMT3B | CXCL1 | EPAS1 |
| 48 | BUB1 | CDKN1B | YBX1 | NDC80 | ZBTB16 |
| 49 | CHEK2 | CCNE1 | CBX2 | JDP2 | BMP2 |
| 50 | ENO1 | CDK1 | TYMS | STAT5A | BUB3 |
| 51 | CCNA2 | BTRC | CHEK2 | RAD21 | ATF3 |
| 52 | NOTCH1 | STAT5A | FOXA1 | C3 | SRF |
| 53 | ZBTB7A | FOXP3 | HMGA1 | RELB | NR2F1 |
| 54 | CDK6 | ORC1 | SGO2 | YWHAZ | FYN |
| 55 | LYN | CENPA | ENO1 | FOXM1 | RBBP5 |
| 56 | FOSL1 | CCND1 | ZWINT | CENPF | NOS3 |
| 57 | RUNX3 | CCNB2 | INCENP | FGF2 | CSF2RB |
| 58 | CDCA5 | CCNE2 | TK1 | EGR1 | TOP2A |
| 59 | EGFR | SMAD3 | TAC1 | COL18A1 | GNG2 |
| 60 | CCNH | TFRC | RBL1 | CYR61 | TEK |
| 61 | WDR77 | ENO1 | TGFB3 | JUNB | CCNB1 |
| 62 | TYMS | TK1 | AR | BMP2 | POLR3K |
| 63 | UBE2C | LAMA3 | PCNA | TK1 | KDM5B |
| 64 | CXCL1 | GPER1 | MCM3 | CAV1 | GNAI1 |
| 65 | SGO1 | APOA1 | TOP2A | NOTCH1 | CD14 |
| 66 | TEAD4 | LPAR3 | PDGFD | APP | CENPF |
| 67 | POLR1C | FGF2 | ZBTB16 | CENPA | SKI |
| 68 | MED7 | CDC25A | DSN1 | NPAS2 | PPARG |
| 69 | ADCY9 | CHRM1 | HSPD1 | JUN | PRKCB |
| 70 | GLI3 | BUB1 | PTGER3 | RARB | CASP1 |
| 71 | MYBL2 | IL2RA | BTRC | OAS3 | TAC1 |
| 72 | TLX1 | CENPU | TFF1 | SAA1 | CCND2 |
| 73 | BIRC5 | SFTPD | BHLHE40 | POMC | ZWINT |
| 74 | HDAC2 | E2F2 | GLI3 | NME1 | TNFSF12 |
| 75 | BCL2L1 | LIN28A | ALYREF | KIF2C | DUSP1 |
| 76 | PPP2CA | FASN | SNRPG | ORC6 | RPS6KA3 |
| 77 | LIN9 | CDCA5 | CENPU | NGFR | PDLIM7 |
| 78 | KIF18A | JAG1 | AGO2 | LPAR3 | MAZ |
| 79 | ADORA2B | UBE2C | GAPDH | AURKB | FABP4 |
| 80 | PBX1 | KIF2C | CCNE2 | CDCA8 | CXCL12 |
| 81 | MSH2 | GSTA2 | MAP3K1 | CDC25A | IRF8 |
| 82 | AGO2 | NFATC1 | MED30 | PRKCA | TBX2 |
| 83 | BCL2A1 | ADCY1 | HDAC2 | RPS6KA2 | ADAR |
| 84 | SF3B6 | TEAD1 | CYCS | TP63 | HSPA4 |
| 85 | NDRG1 | BUB1B | E2F3 | FOSL1 | PBX1 |
| 86 | MCM3 | GATA3 | RORC | NFATC1 | PLCB2 |
| 87 | NMU | PLAU | LIN9 | GATA3 | CSF2RA |
| 88 | ADCY6 | CCNA2 | MSH2 | CTGF | RPL4 |
| 89 | INCENP | CDC20 | ADCY9 | SOCS3 | UCHL5 |
| 90 | TAF11 | CCNB1 | ADRA2A | TYMS | RUVBL1 |
| 91 | NKX2-5 | GLI3 | CDK8 | E2F2 | NR4A1 |
| 92 | RNASEL | GNA14 | ODC1 | FOXA1 | RPL5 |
| 93 | TFDP1 | LMNB1 | IKBKB | A2M | CCL2 |
| 94 | SREBF1 | PTGER3 | IL12RB2 | KIT | PDGFD |
| 95 | NRIP1 | CEBPD | ZWILCH | EDN1 | CD3E |
| 96 | ALYREF | IRF4 | DUSP4 | TFF1 | BCL6 |
| 97 | SNRPD1 | CD44 | MCM8 | ETS1 | ETS1 |
| 98 | SSRP1 | EGR3 | AGTR1 | SOX9 | RPL11 |
| 99 | CDK8 | SDC2 | YWHAZ | FYN | KNTC1 |
| 100 | CCND1 | FHL2 | IRS1 | RUNX3 | IGF2 |
| 101 | MAPK9 | E2F1 | PABPC1 | NPY | HEY1 |
| 102 | KDM1A | PGR | IGF1R | RARA | CKAP5 |
| 103 | EPOR | BHLHE40 | H2AFX | PBX1 | NR3C1 |
| 104 | BCL2 | ISG20 | TNFSF12 | TEAD2 | KLF2 |
| 105 | DUSP4 | GNA15 | RUNX1 | MAFF | STAT5A |
| 106 | GAPDH | PLK1 | CEBPB | PENK | NCAM1 |
| 107 | SNRPG | KIF18A | NDRG1 | ORC1 | CDK1 |
| 108 | BTRC | FPR3 | GRP | CEBPD | GADD45A |
| 109 | CENPF | NPY | KLF2 | FOS | RBBP7 |
| 110 | E2F1 | AGTR1 | ADCY1 | IL6 | JUN |
| 111 | TFF1 | BMP2 | NMU | CCKBR | KLF5 |
| 112 | AURKA | LYZ | RARA | GNA15 | RPS6KA2 |
| 113 | BUB1B | PDGFD | GAL | GADD45A | SGO1 |
| 114 | RBL1 | HBB | IGF2 | ASCL1 | ZEB2 |
| 115 | DNMT3B | MYB | IFITM2 | CCNA1 | RPL3 |
| 116 | MAP3K1 | CXCR6 | EPOR | TRH | PRKCD |
| 117 | SENP3 | CENPE | KNTC1 | S1PR1 | RAD21 |
| 118 | WWTR1 | AURKB | MMP1 | GSTA1 | CD247 |
| 119 | GNA14 | CDCA8 | EGR3 | CDK6 | MAPK11 |
| 120 | RORC | CDT1 | TEK | RPS6KA3 | SNRPE |
| 121 | RELB | CDH1 | CENPH | MAPK11 | SGO2 |
| 122 | ZBTB16 | BIRC5 | ARHGAP35 | GNAI1 | SOCS3 |
| 123 | NFIC | IFITM2 | GSTA2 | CHRM1 | LPAR3 |
| 124 | YES1 | CAV1 | ADCY6 | PIM1 | TLR4 |
| 125 | IGF1R | CCKBR | NRIP1 | SIX1 | CCNE2 |
| 126 | IFITM2 | IFITM1 | FOS | SIX5 | WWTR1 |
| 127 | CDKN2C | NMU | ZEB1 | GATA6 | IKZF1 |
| 128 | MAD2L1 | NRIP1 | DUSP1 | HSPB1 | FOS |
| 129 | PTGS2 | TAF4B | IL2RA | KLF5 | GBP2 |
| 130 | BMP2 | EGR1 | RAD21 | CDT1 | CDK6 |
| 131 | IRS1 | LEF1 | BMP4 | NCAM1 | CEBPD |
| 132 | RB1 | EDNRB | E2F5 | SYCP2 | ALDOA |
| 133 | GSTA1 | FOXM1 | HBB | ATF3 | GSTA1 |
| 134 | CENPE | FOS | THRB | CDH1 | SOCS1 |
| 135 | GDF15 | BCL2A1 | GDF15 | CD44 | NUF2 |
| 136 | IL2RA | KIT | TFRC | MMP2 | NFATC2 |
| 137 | E2F5 | ADRA2A | BCL2A1 | SNAI2 | TCF7L2 |
| 138 | DNMT3A | AGT | GNA14 | GATA4 | CSF1R |
| 139 | THRB | IGF2 | S1PR1 | AKT3 | IRAK2 |
| 140 | FASN | CTGF | MED13L | TAC1 | TWIST1 |
| 141 | ARHGAP35 | LPL | CCND1 | CCND1 | KIF18A |
| 142 | PRKCD | NGFR | TLX1 | ADORA2B | PCNA |
| 143 | E2F4 | CDH2 | LPAR1 | GDF15 | KLK3 |
| 144 | CHRM1 | NR4A1 | FLT3 | IFIT1 | LIN9 |
| 145 | SNAI1 | GNAO1 | F2R | TLR2 | CD3D |
| 146 | CTNNA1 | PTGS2 | RPS6KA2 | ICAM1 | ADCY3 |
| 147 | ETV4 | IL2RG | NME1 | OAS1 | BUB1B |
| 148 | POMC | FN1 | HSPA2 | TLE1 | NOTCH1 |
| 149 | PML | ASCL1 | PDGFRB | LPL | POLR2K |
| 150 | SOX9 | LEP | CXCL12 | DKK1 | JAG1 |
| 151 | GATA2 | AR | HIF1A | ZBTB16 | BUB1 |
| 152 | RXRA | EGFR | GPER1 | EGR2 | C3 |
| 153 | PRKCA | WT1 | FOSB | DUSP1 | DSN1 |
| 154 | PPARD | NR2F1 | CDKN2C | NR4A1 | AURKA |
| 155 | SUPT5H | FOSB | TEAD4 | IGF1R | HBB |
| 156 | ZNF263 | SAA1 | TP63 | RAC2 | CIITA |
| 157 | SRF | SOX2 | ADCY5 | CSF2RA | LMNB1 |
| 158 | TAF9 |  | TAF4B | ETV4 | LYN |
| 159 | IKBKB |  | EGR1 | CSF2RB | CTGF |
| 160 | SAP30 |  | PBX1 | CDC20 | CD8A |
| 161 | ERBB2 |  | LAMA3 | KLK3 | CENPU |
| 162 | SETD7 |  | NR2F2 | FOSB | LCK |
| 163 | BMP4 |  | SREBF1 | TGFB2 | CNR1 |
| 164 | RARB |  | CAV1 | EDN2 | PLCB1 |
| 165 | PTEN |  | NR4A2 | LEP | RAC2 |
| 166 | TRH |  | EBF1 | LIN28A | CENPE |
| 167 | GNAI1 |  | JUNB | FOXJ1 | KDR |
| 168 | HMGN3 |  | SIX5 | JAK3 | MYC |
| 169 | SMAD5 |  | BATF | CNR1 | CCNA1 |
| 170 | CCNA1 |  | DCN | AR | GNA15 |
| 171 | ANXA1 |  | IVL | GAL | ADIPOQ |
| 172 | MED13L |  | EDNRB | HBB | ESPL1 |
| 173 | TAC1 |  | LEF1 | MYB | BIRC5 |
| 174 | POLR3D |  | LYN | KLF4 | CBX4 |
| 175 | MAPK3 |  | GRM4 | BIRC3 | PIM1 |
| 176 | CDK1 |  | CAMK2B | IFI6 | SPI1 |
| 177 | ACTL6A |  | CXCL8 | SOCS1 | MAD2L1 |
| 178 | TNF |  | BCL11A | FASN | JUNB |
| 179 | CXCL8 |  | EDNRA | GATA2 | SERPINE1 |
| 180 | MMP1 |  | CDKN2A | SOX2 | LAMA3 |
| 181 | ZFP42 |  | NGFR | IRF4 | SFN |
| 182 | PDGFD |  | ADIPOQ | IL1B | COL18A1 |
| 183 | HDAC7 |  | TRH | PRKCB | DKK1 |
| 184 | HSPB1 |  | SNAI1 | IL12RB2 | CDCA8 |
| 185 | SGO2 |  | GATA2 | DCN | PLK1 |
| 186 | MYC |  | KLK3 | CD247 | TK1 |
| 187 | PDPK1 |  | NR2F1 | WNT5A | CHRM1 |
| 188 | RPS27A |  | FPR3 | NTS | FOXM1 |
| 189 | TLE1 |  | FABP4 | LCK | MYB |
| 190 | CCNB1 |  | CCKBR | CD40LG | CCR7 |
| 191 | S1PR3 |  | WNT5A | IGF2 | IRF4 |
| 192 | RBM5 |  | CCR1 | FABP4 | VAV1 |
| 193 | ICAM1 |  | NKX2-5 | NKX2-5 | NME1 |
| 194 | PABPC1 |  | COL1A2 | ZFP42 | RRM2 |
| 195 | ZEB1 |  | STAT1 | DUSP4 | CDC6 |
| 196 | GATA6 |  | TLE1 | SERPINA1 | CD36 |
| 197 | ADRA2A |  | KLF4 | ISG15 | CD40LG |
| 198 | F2R |  | MMP2 | CD3D | E2F1 |
| 199 | TCF7L2 |  | CXCL1 | GRP | CENPA |
| 200 | PDGFRB |  | FOXP3 | PAX5 | IL2RG |
| 201 | CHD1 |  | ZFP42 | CD3E | BMI1 |
| 202 | GRM4 |  | CHRM4 | CXCL8 | BTK |
| 203 | SMAD3 |  | KLF5 | ADCY1 | NDC80 |
| 204 | AGTR1 |  | FHL2 | EGR3 | CCNA2 |
| 205 | PTGER3 |  | TFAP2C | ADIPOQ | BRCA1 |
| 206 | NUP160 |  | SERPINA1 | IVL | CDCA5 |
| 207 | ESPL1 |  | CDK6 |  | UBE2C |
| 208 | IGF2 |  | FOSL1 |  | CEBPB |
| 209 | ELF1 |  | RUNX3 |  | POMC |
| 210 | CBX4 |  | CHRM1 |  | CD3G |
| 211 | CYCS |  | MMP9 |  | APLNR |
| 212 | ZKSCAN1 |  | PMAIP1 |  | DHFR |
| 213 | GRP |  | HOXB4 |  | KLF4 |
| 214 | LMNB1 |  | ADORA2B |  | EZH2 |
| 215 | BDKRB2 |  | LYZ |  | TCF4 |
| 216 | ADCY3 |  | CYR61 |  | CCNB2 |
| 217 | PGR |  | FGF2 |  | BIRC3 |
| 218 | TP53BP1 |  | APOA1 |  | KIF2C |
| 219 | RRM2 |  | GSTA1 |  | ADORA2B |
| 220 | BATF |  | DKK1 |  | CXCR6 |
| 221 | TGS1 |  | LEP |  | MCM8 |
| 222 | DNMT1 |  | SFTPD |  | DCN |
| 223 | GNA15 |  | CTGF |  | MMP2 |
| 224 | BMI1 |  | MYCN |  | RBL1 |
| 225 | H2AFX |  | OASL |  | VCAM1 |
| 226 | SMARCC2 |  | MX1 |  | CDC25A |
| 227 | EDNRA |  | ESRRG |  | E2F5 |
| 228 | APP |  | EDN3 |  | CD4 |
| 229 | MED16 |  | NTS |  | HCK |
| 230 | KLF2 |  | FOXJ1 |  | PMAIP1 |
| 231 | GSTA2 |  | CLU |  | SNAI1 |
| 232 | ADCY5 |  | PENK |  | ZEB1 |
| 233 | FOXJ1 |  | EGFR |  | IRS2 |
| 234 | SOCS1 |  | ACTN2 |  | GATA3 |
| 235 | LPAR2 |  | GNAO1 |  | ZNF217 |
| 236 | TEK |  | CD36 |  | AKT3 |
| 237 | TEAD2 |  | APOA2 |  | NPY |
| 238 | ZNF217 |  | CDH1 |  | ADCY5 |
| 239 | DCN |  | TNF |  | CDC45 |
| 240 | CAMK2B |  | CNR1 |  | AURKB |
| 241 | APOA2 |  | ALB |  | LEP |
| 242 | CASP7 |  | IRF4 |  | NPAS2 |
| 243 | TCF3 |  | AGT |  | PTAFR |
| 244 | KIT |  | WT1 |  | CHEK1 |
| 245 | CLASP2 |  | PAX5 |  | ESR1 |
| 246 | MDM2 |  | EPO |  | FPR1 |
| 247 | ADCY1 |  | LPAR3 |  | SYCP2 |
| 248 | TK1 |  | SAA1 |  | TYMS |
| 249 | NFKB2 |  | LIN28A |  | TGFB3 |
| 250 | JAK3 |  |  |  |  |
| 251 | RPS6KA3 |  |  |  |  |
| 252 | PCNA |  |  |  |  |
| 253 | BDP1 |  |  |  |  |
| 254 | SAA1 |  |  |  |  |
| 255 | HSF1 |  |  |  |  |
| 256 | VEGFA |  |  |  |  |
| 257 | LPAR3 |  |  |  |  |
| 258 | TLR2 |  |  |  |  |
| 259 | EDN1 |  |  |  |  |
| 260 | PRKDC |  |  |  |  |
| 261 | NR2F1 |  |  |  |  |
| 262 | DKK1 |  |  |  |  |
| 263 | RPS20 |  |  |  |  |
| 264 | HSPA2 |  |  |  |  |
| 265 | IVL |  |  |  |  |
| 266 | CDK19 |  |  |  |  |
| 267 | COL1A2 |  |  |  |  |
| 268 | NF1 |  |  |  |  |
| 269 | FLT3 |  |  |  |  |
| 270 | ALDOA |  |  |  |  |
| 271 | CDC6 |  |  |  |  |
| 272 | STAG3 |  |  |  |  |
| 273 | MX2 |  |  |  |  |
| 274 | LPAR1 |  |  |  |  |
| 275 | FYN |  |  |  |  |
| 276 | CHRM4 |  |  |  |  |
| 277 | LCK |  |  |  |  |
| 278 | THRA |  |  |  |  |
| 279 | CXCL12 |  |  |  |  |
| 280 | TOP2A |  |  |  |  |
| 281 | DUSP1 |  |  |  |  |
| 282 | TFAP2C |  |  |  |  |
| 283 | LEF1 |  |  |  |  |
| 284 | RPS16 |  |  |  |  |
| 285 | NR4A2 |  |  |  |  |
| 286 | TCF4 |  |  |  |  |
| 287 | RUNX1 |  |  |  |  |
| 288 | IL23A |  |  |  |  |
| 289 | PDGFB |  |  |  |  |
| 290 | HLA-A |  |  |  |  |
| 291 | CD14 |  |  |  |  |
| 292 | RBL2 |  |  |  |  |
| 293 | EGR3 |  |  |  |  |
| 294 | PTPN1 |  |  |  |  |
| 295 | NR2F2 |  |  |  |  |
| 296 | HOXB4 |  |  |  |  |
| 297 | NPY |  |  |  |  |
| 298 | MAFF |  |  |  |  |
| 299 | IRF4 |  |  |  |  |
| 300 | SFN |  |  |  |  |
| 301 | CCR1 |  |  |  |  |
| 302 | CXCR4 |  |  |  |  |
| 303 | EBF1 |  |  |  |  |
| 304 | MYCN |  |  |  |  |
| 305 | CD40 |  |  |  |  |
| 306 | IL6 |  |  |  |  |
| 307 | IFI35 |  |  |  |  |
| 308 | MMP9 |  |  |  |  |
| 309 | CCL2 |  |  |  |  |
| 310 | VCAM1 |  |  |  |  |
| 311 | IL2RG |  |  |  |  |
| 312 | ACTN2 |  |  |  |  |
| 313 | ASCL1 |  |  |  |  |
| 314 | HLA-B |  |  |  |  |
| 315 | FOXP3 |  |  |  |  |
| 316 | ISG20 |  |  |  |  |
| 317 | ZWINT |  |  |  |  |
| 318 | NPAS2 |  |  |  |  |
| 319 | IL1B |  |  |  |  |
| 320 | CASP1 |  |  |  |  |
| 321 | BIRC3 |  |  |  |  |
| 322 | TBX2 |  |  |  |  |
| 323 | PAX5 |  |  |  |  |
| 324 | FOS |  |  |  |  |
| 325 | CSF2RA |  |  |  |  |
| 326 | MITF |  |  |  |  |
| 327 | ETS1 |  |  |  |  |
| 328 | S1PR1 |  |  |  |  |
| 329 | HIF1A |  |  |  |  |
| 330 | NR1D1 |  |  |  |  |
| 331 | TFRC |  |  |  |  |
| 332 | EDNRB |  |  |  |  |
| 333 | ADIPOQ |  |  |  |  |
| 334 | HDAC9 |  |  |  |  |
| 335 | GPER1 |  |  |  |  |
| 336 | LAMA3 |  |  |  |  |
| 337 | MMP2 |  |  |  |  |
| 338 | KLK3 |  |  |  |  |
| 339 | ESRRG |  |  |  |  |
| 340 | ALB |  |  |  |  |
| 341 | CLU |  |  |  |  |
| 342 | IL18 |  |  |  |  |
| 343 | KLF4 |  |  |  |  |
| 344 | HBB |  |  |  |  |
| 345 | CSF2RB |  |  |  |  |
| 346 | CD36 |  |  |  |  |
| 347 | ADORA1 |  |  |  |  |
| 348 | SDC2 |  |  |  |  |
| 349 | APLNR |  |  |  |  |
| 350 | IRF7 |  |  |  |  |
| 351 | WNT5A |  |  |  |  |
| 352 | CXCR6 |  |  |  |  |
| 353 | CD247 |  |  |  |  |
| 354 | FOSB |  |  |  |  |
| 355 | NCAM1 |  |  |  |  |
| 356 | FABP4 |  |  |  |  |
| 357 | MX1 |  |  |  |  |
| 358 | APOA1 |  |  |  |  |
| 359 | SYCP2 |  |  |  |  |
| 360 | OASL |  |  |  |  |
| 361 | OXTR |  |  |  |  |
| 362 | TLR7 |  |  |  |  |
| 363 | GATA4 |  |  |  |  |
| 364 | SIX1 |  |  |  |  |
| 365 | SERPINA1 |  |  |  |  |
| 366 | LYZ |  |  |  |  |
| 367 | EDN3 |  |  |  |  |
| 368 | FGF2 |  |  |  |  |
| 369 | CD3D |  |  |  |  |
| 370 | TP63 |  |  |  |  |
| 371 | LIN28A |  |  |  |  |
| 372 | EDN2 |  |  |  |  |
| 373 | PENK |  |  |  |  |
| 374 | NTS |  |  |  |  |
| 375 | SOX2 |  |  |  |  |
| 376 | WT1 |  |  |  |  |
