# Supplementary material for: RNA-Seq-Based Breast Cancer Subtypes Classification Using Machine Learning Approaches
Source: Comput Intell Neurosci. 2020 Oct 29;2020:4737969. doi: 10.1155/2020/4737969 (PMC7644310; doi:10.1155/2020/4737969)
Supplement: Supplementary Materials — Figure S1: heatmap for Her2 and non Her2 groups. The left group 1 represents the Her2 group and the right group 2 denotes the non-Her2 group. Figure S2: heatmap for LumA and non-LumA groups. The left group 1 represents the LumA group and the right group 2 denotes the non-LumA group. Figure S3: heatmap for LumB and non-LumB groups. The left group 1 represents the LumB group and the right group 2 denotes the non-LumB group. Figure S4: heatmap for Normal-like and non-Normal-like groups. The left group 1 represents the Normal-like group and the right group 2 denotes the non-Normal-like group. S1 File: the detailed information of weighted DEGs for classification. S2 File: the detailed information of weighted DEGs for GO enrichment analysis. S3 File: the detailed enriched GO terms results for Basal-like subtype. S4 File: the detailed enriched GO terms results for Her2 subtype. S5 File: the detailed enriched GO terms results for LumA subtype. S6 File: the detailed enriched GO terms results for LumB subtype. S7 File: the detailed enriched GO terms results for Normal-like subtype. [file 4737969.f1.zip › supplementary materials/S2 File.docx]

**S2 File:** The detailed information of weighted DEGs for GO enrichment analysis.

| **No.** | **Basal-like** | **Her2** | **LumA** | **LumB** | **Normal-like** |
| --- | --- | --- | --- | --- | --- |
| 1 | FOXA1 | GRB7 | CENPN | ESPL1 | SPRY2 |
| 2 | BCL11A | FGFR4 | CENPA | PRIM1 | TP63 |
| 3 | RGMA | ERBB2 | CCNB2 | CCNE2 | NGFR |
| 4 | GATA3 | TCAP | MYBL2 | RACGAP1 | COL17A1 |
| 5 | VGLL1 | PSMD3 | ORC6 | DSN1 | RHOJ |
| 6 | CCNE1 | RARG | KIF2C | CDC25C | SERPINF2 |
| 7 | CDCA7 | GGT1 | CDC20 | DTL | PDGFA |
| 8 | RHOB | MMP1 | CCNA2 | CENPE | OXTR |
| 9 | GABBR2 | LBP | CDC45 | AURKA | CXCL2 |
| 10 | PRKX | IGF1R | PLK1 | ZWINT | DMD |
| 11 | KCNQ4 | S100A7 | AURKA | NEK2 | PIK3C2G |
| 12 | AR | HDAC5 | ORC1 | DHFR | PROS1 |
| 13 | IL12RB2 | EZH1 | CDCA8 | FANCI | EDN3 |
| 14 | CENPA | RRM2 | UBE2C | CDC6 | HOXA5 |
| 15 | YBX1 | DNMT3B | CDC25A | SGO1 | CX3CL1 |
| 16 | AURKB | CBR3 | SKA1 | GSN | FGF2 |
| 17 | CTPS1 | CBX2 | SGO1 | BRCA1 | AK5 |
| 18 | CDC20 | PYY | BIRC5 | CENPU | HLF |
| 19 | KCNG1 | PIP5K1B | FOXM1 | BUB1B | ALDH1A3 |
| 20 | FABP5 | ELP2 | BUB1 | KNTC1 | RELN |
| 21 | FANCE | IDI1 | HJURP | CCNB1 | EGR2 |
| 22 | CDCA8 | DUSP10 | CDCA5 | KIF18A | EDNRB |
| 23 | TSC22D3 | CDC6 | MAPT | TOP2A | S100B |
| 24 | GAL | PMAIP1 | AURKB | PLK4 | MYH11 |
| 25 | CDC25A | SMAD4 | NDC80 | GSTP1 | TCF7 |
| 26 | ORC1 | IL6ST | CHEK1 | MAD2L1 | NPR1 |
| 27 | PUM3 | NPY5R | KDM4B | GDF5 | EPHA2 |
| 28 | TAF4B | BCL2 | RAD51 | BRIP1 | IL3RA |
| 29 | SKP2 | CGN | KPNA2 | DEFB1 | KIT |
| 30 | TCF7L1 | MED28 | CTPS1 | EDN3 | PTGER4 |
| 31 | IL6ST | TRH | RRM2 | SKA1 | DEFB1 |
| 32 | LRP8 | MED13L | PTTG1 | RAD51 | KRT14 |
| 33 | NDC80 | PAX7 | CENPO | VGLL1 | S1PR1 |
| 34 | RARA | VAMP2 | KIF18A | SKA2 | ID1 |
| 35 | CXCL5 | SULT1E1 | E2F2 | SLC9A3R1 | EPHB1 |
| 36 | WNT6 | MYC | BLM | CDK1 | NRG1 |
| 37 | RNF8 | MED24 | BUB1B | BIRC5 | PLPP3 |
| 38 | E2F3 | ALOX15B | MAD2L1 | FANCD2 | MMRN1 |
| 39 | VAV3 | MED1 | APH1B | MRPS23 | LDB2 |
| 40 | CCKBR | STAT5B | MCM6 | PSMD12 | ACVRL1 |
| 41 | CHEK1 | MVK | IL6ST | CX3CL1 | FCER1A |
| 42 | GDF5 | WNT3 | CCNE1 | HELLS | LMO2 |
| 43 | KIF2C | KANSL1 | CDK1 | ECT2 | EGFR |
| 44 | NCK2 | ESR1 | CENPE | LMNB1 | SGK1 |
| 45 | SKP1 | CHN1 | ESR1 | CENPQ | ETS2 |
| 46 | LPIN1 | IRS1 | EZH2 | SPRY2 | DLL1 |
| 47 | PAPSS1 | DKK1 | NUF2 | NUF2 | BCL11A |
| 48 | SAR1B | ITGB6 | OIP5 | CRHR1 | CAV1 |
| 49 | ORC6 | PSMD5 | CCNB1 | UBE2C | GSTM5 |
| 50 | CENPN | UGT2B11 | CKS1B | CPT1A | PLK3 |
| 51 | KDM4B | FGG | CDC25B | POLR2K | MYLK |
| 52 | MARCO | DDC | E2F1 | POLE2 | HBEGF |
| 53 | CEBPB | P2RY6 | BCL2 | OIP5 | EDN1 |
| 54 | SKA1 | SDC1 | ERBB4 | CCNA2 | NTRK2 |
| 55 | MED30 | RBBP8 | MCM2 | WNT6 | CYGB |
| 56 | BHLHE40 | KAT2A | DBF4 | PCNA | C4BPA |
| 57 | MAGOH | STAT6 | LRP8 | WDHD1 | SAA1 |
| 58 | RIPK2 | KDM4B | RFC4 | E2F1 | RGS2 |
| 59 | LMO4 | TUBG2 | LMNB1 | KPNA2 | KRT5 |
| 60 | HJURP | OPRL1 | TUBA1C | TRIP10 | TF |
| 61 | NEK9 | FABP6 | SKP2 | CXCL2 | NDN |
| 62 | SFRP1 | HMGCS1 | MCM4 | ETS2 | SNAI2 |
| 63 | COL9A3 | BCAN | RANBP1 | SGO2 | ACTA2 |
| 64 | FOXP1 | B3GNT3 | PGR | ANXA1 | NOTCH4 |
| 65 | POLR1E | PDE6B | CDC6 | MCM8 | SFRP1 |
| 66 | CBX2 | IVL | ESPL1 | MTDH | TGFBR2 |
| 67 | HMGA1 | CHPT1 | SPC24 | FZD7 | GNG7 |
| 68 | NUP93 | SRD5A1 | FBXO5 | NSF | INPP5D |
| 69 | GTPBP4 | FGFR2 | CENPF | ASF1B | TGFB1I1 |
| 70 | ENPP1 | TLE1 | CDT1 | LEFTY2 | RARB |
| 71 | PDE9A | ALDH3B2 | GATA3 | WWTR1 | TPM3 |
| 72 | ETV6 | HIST1H4H | CDC7 | WWP1 | FLI1 |
| 73 | KLF5 | ME1 | NUP93 | S100B | GSN |
| 74 | E2F2 | NKX2-2 | CLSPN | EPHA2 | TWIST2 |
| 75 | RFC4 | NAB2 | RACGAP1 | RRM2 | PTGS2 |
| 76 | TADA2B | EGLN3 | MYB | CLSPN | FGF7 |
| 77 | CDC45 | AURKA | DNMT3B | FFAR2 | FOXO1 |
| 78 | S100B | HOXB13 | RHOB | PTPN1 | JAK3 |
| 79 | UQCRH | ING4 | CALCOCO1 | RGMA | DUSP7 |
| 80 | CKS1B | BRAF | FEN1 | TCF7L1 | MET |
| 81 | ODC1 | GDF5 | DKC1 | FBXO5 | FGF1 |
| 82 | PLK1 | IKZF3 | GTPBP4 | RBL1 | ANGPTL4 |
| 83 | CCNB2 | RPL19 | SIRT3 | CCNB2 | EGR3 |
| 84 | PRLR | AKT1 | YBX1 | DMD | LAMB3 |
| 85 | CA9 | TLE3 | CBX2 | COL16A1 | MKL1 |
| 86 | FOXM1 | NME5 | TFF3 | CLTC | B3GNT3 |
| 87 | HEXIM1 | GSR | MCM7 | MRPL13 | MAFF |
| 88 | MMP7 | HIF1A | GMPS | BUB1 | KRT17 |
| 89 | SERBP1 | SLC25A5 | PLK4 | MYBL2 | CCL21 |
| 90 | CDT1 | CDON | NCAPG2 | CDCA5 | PIK3CD |
| 91 | PIM1 | ADCY9 | SPDL1 | C4BPA | DUSP2 |
| 92 | ESR1 | GALNT3 | TYMS | SPC24 | CD40 |
| 93 | MYB | ORC6 | RIPK2 | EGFR | ANXA1 |
| 94 | CDKN2A | CDC45 | H2AFZ | PDE9A | MYCT1 |
| 95 | TGFB3 | CDK8 | CHEK2 | MFGE8 | EBF1 |
| 96 | WWP1 | PPA1 | CHRD | CCNF | NDRG2 |
| 97 | BLM | CALCOCO1 | LAMB2 | BCL11A | A2M |
| 98 | TLE3 | MYCN | PSMB2 | EFNB1 | CCL28 |
| 99 | PLCG2 | EPO | TADA2B | PLK1 | HOXA9 |
| 100 | UGCG | SHMT2 | FOXA1 | TCF7L2 | NPY2R |
| 101 | EZH2 | CST3 | NME5 | NDRG2 | RGMA |
| 102 | NUF2 | EDN3 | HMGA1 | PTGS2 | PRKCA |
| 103 | HSD17B4 | COMT | CCT5 | NCAPG2 | PDE9A |
| 104 | BYSL | UCP2 | XPO5 | CDC45 | ZYX |
| 105 | BUB1 | CXCL8 | CDCA7 | GNG7 | S1PR2 |
| 106 | NIP7 | GPI | SGO2 | CD82 | CXCL1 |
| 107 | NOP2 | SMARCD3 | PSMD7 | SFRP1 | ITGA9 |
| 108 | EPHB1 | POT1 | KCND3 | PTTG1 | NUP210 |
| 109 | GCNT2 | SLC27A1 | ENO1 | LAMB3 | LPAR6 |
| 110 | IFNAR2 | CXCL11 | NIP7 | FKBP4 | CDH5 |
| 111 | CHEK2 | CENPN | ZWINT | RBMS1 | RUNX3 |
| 112 | FAF1 | CRYZ | INCENP | TUBB6 | ETV5 |
| 113 | ENO1 | IKBKB | SLC27A1 | FES | PARP1 |
| 114 | PLA2G4A | MAP3K5 | TK1 | ESR1 | SMG7 |
| 115 | XPO5 | IRS2 | TAC1 | CXCL1 | EGR1 |
| 116 | MFGE8 | NDRG2 | CDC25C | RAD51C | DUSP6 |
| 117 | CCNA2 | ZWINT | RBL1 | NDC80 | EFNB3 |
| 118 | PSMB2 | MYBL2 | TGFB3 | JDP2 | IL6 |
| 119 | NOTCH1 | TRIB3 | FANCI | STAT5A | TLR2 |
| 120 | ZBTB7A | ADM | ASF1B | RAD21 | FOSL1 |
| 121 | CDK6 | UGCG | AR | ZFP36L1 | TRPC6 |
| 122 | SLC9A3R1 | LAMB2 | GMNN | HJURP | BIN1 |
| 123 | LYN | CBX7 | UTP4 | ITGB4 | CYR61 |
| 124 | BTF3 | GSTM2 | TLE3 | C3 | SRP9 |
| 125 | RANBP1 | TAC1 | CENPM | RELB | GAS1 |
| 126 | DKC1 | HDAC11 | PCNA | YWHAZ | ALDH1A1 |
| 127 | ZFYVE16 | SGO1 | CBX7 | FOXM1 | FAS |
| 128 | MCM5 | MGP | FANCD2 | BLM | LPL |
| 129 | PRKAG1 | SCD | GAMT | CENPM | CD79B |
| 130 | FOSL1 | ADM2 | MRPL15 | CENPF | ENG |
| 131 | MCM7 | TGFBR1 | GFRA1 | FGF2 | CBX7 |
| 132 | ERBB3 | FGB | MCM3 | EGR1 | MEF2C |
| 133 | EPHB3 | NEDD4L | MCM5 | LAMC2 | PENK |
| 134 | NMB | ABCA3 | TOP2A | SMO | CCL19 |
| 135 | DUSP7 | ASS1 | PRKAB1 | COL18A1 | IL4R |
| 136 | PTPN2 | INSR | PDGFD | CYR61 | COL4A6 |
| 137 | ACVR1B | PCM1 | ZBTB16 | JUNB | PLA2G4A |
| 138 | EFNA5 | TNNC1 | DSN1 | BMP2 | FOSB |
| 139 | SMO | UGT2B7 | NEK2 | GCNT2 | MAF |
| 140 | UTP4 | FGD1 | HSPD1 | NCK2 | MTA3 |
| 141 | CENPO | CXCL9 | VRK1 | MCM2 | IFNGR1 |
| 142 | RUNX3 | ARHGEF2 | PTGER3 | LMNA | COL14A1 |
| 143 | CDCA5 | NAMPT | BTRC | RRM2B | ALB |
| 144 | IRAK1 | ADCY3 | CISH | PLCD1 | CCND3 |
| 145 | PTTG1 | BAG1 | TFF1 | TK1 | PYGO2 |
| 146 | TUBB6 | AQP9 | HEXIM1 | LTF | ELMO1 |
| 147 | MRPL2 | PLCB4 | SF3B3 | KRT17 | TUBB6 |
| 148 | EGFR | FOXO4 | UBE2S | ITGA6 | EPAS1 |
| 149 | ITGB8 | CDKN1B | BHLHE40 | ALDH1A3 | ZBTB16 |
| 150 | PHF5A | ARHGEF25 | RCC1 | VIM | INSL3 |
| 151 | COL27A1 | CCNE1 | PSME4 | GAS6 | BMP2 |
| 152 | CCNH | HJURP | GLI3 | WNT11 | FGR |
| 153 | WDR77 | PFKP | BTF3 | LIF | BUB3 |
| 154 | MSN | DOCK1 | ALYREF | EPHB1 | CDKN1C |
| 155 | COL22A1 | CDK1 | UGCG | CAV1 | EFNB1 |
| 156 | NCOA7 | MRPL27 | SHC2 | TPM2 | PDGFRA |
| 157 | LSM2 | BTRC | SHMT2 | FAT1 | ATF3 |
| 158 | MRPS5 | STAT5A | RHOBTB2 | NOTCH1 | ADH1B |
| 159 | TEX10 | EGLN2 | SNRPA1 | APP | WLS |
| 160 | ITGB5 | CAMP | HELLS | PTGFR | SRF |
| 161 | TYMS | FOXP3 | GABBR2 | GPX7 | NDUFS2 |
| 162 | UBE2C | ORC1 | SNRPG | CENPA | MYL9 |
| 163 | GRPR | DOK1 | CENPU | FEN1 | NR2F1 |
| 164 | GSTP1 | ACADS | MSH6 | NPAS2 | WNT5B |
| 165 | EID1 | LRP6 | DCAF13 | BMP6 | FKBP4 |
| 166 | CXCL1 | GRPR | PNPLA2 | COL27A1 | TPM2 |
| 167 | SF3A3 | HHEX | AGO2 | ITGB8 | NPR2 |
| 168 | TNFRSF21 | CENPA | UQCRH | JUN | GAS6 |
| 169 | SRD5A1 | CCND1 | GAPDH | RARB | NCOA7 |
| 170 | CDC7 | APH1B | CCNE2 | TRIP6 | EFNB2 |
| 171 | SGO1 | NPY1R | NUMA1 | OAS3 | FZD7 |
| 172 | DUSP5 | CCNB2 | WDHD1 | CDC7 | RRM1 |
| 173 | ITPR1 | POLI | COL14A1 | CDKN1C | FYN |
| 174 | RHEB | WWP1 | MAP3K1 | SAA1 | F2RL1 |
| 175 | SIX3 | APBB1 | MXD4 | POMC | RBBP5 |
| 176 | CEBPG | CCNE2 | MIS18A | LMO4 | NOS3 |
| 177 | LSM6 | PHB | VAMP2 | ID1 | MFGE8 |
| 178 | RPF2 | SMAD3 | RFC3 | PLCG2 | BCL9 |
| 179 | HNRNPH2 | TFRC | FANCE | BHLHE41 | BMP6 |
| 180 | DBF4 | ENO1 | CST3 | SMC4 | PLCG2 |
| 181 | MSX2 | TKT | MED30 | PROS1 | VWF |
| 182 | PFKP | CLSPN | TCP1 | SCD | CSF2RB |
| 183 | CISH | SNAPC2 | HDAC2 | TNNI2 | COL7A1 |
| 184 | BAK1 | COL27A1 | CYCS | CXCL5 | TNNI2 |
| 185 | SNRPA1 | KCNC2 | ITPR1 | NME1 | BHLHE41 |
| 186 | GPX7 | MYH14 | E2F3 | KIF2C | TOP2A |
| 187 | TEAD4 | WNT2 | GRPR | MET | SUN2 |
| 188 | ERBB4 | TK1 | DTL | ORC6 | VGLL1 |
| 189 | TFF3 | EYA1 | PSMA7 | MYBPC1 | CCT3 |
| 190 | PSME4 | LAMA3 | TBX3 | KRT5 | MT2A |
| 191 | MRPL15 | EPHB4 | DHCR7 | PIK3C2G | SOCS2 |
| 192 | POLR1C | SIRT3 | BYSL | NGFR | JAG2 |
| 193 | DGAT2 | PFKM | RORC | CYP7B1 | NEK2 |
| 194 | NCK1 | RAD51 | LIN9 | SPHK1 | GNG2 |
| 195 | MED7 | GPER1 | MSH2 | CENPN | ARHGAP26 |
| 196 | TINF2 | APOA1 | NOP2 | LPAR3 | KCNB1 |
| 197 | IL13RA1 | LPAR3 | ADCY9 | AURKB | EBI3 |
| 198 | ADCY9 | FGF2 | ECT2 | PIK3CD | TEK |
| 199 | LEO1 | KPNA2 | FOXP1 | CDCA8 | GPX3 |
| 200 | GLI3 | WNT4 | GSTM2 | ZNF521 | PIK3R1 |
| 201 | NUMA1 | MAPT | FCER1A | CDC25A | CTSG |
| 202 | THPO | GFRA1 | POLI | C1S | ECT2 |
| 203 | TP53BP2 | CYP2A6 | MYH11 | TF | EPHA4 |
| 204 | XAGE2 | GAB2 | SRSF5 | DLL1 | CCNB1 |
| 205 | DAPK1 | COL2A1 | ADRA2A | GAS1 | POLR3K |
| 206 | MYBL2 | NTRK2 | THPO | BIN1 | KDM5B |
| 207 | S1PR2 | FCER1A | STIP1 | FZD10 | LIF |
| 208 | USP1 | WNT7B | CDK8 | RET | GNAI1 |
| 209 | TLX1 | CDC25A | FGF10 | CD79B | TADA1 |
| 210 | BIRC5 | CHRM1 | ODC1 | PRKCA | MMP7 |
| 211 | MAML3 | BUB1 | DOK1 | MCM4 | VIM |
| 212 | APH1B | TGFBR3 | IKBKB | NPY2R | RBMS1 |
| 213 | HDAC2 | PRKAA1 | IL12RB2 | FCER1A | CD14 |
| 214 | BCL2L1 | DUSP6 | VIPR1 | IL27RA | CENPF |
| 215 | NFKBIE | PSIP1 | VAV3 | RPS6KA2 | DTL |
| 216 | CYP17A1 | GJA1 | ZWILCH | TWIST2 | PTGFR |
| 217 | PPP1CB | MMP13 | EZH1 | SGK1 | SKI |
| 218 | NOL10 | IL2RA | DUSP4 | TP63 | TUBB2B |
| 219 | PPP2CA | MGST1 | BRIP1 | TNNT3 | EPRS |
| 220 | GPC2 | CENPU | ITGB5 | ETV5 | SATB1 |
| 221 | LIN9 | SFTPD | MCM8 | WNT5B | CYP7B1 |
| 222 | NCAN | E2F2 | AGTR1 | DES | PPARG |
| 223 | KIF18A | KCNF1 | YWHAZ | TPM1 | PRKCB |
| 224 | DHCR24 | LIN28A | AQP9 | CXCL16 | CASP1 |
| 225 | ADORA2B | FASN | TSC22D3 | FOSL1 | SDC3 |
| 226 | PBX1 | CDCA5 | DOCK1 | MYLK | PLD1 |
| 227 | PGM1 | GABBR1 | SRD5A1 | COL9A3 | P2RY13 |
| 228 | PFDN2 | CLTC | ERBB3 | KCNG1 | TFB2M |
| 229 | MCM6 | CYP2J2 | IRS1 | NFIB | GPX2 |
| 230 | MSH2 | JAG1 | MAML3 | PLA2G4A | SERPING1 |
| 231 | CCL20 | UBE2C | COL4A5 | NFATC1 | SULT1E1 |
| 232 | AGO2 | ACACB | PABPC1 | WNT10A | TAC1 |
| 233 | CXCL16 | ITPR2 | IGF1R | GATA3 | PTH1R |
| 234 | SOD2 | KIF2C | H2AFX | SERPINF2 | CCND2 |
| 235 | BCL2A1 | GSTA2 | HSD17B4 | CTGF | TGFBR3 |
| 236 | RCC2 | NFATC1 | LPIN1 | ENPP1 | HLA-E |
| 237 | PRKAB1 | ECT2 | SSTR1 | CGA | ZWINT |
| 238 | SF3B6 | ADCY1 | GPI | SOCS3 | TNFSF12 |
| 239 | TBX3 | CXCL10 | TNFSF12 | TYMS | DLL4 |
| 240 | UBE2S | PROCR | TUBA1B | E2F2 | FES |
| 241 | VIPR1 | TEAD1 | RUNX1 | FOXA1 | DUSP1 |
| 242 | NFIB | BUB1B | NUP205 | ALDH3A1 | RPS6KA3 |
| 243 | MRPL9 | GATA3 | CTSG | HOXA5 | PDLIM7 |
| 244 | SEH1L | PLAU | MMRN1 | A2M | MAZ |
| 245 | NDRG1 | GUCY1A2 | CEBPB | KIT | ZNF521 |
| 246 | PLD1 | CCNA2 | PYY | PLEKHG5 | FABP4 |
| 247 | TAF9B | DHCR7 | NDRG1 | EDN1 | CXCL12 |
| 248 | MCM3 | CDC20 | FMOD | TFF1 | RHOU |
| 249 | CRNKL1 | MAOB | ABCA3 | LOR | GGT5 |
| 250 | GAMT | CCNB1 | MUC1 | COL9A1 | IRF8 |
| 251 | NMU | GLI3 | SNAPC2 | FZD1 | TBX2 |
| 252 | OXGR1 | GPC2 | GRP | COL17A1 | CARD11 |
| 253 | ADCY6 | GNA14 | BNIP3L | RHOJ | RACGAP1 |
| 254 | INCENP | LRP8 | LAMA2 | ETS1 | ADAR |
| 255 | TAF11 | PDCD4 | KLF2 | SOX9 | HSPA4 |
| 256 | NKX2-5 | GZMB | ADCY1 | AK5 | PBX1 |
| 257 | DMD | LMNB1 | NMU | NPR1 | RPL13 |
| 258 | IL27RA | P2RY2 | ENTPD8 | RGS2 | PLCB2 |
| 259 | RNASEL | PTGER3 | AK5 | COL14A1 | PIP5K1A |
| 260 | STAM2 | F5 | COL4A6 | IL3RA | CDC25C |
| 261 | CAD | AFDN | PUM3 | PLD1 | CSF2RA |
| 262 | FZD7 | CA9 | RARA | FYN | RPL4 |
| 263 | GATAD2A | ITGA9 | GPC2 | RELN | CCNF |
| 264 | TFDP1 | SYT1 | PRLR | RUNX3 | AHCTF1 |
| 265 | SREBF1 | CEBPD | GSTM3 | NPY | DES |
| 266 | NRIP1 | FFAR2 | TKT | NPR2 | UCHL5 |
| 267 | CYP7B1 | EFNB3 | CITED1 | EXOC2 | POU2AF1 |
| 268 | RAD51 | IRF4 | GAL | KRT14 | IL7R |
| 269 | FAM13B | ALDH3A2 | IGF2 | RARA | COL16A1 |
| 270 | XPC | CD44 | ACACB | PBX1 | RUVBL1 |
| 271 | ALYREF | BGLAP | PCM1 | HBEGF | NR4A1 |
| 272 | SNRPD1 | EGR3 | PER2 | IGFBP3 | RPL5 |
| 273 | SSRP1 | LEFTY2 | CDON | TEAD2 | AOX1 |
| 274 | CDK8 | GAS6 | SMC4 | MAFF | RPS25 |
| 275 | MAPT | SDC2 | NME3 | PENK | CCL2 |
| 276 | UPF3B | OIP5 | PDE6B | GGT5 | IL27RA |
| 277 | MCM2 | FHL2 | F2RL2 | ASS1 | NCF4 |
| 278 | NOP58 | LRP2 | CYP17A1 | TCF7 | PDGFD |
| 279 | VPS72 | E2F1 | PRKX | COL6A2 | CD3E |
| 280 | CSNK2A2 | HIST1H2BJ | IFITM2 | MYH11 | CRNKL1 |
| 281 | GART | BAG4 | EPOR | ORC1 | KPNA2 |
| 282 | BID | CYP2A13 | RIPK3 | GFRA1 | PRIM1 |
| 283 | SPC24 | HK2 | GAS6 | ITGA9 | BCL6 |
| 284 | WNT10A | PLCD4 | KNTC1 | MMP7 | ETS1 |
| 285 | CCND1 | PGR | POLE2 | NTRK2 | NUP155 |
| 286 | MAPK9 | FZD4 | MRPL13 | HLF | GPR4 |
| 287 | MFAP1 | BHLHE40 | ARHGEF3 | UGT2B7 | LIFR |
| 288 | UBA2 | CDC25B | MAOA | CEBPD | ARHGEF25 |
| 289 | MGST2 | CTLA4 | AZGP1 | FOS | ACTN1 |
| 290 | POLD1 | ISG20 | MYCT1 | IL6 | RPL11 |
| 291 | KITLG | GNA15 | DDC | FAS | LOR |
| 292 | SLC9A1 | HIST1H4I | CCNF | CCKBR | KNTC1 |
| 293 | RAD17 | PLK1 | GHRH | PTH1R | TUBG1 |
| 294 | SF3B3 | CD79A | EIF4EBP1 | GNA15 | FZD1 |
| 295 | SPOP | ITGB8 | USP1 | GADD45A | DOK2 |
| 296 | KDM1A | GAMT | IDI1 | ASCL1 | IGF2 |
| 297 | PLIN2 | COL9A1 | ELP2 | CCNA1 | HEY1 |
| 298 | EPOR | ASF1B | NOTCH4 | TRH | TNNT3 |
| 299 | BCL2 | KIF18A | PDCD4 | S1PR1 | CKAP5 |
| 300 | ENTPD8 | HIST1H3J | LRP2 | GSTA1 | MAFB |
| 301 | DUSP4 | ENTPD8 | APBB1 | NRG1 | WNT6 |
| 302 | MET | FPR3 | ME1 | NCOA7 | NFIB |
| 303 | SH3KBP1 | GSTO2 | NEDD4L | DUSP6 | MAP3K5 |
| 304 | XPA | NPY | MMP1 | CDK6 | RPL34 |
| 305 | GAPDH | GNAZ | EGR3 | WLS | FANCF |
| 306 | PPM1A | SPC24 | TEK | CYGB | NR3C1 |
| 307 | PLXNA1 | AGTR1 | MSX2 | RPS6KA3 | HMGCS2 |
| 308 | SNRPG | TNNT3 | CENPH | SULT1E1 | S1PR4 |
| 309 | BTRC | POU2AF1 | NDUFB9 | FZD8 | KLF2 |
| 310 | MCM4 | NPR2 | VIPR2 | CYBA | ERBB3 |
| 311 | PER2 | LIFR | MARCO | MAPK11 | FANCI |
| 312 | CENPF | RIPK3 | LPAR6 | GNAI1 | STAT5A |
| 313 | E2F1 | PLA2G2A | LDB2 | ACTA2 | NCAM1 |
| 314 | BECN1 | TLE2 | NFATC4 | CHRM1 | CDK1 |
| 315 | TFF1 | HIST1H2BK | IRAK1 | PIM1 | GADD45A |
| 316 | AURKA | FZD7 | PFKP | TGFB1I1 | PLA2G2A |
| 317 | BUB1B | TCEA3 | TCEA3 | INPP5D | ZAP70 |
| 318 | EIF4B | TBX3 | KCNC2 | ADCYAP1 | MYO6 |
| 319 | LNPEP | FES | HHEX | SIX1 | RBBP7 |
| 320 | TAF1D | IL4I1 | ARHGAP35 | SIX5 | LAMA2 |
| 321 | RBL1 | CITED1 | GSTA2 | GATA6 | MRPL24 |
| 322 | DNMT3B | BMP2 | ADCY6 | EBI3 | JUN |
| 323 | CDC25B | ISYNA1 | CA9 | P2RY13 | NSF |
| 324 | RCC1 | LYZ | EGLN2 | CCL21 | NRP1 |
| 325 | NSA2 | PDGFD | PLIN2 | HSPB1 | KLF5 |
| 326 | FBL | HBB | NRIP1 | TNC | RPS6KA2 |
| 327 | MAP3K1 | UCP1 | KCNG1 | COL6A1 | RFC5 |
| 328 | NMI | CTF1 | FOS | KLF5 | IGFBP3 |
| 329 | SENP3 | MYB | GBP1 | TGFBR2 | SGO1 |
| 330 | WWTR1 | ARHGEF6 | ENPP1 | TFF3 | LY96 |
| 331 | RAB11A | GPX2 | WDR31 | ANGPTL4 | ZEB2 |
| 332 | GNA14 | CXCR6 | PFDN2 | NMB | RPL3 |
| 333 | PLAUR | COL16A1 | NDN | CDT1 | PRKCD |
| 334 | RORC | BMP5 | ZEB1 | NCAM1 | MMP19 |
| 335 | COL9A1 | ZNF521 | VWF | TNNT1 | BMP5 |
| 336 | GSTM3 | PTTG1 | BAG1 | OSMR | RAD21 |
| 337 | OIP5 | HSPA6 | NTRK2 | SYCP2 | CD247 |
| 338 | AZGP1 | RHOV | CFD | ATF3 | SSTR1 |
| 339 | GFM2 | NPY2R | PLIN1 | CDH1 | MAPK11 |
| 340 | GMPS | CENPE | TNFRSF21 | MYL9 | SNRPE |
| 341 | GMNN | ENO3 | GNG12 | CD44 | RPL21 |
| 342 | RELB | HBA2 | DUSP1 | MMP2 | CD82 |
| 343 | FBXO5 | AURKB | POLD4 | SNAI2 | RPS17 |
| 344 | NFIA | ASPH | DLC1 | ITGB6 | CXCL5 |
| 345 | ZBTB16 | CDCA8 | IL2RA | GATA4 | MRPL13 |
| 346 | NFIC | CDT1 | NPY1R | B3GNT3 | SGO2 |
| 347 | CBFB | S100B | RAD21 | EFNA5 | SOCS3 |
| 348 | YES1 | NFATC4 | COL16A1 | AKT3 | MYBPC1 |
| 349 | SIRT3 | CDH1 | BMP4 | ERBB4 | OIP5 |
| 350 | POLI | BIRC5 | E2F5 | TAC1 | ITGB3 |
| 351 | KIF3B | MMP3 | TUBB4A | CCND1 | COL15A1 |
| 352 | IGF1R | IFITM2 | P2RY6 | FST | LPAR3 |
| 353 | SPHK1 | CAV1 | RHOH | ADORA2B | TLR4 |
| 354 | RPL39L | HIST1H4B | WWP1 | GDF15 | CCNE2 |
| 355 | PSMB4 | CCKBR | CXCL10 | SYT1 | WWTR1 |
| 356 | IFITM2 | ZP3 | HBB | IFIT1 | FANCD2 |
| 357 | AP2B1 | KCNG1 | HMGCS2 | TRAF1 | TRIP10 |
| 358 | EPHA2 | MAOA | GSC | PDGFRA | MCM2 |
| 359 | CALCOCO1 | COL11A1 | THRB | NTF3 | IKZF1 |
| 360 | CDKN2C | CDCA7 | KCNQ4 | INSL3 | FOS |
| 361 | SPDL1 | HTR3A | TUBG2 | ADRB1 | ASF1B |
| 362 | GDI2 | SERPINF2 | GDF15 | GABBR2 | WDHD1 |
| 363 | CHRAC1 | BMPR1B | TFRC | TLR2 | GBP2 |
| 364 | KRT17 | TF | HDAC11 | ICAM1 | TNFAIP3 |
| 365 | MAD2L1 | IGFBP1 | FST | SATB1 | TMEM173 |
| 366 | CLSPN | FGF10 | UCP1 | CTSG | RPS9 |
| 367 | PTGS2 | RGMA | WLS | MMP14 | DLC1 |
| 368 | BMP2 | CD82 | BCL2A1 | IRAK3 | CDK6 |
| 369 | MSH6 | IFITM1 | GNA14 | OAS1 | SRGN |
| 370 | KIF3A | GNG7 | GJA1 | HOXA9 | CEBPD |
| 371 | IRS1 | INHBB | S1PR1 | H2AFY2 | ALDOA |
| 372 | RB1 | CCR2 | NFIA | MMRN1 | LAMC2 |
| 373 | ZPR1 | CXCL13 | PLCD4 | NPY5R | GSTA1 |
| 374 | AFF4 | MYH11 | MED13L | GPX3 | SOCS1 |
| 375 | HMGCS2 | PDE4B | DUSP16 | CCL19 | USP14 |
| 376 | SRP14 | WNT11 | CCND1 | TLE1 | NUF2 |
| 377 | GSTA1 | FGFR3 | LIFR | PIP5K1B | NFATC2 |
| 378 | CENPE | NMU | SFRP2 | LPL | ITGB4 |
| 379 | GDF15 | ADCYAP1 | BBC3 | RHOV | TCF7L2 |
| 380 | SLC9A3R2 | NRIP1 | TLX1 | DAPP1 | CTPS2 |
| 381 | CALR | CYP17A1 | RHOJ | VIPR2 | NTF3 |
| 382 | RPS7 | ANPEP | ACADS | FMOD | CSF1R |
| 383 | PRDX3 | CRABP2 | LPAR1 | DKK1 | SLC27A1 |
| 384 | SAP30L | TAF4B | FLT3 | ZBTB16 | IRAK2 |
| 385 | LAMB2 | ALDH1A3 | F2R | KCNQ4 | NCK2 |
| 386 | CST3 | MARCO | AKAP9 | COL7A1 | PLK4 |
| 387 | IL2RA | STAR | HEYL | EGR2 | TWIST1 |
| 388 | UGT2B7 | KISS1R | SLC9A3R2 | DUSP1 | MRPS23 |
| 389 | L1CAM | GSTA3 | VGLL1 | TUBA4A | KIF18A |
| 390 | E2F5 | IL27RA | RPS6KA2 | NR4A1 | NLK |
| 391 | DNMT3A | NFIA | NME1 | MSC | PCNA |
| 392 | PTPA | PDE5A | PTH1R | ELP2 | KLK3 |
| 393 | TUBA1C | KCND3 | GGT5 | IGF1R | LIN9 |
| 394 | CDS1 | GNG3 | COL17A1 | RAC2 | PROCR |
| 395 | CCT4 | GBP1 | GSTM5 | CSF2RA | CD3D |
| 396 | PHF12 | CCL11 | SOCS2 | NDN | IL6R |
| 397 | FZD4 | CDC14A | HSPA2 | PDE4B | AP1M2 |
| 398 | MUC1 | PLCE1 | OPRL1 | ETV4 | ADCY3 |
| 399 | ETV5 | LAMC2 | PYCARD | EPHA4 | BUB1B |
| 400 | EIF3B | FGFR1 | PDGFRB | CSF2RB | NOTCH1 |
| 401 | RBMS1 | EGR1 | TNNT3 | PLA2G2A | POLR2K |
| 402 | MAP4K4 | GPC6 | CXCL12 | CDC20 | JAG1 |
| 403 | THRB | FZD8 | DAPK1 | HP | BUB1 |
| 404 | FASN | TUBB2B | HIF1A | SERPINI1 | TNC |
| 405 | ARHGAP35 | LEF1 | CDH5 | FGF7 | LEFTY2 |
| 406 | VRK1 | EDNRB | GPER1 | VAV3 | KCNG1 |
| 407 | PRKCD | KRT14 | FOSB | SFTPA2 | ZFP36L1 |
| 408 | E2F4 | P2RY10 | GSTM4 | KLK3 | C3 |
| 409 | ARID2 | DEFB1 | CYP2A6 | PRKX | LPIN1 |
| 410 | CHRM1 | NTF3 | NCOA7 | CBR3 | FZD10 |
| 411 | PPP3CB | CYP2C8 | CDKN2C | FOSB | DSN1 |
| 412 | KRT5 | FOXM1 | FABP5 | GRPR | AURKA |
| 413 | BAZ2A | TNFSF10 | TEAD4 | TUBB2B | AP2B1 |
| 414 | ARPC5 | FOS | HIST1H2BJ | MYL2 | HSPA1B |
| 415 | SNAI1 | FGA | TP63 | FGF1 | BRIP1 |
| 416 | SNRPA | KCNQ5 | ADCY5 | KCNA5 | MSN |
| 417 | NFATC4 | BCL2A1 | GZMB | S1PR4 | HBB |
| 418 | CTNNA1 | KIT | TAF4B | CTF1 | TNFSF11 |
| 419 | GBP1 | SFRP1 | SYT1 | TGFB2 | CIITA |
| 420 | ETV4 | ITGA2 | EGR1 | CD79A | LMNB1 |
| 421 | CSNK1A1 | ADRA2A | PBX1 | EDN2 | LYN |
| 422 | POMC | CSF3R | LAMA3 | LEP | CTGF |
| 423 | DEFB1 | BMP7 | UGT2B7 | MMP3 | CD8A |
| 424 | PML | EPHB1 | ARRB1 | UGCG | GSTP1 |
| 425 | SOX9 | CD19 | NR2F2 | LIN28A | QKI |
| 426 | ABCA3 | AGT | SPARC | PDE3A | LRP1 |
| 427 | SPCS1 | CD2 | SREBF1 | MGP | IRAK3 |
| 428 | GATA2 | IGF2 | CAV1 | WNT3 | CENPU |
| 429 | MRPL11 | CTGF | ADH1B | BMP5 | ALDH3A1 |
| 430 | RXRA | INSL3 | NR4A2 | FOXJ1 | FEN1 |
| 431 | UGT2B11 | ALDH3A1 | EBF1 | JAK3 | CNTFR |
| 432 | NCAPG2 | CRHR1 | CXCL5 | GSTA3 | AVPR1A |
| 433 | GFRA1 | PTGFR | TGFB1I1 | EFNB3 | KCNQ1 |
| 434 | PRKCA | TNNT1 | LMO4 | PFKP | LCK |
| 435 | MLH1 | L1CAM | FGF1 | COL9A2 | EYA2 |
| 436 | POLD4 | FAT1 | GSTO2 | CCL28 | IGFBP1 |
| 437 | FFAR2 | GPR132 | WNT4 | CNR1 | CCR2 |
| 438 | FEN1 | LPL | GPC1 | AR | CNR1 |
| 439 | PPARD | NGFR | JUNB | GAL | PLCB1 |
| 440 | SSTR1 | CHRD | SIX5 | HBB | NPEPPS |
| 441 | ELP2 | EPHA7 | BATF | MYB | TNFRSF21 |
| 442 | SUPT5H | MMRN1 | LRP1 | KLF4 | RAC2 |
| 443 | CRHR1 | CDH2 | KITLG | BIRC3 | MCM4 |
| 444 | ZNF263 | HIST1H4A | CCL20 | MSX2 | MRPL58 |
| 445 | PYY | HOXB8 | DCN | KITLG | KCNA5 |
| 446 | PSMD7 | NR4A1 | BMPR1B | GAMT | CENPE |
| 447 | B3GNT3 | HEY2 | COL8A2 | IFI6 | CITED1 |
| 448 | SRF | PTGER2 | IVL | HTR3A | MDFIC |
| 449 | ARRB1 | COL9A2 | IL4I1 | SOCS1 | CD69 |
| 450 | PNPT1 | GHRH | CXCL11 | POU2AF1 | HIST2H2BE |
| 451 | TUBA4A | GNAO1 | CITED2 | FASN | KDR |
| 452 | CDCA7L | PTGS2 | SOD2 | KCNB1 | ANPEP |
| 453 | PSIP1 | KCNB1 | DUSP5 | COL4A6 | SOD2 |
| 454 | FAT1 | SCT | EDNRB | HSD11B1 | TCF7L1 |
| 455 | NME3 | IL2RG | ADM2 | CD69 | MYC |
| 456 | UTP14C | PLA2G4A | RET | XAGE2 | CCNA1 |
| 457 | CX3CL1 | HIST1H2BL | ADM | COL8A2 | GADD45B |
| 458 | NDUFS4 | FN1 | LEF1 | CRABP2 | HBA2 |
| 459 | TAF9 | ASCL1 | LYN | SIX3 | GNA15 |
| 460 | NME5 | CXCR3 | GRM4 | LBP | ALOX15B |
| 461 | IKBKB | COL14A1 | CYGB | LAMA2 | TRAF1 |
| 462 | IL4I1 | TNNI1 | FZD4 | GATA2 | OSMR |
| 463 | SAP30 | LEP | NPY2R | SOX2 | CD79A |
| 464 | ERBB2 | CFD | CAMK2B | RBP1 | INHBB |
| 465 | GZMB | GSTT2B | CXCL8 | UCP1 | ADIPOQ |
| 466 | NEDD4 | SERPINI1 | CHD7 | PROK1 | ESPL1 |
| 467 | SETD7 | AR | BCL11A | FGFR3 | PRLR |
| 468 | BMP4 | GATA5 | GALNT3 | IRF4 | CKS1B |
| 469 | CCT6A | EGFR | WNT6 | GAD1 | BIRC5 |
| 470 | RARB | ZBP1 | PLOD2 | IL1B | CLTC |
| 471 | MRTO4 | CGA | EDNRA | PRKCB | SMC4 |
| 472 | MYBBP1A | AK5 | KCND2 | SELE | CBX4 |
| 473 | FGF10 | WT1 | CDKN2A | PCK1 | PIM1 |
| 474 | PTEN | NR2F1 | F13A1 | IL12RB2 | PRKAR1B |
| 475 | TNNI2 | VIPR2 | DLL1 | KRT1 | FZD4 |
| 476 | CHRD | BTC | NKX3-1 | HBA2 | SPI1 |
| 477 | TRH | CTSG | HBA2 | DGAT2 | KCNAB2 |
| 478 | ADM | FOSB | NGFR | COL22A1 | POLE2 |
| 479 | BRIX1 | SAA1 | CBFA2T3 | SSTR1 | ARHGAP4 |
| 480 | GNAI1 | SOX2 | ADIPOQ | CD8B | MAOA |
| 481 | TF | THPO | TRH | KISS1R | GZMA |
| 482 | TCEA3 | TNFRSF18 | SNAI1 | KCNQ5 | HIST1H3J |
| 483 | GALC |  | CTLA4 | DCN | PIP5K1B |
| 484 | HMGN3 |  | GATA2 | IL7R | RPS19 |
| 485 | SMAD5 |  | KLK3 | TNNC1 | EREG |
| 486 | CCNA1 |  | NR2F1 | ENTPD8 | ACACB |
| 487 | PSME1 |  | TNNC1 | MYH2 | MAD2L1 |
| 488 | GTF3C1 |  | FPR3 | SFRP2 | JUNB |
| 489 | DCAF13 |  | OXGR1 | CCR2 | LTF |
| 490 | FGD1 |  | CALCRL | CD19 | RPL18A |
| 491 | KCND3 |  | FZD10 | ZAP70 | SERPINE1 |
| 492 | KPNA2 |  | EPHB3 | CCL20 | ABCA3 |
| 493 | RHOH |  | FABP4 | BMPR1B | LAMA3 |
| 494 | CHAF1A |  | TGFBR3 | ADH1B | MSX1 |
| 495 | ANXA1 |  | CCKBR | SHC2 | EFNA5 |
| 496 | MED13L |  | TAT | HIST1H3J | FGB |
| 497 | CPT1A |  | WNT5A | BCAN | SFN |
| 498 | UQCRQ |  | HSD17B1 | CD247 | HJURP |
| 499 | GPI |  | COL1A1 | WNT5A | COL18A1 |
| 500 | PLCD4 |  | CCR1 | NTS | DKK1 |
| 501 | CCT5 |  | NKX2-5 | LCK | NCAPG2 |
| 502 | BRD8 |  | PCK1 | ALOX15 | CDCA8 |
| 503 | CENPM |  | XAGE2 | S100A7 | HIST1H2BD |
| 504 | AQP9 |  | COL1A2 | SOCS2 | MAP3K8 |
| 505 | TAC1 |  | RPL39L | CA9 | IL10RA |
| 506 | ZNF350 |  | HERC5 | CD40LG | NET1 |
| 507 | PLK4 |  | MAOB | MARCO | PLK1 |
| 508 | POLR3D |  | NCAN | L1CAM | TK1 |
| 509 | MAPK3 |  | EPHA3 | CITED1 | SKA1 |
| 510 | RHOBTB2 |  | GSTA4 | NKX6-1 | CENPO |
| 511 | TCP1 |  | HIST1H3J | IGF2 | RAD51 |
| 512 | CDK1 |  | STAT1 | MUC2 | CHRM1 |
| 513 | RGS2 |  | TLE1 | FABP4 | P2RY6 |
| 514 | ACTL6A |  | B3GNT3 | NCAN | FOXM1 |
| 515 | HDAC11 |  | KCNA5 | NKX2-5 | MYB |
| 516 | RPS27L |  | COL6A3 | ANPEP | FGG |
| 517 | TNF |  | F5 | HIST1H4H | CCR7 |
| 518 | SUV39H1 |  | COL3A1 | FOXA3 | IRF4 |
| 519 | NUP205 |  | BATF2 | ZFP42 | TCEA3 |
| 520 | CXCL8 |  | KLF4 | DUSP4 | VAV1 |
| 521 | MMP1 |  | PLCB4 | SERPINA1 | SLC9A3R1 |
| 522 | UBE3A |  | P2RY13 | IGFBP1 | NT5E |
| 523 | ZFP42 |  | MMP2 | PAX7 | NME1 |
| 524 | SRSF5 |  | GNG7 | TGFBR3 | RRM2 |
| 525 | ITM2B |  | CXCL1 | ISG15 | HELLS |
| 526 | HERC5 |  | FOXP3 | CAMP | TAT |
| 527 | ENY2 |  | AFP | HIST1H2BL | COL4A2 |
| 528 | PDGFD |  | ENTPD3 | EREG | CDC6 |
| 529 | AFP |  | COL10A1 | CD3D | CD36 |
| 530 | ACTN4 |  | KISS1R | GSTM5 | CD40LG |
| 531 | PFDN5 |  | ZFP42 | UGT2B4 | CLSPN |
| 532 | NDUFS5 |  | CHRM4 | NKX3-1 | S1PR5 |
| 533 | TUBB4A |  | PDE5A | MAOB | EXOC2 |
| 534 | PHF20 |  | NPR1 | SCGB1A1 | E2F1 |
| 535 | HDAC7 |  | CDCA7L | GRP | RHOV |
| 536 | HSPB1 |  | SIX3 | PAX5 | CENPA |
| 537 | SGO2 |  | HSPG2 | MAOA | FAT1 |
| 538 | CEP290 |  | KLF5 | CD3E | GDF5 |
| 539 | SHMT2 |  | MMP10 | PLIN1 | IL2RG |
| 540 | PNPLA2 |  | FHL2 | CXCL8 | HEY2 |
| 541 | KLF11 |  | NFIB | AQP9 | GPC3 |
| 542 | YWHAQ |  | TIMP3 | ADCY1 | UGT2B7 |
| 543 | ARID4A |  | FABP6 | EGR3 | BMI1 |
| 544 | MYC |  | CKM | ADIPOQ | GPX7 |
| 545 | DUSP2 |  | PDIA2 | IVL | BTK |
| 546 | CXCL10 |  | TFAP2C | WDR38 | NDC80 |
| 547 | PDPK1 |  | DHCR24 | KCNF1 | CD8B |
| 548 | RPS27A |  | IKZF3 | CNTFR | PLEKHG5 |
| 549 | GHRH |  | CGN | PYY | CCNA2 |
| 550 | XIAP |  | L1CAM | BMP7 | C1S |
| 551 | LAMC2 |  | KCNF1 | OXGR1 | BRCA1 |
| 552 | NDUFA2 |  | SERPINA1 | EYA1 | SMC2 |
| 553 | TLE1 |  | CDK6 |  | CDCA5 |
| 554 | CCNB1 |  | FOSL1 |  | HLA-DPA1 |
| 555 | S1PR3 |  | DUSP2 |  | CRHR1 |
| 556 | DUSP16 |  | HTR3A |  | UBE2C |
| 557 | SERPINH1 |  | RUNX3 |  | CCP110 |
| 558 | TOLLIP |  | CTSK |  | RPS28 |
| 559 | RBM5 |  | CSF3R |  | PLIN1 |
| 560 | NEK2 |  | COL8A1 |  | CEBPB |
| 561 | TUBGCP3 |  | CHRM1 |  | WWP1 |
| 562 | ARHGEF3 |  | MMP9 |  | ADM |
| 563 | GNG4 |  | GPC4 |  | BARD1 |
| 564 | ICAM1 |  | FGFR3 |  | HDAC11 |
| 565 | PTMA |  | PMAIP1 |  | F2RL2 |
| 566 | IQCB1 |  | ASS1 |  | CBX8 |
| 567 | NEDD4L |  | UGT2B11 |  | SPHK1 |
| 568 | LCOR |  | GUCY1A2 |  | POMC |
| 569 | PABPC1 |  | HOXB4 |  | CD3G |
| 570 | BNIP3L |  | CTF1 |  | NRP2 |
| 571 | RET |  | ITGA2 |  | HSD11B1 |
| 572 | MCF2L |  | LUM |  | MSC |
| 573 | MXD4 |  | NPY5R |  | VIPR1 |
| 574 | NPEPPS |  | SERPINC1 |  | APLNR |
| 575 | ZEB1 |  | PRKAR2B |  | RFC4 |
| 576 | FDPS |  | ALDOC |  | DHFR |
| 577 | NDRG2 |  | SULT1E1 |  | CGN |
| 578 | WDR31 |  | ADORA2B |  | KLF4 |
| 579 | GATA6 |  | GNG4 |  | ARHGAP39 |
| 580 | ADRA2A |  | LYZ |  | GZMB |
| 581 | F2R |  | HOXB13 |  | DBF4 |
| 582 | FANCG |  | BCAN |  | EZH2 |
| 583 | UPF2 |  | HIST1H2BL |  | COL9A3 |
| 584 | ASS1 |  | GAD1 |  | TCF4 |
| 585 | SLC2A1 |  | GHR |  | CCNB2 |
| 586 | CD82 |  | MYH2 |  | BIRC3 |
| 587 | KCNC2 |  | HIST4H4 |  | LBP |
| 588 | FKBP3 |  | FZD6 |  | KIF2C |
| 589 | TCF7L2 |  | CYR61 |  | AHR |
| 590 | PDGFRB |  | FGF2 |  | SPC24 |
| 591 | TLR1 |  | INSL3 |  | CYP19A1 |
| 592 | CHD1 |  | MGP |  | LCP2 |
| 593 | RASA1 |  | APOA1 |  | ADORA2B |
| 594 | EIF3E |  | P2RY2 |  | CXCR6 |
| 595 | RIPK3 |  | ALDH1A1 |  | TLE2 |
| 596 | MYO6 |  | COL7A1 |  | ITGA6 |
| 597 | MRGBP |  | CXCL9 |  | CFD |
| 598 | AP1M2 |  | COL5A1 |  | PDE4B |
| 599 | ARHGEF2 |  | SSTR2 |  | MCM8 |
| 600 | GRM4 |  | AOX1 |  | DCN |
| 601 | SMAD3 |  | DGAT2 |  | FGFR1 |
| 602 | MRPS12 |  | COL12A1 |  | COL11A1 |
| 603 | EIF4EBP1 |  | COL22A1 |  | ALOX15 |
| 604 | PDIA2 |  | COL5A2 |  | MMP2 |
| 605 | AGTR1 |  | GSTA1 |  | AFP |
| 606 | GAB1 |  | DKK1 |  | RBL1 |
| 607 | CKM |  | LEP |  | CBX6 |
| 608 | MRPL4 |  | IRAK3 |  | MMP3 |
| 609 | MAP2K3 |  | TUBA4A |  | BMP7 |
| 610 | DDC |  | SFTPD |  | KRT1 |
| 611 | KIF3C |  | CTGF |  | VCAM1 |
| 612 | GNB4 |  | GSTP1 |  | WNT10A |
| 613 | ZNF521 |  | SERPINI1 |  | CDC25A |
| 614 | DHCR7 |  | HLF |  | HIST1H4D |
| 615 | ARHGEF12 |  | CXCL13 |  | ADRB1 |
| 616 | PTGER3 |  | S100A7 |  | PTTG1 |
| 617 | NUP160 |  | EFNA5 |  | COL6A2 |
| 618 | P2RY2 |  | MYCN |  | COL10A1 |
| 619 | ESPL1 |  | OASL |  | E2F5 |
| 620 | HTR3A |  | CYP2C8 |  | CD4 |
| 621 | IGF2 |  | COL9A3 |  | HCK |
| 622 | MUC5B |  | MX1 |  | HK2 |
| 623 | ELF1 |  | ESRRG |  | C1QA |
| 624 | ANAPC2 |  | EDN3 |  | HLA-DRA |
| 625 | SHC2 |  | ZBP1 |  | PMAIP1 |
| 626 | ACAN |  | NTS |  | SNAI1 |
| 627 | COL4A5 |  | EFNB3 |  | SMO |
| 628 | SLC27A1 |  | GPC3 |  | HLA-DRB1 |
| 629 | CD2BP2 |  | FOXJ1 |  | CD2 |
| 630 | KISS1R |  | PLA2G4A |  | ZEB1 |
| 631 | CBX4 |  | CLU |  | TUBA4A |
| 632 | PCK2 |  | PENK |  | IRS2 |
| 633 | CYCS |  | EGFR |  | SELE |
| 634 | ASF1B |  | RELN |  | BLM |
| 635 | ZKSCAN1 |  | ACAN |  | SERPINC1 |
| 636 | GEMIN8 |  | PRKAA2 |  | MYL2 |
| 637 | LPAR6 |  | ACTN2 |  | GATA3 |
| 638 | WDR3 |  | PTHLH |  | ZNF217 |
| 639 | RHOC |  | FGFR4 |  | GSTM2 |
| 640 | H1F0 |  | RGMA |  | ELP2 |
| 641 | TSPO |  | HIST1H4A |  | AKT3 |
| 642 | GRP |  | GNAO1 |  | NPY |
| 643 | LMNB1 |  | CD36 |  | ADCY5 |
| 644 | BDKRB2 |  | WNT10A |  | CDC45 |
| 645 | ADCY3 |  | MMP7 |  | CD19 |
| 646 | PGR |  | APOA2 |  | HLA-DQA1 |
| 647 | TP53BP1 |  | CDH1 |  | KCNQ4 |
| 648 | RRM2 |  | POU2AF1 |  | CYP2J2 |
| 649 | BATF |  | KRT14 |  | AURKB |
| 650 | QKI |  | UGT2B4 |  | ITGB6 |
| 651 | LRP6 |  | KRT1 |  | GPR132 |
| 652 | TGS1 |  | TNF |  | ALDOC |
| 653 | DNMT1 |  | GGT1 |  | MGP |
| 654 | DAPP1 |  | MUC5B |  | CAMK2D |
| 655 | CTSG |  | EGF |  | ITGA2 |
| 656 | GNA15 |  | LBP |  | TNNT1 |
| 657 | CYP2A6 |  | CNR1 |  | LEP |
| 658 | H2AFZ |  | ALB |  | KDM4B |
| 659 | BMI1 |  | HIST1H4B |  | PTGER2 |
| 660 | P2RY6 |  | IRF4 |  | NPAS2 |
| 661 | NSF |  | ADRB1 |  | TRAT1 |
| 662 | RFC3 |  | LTF |  | PTHLH |
| 663 | HTT |  | NPW |  | STAR |
| 664 | H2AFX |  | PAX7 |  | PTAFR |
| 665 | GSTO2 |  | TNNT1 |  | HLA-DQB1 |
| 666 | VAMP2 |  | TNNI3 |  | RAB3IP |
| 667 | NAB1 |  | NTF3 |  | CHEK1 |
| 668 | SDC4 |  | CCL21 |  | APOE |
| 669 | PLCB4 |  | AGT |  | SELL |
| 670 | DOCK1 |  | ALOX15B |  | FST |
| 671 | MIS18A |  | ADRA2C |  | FMOD |
| 672 | ADM2 |  | LEFTY2 |  | ESR1 |
| 673 | SNAPC2 |  | PROK1 |  | HSPG2 |
| 674 | SMARCC2 |  | WT1 |  | FPR1 |
| 675 | PRKAR1A |  | PAX5 |  | CRABP2 |
| 676 | F2RL2 |  | EPO |  | SYCP2 |
| 677 | PDCD4 |  | DEFB1 |  | COL6A1 |
| 678 | APC |  | ALDH3B2 |  | GALNT3 |
| 679 | FMOD |  | LPAR3 |  | CXCR3 |
| 680 | PTGFR |  | SAA1 |  | TRIP6 |
| 681 | EDNRA |  | GSTM1 |  | OXGR1 |
| 682 | COL14A1 |  | MYBPC1 |  | CENPM |
| 683 | APP |  | CGA |  | NR0B1 |
| 684 | MED16 |  | FGG |  | TYMS |
| 685 | SFTPA2 |  | LIN28A |  | TGFB3 |
| 686 | KLF2 |  | HP |  | HSPA1A |
| 687 | GSTA2 |  | PEG10 |  | TNNI3 |
| 688 | EFNB1 |  | CCL19 |  | HIST1H4I |
| 689 | MYCT1 |  | UGT2B17 |  |  |
| 690 | EZH1 |  |  |  |  |
| 691 | CDC25C |  |  |  |  |
| 692 | GHR |  |  |  |  |
| 693 | GSTM5 |  |  |  |  |
| 694 | CITED1 |  |  |  |  |
| 695 | ALDH3B2 |  |  |  |  |
| 696 | ADCY5 |  |  |  |  |
| 697 | ITGA6 |  |  |  |  |
| 698 | PPP2R5A |  |  |  |  |
| 699 | BRD3 |  |  |  |  |
| 700 | DOK1 |  |  |  |  |
| 701 | RPS19 |  |  |  |  |
| 702 | FOXJ1 |  |  |  |  |
| 703 | KCNS3 |  |  |  |  |
| 704 | SOCS1 |  |  |  |  |
| 705 | NFKBIB |  |  |  |  |
| 706 | LPAR2 |  |  |  |  |
| 707 | GUCY1A2 |  |  |  |  |
| 708 | PIK3CD |  |  |  |  |
| 709 | ADRA2C |  |  |  |  |
| 710 | TEK |  |  |  |  |
| 711 | HIST1H2BJ |  |  |  |  |
| 712 | CBFA2T3 |  |  |  |  |
| 713 | HMGCR |  |  |  |  |
| 714 | TEAD2 |  |  |  |  |
| 715 | COL4A6 |  |  |  |  |
| 716 | LDB2 |  |  |  |  |
| 717 | PCM1 |  |  |  |  |
| 718 | COL10A1 |  |  |  |  |
| 719 | SFRP2 |  |  |  |  |
| 720 | ZNF217 |  |  |  |  |
| 721 | DCN |  |  |  |  |
| 722 | EPHA3 |  |  |  |  |
| 723 | CAMK2B |  |  |  |  |
| 724 | EXOC2 |  |  |  |  |
| 725 | APOA2 |  |  |  |  |
| 726 | ACKR3 |  |  |  |  |
| 727 | CASP7 |  |  |  |  |
| 728 | NLK |  |  |  |  |
| 729 | TCF3 |  |  |  |  |
| 730 | KIT |  |  |  |  |
| 731 | PSMA7 |  |  |  |  |
| 732 | CLASP2 |  |  |  |  |
| 733 | MDM2 |  |  |  |  |
| 734 | ADCY1 |  |  |  |  |
| 735 | HELLS |  |  |  |  |
| 736 | SPARC |  |  |  |  |
| 737 | COL6A3 |  |  |  |  |
| 738 | RBP1 |  |  |  |  |
| 739 | H2AFY2 |  |  |  |  |
| 740 | WDHD1 |  |  |  |  |
| 741 | RGS10 |  |  |  |  |
| 742 | TK1 |  |  |  |  |
| 743 | CDKN2D |  |  |  |  |
| 744 | NFKB2 |  |  |  |  |
| 745 | GALNT3 |  |  |  |  |
| 746 | ALDH1A3 |  |  |  |  |
| 747 | LAMA2 |  |  |  |  |
| 748 | CBX7 |  |  |  |  |
| 749 | CD79B |  |  |  |  |
| 750 | JAK3 |  |  |  |  |
| 751 | RPS6KA3 |  |  |  |  |
| 752 | PCNA |  |  |  |  |
| 753 | BDP1 |  |  |  |  |
| 754 | SAA1 |  |  |  |  |
| 755 | CYBA |  |  |  |  |
| 756 | HSF1 |  |  |  |  |
| 757 | VEGFA |  |  |  |  |
| 758 | GSC |  |  |  |  |
| 759 | CXCL11 |  |  |  |  |
| 760 | WNT5B |  |  |  |  |
| 761 | MMRN1 |  |  |  |  |
| 762 | LPAR3 |  |  |  |  |
| 763 | TLR2 |  |  |  |  |
| 764 | INSL3 |  |  |  |  |
| 765 | SUN2 |  |  |  |  |
| 766 | ADAM17 |  |  |  |  |
| 767 | HIST4H4 |  |  |  |  |
| 768 | SYT1 |  |  |  |  |
| 769 | POU2AF1 |  |  |  |  |
| 770 | EDN1 |  |  |  |  |
| 771 | FANCD2 |  |  |  |  |
| 772 | HEATR1 |  |  |  |  |
| 773 | TKT |  |  |  |  |
| 774 | PRKDC |  |  |  |  |
| 775 | BLNK |  |  |  |  |
| 776 | AKAP9 |  |  |  |  |
| 777 | NR2F1 |  |  |  |  |
| 778 | CHD7 |  |  |  |  |
| 779 | DKK1 |  |  |  |  |
| 780 | PARD3 |  |  |  |  |
| 781 | RPS20 |  |  |  |  |
| 782 | HSPA2 |  |  |  |  |
| 783 | GSR |  |  |  |  |
| 784 | IKBKE |  |  |  |  |
| 785 | IVL |  |  |  |  |
| 786 | ME1 |  |  |  |  |
| 787 | TIMP3 |  |  |  |  |
| 788 | SERPINI1 |  |  |  |  |
| 789 | TRIB3 |  |  |  |  |
| 790 | CDK19 |  |  |  |  |
| 791 | CGA |  |  |  |  |
| 792 | TAT |  |  |  |  |
| 793 | ALDH3A2 |  |  |  |  |
| 794 | MRPL12 |  |  |  |  |
| 795 | COL1A2 |  |  |  |  |
| 796 | NF1 |  |  |  |  |
| 797 | VWF |  |  |  |  |
| 798 | FLT3 |  |  |  |  |
| 799 | ALDOA |  |  |  |  |
| 800 | CDC6 |  |  |  |  |
| 801 | ACACB |  |  |  |  |
| 802 | FZD6 |  |  |  |  |
| 803 | GSTA4 |  |  |  |  |
| 804 | CBR3 |  |  |  |  |
| 805 | FANCI |  |  |  |  |
| 806 | STAG3 |  |  |  |  |
| 807 | MX2 |  |  |  |  |
| 808 | KRT14 |  |  |  |  |
| 809 | GJA1 |  |  |  |  |
| 810 | LPAR1 |  |  |  |  |
| 811 | FYN |  |  |  |  |
| 812 | RPL22L1 |  |  |  |  |
| 813 | CTLA4 |  |  |  |  |
| 814 | COL3A1 |  |  |  |  |
| 815 | PLOD2 |  |  |  |  |
| 816 | GPX4 |  |  |  |  |
| 817 | NDN |  |  |  |  |
| 818 | COL1A1 |  |  |  |  |
| 819 | LMNA |  |  |  |  |
| 820 | HEYL |  |  |  |  |
| 821 | DUSP10 |  |  |  |  |
| 822 | BRIP1 |  |  |  |  |
| 823 | NDUFB9 |  |  |  |  |
| 824 | ANGPTL4 |  |  |  |  |
| 825 | TNFAIP3 |  |  |  |  |
| 826 | LAMB3 |  |  |  |  |
| 827 | MYH11 |  |  |  |  |
| 828 | KMT2D |  |  |  |  |
| 829 | CHRM4 |  |  |  |  |
| 830 | EGLN2 |  |  |  |  |
| 831 | KCND2 |  |  |  |  |
| 832 | LCK |  |  |  |  |
| 833 | CLTC |  |  |  |  |
| 834 | CITED2 |  |  |  |  |
| 835 | STUB1 |  |  |  |  |
| 836 | THRA |  |  |  |  |
| 837 | UCP2 |  |  |  |  |
| 838 | LUM |  |  |  |  |
| 839 | CXCL12 |  |  |  |  |
| 840 | COL12A1 |  |  |  |  |
| 841 | GPC4 |  |  |  |  |
| 842 | GSTA3 |  |  |  |  |
| 843 | GSTM4 |  |  |  |  |
| 844 | TUBB2B |  |  |  |  |
| 845 | SMC4 |  |  |  |  |
| 846 | TOP2A |  |  |  |  |
| 847 | ARHGEF16 |  |  |  |  |
| 848 | F13A1 |  |  |  |  |
| 849 | NOTCH4 |  |  |  |  |
| 850 | HIST1H2BD |  |  |  |  |
| 851 | DUSP1 |  |  |  |  |
| 852 | TFAP2C |  |  |  |  |
| 853 | LEF1 |  |  |  |  |
| 854 | BIN1 |  |  |  |  |
| 855 | HHEX |  |  |  |  |
| 856 | CTSK |  |  |  |  |
| 857 | CFD |  |  |  |  |
| 858 | GPX2 |  |  |  |  |
| 859 | RPS16 |  |  |  |  |
| 860 | CDON |  |  |  |  |
| 861 | RRM2B |  |  |  |  |
| 862 | NR4A2 |  |  |  |  |
| 863 | TCF4 |  |  |  |  |
| 864 | RUNX1 |  |  |  |  |
| 865 | IL23A |  |  |  |  |
| 866 | THBS1 |  |  |  |  |
| 867 | PDGFB |  |  |  |  |
| 868 | UGT2B4 |  |  |  |  |
| 869 | VIM |  |  |  |  |
| 870 | AGL |  |  |  |  |
| 871 | CDH5 |  |  |  |  |
| 872 | TUBG2 |  |  |  |  |
| 873 | WLS |  |  |  |  |
| 874 | EPHX1 |  |  |  |  |
| 875 | HLA-A |  |  |  |  |
| 876 | RACGAP1 |  |  |  |  |
| 877 | CD14 |  |  |  |  |
| 878 | COL5A2 |  |  |  |  |
| 879 | RBL2 |  |  |  |  |
| 880 | FES |  |  |  |  |
| 881 | TNFSF10 |  |  |  |  |
| 882 | CCP110 |  |  |  |  |
| 883 | CXCL2 |  |  |  |  |
| 884 | SOX4 |  |  |  |  |
| 885 | EGR3 |  |  |  |  |
| 886 | CALCRL |  |  |  |  |
| 887 | MAOA |  |  |  |  |
| 888 | PTPN1 |  |  |  |  |
| 889 | NR2F2 |  |  |  |  |
| 890 | BMP6 |  |  |  |  |
| 891 | BAG1 |  |  |  |  |
| 892 | HOXB4 |  |  |  |  |
| 893 | BCAT1 |  |  |  |  |
| 894 | NPY |  |  |  |  |
| 895 | GSTM2 |  |  |  |  |
| 896 | SCD |  |  |  |  |
| 897 | PLIN1 |  |  |  |  |
| 898 | WNT11 |  |  |  |  |
| 899 | LTBP1 |  |  |  |  |
| 900 | EGF |  |  |  |  |
| 901 | FKBP4 |  |  |  |  |
| 902 | MAFF |  |  |  |  |
| 903 | IRF4 |  |  |  |  |
| 904 | HIST2H2BE |  |  |  |  |
| 905 | LOR |  |  |  |  |
| 906 | LIF |  |  |  |  |
| 907 | TRIP6 |  |  |  |  |
| 908 | ITGB4 |  |  |  |  |
| 909 | SFN |  |  |  |  |
| 910 | MAP2K4 |  |  |  |  |
| 911 | ITGA9 |  |  |  |  |
| 912 | PSMB9 |  |  |  |  |
| 913 | FDXR |  |  |  |  |
| 914 | WNT7B |  |  |  |  |
| 915 | TRAF1 |  |  |  |  |
| 916 | BBC3 |  |  |  |  |
| 917 | TCF7 |  |  |  |  |
| 918 | CCR1 |  |  |  |  |
| 919 | CXCR4 |  |  |  |  |
| 920 | DTL |  |  |  |  |
| 921 | GPR68 |  |  |  |  |
| 922 | LRP1 |  |  |  |  |
| 923 | FCER1A |  |  |  |  |
| 924 | ERN1 |  |  |  |  |
| 925 | COL8A2 |  |  |  |  |
| 926 | VIPR2 |  |  |  |  |
| 927 | EBF1 |  |  |  |  |
| 928 | SOCS2 |  |  |  |  |
| 929 | MYCN |  |  |  |  |
| 930 | EPHA7 |  |  |  |  |
| 931 | DLC1 |  |  |  |  |
| 932 | S1PR5 |  |  |  |  |
| 933 | CD8B |  |  |  |  |
| 934 | PARD6A |  |  |  |  |
| 935 | CD40 |  |  |  |  |
| 936 | IL6 |  |  |  |  |
| 937 | IFI35 |  |  |  |  |
| 938 | MMP9 |  |  |  |  |
| 939 | CCL2 |  |  |  |  |
| 940 | KCNQ5 |  |  |  |  |
| 941 | SSTR2 |  |  |  |  |
| 942 | SULT1E1 |  |  |  |  |
| 943 | ALDOC |  |  |  |  |
| 944 | FGFR3 |  |  |  |  |
| 945 | ALOX15B |  |  |  |  |
| 946 | VCAM1 |  |  |  |  |
| 947 | AK5 |  |  |  |  |
| 948 | BTC |  |  |  |  |
| 949 | PTH1R |  |  |  |  |
| 950 | COL5A1 |  |  |  |  |
| 951 | IL2RG |  |  |  |  |
| 952 | RGS4 |  |  |  |  |
| 953 | ACTN2 |  |  |  |  |
| 954 | CSF3R |  |  |  |  |
| 955 | SLC25A4 |  |  |  |  |
| 956 | FAS |  |  |  |  |
| 957 | UTRN |  |  |  |  |
| 958 | ASCL1 |  |  |  |  |
| 959 | PDE4B |  |  |  |  |
| 960 | PIK3R1 |  |  |  |  |
| 961 | ENTPD3 |  |  |  |  |
| 962 | F5 |  |  |  |  |
| 963 | HLA-B |  |  |  |  |
| 964 | FOXP3 |  |  |  |  |
| 965 | KCNF1 |  |  |  |  |
| 966 | CXCL13 |  |  |  |  |
| 967 | ISG20 |  |  |  |  |
| 968 | RAB3IP |  |  |  |  |
| 969 | ZWINT |  |  |  |  |
| 970 | SATB1 |  |  |  |  |
| 971 | N4BP2 |  |  |  |  |
| 972 | LRRK2 |  |  |  |  |
| 973 | NPAS2 |  |  |  |  |
| 974 | IL1B |  |  |  |  |
| 975 | CASP1 |  |  |  |  |
| 976 | GNG12 |  |  |  |  |
| 977 | PYCARD |  |  |  |  |
| 978 | BIRC3 |  |  |  |  |
| 979 | TBX2 |  |  |  |  |
| 980 | MMP10 |  |  |  |  |
| 981 | DUSP6 |  |  |  |  |
| 982 | PAX5 |  |  |  |  |
| 983 | FOS |  |  |  |  |
| 984 | CSF2RA |  |  |  |  |
| 985 | BMPR1B |  |  |  |  |
| 986 | MITF |  |  |  |  |
| 987 | INHBB |  |  |  |  |
| 988 | ITGA2 |  |  |  |  |
| 989 | GPC3 |  |  |  |  |
| 990 | PDE5A |  |  |  |  |
| 991 | SERPINC1 |  |  |  |  |
| 992 | TGFBI |  |  |  |  |
| 993 | ETS1 |  |  |  |  |
| 994 | OSM |  |  |  |  |
| 995 | S1PR1 |  |  |  |  |
| 996 | HIF1A |  |  |  |  |
| 997 | NR1D1 |  |  |  |  |
| 998 | NGEF |  |  |  |  |
| 999 | ARHGDIB |  |  |  |  |
| 1000 | ZBP1 |  |  |  |  |
| 1001 | BATF2 |  |  |  |  |
| 1002 | GGT5 |  |  |  |  |
| 1003 | APBB1 |  |  |  |  |
| 1004 | TFRC |  |  |  |  |
| 1005 | PDE6B |  |  |  |  |
| 1006 | EDNRB |  |  |  |  |
| 1007 | ALOX15 |  |  |  |  |
| 1008 | FGF1 |  |  |  |  |
| 1009 | CRABP2 |  |  |  |  |
| 1010 | HSPA1A |  |  |  |  |
| 1011 | LIFR |  |  |  |  |
| 1012 | ADIPOQ |  |  |  |  |
| 1013 | HDAC9 |  |  |  |  |
| 1014 | MSX1 |  |  |  |  |
| 1015 | LRP2 |  |  |  |  |
| 1016 | S100A7 |  |  |  |  |
| 1017 | CTSS |  |  |  |  |
| 1018 | COL8A1 |  |  |  |  |
| 1019 | UCP1 |  |  |  |  |
| 1020 | GPER1 |  |  |  |  |
| 1021 | LAMA3 |  |  |  |  |
| 1022 | ALDH1A1 |  |  |  |  |
| 1023 | AOX1 |  |  |  |  |
| 1024 | IKZF3 |  |  |  |  |
| 1025 | MMP2 |  |  |  |  |
| 1026 | CD79A |  |  |  |  |
| 1027 | KLK3 |  |  |  |  |
| 1028 | ALDH3A1 |  |  |  |  |
| 1029 | HSD17B1 |  |  |  |  |
| 1030 | HSPA6 |  |  |  |  |
| 1031 | ESRRG |  |  |  |  |
| 1032 | SPRY2 |  |  |  |  |
| 1033 | ALB |  |  |  |  |
| 1034 | STAR |  |  |  |  |
| 1035 | NKX3-1 |  |  |  |  |
| 1036 | GNG3 |  |  |  |  |
| 1037 | ADH1B |  |  |  |  |
| 1038 | VCAN |  |  |  |  |
| 1039 | TRPC6 |  |  |  |  |
| 1040 | HIST1H4L |  |  |  |  |
| 1041 | CLU |  |  |  |  |
| 1042 | FGB |  |  |  |  |
| 1043 | IL18 |  |  |  |  |
| 1044 | MUC2 |  |  |  |  |
| 1045 | LEFTY2 |  |  |  |  |
| 1046 | KCNA5 |  |  |  |  |
| 1047 | FGFR1 |  |  |  |  |
| 1048 | WNT9A |  |  |  |  |
| 1049 | KLF4 |  |  |  |  |
| 1050 | EYA2 |  |  |  |  |
| 1051 | PLCE1 |  |  |  |  |
| 1052 | HIST1H4C |  |  |  |  |
| 1053 | HBB |  |  |  |  |
| 1054 | DOK2 |  |  |  |  |
| 1055 | CSF2RB |  |  |  |  |
| 1056 | PRKAR2B |  |  |  |  |
| 1057 | PCK1 |  |  |  |  |
| 1058 | CGN |  |  |  |  |
| 1059 | CD36 |  |  |  |  |
| 1060 | ADORA1 |  |  |  |  |
| 1061 | SDC2 |  |  |  |  |
| 1062 | APLNR |  |  |  |  |
| 1063 | IRF7 |  |  |  |  |
| 1064 | WNT5A |  |  |  |  |
| 1065 | CXCR6 |  |  |  |  |
| 1066 | HBA2 |  |  |  |  |
| 1067 | SPP1 |  |  |  |  |
| 1068 | CD247 |  |  |  |  |
| 1069 | PEG10 |  |  |  |  |
| 1070 | WNT4 |  |  |  |  |
| 1071 | FST |  |  |  |  |
| 1072 | UGT2B17 |  |  |  |  |
| 1073 | FOSB |  |  |  |  |
| 1074 | KCNH2 |  |  |  |  |
| 1075 | HIST1H4B |  |  |  |  |
| 1076 | TNNT1 |  |  |  |  |
| 1077 | SMARCA1 |  |  |  |  |
| 1078 | CD19 |  |  |  |  |
| 1079 | NCAM1 |  |  |  |  |
| 1080 | C4BPA |  |  |  |  |
| 1081 | MYH2 |  |  |  |  |
| 1082 | GPR18 |  |  |  |  |
| 1083 | FABP4 |  |  |  |  |
| 1084 | GSTT2B |  |  |  |  |
| 1085 | HOXB13 |  |  |  |  |
| 1086 | PRKAA2 |  |  |  |  |
| 1087 | MX1 |  |  |  |  |
| 1088 | APOA1 |  |  |  |  |
| 1089 | EGLN3 |  |  |  |  |
| 1090 | C1S |  |  |  |  |
| 1091 | CXCL9 |  |  |  |  |
| 1092 | SYCP2 |  |  |  |  |
| 1093 | PTHLH |  |  |  |  |
| 1094 | CYP2C8 |  |  |  |  |
| 1095 | OASL |  |  |  |  |
| 1096 | OXTR |  |  |  |  |
| 1097 | TLR7 |  |  |  |  |
| 1098 | NPW |  |  |  |  |
| 1099 | GATA4 |  |  |  |  |
| 1100 | SIX1 |  |  |  |  |
| 1101 | ZAP70 |  |  |  |  |
| 1102 | TCAP |  |  |  |  |
| 1103 | CDC14A |  |  |  |  |
| 1104 | HIST1H3J |  |  |  |  |
| 1105 | SERPINA1 |  |  |  |  |
| 1106 | LYZ |  |  |  |  |
| 1107 | S1PR4 |  |  |  |  |
| 1108 | TNNI3 |  |  |  |  |
| 1109 | KCNB1 |  |  |  |  |
| 1110 | EDN3 |  |  |  |  |
| 1111 | NPY1R |  |  |  |  |
| 1112 | HIST1H4E |  |  |  |  |
| 1113 | GRB7 |  |  |  |  |
| 1114 | BMP5 |  |  |  |  |
| 1115 | FGF2 |  |  |  |  |
| 1116 | IL7R |  |  |  |  |
| 1117 | LBP |  |  |  |  |
| 1118 | GSTM1 |  |  |  |  |
| 1119 | CD3D |  |  |  |  |
| 1120 | ADCYAP1 |  |  |  |  |
| 1121 | MYBPC1 |  |  |  |  |
| 1122 | FGG |  |  |  |  |
| 1123 | TNNI1 |  |  |  |  |
| 1124 | HIST1H4A |  |  |  |  |
| 1125 | WDR38 |  |  |  |  |
| 1126 | HSD11B1 |  |  |  |  |
| 1127 | COL2A1 |  |  |  |  |
| 1128 | NR0B1 |  |  |  |  |
| 1129 | COL9A2 |  |  |  |  |
| 1130 | SCT |  |  |  |  |
| 1131 | COL17A1 |  |  |  |  |
| 1132 | NPY2R |  |  |  |  |
| 1133 | EREG |  |  |  |  |
| 1134 | TNNT3 |  |  |  |  |
| 1135 | TP63 |  |  |  |  |
| 1136 | LIN28A |  |  |  |  |
| 1137 | CD2 |  |  |  |  |
| 1138 | EDN2 |  |  |  |  |
| 1139 | DES |  |  |  |  |
| 1140 | AMH |  |  |  |  |
| 1141 | TGFBR3 |  |  |  |  |
| 1142 | FABP6 |  |  |  |  |
| 1143 | BMP7 |  |  |  |  |
| 1144 | PENK |  |  |  |  |
| 1145 | CNTFR |  |  |  |  |
| 1146 | HOXB8 |  |  |  |  |
| 1147 | HIST1H2BL |  |  |  |  |
| 1148 | COL11A1 |  |  |  |  |
| 1149 | PAX7 |  |  |  |  |
| 1150 | PIK3C2G |  |  |  |  |
| 1151 | HIST1H4H |  |  |  |  |
| 1152 | CAMP |  |  |  |  |
| 1153 | NTS |  |  |  |  |
| 1154 | FGA |  |  |  |  |
| 1155 | MAOB |  |  |  |  |
| 1156 | SOX2 |  |  |  |  |
| 1157 | WT1 |  |  |  |  |
