# Supplementary material for: RNA-Seq-Based Breast Cancer Subtypes Classification Using Machine Learning Approaches
Source: Comput Intell Neurosci. 2020 Oct 29;2020:4737969. doi: 10.1155/2020/4737969 (PMC7644310; doi:10.1155/2020/4737969)
Supplement: Supplementary Materials — Figure S1: heatmap for Her2 and non Her2 groups. The left group 1 represents the Her2 group and the right group 2 denotes the non-Her2 group. Figure S2: heatmap for LumA and non-LumA groups. The left group 1 represents the LumA group and the right group 2 denotes the non-LumA group. Figure S3: heatmap for LumB and non-LumB groups. The left group 1 represents the LumB group and the right group 2 denotes the non-LumB group. Figure S4: heatmap for Normal-like and non-Normal-like groups. The left group 1 represents the Normal-like group and the right group 2 denotes the non-Normal-like group. S1 File: the detailed information of weighted DEGs for classification. S2 File: the detailed information of weighted DEGs for GO enrichment analysis. S3 File: the detailed enriched GO terms results for Basal-like subtype. S4 File: the detailed enriched GO terms results for Her2 subtype. S5 File: the detailed enriched GO terms results for LumA subtype. S6 File: the detailed enriched GO terms results for LumB subtype. S7 File: the detailed enriched GO terms results for Normal-like subtype. [file 4737969.f1.zip › supplementary materials/S3 File.docx]

**S3 File:** The detailed enriched GO terms results for Basal-like subtype.

**Control group:**

| **No.** | **ID** | **Description** | **GeneRatio** | **BgRatio** | **pvalue** | **p.adjust** | **count** |
| --- | --- | --- | --- | --- | --- | --- | --- |
| 1 | GO:0002009 | morphogenesis of an epithelium | 462/5151 | 36542/668746 | 0 | 0 | 462 |
| 2 | GO:0032496 | response to lipopolysaccharide | 340/2775 | 36542/668746 | 0 | 0 | 340 |
| 3 | GO:0002237 | response to molecule of bacterial origin | 349/2850 | 36542/668746 | 0 | 0 | 349 |
| 4 | GO:0045785 | positive regulation of cell adhesion | 363/3160 | 36542/668746 | 0 | 0 | 363 |
| 5 | GO:0022407 | regulation of cell-cell adhesion | 285/3003 | 36542/668746 | 0 | 0 | 285 |
| 6 | GO:0042063 | gliogenesis | 263/1830 | 36542/668746 | 0 | 0 | 263 |
| 7 | GO:0018108 | peptidyl-tyrosine phosphorylation | 269/2701 | 36542/668746 | 0 | 0 | 269 |
| 8 | GO:0018212 | peptidyl-tyrosine modification | 269/2701 | 36542/668746 | 0 | 0 | 269 |
| 9 | GO:0016055 | Wnt signaling pathway | 299/3240 | 36542/668746 | 0 | 0 | 299 |
| 10 | GO:0198738 | cell-cell signaling by wnt | 299/3240 | 36542/668746 | 0 | 0 | 299 |
| 11 | GO:0022612 | gland morphogenesis | 95/666 | 36542/668746 | 0 | 0 | 95 |
| 12 | GO:0022409 | positive regulation of cell-cell adhesion | 180/1485 | 36542/668746 | 0 | 0 | 180 |
| 13 | GO:0050673 | epithelial cell proliferation | 307/2701 | 36542/668746 | 0 | 0 | 307 |
| 14 | GO:0050678 | regulation of epithelial cell proliferation | 218/2016 | 36542/668746 | 0 | 0 | 218 |
| 15 | GO:0043491 | protein kinase B signaling | 158/1275 | 36542/668746 | 0 | 0 | 158 |
| 16 | GO:0001667 | ameboidal-type cell migration | 244/2556 | 36542/668746 | 0 | 0 | 244 |
| 17 | GO:0009612 | response to mechanical stimulus | 126/946 | 36542/668746 | 0 | 0 | 126 |
| 18 | GO:1903037 | regulation of leukocyte cell-cell adhesion | 171/1431 | 36542/668746 | 0 | 0 | 171 |
| 19 | GO:0051896 | regulation of protein kinase B signaling | 141/1035 | 36542/668746 | 0 | 0 | 141 |
| 20 | GO:0007159 | leukocyte cell-cell adhesion | 186/1540 | 36542/668746 | 0 | 0 | 186 |
| 21 | GO:1903039 | positive regulation of leukocyte cell-cell adhesion | 128/861 | 36542/668746 | 0 | 0 | 128 |
| 22 | GO:1901342 | regulation of vasculature development | 264/2016 | 36542/668746 | 0 | 0 | 264 |
| 23 | GO:0010631 | epithelial cell migration | 173/1540 | 36542/668746 | 0 | 0 | 173 |
| 24 | GO:0090132 | epithelium migration | 173/1540 | 36542/668746 | 0 | 0 | 173 |
| 25 | GO:0090130 | tissue migration | 173/1540 | 36542/668746 | 0 | 0 | 173 |
| 26 | GO:0050870 | positive regulation of T cell activation | 108/741 | 36542/668746 | 0 | 0 | 108 |
| 27 | GO:0050679 | positive regulation of epithelial cell proliferation | 106/630 | 36542/668746 | 0 | 0 | 106 |
| 28 | GO:0045765 | regulation of angiogenesis | 220/1378 | 36542/668746 | 0 | 0 | 220 |
| 29 | GO:0045933 | positive regulation of muscle contraction | 31/91 | 36542/668746 | 0 | 0 | 31 |
| 30 | GO:0043588 | skin development | 138/1081 | 36542/668746 | 0 | 0 | 138 |
| 31 | GO:0003012 | muscle system process | 249/2628 | 36542/668746 | 1.11E-16 | 6.04E-15 | 249 |
| 32 | GO:0034612 | response to tumor necrosis factor | 104/780 | 36542/668746 | 1.11E-16 | 6.04E-15 | 104 |
| 33 | GO:0048771 | tissue remodeling | 63/351 | 36542/668746 | 1.11E-16 | 6.04E-15 | 63 |
| 34 | GO:0001763 | morphogenesis of a branching structure | 157/1431 | 36542/668746 | 2.22E-16 | 1.11E-14 | 157 |
| 35 | GO:0051897 | positive regulation of protein kinase B signaling | 103/780 | 36542/668746 | 2.22E-16 | 1.11E-14 | 103 |
| 36 | GO:0007517 | muscle organ development | 212/2145 | 36542/668746 | 2.22E-16 | 1.11E-14 | 212 |
| 37 | GO:0051251 | positive regulation of lymphocyte activation | 133/1128 | 36542/668746 | 2.22E-16 | 1.11E-14 | 133 |
| 38 | GO:0061138 | morphogenesis of a branching epithelium | 144/1275 | 36542/668746 | 4.44E-16 | 2.08E-14 | 144 |
| 39 | GO:0048708 | astrocyte differentiation | 39/153 | 36542/668746 | 4.44E-16 | 2.08E-14 | 39 |
| 40 | GO:0046488 | phosphatidylinositol metabolic process | 89/630 | 36542/668746 | 4.44E-16 | 2.08E-14 | 89 |
| 41 | GO:0070372 | regulation of ERK1 and ERK2 cascade | 234/2485 | 36542/668746 | 1.44E-15 | 6.62E-14 | 234 |
| 42 | GO:0002040 | sprouting angiogenesis | 47/231 | 36542/668746 | 5.77E-15 | 2.6E-13 | 47 |
| 43 | GO:0046849 | bone remodeling | 33/120 | 36542/668746 | 6.88E-15 | 3.04E-13 | 33 |
| 44 | GO:0046486 | glycerolipid metabolic process | 139/1275 | 36542/668746 | 2.12E-14 | 9.19E-13 | 139 |
| 45 | GO:0031589 | cell-substrate adhesion | 168/1653 | 36542/668746 | 2.42E-14 | 9.94E-13 | 168 |
| 46 | GO:0070371 | ERK1 and ERK2 cascade | 249/2775 | 36542/668746 | 3.94E-14 | 1.56E-12 | 249 |
| 47 | GO:0055123 | digestive system development | 96/780 | 36542/668746 | 2.06E-13 | 7.89E-12 | 96 |
| 48 | GO:0071356 | cellular response to tumor necrosis factor | 86/666 | 36542/668746 | 2.57E-13 | 9.68E-12 | 86 |
| 49 | GO:0046834 | lipid phosphorylation | 65/435 | 36542/668746 | 2.92E-13 | 1.07E-11 | 65 |
| 50 | GO:0048732 | gland development | 490/6441 | 36542/668746 | 3.3E-13 | 1.19E-11 | 490 |
| 51 | GO:0030111 | regulation of Wnt signaling pathway | 139/1326 | 36542/668746 | 4.02E-13 | 1.43E-11 | 139 |
| 52 | GO:0007162 | negative regulation of cell adhesion | 142/1378 | 36542/668746 | 8.12E-13 | 2.83E-11 | 142 |
| 53 | GO:0050769 | positive regulation of neurogenesis | 285/3403 | 36542/668746 | 1.79E-12 | 6.06E-11 | 285 |
| 54 | GO:0007059 | chromosome segregation | 148/1485 | 36542/668746 | 3.37E-12 | 1.08E-10 | 148 |
| 55 | GO:0048871 | multicellular organismal homeostasis | 306/3741 | 36542/668746 | 4.19E-12 | 1.31E-10 | 306 |
| 56 | GO:0090257 | regulation of muscle system process | 113/1035 | 36542/668746 | 4.72E-12 | 1.43E-10 | 113 |
| 57 | GO:0010632 | regulation of epithelial cell migration | 124/1176 | 36542/668746 | 4.75E-12 | 1.43E-10 | 124 |
| 58 | GO:0008544 | epidermis development | 151/1540 | 36542/668746 | 6.93E-12 | 2.05E-10 | 151 |
| 59 | GO:0002064 | epithelial cell development | 127/1225 | 36542/668746 | 8.26E-12 | 2.36E-10 | 127 |
| 60 | GO:0010517 | regulation of phospholipase activity | 33/153 | 36542/668746 | 1.1E-11 | 3.07E-10 | 33 |
| 61 | GO:0009314 | response to radiation | 203/2278 | 36542/668746 | 1.45E-11 | 4E-10 | 203 |
| 62 | GO:0007611 | learning or memory | 56/378 | 36542/668746 | 1.7E-11 | 4.62E-10 | 56 |
| 63 | GO:0071496 | cellular response to external stimulus | 149/1540 | 36542/668746 | 2.48E-11 | 6.59E-10 | 149 |
| 64 | GO:0045987 | positive regulation of smooth muscle contraction | 21/66 | 36542/668746 | 2.47E-11 | 6.59E-10 | 21 |
| 65 | GO:0006939 | smooth muscle contraction | 53/351 | 36542/668746 | 2.8E-11 | 7.37E-10 | 53 |
| 66 | GO:0042326 | negative regulation of phosphorylation | 246/2926 | 36542/668746 | 3.78E-11 | 9.84E-10 | 246 |
| 67 | GO:0006979 | response to oxidative stress | 251/3003 | 36542/668746 | 4.25E-11 | 1.09E-09 | 251 |
| 68 | GO:0034330 | cell junction organization | 117/1128 | 36542/668746 | 5.09E-11 | 1.28E-09 | 117 |
| 69 | GO:0016202 | regulation of striated muscle tissue development | 60/435 | 36542/668746 | 6.55E-11 | 1.6E-09 | 60 |
| 70 | GO:1901861 | regulation of muscle tissue development | 60/435 | 36542/668746 | 6.55E-11 | 1.6E-09 | 60 |
| 71 | GO:0048634 | regulation of muscle organ development | 60/435 | 36542/668746 | 6.55E-11 | 1.6E-09 | 60 |
| 72 | GO:1904894 | positive regulation of STAT cascade | 38/210 | 36542/668746 | 8.46E-11 | 2.04E-09 | 38 |
| 73 | GO:0007409 | axonogenesis | 139/1431 | 36542/668746 | 8.55E-11 | 2.04E-09 | 139 |
| 74 | GO:0006936 | muscle contraction | 151/1596 | 36542/668746 | 9.08E-11 | 2.15E-09 | 151 |
| 75 | GO:0097696 | STAT cascade | 65/496 | 36542/668746 | 1E-10 | 2.34E-09 | 65 |
| 76 | GO:0061564 | axon development | 172/1891 | 36542/668746 | 1.04E-10 | 2.42E-09 | 172 |
| 77 | GO:0048565 | digestive tract development | 82/703 | 36542/668746 | 1.55E-10 | 3.56E-09 | 82 |
| 78 | GO:0035265 | organ growth | 88/780 | 36542/668746 | 1.9E-10 | 4.31E-09 | 88 |
| 79 | GO:0002042 | cell migration involved in sprouting angiogenesis | 20/66 | 36542/668746 | 1.97E-10 | 4.43E-09 | 20 |
| 80 | GO:1903524 | positive regulation of blood circulation | 29/136 | 36542/668746 | 2.52E-10 | 5.62E-09 | 29 |
| 81 | GO:0046854 | phosphatidylinositol phosphorylation | 56/406 | 36542/668746 | 2.75E-10 | 6.01E-09 | 56 |
| 82 | GO:0045619 | regulation of lymphocyte differentiation | 61/465 | 36542/668746 | 3.51E-10 | 7.54E-09 | 61 |
| 83 | GO:0044843 | cell cycle G1/S phase transition | 174/1953 | 36542/668746 | 3.89E-10 | 8.28E-09 | 174 |
| 84 | GO:0048754 | branching morphogenesis of an epithelial tube | 100/946 | 36542/668746 | 4.35E-10 | 9.17E-09 | 100 |
| 85 | GO:0000082 | G1/S transition of mitotic cell cycle | 169/1891 | 36542/668746 | 5.47E-10 | 1.14E-08 | 169 |
| 86 | GO:0010810 | regulation of cell-substrate adhesion | 93/861 | 36542/668746 | 5.77E-10 | 1.19E-08 | 93 |
| 87 | GO:0070302 | regulation of stress-activated protein kinase signaling cascade | 63/496 | 36542/668746 | 6.81E-10 | 1.4E-08 | 63 |
| 88 | GO:0043409 | negative regulation of MAPK cascade | 83/741 | 36542/668746 | 8.51E-10 | 1.73E-08 | 83 |
| 89 | GO:0070482 | response to oxygen levels | 271/3403 | 36542/668746 | 8.73E-10 | 1.76E-08 | 271 |
| 90 | GO:1903708 | positive regulation of hemopoiesis | 71/595 | 36542/668746 | 9.39E-10 | 1.88E-08 | 71 |
| 91 | GO:0007568 | aging | 190/2211 | 36542/668746 | 1.09E-09 | 2.14E-08 | 190 |
| 92 | GO:0060191 | regulation of lipase activity | 38/231 | 36542/668746 | 1.43E-09 | 2.77E-08 | 38 |
| 93 | GO:0030049 | muscle filament sliding | 19/66 | 36542/668746 | 1.46E-09 | 2.78E-08 | 19 |
| 94 | GO:0033275 | actin-myosin filament sliding | 19/66 | 36542/668746 | 1.46E-09 | 2.78E-08 | 19 |
| 95 | GO:0000302 | response to reactive oxygen species | 119/1225 | 36542/668746 | 1.79E-09 | 3.37E-08 | 119 |
| 96 | GO:0099177 | regulation of trans-synaptic signaling | 115/1176 | 36542/668746 | 2.27E-09 | 4.25E-08 | 115 |
| 97 | GO:0070555 | response to interleukin-1 | 67/561 | 36542/668746 | 2.62E-09 | 4.87E-08 | 67 |
| 98 | GO:0048863 | stem cell differentiation | 94/903 | 36542/668746 | 3.03E-09 | 5.5E-08 | 94 |
| 99 | GO:0045444 | fat cell differentiation | 94/903 | 36542/668746 | 3.03E-09 | 5.5E-08 | 94 |
| 100 | GO:0050804 | modulation of chemical synaptic transmission | 111/1128 | 36542/668746 | 3.01E-09 | 5.5E-08 | 111 |
| 101 | GO:0017145 | stem cell division | 17/55 | 36542/668746 | 3.16E-09 | 5.69E-08 | 17 |
| 102 | GO:0001894 | tissue homeostasis | 64/528 | 36542/668746 | 3.27E-09 | 5.84E-08 | 64 |
| 103 | GO:0055024 | regulation of cardiac muscle tissue development | 33/190 | 36542/668746 | 4.12E-09 | 7.3E-08 | 33 |
| 104 | GO:0034599 | cellular response to oxidative stress | 140/1540 | 36542/668746 | 5.33E-09 | 9.31E-08 | 140 |
| 105 | GO:1901988 | negative regulation of cell cycle phase transition | 110/1128 | 36542/668746 | 5.75E-09 | 9.97E-08 | 110 |
| 106 | GO:0071621 | granulocyte chemotaxis | 48/351 | 36542/668746 | 6.44E-09 | 1.11E-07 | 48 |
| 107 | GO:1903829 | positive regulation of cellular protein localization | 160/1830 | 36542/668746 | 6.72E-09 | 1.15E-07 | 160 |
| 108 | GO:0042303 | molting cycle | 41/276 | 36542/668746 | 7.26E-09 | 1.22E-07 | 41 |
| 109 | GO:0042633 | hair cycle | 41/276 | 36542/668746 | 7.26E-09 | 1.22E-07 | 41 |
| 110 | GO:0002262 | myeloid cell homeostasis | 63/528 | 36542/668746 | 7.89E-09 | 1.32E-07 | 63 |
| 111 | GO:0098813 | nuclear chromosome segregation | 106/1081 | 36542/668746 | 8.02E-09 | 1.33E-07 | 106 |
| 112 | GO:1901987 | regulation of cell cycle phase transition | 239/3003 | 36542/668746 | 8.51E-09 | 1.4E-07 | 239 |
| 113 | GO:0001818 | negative regulation of cytokine production | 89/861 | 36542/668746 | 1.08E-08 | 1.75E-07 | 89 |
| 114 | GO:0045621 | positive regulation of lymphocyte differentiation | 30/171 | 36542/668746 | 1.6E-08 | 2.59E-07 | 30 |
| 115 | GO:0046677 | response to antibiotic | 193/2346 | 36542/668746 | 2.1E-08 | 3.35E-07 | 193 |
| 116 | GO:0060402 | calcium ion transport into cytosol | 40/276 | 36542/668746 | 2.21E-08 | 3.48E-07 | 40 |
| 117 | GO:0060401 | cytosolic calcium ion transport | 40/276 | 36542/668746 | 2.21E-08 | 3.48E-07 | 40 |
| 118 | GO:0048545 | response to steroid hormone | 331/4465 | 36542/668746 | 2.53E-08 | 3.95E-07 | 331 |
| 119 | GO:1902107 | positive regulation of leukocyte differentiation | 49/378 | 36542/668746 | 2.57E-08 | 3.98E-07 | 49 |
| 120 | GO:0046850 | regulation of bone remodeling | 13/36 | 36542/668746 | 2.71E-08 | 4.14E-07 | 13 |
| 121 | GO:0002695 | negative regulation of leukocyte activation | 42/300 | 36542/668746 | 2.76E-08 | 4.2E-07 | 42 |
| 122 | GO:0031670 | cellular response to nutrient | 19/78 | 36542/668746 | 3.01E-08 | 4.55E-07 | 19 |
| 123 | GO:0002285 | lymphocyte activation involved in immune response | 44/325 | 36542/668746 | 3.58E-08 | 5.38E-07 | 44 |
| 124 | GO:0032872 | regulation of stress-activated MAPK cascade | 56/465 | 36542/668746 | 3.71E-08 | 5.53E-07 | 56 |
| 125 | GO:0050890 | cognition | 61/528 | 36542/668746 | 4.36E-08 | 6.41E-07 | 61 |
| 126 | GO:0033598 | mammary gland epithelial cell proliferation | 10/21 | 36542/668746 | 4.78E-08 | 6.9E-07 | 10 |
| 127 | GO:0030856 | regulation of epithelial cell differentiation | 69/630 | 36542/668746 | 5E-08 | 7.18E-07 | 69 |
| 128 | GO:0051051 | negative regulation of transport | 173/2080 | 36542/668746 | 5.27E-08 | 7.52E-07 | 173 |
| 129 | GO:0006940 | regulation of smooth muscle contraction | 31/190 | 36542/668746 | 5.33E-08 | 7.56E-07 | 31 |
| 130 | GO:0031345 | negative regulation of cell projection organization | 37/253 | 36542/668746 | 5.73E-08 | 8.03E-07 | 37 |
| 131 | GO:0032612 | interleukin-1 production | 29/171 | 36542/668746 | 5.94E-08 | 8.23E-07 | 29 |
| 132 | GO:0045446 | endothelial cell differentiation | 29/171 | 36542/668746 | 5.94E-08 | 8.23E-07 | 29 |
| 133 | GO:0045453 | bone resorption | 14/45 | 36542/668746 | 7E-08 | 9.63E-07 | 14 |
| 134 | GO:0015908 | fatty acid transport | 20/91 | 36542/668746 | 8.41E-08 | 1.14E-06 | 20 |
| 135 | GO:0000280 | nuclear division | 259/3403 | 36542/668746 | 9.56E-08 | 1.29E-06 | 259 |
| 136 | GO:0014013 | regulation of gliogenesis | 43/325 | 36542/668746 | 9.82E-08 | 1.31E-06 | 43 |
| 137 | GO:1901991 | negative regulation of mitotic cell cycle phase transition | 95/990 | 36542/668746 | 1.23E-07 | 1.64E-06 | 95 |
| 138 | GO:0010634 | positive regulation of epithelial cell migration | 79/780 | 36542/668746 | 1.58E-07 | 2.09E-06 | 79 |
| 139 | GO:0021782 | glial cell development | 32/210 | 36542/668746 | 1.67E-07 | 2.19E-06 | 32 |
| 140 | GO:0044706 | multi-multicellular organism process | 101/1081 | 36542/668746 | 1.8E-07 | 2.33E-06 | 101 |
| 141 | GO:0010812 | negative regulation of cell-substrate adhesion | 28/171 | 36542/668746 | 2.12E-07 | 2.69E-06 | 28 |
| 142 | GO:0042542 | response to hydrogen peroxide | 59/528 | 36542/668746 | 2.24E-07 | 2.83E-06 | 59 |
| 143 | GO:0045580 | regulation of T cell differentiation | 49/406 | 36542/668746 | 2.35E-07 | 2.96E-06 | 49 |
| 144 | GO:0031016 | pancreas development | 26/153 | 36542/668746 | 2.68E-07 | 3.33E-06 | 26 |
| 145 | GO:0032652 | regulation of interleukin-1 production | 26/153 | 36542/668746 | 2.68E-07 | 3.33E-06 | 26 |
| 146 | GO:0045931 | positive regulation of mitotic cell cycle | 97/1035 | 36542/668746 | 2.7E-07 | 3.34E-06 | 97 |
| 147 | GO:0030258 | lipid modification | 84/861 | 36542/668746 | 3.19E-07 | 3.93E-06 | 84 |
| 148 | GO:0001649 | osteoblast differentiation | 100/1081 | 36542/668746 | 3.26E-07 | 3.99E-06 | 100 |
| 149 | GO:0007219 | Notch signaling pathway | 51/435 | 36542/668746 | 3.33E-07 | 4.06E-06 | 51 |
| 150 | GO:0042098 | T cell proliferation | 61/561 | 36542/668746 | 3.63E-07 | 4.4E-06 | 61 |
| 151 | GO:0070374 | positive regulation of ERK1 and ERK2 cascade | 124/1431 | 36542/668746 | 4.75E-07 | 5.73E-06 | 124 |
| 152 | GO:0010717 | regulation of epithelial to mesenchymal transition | 35/253 | 36542/668746 | 4.99E-07 | 5.96E-06 | 35 |
| 153 | GO:0003158 | endothelium development | 35/253 | 36542/668746 | 4.99E-07 | 5.96E-06 | 35 |
| 154 | GO:0048145 | regulation of fibroblast proliferation | 37/276 | 36542/668746 | 5.28E-07 | 6.24E-06 | 37 |
| 155 | GO:0097553 | calcium ion transmembrane import into cytosol | 31/210 | 36542/668746 | 5.26E-07 | 6.24E-06 | 31 |
| 156 | GO:1904892 | regulation of STAT cascade | 48/406 | 36542/668746 | 5.66E-07 | 6.66E-06 | 48 |
| 157 | GO:0043407 | negative regulation of MAP kinase activity | 39/300 | 36542/668746 | 5.87E-07 | 6.87E-06 | 39 |
| 158 | GO:0006937 | regulation of muscle contraction | 63/595 | 36542/668746 | 5.93E-07 | 6.9E-06 | 63 |
| 159 | GO:0031668 | cellular response to extracellular stimulus | 80/820 | 36542/668746 | 5.96E-07 | 6.9E-06 | 80 |
| 160 | GO:0001655 | urogenital system development | 233/3081 | 36542/668746 | 6.35E-07 | 7.32E-06 | 233 |
| 161 | GO:0033673 | negative regulation of kinase activity | 116/1326 | 36542/668746 | 6.82E-07 | 7.82E-06 | 116 |
| 162 | GO:0072511 | divalent inorganic cation transport | 92/990 | 36542/668746 | 7.66E-07 | 8.74E-06 | 92 |
| 163 | GO:2000027 | regulation of animal organ morphogenesis | 95/1035 | 36542/668746 | 8.77E-07 | 9.96E-06 | 95 |
| 164 | GO:0032102 | negative regulation of response to external stimulus | 98/1081 | 36542/668746 | 1.03E-06 | 1.16E-05 | 98 |
| 165 | GO:0070588 | calcium ion transmembrane transport | 45/378 | 36542/668746 | 1.03E-06 | 1.16E-05 | 45 |
| 166 | GO:1903531 | negative regulation of secretion by cell | 57/528 | 36542/668746 | 1.07E-06 | 1.2E-05 | 57 |
| 167 | GO:0031098 | stress-activated protein kinase signaling cascade | 79/820 | 36542/668746 | 1.13E-06 | 1.26E-05 | 79 |
| 168 | GO:0032355 | response to estradiol | 76/780 | 36542/668746 | 1.17E-06 | 1.29E-05 | 76 |
| 169 | GO:0051924 | regulation of calcium ion transport | 76/780 | 36542/668746 | 1.17E-06 | 1.29E-05 | 76 |
| 170 | GO:0001666 | response to hypoxia | 221/2926 | 36542/668746 | 1.31E-06 | 1.44E-05 | 221 |
| 171 | GO:0001952 | regulation of cell-matrix adhesion | 36/276 | 36542/668746 | 1.43E-06 | 1.57E-05 | 36 |
| 172 | GO:0019233 | sensory perception of pain | 32/231 | 36542/668746 | 1.45E-06 | 1.58E-05 | 32 |
| 173 | GO:0036293 | response to decreased oxygen levels | 225/3003 | 36542/668746 | 1.84E-06 | 1.99E-05 | 225 |
| 174 | GO:0046456 | icosanoid biosynthetic process | 11/36 | 36542/668746 | 2.17E-06 | 2.32E-05 | 11 |
| 175 | GO:0034103 | regulation of tissue remodeling | 15/66 | 36542/668746 | 2.15E-06 | 2.32E-05 | 15 |
| 176 | GO:0032956 | regulation of actin cytoskeleton organization | 75/780 | 36542/668746 | 2.21E-06 | 2.36E-05 | 75 |
| 177 | GO:0042102 | positive regulation of T cell proliferation | 26/171 | 36542/668746 | 2.38E-06 | 2.52E-05 | 26 |
| 178 | GO:0051983 | regulation of chromosome segregation | 44/378 | 36542/668746 | 2.45E-06 | 2.58E-05 | 44 |
| 179 | GO:0001780 | neutrophil homeostasis | 5/6 | 36542/668746 | 2.79E-06 | 2.93E-05 | 5 |
| 180 | GO:0051348 | negative regulation of transferase activity | 124/1485 | 36542/668746 | 2.96E-06 | 3.09E-05 | 124 |
| 181 | GO:1903169 | regulation of calcium ion transmembrane transport | 35/276 | 36542/668746 | 3.78E-06 | 3.89E-05 | 35 |
| 182 | GO:0051250 | negative regulation of lymphocyte activation | 33/253 | 36542/668746 | 3.82E-06 | 3.93E-05 | 33 |
| 183 | GO:0048144 | fibroblast proliferation | 37/300 | 36542/668746 | 3.92E-06 | 3.98E-05 | 37 |
| 184 | GO:0035296 | regulation of tube diameter | 48/435 | 36542/668746 | 3.95E-06 | 3.98E-05 | 48 |
| 185 | GO:0097746 | regulation of blood vessel diameter | 48/435 | 36542/668746 | 3.95E-06 | 3.98E-05 | 48 |
| 186 | GO:0097756 | negative regulation of blood vessel diameter | 31/231 | 36542/668746 | 4.1E-06 | 4.11E-05 | 31 |
| 187 | GO:0035924 | cellular response to vascular endothelial growth factor stimulus | 16/78 | 36542/668746 | 4.2E-06 | 4.2E-05 | 16 |
| 188 | GO:0007160 | cell-matrix adhesion | 71/741 | 36542/668746 | 4.55E-06 | 4.53E-05 | 71 |
| 189 | GO:0001516 | prostaglandin biosynthetic process | 6/10 | 36542/668746 | 4.61E-06 | 4.56E-05 | 6 |
| 190 | GO:0046457 | prostanoid biosynthetic process | 6/10 | 36542/668746 | 4.61E-06 | 4.56E-05 | 6 |
| 191 | GO:0061180 | mammary gland epithelium development | 29/210 | 36542/668746 | 4.66E-06 | 4.59E-05 | 29 |
| 192 | GO:0050866 | negative regulation of cell activation | 43/378 | 36542/668746 | 5.67E-06 | 5.53E-05 | 43 |
| 193 | GO:1901652 | response to peptide | 331/4753 | 36542/668746 | 6.23E-06 | 6.05E-05 | 331 |
| 194 | GO:0002828 | regulation of type 2 immune response | 7/15 | 36542/668746 | 6.33E-06 | 6.12E-05 | 7 |
| 195 | GO:0060249 | anatomical structure homeostasis | 166/2145 | 36542/668746 | 6.68E-06 | 6.43E-05 | 166 |
| 196 | GO:0050851 | antigen receptor-mediated signaling pathway | 45/406 | 36542/668746 | 6.88E-06 | 6.6E-05 | 45 |
| 197 | GO:0051091 | positive regulation of DNA-binding transcription factor activity | 85/946 | 36542/668746 | 7.05E-06 | 6.74E-05 | 85 |
| 198 | GO:0015718 | monocarboxylic acid transport | 25/171 | 36542/668746 | 7.47E-06 | 7.11E-05 | 25 |
| 199 | GO:0048546 | digestive tract morphogenesis | 20/120 | 36542/668746 | 8.22E-06 | 7.76E-05 | 20 |
| 200 | GO:0002768 | immune response-regulating cell surface receptor signaling pathway | 94/1081 | 36542/668746 | 9.04E-06 | 8.46E-05 | 94 |
| 201 | GO:0051302 | regulation of cell division | 54/528 | 36542/668746 | 9.7E-06 | 9.05E-05 | 54 |
| 202 | GO:0019229 | regulation of vasoconstriction | 23/153 | 36542/668746 | 1.07E-05 | 9.9E-05 | 23 |
| 203 | GO:0030850 | prostate gland development | 30/231 | 36542/668746 | 1.12E-05 | 0.000103 | 30 |
| 204 | GO:0033135 | regulation of peptidyl-serine phosphorylation | 30/231 | 36542/668746 | 1.12E-05 | 0.000103 | 30 |
| 205 | GO:0045165 | cell fate commitment | 148/1891 | 36542/668746 | 1.15E-05 | 0.000105 | 148 |
| 206 | GO:0010948 | negative regulation of cell cycle process | 148/1891 | 36542/668746 | 1.15E-05 | 0.000105 | 148 |
| 207 | GO:0007411 | axon guidance | 64/666 | 36542/668746 | 1.18E-05 | 0.000107 | 64 |
| 208 | GO:0097485 | neuron projection guidance | 64/666 | 36542/668746 | 1.18E-05 | 0.000107 | 64 |
| 209 | GO:1904062 | regulation of cation transmembrane transport | 84/946 | 36542/668746 | 1.22E-05 | 0.000111 | 84 |
| 210 | GO:2000816 | negative regulation of mitotic sister chromatid separation | 18/105 | 36542/668746 | 1.53E-05 | 0.000137 | 18 |
| 211 | GO:1905819 | negative regulation of chromosome separation | 18/105 | 36542/668746 | 1.53E-05 | 0.000137 | 18 |
| 212 | GO:0048730 | epidermis morphogenesis | 10/36 | 36542/668746 | 1.62E-05 | 0.000144 | 10 |
| 213 | GO:0010522 | regulation of calcium ion transport into cytosol | 26/190 | 36542/668746 | 1.65E-05 | 0.000147 | 26 |
| 214 | GO:0061982 | meiosis I cell cycle process | 26/190 | 36542/668746 | 1.65E-05 | 0.000147 | 26 |
| 215 | GO:0007127 | meiosis I | 21/136 | 36542/668746 | 1.67E-05 | 0.000147 | 21 |
| 216 | GO:1905207 | regulation of cardiocyte differentiation | 15/78 | 36542/668746 | 1.88E-05 | 0.000165 | 15 |
| 217 | GO:0051090 | regulation of DNA-binding transcription factor activity | 167/2211 | 36542/668746 | 2.3E-05 | 0.000198 | 167 |
| 218 | GO:0001953 | negative regulation of cell-matrix adhesion | 11/45 | 36542/668746 | 2.32E-05 | 0.000198 | 11 |
| 219 | GO:0050710 | negative regulation of cytokine secretion | 11/45 | 36542/668746 | 2.32E-05 | 0.000198 | 11 |
| 220 | GO:0045995 | regulation of embryonic development | 37/325 | 36542/668746 | 2.38E-05 | 0.000201 | 37 |
| 221 | GO:0050671 | positive regulation of lymphocyte proliferation | 33/276 | 36542/668746 | 2.38E-05 | 0.000201 | 33 |
| 222 | GO:0032946 | positive regulation of mononuclear cell proliferation | 33/276 | 36542/668746 | 2.38E-05 | 0.000201 | 33 |
| 223 | GO:0051048 | negative regulation of secretion | 71/780 | 36542/668746 | 2.48E-05 | 0.000209 | 71 |
| 224 | GO:0010975 | regulation of neuron projection development | 142/1830 | 36542/668746 | 2.55E-05 | 0.000214 | 142 |
| 225 | GO:0007044 | cell-substrate junction assembly | 41/378 | 36542/668746 | 2.82E-05 | 0.000232 | 41 |
| 226 | GO:1903034 | regulation of response to wounding | 41/378 | 36542/668746 | 2.82E-05 | 0.000232 | 41 |
| 227 | GO:0051403 | stress-activated MAPK cascade | 68/741 | 36542/668746 | 2.81E-05 | 0.000232 | 68 |
| 228 | GO:0043547 | positive regulation of GTPase activity | 68/741 | 36542/668746 | 2.81E-05 | 0.000232 | 68 |
| 229 | GO:0045017 | glycerolipid biosynthetic process | 41/378 | 36542/668746 | 2.82E-05 | 0.000232 | 41 |
| 230 | GO:0001942 | hair follicle development | 29/231 | 36542/668746 | 2.93E-05 | 0.000237 | 29 |
| 231 | GO:0022404 | molting cycle process | 29/231 | 36542/668746 | 2.93E-05 | 0.000237 | 29 |
| 232 | GO:0022405 | hair cycle process | 29/231 | 36542/668746 | 2.93E-05 | 0.000237 | 29 |
| 233 | GO:0098773 | skin epidermis development | 29/231 | 36542/668746 | 2.93E-05 | 0.000237 | 29 |
| 234 | GO:0007584 | response to nutrient | 82/946 | 36542/668746 | 3.55E-05 | 0.000285 | 82 |
| 235 | GO:0045930 | negative regulation of mitotic cell cycle | 145/1891 | 36542/668746 | 3.73E-05 | 0.000298 | 145 |
| 236 | GO:0014706 | striated muscle tissue development | 183/2485 | 36542/668746 | 3.94E-05 | 0.000314 | 183 |
| 237 | GO:0007260 | tyrosine phosphorylation of STAT protein | 25/190 | 36542/668746 | 4.61E-05 | 0.000364 | 25 |
| 238 | GO:1990868 | response to chemokine | 25/190 | 36542/668746 | 4.61E-05 | 0.000364 | 25 |
| 239 | GO:1990869 | cellular response to chemokine | 25/190 | 36542/668746 | 4.61E-05 | 0.000364 | 25 |
| 240 | GO:0045927 | positive regulation of growth | 97/1176 | 36542/668746 | 5.08E-05 | 0.000399 | 97 |
| 241 | GO:0048285 | organelle fission | 266/3828 | 36542/668746 | 5.33E-05 | 0.000417 | 266 |
| 242 | GO:0045598 | regulation of fat cell differentiation | 36/325 | 36542/668746 | 5.4E-05 | 0.00042 | 36 |
| 243 | GO:0070665 | positive regulation of leukocyte proliferation | 36/325 | 36542/668746 | 5.4E-05 | 0.00042 | 36 |
| 244 | GO:0071347 | cellular response to interleukin-1 | 40/378 | 36542/668746 | 6.05E-05 | 0.000467 | 40 |
| 245 | GO:1902106 | negative regulation of leukocyte differentiation | 30/253 | 36542/668746 | 6.33E-05 | 0.000487 | 30 |
| 246 | GO:0071383 | cellular response to steroid hormone stimulus | 128/1653 | 36542/668746 | 6.66E-05 | 0.000509 | 128 |
| 247 | GO:0051321 | meiotic cell cycle | 90/1081 | 36542/668746 | 6.65E-05 | 0.000509 | 90 |
| 248 | GO:0051147 | regulation of muscle cell differentiation | 42/406 | 36542/668746 | 6.76E-05 | 0.000515 | 42 |
| 249 | GO:0055001 | muscle cell development | 44/435 | 36542/668746 | 7.79E-05 | 0.000592 | 44 |
| 250 | GO:0014821 | phasic smooth muscle contraction | 7/21 | 36542/668746 | 8.55E-05 | 0.000643 | 7 |
| 251 | GO:0002825 | regulation of T-helper 1 type immune response | 7/21 | 36542/668746 | 8.55E-05 | 0.000643 | 7 |
| 252 | GO:0042092 | type 2 immune response | 7/21 | 36542/668746 | 8.55E-05 | 0.000643 | 7 |
| 253 | GO:1905208 | negative regulation of cardiocyte differentiation | 6/15 | 36542/668746 | 8.66E-05 | 0.00065 | 6 |
| 254 | GO:0060562 | epithelial tube morphogenesis | 185/2556 | 36542/668746 | 8.92E-05 | 0.000667 | 185 |
| 255 | GO:0031069 | hair follicle morphogenesis | 8/28 | 36542/668746 | 9.18E-05 | 0.000682 | 8 |
| 256 | GO:0045124 | regulation of bone resorption | 8/28 | 36542/668746 | 9.18E-05 | 0.000682 | 8 |
| 257 | GO:1903320 | regulation of protein modification by small protein conjugation or removal | 46/465 | 36542/668746 | 9.24E-05 | 0.000684 | 46 |
| 258 | GO:0033046 | negative regulation of sister chromatid segregation | 18/120 | 36542/668746 | 9.51E-05 | 0.000694 | 18 |
| 259 | GO:0051985 | negative regulation of chromosome segregation | 18/120 | 36542/668746 | 9.51E-05 | 0.000694 | 18 |
| 260 | GO:0042093 | T-helper cell differentiation | 18/120 | 36542/668746 | 9.51E-05 | 0.000694 | 18 |
| 261 | GO:0002287 | alpha-beta T cell activation involved in immune response | 18/120 | 36542/668746 | 9.51E-05 | 0.000694 | 18 |
| 262 | GO:0002292 | T cell differentiation involved in immune response | 18/120 | 36542/668746 | 9.51E-05 | 0.000694 | 18 |
| 263 | GO:0045603 | positive regulation of endothelial cell differentiation | 5/10 | 36542/668746 | 9.73E-05 | 0.000707 | 5 |
| 264 | GO:0060537 | muscle tissue development | 189/2628 | 36542/668746 | 0.000105 | 0.000755 | 189 |
| 265 | GO:0045622 | regulation of T-helper cell differentiation | 9/36 | 36542/668746 | 0.000106 | 0.000755 | 9 |
| 266 | GO:1900408 | negative regulation of cellular response to oxidative stress | 9/36 | 36542/668746 | 0.000106 | 0.000755 | 9 |
| 267 | GO:1903202 | negative regulation of oxidative stress-induced cell death | 9/36 | 36542/668746 | 0.000106 | 0.000755 | 9 |
| 268 | GO:1902883 | negative regulation of response to oxidative stress | 9/36 | 36542/668746 | 0.000106 | 0.000755 | 9 |
| 269 | GO:0050880 | regulation of blood vessel size | 48/496 | 36542/668746 | 0.000112 | 0.000796 | 48 |
| 270 | GO:0035150 | regulation of tube size | 48/496 | 36542/668746 | 0.000112 | 0.000796 | 48 |
| 271 | GO:0014009 | glial cell proliferation | 15/91 | 36542/668746 | 0.000121 | 0.000846 | 15 |
| 272 | GO:0046638 | positive regulation of alpha-beta T cell differentiation | 15/91 | 36542/668746 | 0.000121 | 0.000846 | 15 |
| 273 | GO:2001251 | negative regulation of chromosome organization | 33/300 | 36542/668746 | 0.000122 | 0.000855 | 33 |
| 274 | GO:0097305 | response to alcohol | 105/1326 | 36542/668746 | 0.000124 | 0.000864 | 105 |
| 275 | GO:0003018 | vascular process in circulatory system | 55/595 | 36542/668746 | 0.000126 | 0.000869 | 55 |
| 276 | GO:0014002 | astrocyte development | 10/45 | 36542/668746 | 0.000129 | 0.000883 | 10 |
| 277 | GO:0045600 | positive regulation of fat cell differentiation | 10/45 | 36542/668746 | 0.000129 | 0.000883 | 10 |
| 278 | GO:0071901 | negative regulation of protein serine/threonine kinase activity | 65/741 | 36542/668746 | 0.000152 | 0.001035 | 65 |
| 279 | GO:0031396 | regulation of protein ubiquitination | 43/435 | 36542/668746 | 0.000155 | 0.001054 | 43 |
| 280 | GO:0061900 | glial cell activation | 11/55 | 36542/668746 | 0.000165 | 0.001115 | 11 |
| 281 | GO:0042274 | ribosomal small subunit biogenesis | 11/55 | 36542/668746 | 0.000165 | 0.001115 | 11 |
| 282 | GO:0051271 | negative regulation of cellular component movement | 88/1081 | 36542/668746 | 0.000168 | 0.001135 | 88 |
| 283 | GO:0070542 | response to fatty acid | 27/231 | 36542/668746 | 0.000181 | 0.001208 | 27 |
| 284 | GO:1903046 | meiotic cell cycle process | 62/703 | 36542/668746 | 0.000183 | 0.001219 | 62 |
| 285 | GO:0006690 | icosanoid metabolic process | 16/105 | 36542/668746 | 0.000188 | 0.001253 | 16 |
| 286 | GO:0071453 | cellular response to oxygen levels | 70/820 | 36542/668746 | 0.000194 | 0.001285 | 70 |
| 287 | GO:0035690 | cellular response to drug | 196/2775 | 36542/668746 | 0.000204 | 0.001349 | 196 |
| 288 | GO:0035051 | cardiocyte differentiation | 59/666 | 36542/668746 | 0.000229 | 0.001508 | 59 |
| 289 | GO:0098727 | maintenance of cell number | 36/351 | 36542/668746 | 0.000251 | 0.001644 | 36 |
| 290 | GO:1902903 | regulation of supramolecular fiber organization | 78/946 | 36542/668746 | 0.000257 | 0.001682 | 78 |
| 291 | GO:0048762 | mesenchymal cell differentiation | 90/1128 | 36542/668746 | 0.000278 | 0.001807 | 90 |
| 292 | GO:0033138 | positive regulation of peptidyl-serine phosphorylation | 17/120 | 36542/668746 | 0.000296 | 0.001909 | 17 |
| 293 | GO:0140014 | mitotic nuclear division | 147/2016 | 36542/668746 | 0.000316 | 0.002024 | 147 |
| 294 | GO:0030324 | lung development | 51/561 | 36542/668746 | 0.000319 | 0.002028 | 51 |
| 295 | GO:0030323 | respiratory tube development | 51/561 | 36542/668746 | 0.000319 | 0.002028 | 51 |
| 296 | GO:0009416 | response to light stimulus | 69/820 | 36542/668746 | 0.00032 | 0.002028 | 69 |
| 297 | GO:0016579 | protein deubiquitination | 51/561 | 36542/668746 | 0.000319 | 0.002028 | 51 |
| 298 | GO:0030278 | regulation of ossification | 58/666 | 36542/668746 | 0.000393 | 0.002488 | 58 |
| 299 | GO:0034614 | cellular response to reactive oxygen species | 53/595 | 36542/668746 | 0.000402 | 0.002528 | 53 |
| 300 | GO:0051216 | cartilage development | 102/1326 | 36542/668746 | 0.000419 | 0.002617 | 102 |
| 301 | GO:0045841 | negative regulation of mitotic metaphase/anaphase transition | 14/91 | 36542/668746 | 0.000419 | 0.002617 | 14 |
| 302 | GO:1902100 | negative regulation of metaphase/anaphase transition of cell cycle | 14/91 | 36542/668746 | 0.000419 | 0.002617 | 14 |
| 303 | GO:0050730 | regulation of peptidyl-tyrosine phosphorylation | 89/1128 | 36542/668746 | 0.000426 | 0.002635 | 89 |
| 304 | GO:1903038 | negative regulation of leukocyte cell-cell adhesion | 26/231 | 36542/668746 | 0.000424 | 0.002635 | 26 |
| 305 | GO:0043087 | regulation of GTPase activity | 89/1128 | 36542/668746 | 0.000426 | 0.002635 | 89 |
| 306 | GO:0046777 | protein autophosphorylation | 74/903 | 36542/668746 | 0.00043 | 0.002658 | 74 |
| 307 | GO:0042509 | regulation of tyrosine phosphorylation of STAT protein | 21/171 | 36542/668746 | 0.000462 | 0.002839 | 21 |
| 308 | GO:0070098 | chemokine-mediated signaling pathway | 21/171 | 36542/668746 | 0.000462 | 0.002839 | 21 |
| 309 | GO:0110020 | regulation of actomyosin structure organization | 18/136 | 36542/668746 | 0.000465 | 0.002851 | 18 |
| 310 | GO:0060560 | developmental growth involved in morphogenesis | 60/703 | 36542/668746 | 0.000526 | 0.003214 | 60 |
| 311 | GO:0070301 | cellular response to hydrogen peroxide | 24/210 | 36542/668746 | 0.000553 | 0.00337 | 24 |
| 312 | GO:0055002 | striated muscle cell development | 31/300 | 36542/668746 | 0.000567 | 0.003438 | 31 |
| 313 | GO:0031032 | actomyosin structure organization | 41/435 | 36542/668746 | 0.00057 | 0.003448 | 41 |
| 314 | GO:0042692 | muscle cell differentiation | 145/2016 | 36542/668746 | 0.000602 | 0.003598 | 145 |
| 315 | GO:0045907 | positive regulation of vasoconstriction | 8/36 | 36542/668746 | 0.000604 | 0.003598 | 8 |
| 316 | GO:0050873 | brown fat cell differentiation | 8/36 | 36542/668746 | 0.000604 | 0.003598 | 8 |
| 317 | GO:0042088 | T-helper 1 type immune response | 8/36 | 36542/668746 | 0.000604 | 0.003598 | 8 |
| 318 | GO:0003044 | regulation of systemic arterial blood pressure mediated by a chemical signal | 8/36 | 36542/668746 | 0.000604 | 0.003598 | 8 |
| 319 | GO:0040013 | negative regulation of locomotion | 82/1035 | 36542/668746 | 0.000613 | 0.003643 | 82 |
| 320 | GO:0001990 | regulation of systemic arterial blood pressure by hormone | 7/28 | 36542/668746 | 0.000621 | 0.00365 | 7 |
| 321 | GO:0030574 | collagen catabolic process | 7/28 | 36542/668746 | 0.000621 | 0.00365 | 7 |
| 322 | GO:0032873 | negative regulation of stress-activated MAPK cascade | 7/28 | 36542/668746 | 0.000621 | 0.00365 | 7 |
| 323 | GO:0070303 | negative regulation of stress-activated protein kinase signaling cascade | 7/28 | 36542/668746 | 0.000621 | 0.00365 | 7 |
| 324 | GO:1903201 | regulation of oxidative stress-induced cell death | 9/45 | 36542/668746 | 0.000638 | 0.003731 | 9 |
| 325 | GO:0051304 | chromosome separation | 29/276 | 36542/668746 | 0.000641 | 0.003739 | 29 |
| 326 | GO:2000146 | negative regulation of cell motility | 73/903 | 36542/668746 | 0.00068 | 0.003958 | 73 |
| 327 | GO:0045992 | negative regulation of embryonic development | 10/55 | 36542/668746 | 0.000718 | 0.00416 | 10 |
| 328 | GO:0032663 | regulation of interleukin-2 production | 10/55 | 36542/668746 | 0.000718 | 0.00416 | 10 |
| 329 | GO:0045839 | negative regulation of mitotic nuclear division | 19/153 | 36542/668746 | 0.000732 | 0.00422 | 19 |
| 330 | GO:0010565 | regulation of cellular ketone metabolic process | 19/153 | 36542/668746 | 0.000732 | 0.00422 | 19 |
| 331 | GO:0048013 | ephrin receptor signaling pathway | 22/190 | 36542/668746 | 0.00077 | 0.004405 | 22 |
| 332 | GO:0048608 | reproductive structure development | 282/4278 | 36542/668746 | 0.000855 | 0.004819 | 282 |
| 333 | GO:0061458 | reproductive system development | 282/4278 | 36542/668746 | 0.000855 | 0.004819 | 282 |
| 334 | GO:0010863 | positive regulation of phospholipase C activity | 11/66 | 36542/668746 | 0.000852 | 0.004819 | 11 |
| 335 | GO:1900274 | regulation of phospholipase C activity | 11/66 | 36542/668746 | 0.000852 | 0.004819 | 11 |
| 336 | GO:0072347 | response to anesthetic | 11/66 | 36542/668746 | 0.000852 | 0.004819 | 11 |
| 337 | GO:1901570 | fatty acid derivative biosynthetic process | 11/66 | 36542/668746 | 0.000852 | 0.004819 | 11 |
| 338 | GO:0043551 | regulation of phosphatidylinositol 3-kinase activity | 16/120 | 36542/668746 | 0.000863 | 0.004844 | 16 |
| 339 | GO:1903426 | regulation of reactive oxygen species biosynthetic process | 16/120 | 36542/668746 | 0.000863 | 0.004844 | 16 |
| 340 | GO:0048143 | astrocyte activation | 5/15 | 36542/668746 | 0.000921 | 0.005141 | 5 |
| 341 | GO:0034501 | protein localization to kinetochore | 5/15 | 36542/668746 | 0.000921 | 0.005141 | 5 |
| 342 | GO:0007088 | regulation of mitotic nuclear division | 81/1035 | 36542/668746 | 0.000934 | 0.005204 | 81 |
| 343 | GO:0010811 | positive regulation of cell-substrate adhesion | 25/231 | 36542/668746 | 0.000954 | 0.005304 | 25 |
| 344 | GO:0018105 | peptidyl-serine phosphorylation | 87/1128 | 36542/668746 | 0.000965 | 0.00535 | 87 |
| 345 | GO:0140013 | meiotic nuclear division | 49/561 | 36542/668746 | 0.000982 | 0.00543 | 49 |
| 346 | GO:0042129 | regulation of T cell proliferation | 36/378 | 36542/668746 | 0.000984 | 0.00543 | 36 |
| 347 | GO:0061383 | trabecula morphogenesis | 12/78 | 36542/668746 | 0.001053 | 0.005744 | 12 |
| 348 | GO:0032623 | interleukin-2 production | 12/78 | 36542/668746 | 0.001053 | 0.005744 | 12 |
| 349 | GO:0036473 | cell death in response to oxidative stress | 12/78 | 36542/668746 | 0.001053 | 0.005744 | 12 |
| 350 | GO:0014066 | regulation of phosphatidylinositol 3-kinase signaling | 32/325 | 36542/668746 | 0.001056 | 0.005745 | 32 |
| 351 | GO:0050670 | regulation of lymphocyte proliferation | 56/666 | 36542/668746 | 0.001104 | 0.00598 | 56 |
| 352 | GO:0032944 | regulation of mononuclear cell proliferation | 56/666 | 36542/668746 | 0.001104 | 0.00598 | 56 |
| 353 | GO:0002067 | glandular epithelial cell differentiation | 20/171 | 36542/668746 | 0.001149 | 0.006181 | 20 |
| 354 | GO:0003015 | heart process | 61/741 | 36542/668746 | 0.001149 | 0.006181 | 61 |
| 355 | GO:0050868 | negative regulation of T cell activation | 20/171 | 36542/668746 | 0.001149 | 0.006181 | 20 |
| 356 | GO:0034101 | erythrocyte homeostasis | 30/300 | 36542/668746 | 0.001162 | 0.006222 | 30 |
| 357 | GO:0030048 | actin filament-based movement | 30/300 | 36542/668746 | 0.001162 | 0.006222 | 30 |
| 358 | GO:0033047 | regulation of mitotic sister chromatid segregation | 23/210 | 36542/668746 | 0.001268 | 0.0067 | 23 |
| 359 | GO:0034502 | protein localization to chromosome | 23/210 | 36542/668746 | 0.001268 | 0.0067 | 23 |
| 360 | GO:0055017 | cardiac muscle tissue growth | 23/210 | 36542/668746 | 0.001268 | 0.0067 | 23 |
| 361 | GO:1903322 | positive regulation of protein modification by small protein conjugation or removal | 23/210 | 36542/668746 | 0.001268 | 0.0067 | 23 |
| 362 | GO:0050954 | sensory perception of mechanical stimulus | 23/210 | 36542/668746 | 0.001268 | 0.0067 | 23 |
| 363 | GO:0030336 | negative regulation of cell migration | 66/820 | 36542/668746 | 0.001314 | 0.006925 | 66 |
| 364 | GO:0045620 | negative regulation of lymphocyte differentiation | 13/91 | 36542/668746 | 0.001345 | 0.007074 | 13 |
| 365 | GO:0048738 | cardiac muscle tissue development | 86/1128 | 36542/668746 | 0.001428 | 0.007471 | 86 |
| 366 | GO:0003198 | epithelial to mesenchymal transition involved in endocardial cushion formation | 4/10 | 36542/668746 | 0.001433 | 0.007471 | 4 |
| 367 | GO:0032691 | negative regulation of interleukin-1 beta production | 4/10 | 36542/668746 | 0.001433 | 0.007471 | 4 |
| 368 | GO:0014829 | vascular smooth muscle contraction | 4/10 | 36542/668746 | 0.001433 | 0.007471 | 4 |
| 369 | GO:0071214 | cellular response to abiotic stimulus | 102/1378 | 36542/668746 | 0.001456 | 0.007553 | 102 |
| 370 | GO:0104004 | cellular response to environmental stimulus | 102/1378 | 36542/668746 | 0.001456 | 0.007553 | 102 |
| 371 | GO:0071695 | anatomical structure maturation | 48/561 | 36542/668746 | 0.001671 | 0.008649 | 48 |
| 372 | GO:0010518 | positive regulation of phospholipase activity | 14/105 | 36542/668746 | 0.001764 | 0.009072 | 14 |
| 373 | GO:0032651 | regulation of interleukin-1 beta production | 14/105 | 36542/668746 | 0.001764 | 0.009072 | 14 |
| 374 | GO:0031398 | positive regulation of protein ubiquitination | 21/190 | 36542/668746 | 0.001794 | 0.009188 | 21 |
| 375 | GO:0051781 | positive regulation of cell division | 18/153 | 36542/668746 | 0.001846 | 0.00935 | 18 |
| 376 | GO:0071478 | cellular response to radiation | 35/378 | 36542/668746 | 0.001844 | 0.00935 | 35 |
| 377 | GO:0002824 | positive regulation of adaptive immune response based on somatic recombination of immune receptors built from immunoglobulin superfamily domains | 18/153 | 36542/668746 | 0.001846 | 0.00935 | 18 |
| 378 | GO:0110053 | regulation of actin filament organization | 41/465 | 36542/668746 | 0.002016 | 0.010169 | 41 |
| 379 | GO:1901989 | positive regulation of cell cycle phase transition | 31/325 | 36542/668746 | 0.002054 | 0.010315 | 31 |
| 380 | GO:0051224 | negative regulation of protein transport | 31/325 | 36542/668746 | 0.002054 | 0.010315 | 31 |
| 381 | GO:0070252 | actin-mediated cell contraction | 24/231 | 36542/668746 | 0.002063 | 0.010339 | 24 |
| 382 | GO:0001701 | in utero embryonic development | 88/1176 | 36542/668746 | 0.002153 | 0.010767 | 88 |
| 383 | GO:1901992 | positive regulation of mitotic cell cycle phase transition | 29/300 | 36542/668746 | 0.002302 | 0.011435 | 29 |
| 384 | GO:0061041 | regulation of wound healing | 29/300 | 36542/668746 | 0.002302 | 0.011435 | 29 |
| 385 | GO:0042531 | positive regulation of tyrosine phosphorylation of STAT protein | 15/120 | 36542/668746 | 0.00236 | 0.01165 | 15 |
| 386 | GO:0060193 | positive regulation of lipase activity | 15/120 | 36542/668746 | 0.00236 | 0.01165 | 15 |
| 387 | GO:0032611 | interleukin-1 beta production | 15/120 | 36542/668746 | 0.00236 | 0.01165 | 15 |
| 388 | GO:0030218 | erythrocyte differentiation | 27/276 | 36542/668746 | 0.002696 | 0.013224 | 27 |
| 389 | GO:0007569 | cell aging | 27/276 | 36542/668746 | 0.002696 | 0.013224 | 27 |
| 390 | GO:0045667 | regulation of osteoblast differentiation | 27/276 | 36542/668746 | 0.002696 | 0.013224 | 27 |
| 391 | GO:0030038 | contractile actin filament bundle assembly | 19/171 | 36542/668746 | 0.002713 | 0.013253 | 19 |
| 392 | GO:0043149 | stress fiber assembly | 19/171 | 36542/668746 | 0.002713 | 0.013253 | 19 |
| 393 | GO:0071236 | cellular response to antibiotic | 47/561 | 36542/668746 | 0.002784 | 0.013459 | 47 |
| 394 | GO:1905818 | regulation of chromosome separation | 22/210 | 36542/668746 | 0.002783 | 0.013459 | 22 |
| 395 | GO:0010039 | response to iron ion | 8/45 | 36542/668746 | 0.002779 | 0.013459 | 8 |
| 396 | GO:0060251 | regulation of glial cell proliferation | 8/45 | 36542/668746 | 0.002779 | 0.013459 | 8 |
| 397 | GO:0051208 | sequestering of calcium ion | 22/210 | 36542/668746 | 0.002783 | 0.013459 | 22 |
| 398 | GO:0035886 | vascular smooth muscle cell differentiation | 9/55 | 36542/668746 | 0.0028 | 0.01348 | 9 |
| 399 | GO:0001954 | positive regulation of cell-matrix adhesion | 9/55 | 36542/668746 | 0.0028 | 0.01348 | 9 |
| 400 | GO:0032429 | regulation of phospholipase A2 activity | 3/6 | 36542/668746 | 0.002879 | 0.013777 | 3 |
| 401 | GO:0070307 | lens fiber cell development | 3/6 | 36542/668746 | 0.002879 | 0.013777 | 3 |
| 402 | GO:0036342 | post-anal tail morphogenesis | 3/6 | 36542/668746 | 0.002879 | 0.013777 | 3 |
| 403 | GO:0045429 | positive regulation of nitric oxide biosynthetic process | 10/66 | 36542/668746 | 0.003003 | 0.014255 | 10 |
| 404 | GO:1904407 | positive regulation of nitric oxide metabolic process | 10/66 | 36542/668746 | 0.003003 | 0.014255 | 10 |
| 405 | GO:0010611 | regulation of cardiac muscle hypertrophy | 10/66 | 36542/668746 | 0.003003 | 0.014255 | 10 |
| 406 | GO:0014743 | regulation of muscle hypertrophy | 10/66 | 36542/668746 | 0.003003 | 0.014255 | 10 |
| 407 | GO:0045132 | meiotic chromosome segregation | 16/136 | 36542/668746 | 0.003204 | 0.015056 | 16 |
| 408 | GO:0014812 | muscle cell migration | 16/136 | 36542/668746 | 0.003204 | 0.015056 | 16 |
| 409 | GO:1903409 | reactive oxygen species biosynthetic process | 16/136 | 36542/668746 | 0.003204 | 0.015056 | 16 |
| 410 | GO:0050806 | positive regulation of synaptic transmission | 16/136 | 36542/668746 | 0.003204 | 0.015056 | 16 |
| 411 | GO:0030178 | negative regulation of Wnt signaling pathway | 36/406 | 36542/668746 | 0.003297 | 0.015429 | 36 |
| 412 | GO:0033045 | regulation of sister chromatid segregation | 25/253 | 36542/668746 | 0.003311 | 0.015463 | 25 |
| 413 | GO:0007094 | mitotic spindle assembly checkpoint | 11/78 | 36542/668746 | 0.003389 | 0.015519 | 11 |
| 414 | GO:0031577 | spindle checkpoint | 11/78 | 36542/668746 | 0.003389 | 0.015519 | 11 |
| 415 | GO:0071173 | spindle assembly checkpoint | 11/78 | 36542/668746 | 0.003389 | 0.015519 | 11 |
| 416 | GO:0022408 | negative regulation of cell-cell adhesion | 34/378 | 36542/668746 | 0.003358 | 0.015519 | 34 |
| 417 | GO:1903428 | positive regulation of reactive oxygen species biosynthetic process | 11/78 | 36542/668746 | 0.003389 | 0.015519 | 11 |
| 418 | GO:0007015 | actin filament organization | 72/946 | 36542/668746 | 0.003384 | 0.015519 | 72 |
| 419 | GO:0030858 | positive regulation of epithelial cell differentiation | 11/78 | 36542/668746 | 0.003389 | 0.015519 | 11 |
| 420 | GO:0035914 | skeletal muscle cell differentiation | 11/78 | 36542/668746 | 0.003389 | 0.015519 | 11 |
| 421 | GO:1900407 | regulation of cellular response to oxidative stress | 11/78 | 36542/668746 | 0.003389 | 0.015519 | 11 |
| 422 | GO:1902882 | regulation of response to oxidative stress | 11/78 | 36542/668746 | 0.003389 | 0.015519 | 11 |
| 423 | GO:0002699 | positive regulation of immune effector process | 40/465 | 36542/668746 | 0.003478 | 0.015861 | 40 |
| 424 | GO:0031669 | cellular response to nutrient levels | 40/465 | 36542/668746 | 0.003478 | 0.015861 | 40 |
| 425 | GO:1904950 | negative regulation of establishment of protein localization | 32/351 | 36542/668746 | 0.003539 | 0.015957 | 32 |
| 426 | GO:0010165 | response to X-ray | 6/28 | 36542/668746 | 0.003533 | 0.015957 | 6 |
| 427 | GO:0045823 | positive regulation of heart contraction | 6/28 | 36542/668746 | 0.003533 | 0.015957 | 6 |
| 428 | GO:0006636 | unsaturated fatty acid biosynthetic process | 6/28 | 36542/668746 | 0.003533 | 0.015957 | 6 |
| 429 | GO:0045646 | regulation of erythrocyte differentiation | 6/28 | 36542/668746 | 0.003533 | 0.015957 | 6 |
| 430 | GO:0042445 | hormone metabolic process | 56/703 | 36542/668746 | 0.003552 | 0.015986 | 56 |
| 431 | GO:1902807 | negative regulation of cell cycle G1/S phase transition | 30/325 | 36542/668746 | 0.00387 | 0.017383 | 30 |
| 432 | GO:0051784 | negative regulation of nuclear division | 20/190 | 36542/668746 | 0.003985 | 0.017626 | 20 |
| 433 | GO:0050886 | endocrine process | 20/190 | 36542/668746 | 0.003985 | 0.017626 | 20 |
| 434 | GO:0035850 | epithelial cell differentiation involved in kidney development | 12/91 | 36542/668746 | 0.003983 | 0.017626 | 12 |
| 435 | GO:0051209 | release of sequestered calcium ion into cytosol | 20/190 | 36542/668746 | 0.003985 | 0.017626 | 20 |
| 436 | GO:0051283 | negative regulation of sequestering of calcium ion | 20/190 | 36542/668746 | 0.003985 | 0.017626 | 20 |
| 437 | GO:0051282 | regulation of sequestering of calcium ion | 20/190 | 36542/668746 | 0.003985 | 0.017626 | 20 |
| 438 | GO:0046209 | nitric oxide metabolic process | 12/91 | 36542/668746 | 0.003983 | 0.017626 | 12 |
| 439 | GO:2001057 | reactive nitrogen species metabolic process | 12/91 | 36542/668746 | 0.003983 | 0.017626 | 12 |
| 440 | GO:0060541 | respiratory system development | 66/861 | 36542/668746 | 0.004114 | 0.018163 | 66 |
| 441 | GO:0030071 | regulation of mitotic metaphase/anaphase transition | 17/153 | 36542/668746 | 0.004397 | 0.019374 | 17 |
| 442 | GO:0016570 | histone modification | 138/2016 | 36542/668746 | 0.004628 | 0.020316 | 138 |
| 443 | GO:0006909 | phagocytosis | 63/820 | 36542/668746 | 0.004712 | 0.020647 | 63 |
| 444 | GO:0042398 | cellular modified amino acid biosynthetic process | 5/21 | 36542/668746 | 0.00474 | 0.020734 | 5 |
| 445 | GO:0150076 | neuroinflammatory response | 13/105 | 36542/668746 | 0.004836 | 0.021074 | 13 |
| 446 | GO:0010718 | positive regulation of epithelial to mesenchymal transition | 13/105 | 36542/668746 | 0.004836 | 0.021074 | 13 |
| 447 | GO:0019216 | regulation of lipid metabolic process | 162/2415 | 36542/668746 | 0.004981 | 0.021663 | 162 |
| 448 | GO:0060419 | heart growth | 26/276 | 36542/668746 | 0.005233 | 0.022717 | 26 |
| 449 | GO:0070997 | neuron death | 108/1540 | 36542/668746 | 0.00563 | 0.024395 | 108 |
| 450 | GO:0051495 | positive regulation of cytoskeleton organization | 37/435 | 36542/668746 | 0.005716 | 0.024721 | 37 |
| 451 | GO:0048146 | positive regulation of fibroblast proliferation | 21/210 | 36542/668746 | 0.005834 | 0.025133 | 21 |
| 452 | GO:0045834 | positive regulation of lipid metabolic process | 39/465 | 36542/668746 | 0.005854 | 0.025133 | 39 |
| 453 | GO:0045778 | positive regulation of ossification | 21/210 | 36542/668746 | 0.005834 | 0.025133 | 21 |
| 454 | GO:0055007 | cardiac muscle cell differentiation | 33/378 | 36542/668746 | 0.005941 | 0.025401 | 33 |
| 455 | GO:0051492 | regulation of stress fiber assembly | 14/120 | 36542/668746 | 0.006025 | 0.025679 | 14 |
| 456 | GO:0010977 | negative regulation of neuron projection development | 18/171 | 36542/668746 | 0.006073 | 0.02584 | 18 |
| 457 | GO:1901214 | regulation of neuron death | 82/1128 | 36542/668746 | 0.006098 | 0.025896 | 82 |
| 458 | GO:0022604 | regulation of cell morphogenesis | 118/1711 | 36542/668746 | 0.00659 | 0.027887 | 118 |
| 459 | GO:0006869 | lipid transport | 57/741 | 36542/668746 | 0.006696 | 0.028282 | 57 |
| 460 | GO:0071248 | cellular response to metal ion | 29/325 | 36542/668746 | 0.007056 | 0.029748 | 29 |
| 461 | GO:0003376 | sphingosine-1-phosphate receptor signaling pathway | 4/15 | 36542/668746 | 0.007479 | 0.031138 | 4 |
| 462 | GO:0090520 | sphingolipid mediated signaling pathway | 4/15 | 36542/668746 | 0.007479 | 0.031138 | 4 |
| 463 | GO:2000696 | regulation of epithelial cell differentiation involved in kidney development | 4/15 | 36542/668746 | 0.007479 | 0.031138 | 4 |
| 464 | GO:0002827 | positive regulation of T-helper 1 type immune response | 4/15 | 36542/668746 | 0.007479 | 0.031138 | 4 |
| 465 | GO:0071425 | hematopoietic stem cell proliferation | 4/15 | 36542/668746 | 0.007479 | 0.031138 | 4 |
| 466 | GO:0001783 | B cell apoptotic process | 4/15 | 36542/668746 | 0.007479 | 0.031138 | 4 |
| 467 | GO:1901976 | regulation of cell cycle checkpoint | 4/15 | 36542/668746 | 0.007479 | 0.031138 | 4 |
| 468 | GO:0030900 | forebrain development | 173/2628 | 36542/668746 | 0.007649 | 0.031707 | 173 |
| 469 | GO:0061614 | pri-miRNA transcription by RNA polymerase II | 15/136 | 36542/668746 | 0.007656 | 0.031707 | 15 |
| 470 | GO:0007565 | female pregnancy | 67/903 | 36542/668746 | 0.007883 | 0.032534 | 67 |
| 471 | GO:0045682 | regulation of epidermis development | 19/190 | 36542/668746 | 0.008417 | 0.034495 | 19 |
| 472 | GO:0050709 | negative regulation of protein secretion | 22/231 | 36542/668746 | 0.008496 | 0.034696 | 22 |
| 473 | GO:0002792 | negative regulation of peptide secretion | 22/231 | 36542/668746 | 0.008496 | 0.034696 | 22 |
| 474 | GO:1904377 | positive regulation of protein localization to cell periphery | 9/66 | 36542/668746 | 0.009534 | 0.038734 | 9 |
| 475 | GO:0033627 | cell adhesion mediated by integrin | 9/66 | 36542/668746 | 0.009534 | 0.038734 | 9 |
| 476 | GO:0002762 | negative regulation of myeloid leukocyte differentiation | 8/55 | 36542/668746 | 0.009696 | 0.03912 | 8 |
| 477 | GO:0097755 | positive regulation of blood vessel diameter | 8/55 | 36542/668746 | 0.009696 | 0.03912 | 8 |
| 478 | GO:0003143 | embryonic heart tube morphogenesis | 8/55 | 36542/668746 | 0.009696 | 0.03912 | 8 |
| 479 | GO:1901655 | cellular response to ketone | 25/276 | 36542/668746 | 0.009781 | 0.039256 | 25 |
| 480 | GO:0018107 | peptidyl-threonine phosphorylation | 25/276 | 36542/668746 | 0.009781 | 0.039256 | 25 |
| 481 | GO:1905477 | positive regulation of protein localization to membrane | 25/276 | 36542/668746 | 0.009781 | 0.039256 | 25 |
| 482 | GO:0034508 | centromere complex assembly | 16/153 | 36542/668746 | 0.009874 | 0.039362 | 16 |
| 483 | GO:1903725 | regulation of phospholipid metabolic process | 16/153 | 36542/668746 | 0.009874 | 0.039362 | 16 |
| 484 | GO:0003300 | cardiac muscle hypertrophy | 16/153 | 36542/668746 | 0.009874 | 0.039362 | 16 |
| 485 | GO:0014897 | striated muscle hypertrophy | 16/153 | 36542/668746 | 0.009874 | 0.039362 | 16 |
| 486 | GO:0045428 | regulation of nitric oxide biosynthetic process | 10/78 | 36542/668746 | 0.009927 | 0.039373 | 10 |
| 487 | GO:0006809 | nitric oxide biosynthetic process | 10/78 | 36542/668746 | 0.009927 | 0.039373 | 10 |
| 488 | GO:0032092 | positive regulation of protein binding | 10/78 | 36542/668746 | 0.009927 | 0.039373 | 10 |
| 489 | GO:0030307 | positive regulation of cell growth | 32/378 | 36542/668746 | 0.010205 | 0.040337 | 32 |
| 490 | GO:0043552 | positive regulation of phosphatidylinositol 3-kinase activity | 7/45 | 36542/668746 | 0.01058 | 0.041331 | 7 |
| 491 | GO:0090218 | positive regulation of lipid kinase activity | 7/45 | 36542/668746 | 0.01058 | 0.041331 | 7 |
| 492 | GO:0045843 | negative regulation of striated muscle tissue development | 7/45 | 36542/668746 | 0.01058 | 0.041331 | 7 |
| 493 | GO:0048635 | negative regulation of muscle organ development | 7/45 | 36542/668746 | 0.01058 | 0.041331 | 7 |
| 494 | GO:1901862 | negative regulation of muscle tissue development | 7/45 | 36542/668746 | 0.01058 | 0.041331 | 7 |
| 495 | GO:0006801 | superoxide metabolic process | 7/45 | 36542/668746 | 0.01058 | 0.041331 | 7 |
| 496 | GO:0031018 | endocrine pancreas development | 11/91 | 36542/668746 | 0.010829 | 0.042024 | 11 |
| 497 | GO:0051057 | positive regulation of small GTPase mediated signal transduction | 11/91 | 36542/668746 | 0.010829 | 0.042024 | 11 |
| 498 | GO:0001736 | establishment of planar polarity | 11/91 | 36542/668746 | 0.010829 | 0.042024 | 11 |
| 499 | GO:0007164 | establishment of tissue polarity | 11/91 | 36542/668746 | 0.010829 | 0.042024 | 11 |
| 500 | GO:1904951 | positive regulation of establishment of protein localization | 189/2926 | 36542/668746 | 0.011199 | 0.043386 | 189 |
| 501 | GO:0007093 | mitotic cell cycle checkpoint | 66/903 | 36542/668746 | 0.011284 | 0.043508 | 66 |
| 502 | GO:0001837 | epithelial to mesenchymal transition | 44/561 | 36542/668746 | 0.011342 | 0.043508 | 44 |
| 503 | GO:0010959 | regulation of metal ion transport | 116/1711 | 36542/668746 | 0.0113 | 0.043508 | 116 |
| 504 | GO:0010389 | regulation of G2/M transition of mitotic cell cycle | 44/561 | 36542/668746 | 0.011342 | 0.043508 | 44 |
| 505 | GO:0043279 | response to alkaloid | 20/210 | 36542/668746 | 0.01167 | 0.044547 | 20 |
| 506 | GO:0043500 | muscle adaptation | 20/210 | 36542/668746 | 0.01167 | 0.044547 | 20 |
| 507 | GO:0007519 | skeletal muscle tissue development | 20/210 | 36542/668746 | 0.01167 | 0.044547 | 20 |
| 508 | GO:0000086 | G2/M transition of mitotic cell cycle | 58/780 | 36542/668746 | 0.012068 | 0.045992 | 58 |
| 509 | GO:0045787 | positive regulation of cell cycle | 212/3321 | 36542/668746 | 0.0121 | 0.04604 | 212 |
| 510 | GO:0060740 | prostate gland epithelium morphogenesis | 12/105 | 36542/668746 | 0.012267 | 0.046223 | 12 |
| 511 | GO:0060512 | prostate gland morphogenesis | 12/105 | 36542/668746 | 0.012267 | 0.046223 | 12 |
| 512 | GO:0031055 | chromatin remodeling at centromere | 12/105 | 36542/668746 | 0.012267 | 0.046223 | 12 |
| 513 | GO:0055021 | regulation of cardiac muscle tissue growth | 12/105 | 36542/668746 | 0.012267 | 0.046223 | 12 |
| 514 | GO:0033143 | regulation of intracellular steroid hormone receptor signaling pathway | 12/105 | 36542/668746 | 0.012267 | 0.046223 | 12 |
| 515 | GO:0071456 | cellular response to hypoxia | 53/703 | 36542/668746 | 0.01239 | 0.046536 | 53 |
| 516 | GO:0036294 | cellular response to decreased oxygen levels | 53/703 | 36542/668746 | 0.01239 | 0.046536 | 53 |
| 517 | GO:0071459 | protein localization to chromosome, centromeric region | 6/36 | 36542/668746 | 0.01259 | 0.047135 | 6 |
| 518 | GO:0046620 | regulation of organ growth | 17/171 | 36542/668746 | 0.012871 | 0.047882 | 17 |
| 519 | GO:0014896 | muscle hypertrophy | 17/171 | 36542/668746 | 0.012871 | 0.047882 | 17 |
| 520 | GO:0031099 | regeneration | 71/990 | 36542/668746 | 0.013273 | 0.049299 | 71 |

**Experiment group:**

| **NO.** | **ID** | **Description** | **GeneRatio** | **BgRatio** | **pvalue** | **p.adjust** | **count** |
| --- | --- | --- | --- | --- | --- | --- | --- |
| 1 | GO:0002460 | adaptive immune response based on somatic recombination of immune receptors built from immunoglobulin superfamily domains | 98/741 | 31426/668746 | 0 | 0 | 98 |
| 2 | GO:0042113 | B cell activation | 99/946 | 31426/668746 | 2.35E-13 | 7.85E-11 | 99 |
| 3 | GO:0007200 | phospholipase C-activating G protein-coupled receptor signaling pathway | 45/300 | 31426/668746 | 8.4E-12 | 2.18E-09 | 45 |
| 4 | GO:0071824 | protein-DNA complex subunit organization | 132/1540 | 31426/668746 | 5.75E-11 | 1.33E-08 | 132 |
| 5 | GO:0090287 | regulation of cellular response to growth factor stimulus | 100/1081 | 31426/668746 | 2.08E-10 | 4.06E-08 | 100 |
| 6 | GO:0006401 | RNA catabolic process | 57/496 | 31426/668746 | 7.98E-10 | 1.33E-07 | 57 |
| 7 | GO:0060964 | regulation of gene silencing by miRNA | 21/91 | 31426/668746 | 1.16E-09 | 1.81E-07 | 21 |
| 8 | GO:0048705 | skeletal system morphogenesis | 67/666 | 31426/668746 | 7.2E-09 | 8.43E-07 | 67 |
| 9 | GO:0060968 | regulation of gene silencing | 28/171 | 31426/668746 | 9.28E-09 | 9.87E-07 | 28 |
| 10 | GO:0032649 | regulation of interferon-gamma production | 26/153 | 31426/668746 | 1.4E-08 | 1.21E-06 | 26 |
| 11 | GO:0032609 | interferon-gamma production | 26/153 | 31426/668746 | 1.4E-08 | 1.21E-06 | 26 |
| 12 | GO:0045637 | regulation of myeloid cell differentiation | 94/1081 | 31426/668746 | 1.47E-08 | 1.23E-06 | 94 |
| 13 | GO:0060147 | regulation of posttranscriptional gene silencing | 21/105 | 31426/668746 | 1.78E-08 | 1.41E-06 | 21 |
| 14 | GO:0031349 | positive regulation of defense response | 174/2415 | 31426/668746 | 3.41E-08 | 2.57E-06 | 174 |
| 15 | GO:0006342 | chromatin silencing | 27/171 | 31426/668746 | 3.71E-08 | 2.71E-06 | 27 |
| 16 | GO:0043406 | positive regulation of MAP kinase activity | 133/1770 | 31426/668746 | 1.36E-07 | 9.1E-06 | 133 |
| 17 | GO:0060349 | bone morphogenesis | 31/231 | 31426/668746 | 1.77E-07 | 1.12E-05 | 31 |
| 18 | GO:0030947 | regulation of vascular endothelial growth factor receptor signaling pathway | 10/28 | 31426/668746 | 3.15E-07 | 1.89E-05 | 10 |
| 19 | GO:0002819 | regulation of adaptive immune response | 45/435 | 31426/668746 | 8.81E-07 | 4.91E-05 | 45 |
| 20 | GO:0048704 | embryonic skeletal system morphogenesis | 17/91 | 31426/668746 | 1.08E-06 | 5.74E-05 | 17 |
| 21 | GO:0097094 | craniofacial suture morphogenesis | 5/6 | 31426/668746 | 1.32E-06 | 6.87E-05 | 5 |
| 22 | GO:0097193 | intrinsic apoptotic signaling pathway | 154/2211 | 31426/668746 | 1.36E-06 | 6.92E-05 | 154 |
| 23 | GO:0045088 | regulation of innate immune response | 105/1378 | 31426/668746 | 1.39E-06 | 6.92E-05 | 105 |
| 24 | GO:0072331 | signal transduction by p53 class mediator | 81/990 | 31426/668746 | 1.52E-06 | 7.41E-05 | 81 |
| 25 | GO:0032479 | regulation of type I interferon production | 21/136 | 31426/668746 | 1.67E-06 | 7.67E-05 | 21 |
| 26 | GO:0032606 | type I interferon production | 21/136 | 31426/668746 | 1.67E-06 | 7.67E-05 | 21 |
| 27 | GO:0045814 | negative regulation of gene expression, epigenetic | 29/231 | 31426/668746 | 1.73E-06 | 7.77E-05 | 29 |
| 28 | GO:0046578 | regulation of Ras protein signal transduction | 43/435 | 31426/668746 | 4.85E-06 | 0.000199 | 43 |
| 29 | GO:0001776 | leukocyte homeostasis | 23/171 | 31426/668746 | 6.15E-06 | 0.000228 | 23 |
| 30 | GO:0090596 | sensory organ morphogenesis | 78/990 | 31426/668746 | 9.03E-06 | 0.000315 | 78 |
| 31 | GO:0000723 | telomere maintenance | 38/378 | 31426/668746 | 1.14E-05 | 0.000374 | 38 |
| 32 | GO:0007266 | Rho protein signal transduction | 38/378 | 31426/668746 | 1.14E-05 | 0.000374 | 38 |
| 33 | GO:0031331 | positive regulation of cellular catabolic process | 64/780 | 31426/668746 | 1.6E-05 | 0.000507 | 64 |
| 34 | GO:0034340 | response to type I interferon | 22/171 | 31426/668746 | 1.97E-05 | 0.000583 | 22 |
| 35 | GO:0048706 | embryonic skeletal system development | 22/171 | 31426/668746 | 1.97E-05 | 0.000583 | 22 |
| 36 | GO:0032729 | positive regulation of interferon-gamma production | 15/91 | 31426/668746 | 2.2E-05 | 0.000621 | 15 |
| 37 | GO:0045652 | regulation of megakaryocyte differentiation | 15/91 | 31426/668746 | 2.2E-05 | 0.000621 | 15 |
| 38 | GO:0050921 | positive regulation of chemotaxis | 43/465 | 31426/668746 | 2.47E-05 | 0.000679 | 43 |
| 39 | GO:0042770 | signal transduction in response to DNA damage | 37/378 | 31426/668746 | 2.62E-05 | 0.000689 | 37 |
| 40 | GO:0007281 | germ cell development | 45/496 | 31426/668746 | 2.59E-05 | 0.000689 | 45 |
| 41 | GO:0022412 | cellular process involved in reproduction in multicellular organism | 76/990 | 31426/668746 | 2.77E-05 | 0.000713 | 76 |
| 42 | GO:0030902 | hindbrain development | 23/190 | 31426/668746 | 3.45E-05 | 0.000869 | 23 |
| 43 | GO:0001773 | myeloid dendritic cell activation | 6/15 | 31426/668746 | 3.73E-05 | 0.000928 | 6 |
| 44 | GO:0006352 | DNA-templated transcription, initiation | 101/1431 | 31426/668746 | 4.72E-05 | 0.001152 | 101 |
| 45 | GO:0019882 | antigen processing and presentation | 29/276 | 31426/668746 | 5.26E-05 | 0.001257 | 29 |
| 46 | GO:0032200 | telomere organization | 38/406 | 31426/668746 | 5.43E-05 | 0.001284 | 38 |
| 47 | GO:0002218 | activation of innate immune response | 64/820 | 31426/668746 | 7E-05 | 0.001621 | 64 |
| 48 | GO:0048568 | embryonic organ development | 159/2485 | 31426/668746 | 7.82E-05 | 0.001794 | 159 |
| 49 | GO:0010332 | response to gamma radiation | 15/105 | 31426/668746 | 0.000121 | 0.002611 | 15 |
| 50 | GO:0060333 | interferon-gamma-mediated signaling pathway | 15/105 | 31426/668746 | 0.000121 | 0.002611 | 15 |
| 51 | GO:0015980 | energy derivation by oxidation of organic compounds | 35/378 | 31426/668746 | 0.000128 | 0.002754 | 35 |
| 52 | GO:0043583 | ear development | 49/595 | 31426/668746 | 0.000132 | 0.002792 | 49 |
| 53 | GO:0048592 | eye morphogenesis | 33/351 | 31426/668746 | 0.000148 | 0.003086 | 33 |
| 54 | GO:0014068 | positive regulation of phosphatidylinositol 3-kinase signaling | 23/210 | 31426/668746 | 0.000161 | 0.00328 | 23 |
| 55 | GO:2001252 | positive regulation of chromosome organization | 42/496 | 31426/668746 | 0.000213 | 0.004144 | 42 |
| 56 | GO:0070231 | T cell apoptotic process | 10/55 | 31426/668746 | 0.000219 | 0.004183 | 10 |
| 57 | GO:0042769 | DNA damage response, detection of DNA damage | 8/36 | 31426/668746 | 0.00022 | 0.004183 | 8 |
| 58 | GO:0010821 | regulation of mitochondrion organization | 38/435 | 31426/668746 | 0.000224 | 0.004186 | 38 |
| 59 | GO:0001569 | branching involved in blood vessel morphogenesis | 7/28 | 31426/668746 | 0.00025 | 0.004635 | 7 |
| 60 | GO:0009896 | positive regulation of catabolic process | 94/1378 | 31426/668746 | 0.000268 | 0.004931 | 94 |
| 61 | GO:0009895 | negative regulation of catabolic process | 34/378 | 31426/668746 | 0.000272 | 0.004966 | 34 |
| 62 | GO:0071480 | cellular response to gamma radiation | 6/21 | 31426/668746 | 0.000317 | 0.005528 | 6 |
| 63 | GO:0060325 | face morphogenesis | 6/21 | 31426/668746 | 0.000317 | 0.005528 | 6 |
| 64 | GO:0060323 | head morphogenesis | 6/21 | 31426/668746 | 0.000317 | 0.005528 | 6 |
| 65 | GO:0007492 | endoderm development | 18/153 | 31426/668746 | 0.000332 | 0.005752 | 18 |
| 66 | GO:0034728 | nucleosome organization | 43/528 | 31426/668746 | 0.00041 | 0.006977 | 43 |
| 67 | GO:0006303 | double-strand break repair via nonhomologous end joining | 14/105 | 31426/668746 | 0.000414 | 0.006977 | 14 |
| 68 | GO:0002706 | regulation of lymphocyte mediated immunity | 22/210 | 31426/668746 | 0.00041 | 0.006977 | 22 |
| 69 | GO:0032680 | regulation of tumor necrosis factor production | 37/435 | 31426/668746 | 0.000445 | 0.007329 | 37 |
| 70 | GO:1903555 | regulation of tumor necrosis factor superfamily cytokine production | 37/435 | 31426/668746 | 0.000445 | 0.007329 | 37 |
| 71 | GO:0060443 | mammary gland morphogenesis | 15/120 | 31426/668746 | 0.000529 | 0.008369 | 15 |
| 72 | GO:0030219 | megakaryocyte differentiation | 15/120 | 31426/668746 | 0.000529 | 0.008369 | 15 |
| 73 | GO:0042176 | regulation of protein catabolic process | 78/1128 | 31426/668746 | 0.000565 | 0.008815 | 78 |
| 74 | GO:0043900 | regulation of multi-organism process | 65/903 | 31426/668746 | 0.000562 | 0.008815 | 65 |
| 75 | GO:0051169 | nuclear transport | 70/990 | 31426/668746 | 0.000579 | 0.008975 | 70 |
| 76 | GO:1904705 | regulation of vascular smooth muscle cell proliferation | 16/136 | 31426/668746 | 0.000691 | 0.010304 | 16 |
| 77 | GO:1990874 | vascular smooth muscle cell proliferation | 16/136 | 31426/668746 | 0.000691 | 0.010304 | 16 |
| 78 | GO:0060337 | type I interferon signaling pathway | 16/136 | 31426/668746 | 0.000691 | 0.010304 | 16 |
| 79 | GO:0071357 | cellular response to type I interferon | 16/136 | 31426/668746 | 0.000691 | 0.010304 | 16 |
| 80 | GO:0051607 | defense response to virus | 29/325 | 31426/668746 | 0.000825 | 0.01185 | 29 |
| 81 | GO:0010639 | negative regulation of organelle organization | 77/1128 | 31426/668746 | 0.000871 | 0.012429 | 77 |
| 82 | GO:0140056 | organelle localization by membrane tethering | 17/153 | 31426/668746 | 0.000919 | 0.012959 | 17 |
| 83 | GO:0030330 | DNA damage response, signal transduction by p53 class mediator | 24/253 | 31426/668746 | 0.000964 | 0.013505 | 24 |
| 84 | GO:0036092 | phosphatidylinositol-3-phosphate biosynthetic process | 9/55 | 31426/668746 | 0.000995 | 0.013695 | 9 |
| 85 | GO:0002709 | regulation of T cell mediated immunity | 9/55 | 31426/668746 | 0.000995 | 0.013695 | 9 |
| 86 | GO:0060324 | face development | 11/78 | 31426/668746 | 0.00104 | 0.014153 | 11 |
| 87 | GO:0014910 | regulation of smooth muscle cell migration | 11/78 | 31426/668746 | 0.00104 | 0.014153 | 11 |
| 88 | GO:1904707 | positive regulation of vascular smooth muscle cell proliferation | 8/45 | 31426/668746 | 0.001075 | 0.014539 | 8 |
| 89 | GO:0097711 | ciliary basal body-plasma membrane docking | 12/91 | 31426/668746 | 0.001149 | 0.015369 | 12 |
| 90 | GO:0002504 | antigen processing and presentation of peptide or polysaccharide antigen via MHC class II | 12/91 | 31426/668746 | 0.001149 | 0.015369 | 12 |
| 91 | GO:0048066 | developmental pigmentation | 7/36 | 31426/668746 | 0.001266 | 0.016826 | 7 |
| 92 | GO:0019079 | viral genome replication | 13/105 | 31426/668746 | 0.001322 | 0.017475 | 13 |
| 93 | GO:2001242 | regulation of intrinsic apoptotic signaling pathway | 56/780 | 31426/668746 | 0.001356 | 0.017724 | 56 |
| 94 | GO:0032640 | tumor necrosis factor production | 37/465 | 31426/668746 | 0.001483 | 0.018752 | 37 |
| 95 | GO:0071706 | tumor necrosis factor superfamily cytokine production | 37/465 | 31426/668746 | 0.001483 | 0.018752 | 37 |
| 96 | GO:0043112 | receptor metabolic process | 25/276 | 31426/668746 | 0.001468 | 0.018752 | 25 |
| 97 | GO:0051973 | positive regulation of telomerase activity | 6/28 | 31426/668746 | 0.001656 | 0.020507 | 6 |
| 98 | GO:0051972 | regulation of telomerase activity | 6/28 | 31426/668746 | 0.001656 | 0.020507 | 6 |
| 99 | GO:0051353 | positive regulation of oxidoreductase activity | 6/28 | 31426/668746 | 0.001656 | 0.020507 | 6 |
| 100 | GO:1901796 | regulation of signal transduction by p53 class mediator | 28/325 | 31426/668746 | 0.001688 | 0.020698 | 28 |
| 101 | GO:0060485 | mesenchyme development | 114/1830 | 31426/668746 | 0.001739 | 0.021193 | 114 |
| 102 | GO:0018205 | peptidyl-lysine modification | 70/1035 | 31426/668746 | 0.001808 | 0.021916 | 70 |
| 103 | GO:0043011 | myeloid dendritic cell differentiation | 3/6 | 31426/668746 | 0.001864 | 0.022368 | 3 |
| 104 | GO:0001822 | kidney development | 107/1711 | 31426/668746 | 0.002078 | 0.024317 | 107 |
| 105 | GO:0006403 | RNA localization | 23/253 | 31426/668746 | 0.002111 | 0.024574 | 23 |
| 106 | GO:0090288 | negative regulation of cellular response to growth factor stimulus | 31/378 | 31426/668746 | 0.002151 | 0.02492 | 31 |
| 107 | GO:0070266 | necroptotic process | 5/21 | 31426/668746 | 0.002475 | 0.028114 | 5 |
| 108 | GO:0009112 | nucleobase metabolic process | 5/21 | 31426/668746 | 0.002475 | 0.028114 | 5 |
| 109 | GO:0002224 | toll-like receptor signaling pathway | 29/351 | 31426/668746 | 0.002624 | 0.029518 | 29 |
| 110 | GO:0006333 | chromatin assembly or disassembly | 36/465 | 31426/668746 | 0.002679 | 0.029852 | 36 |
| 111 | GO:0048562 | embryonic organ morphogenesis | 69/1035 | 31426/668746 | 0.002731 | 0.029997 | 69 |
| 112 | GO:0032760 | positive regulation of tumor necrosis factor production | 17/171 | 31426/668746 | 0.003049 | 0.032732 | 17 |
| 113 | GO:0032642 | regulation of chemokine production | 17/171 | 31426/668746 | 0.003049 | 0.032732 | 17 |
| 114 | GO:1903557 | positive regulation of tumor necrosis factor superfamily cytokine production | 17/171 | 31426/668746 | 0.003049 | 0.032732 | 17 |
| 115 | GO:0043473 | pigmentation | 10/78 | 31426/668746 | 0.003543 | 0.037684 | 10 |
| 116 | GO:0002495 | antigen processing and presentation of peptide antigen via MHC class II | 10/78 | 31426/668746 | 0.003543 | 0.037684 | 10 |
| 117 | GO:0031023 | microtubule organizing center organization | 12/105 | 31426/668746 | 0.003895 | 0.041052 | 12 |
| 118 | GO:0048002 | antigen processing and presentation of peptide antigen | 18/190 | 31426/668746 | 0.003932 | 0.04108 | 18 |
| 119 | GO:0065004 | protein-DNA complex assembly | 73/1128 | 31426/668746 | 0.004322 | 0.04414 | 73 |
| 120 | GO:2000045 | regulation of G1/S transition of mitotic cell cycle | 37/496 | 31426/668746 | 0.004339 | 0.04414 | 37 |
| 121 | GO:0032722 | positive regulation of chemokine production | 13/120 | 31426/668746 | 0.004337 | 0.04414 | 13 |
| 122 | GO:0035019 | somatic stem cell population maintenance | 13/120 | 31426/668746 | 0.004337 | 0.04414 | 13 |
| 123 | GO:1901213 | regulation of transcription from RNA polymerase II promoter involved in heart development | 4/15 | 31426/668746 | 0.004382 | 0.04439 | 4 |
| 124 | GO:2000134 | negative regulation of G1/S transition of mitotic cell cycle | 25/300 | 31426/668746 | 0.00442 | 0.044467 | 25 |
| 125 | GO:0006275 | regulation of DNA replication | 22/253 | 31426/668746 | 0.004428 | 0.044467 | 22 |
| 126 | GO:0035107 | appendage morphogenesis | 35/465 | 31426/668746 | 0.004712 | 0.046716 | 35 |
| 127 | GO:0035108 | limb morphogenesis | 35/465 | 31426/668746 | 0.004712 | 0.046716 | 35 |
| 128 | GO:0001959 | regulation of cytokine-mediated signaling pathway | 35/465 | 31426/668746 | 0.004712 | 0.046716 | 35 |
| 129 | GO:0042551 | neuron maturation | 7/45 | 31426/668746 | 0.004761 | 0.046812 | 7 |
| 130 | GO:0030199 | collagen fibril organization | 14/136 | 31426/668746 | 0.004983 | 0.048791 | 14 |
| 131 | GO:0032602 | chemokine production | 19/210 | 31426/668746 | 0.005125 | 0.049766 | 19 |

**Common:**

| No. | ID | Description | GeneRatio | BgRatio | pvalue | p.adjust | count |
| --- | --- | --- | --- | --- | --- | --- | --- |
| 1 | GO:0043062 | extracellular structure organization | 301/3321 | 31426/668746 | 0 | 0 | 301 |
| 2 | GO:0030098 | lymphocyte differentiation | 245/2415 | 31426/668746 | 0 | 0 | 245 |
| 3 | GO:0050727 | regulation of inflammatory response | 181/2080 | 31426/668746 | 5.11E-15 | 2.99E-12 | 181 |
| 4 | GO:0007249 | I-kappaB kinase/NF-kappaB signaling | 81/666 | 31426/668746 | 1.29E-14 | 6.03E-12 | 81 |
| 5 | GO:0042110 | T cell activation | 251/3240 | 31426/668746 | 2.65E-14 | 1.03E-11 | 251 |
| 6 | GO:0032103 | positive regulation of response to external stimulus | 148/1711 | 31426/668746 | 2.17E-12 | 6.35E-10 | 148 |
| 7 | GO:0022613 | ribonucleoprotein complex biogenesis | 108/1176 | 31426/668746 | 6.26E-11 | 1.33E-08 | 108 |
| 8 | GO:0001704 | formation of primary germ layer | 62/561 | 31426/668746 | 7.16E-10 | 1.29E-07 | 62 |
| 9 | GO:0001654 | eye development | 119/1431 | 31426/668746 | 2.76E-09 | 3.58E-07 | 119 |
| 10 | GO:0150063 | visual system development | 119/1431 | 31426/668746 | 2.76E-09 | 3.58E-07 | 119 |
| 11 | GO:0048880 | sensory system development | 119/1431 | 31426/668746 | 2.76E-09 | 3.58E-07 | 119 |
| 12 | GO:0006364 | rRNA processing | 42/325 | 31426/668746 | 4.18E-09 | 5.14E-07 | 42 |
| 13 | GO:0009615 | response to virus | 83/903 | 31426/668746 | 8.77E-09 | 9.77E-07 | 83 |
| 14 | GO:0034341 | response to interferon-gamma | 52/465 | 31426/668746 | 1.06E-08 | 1.04E-06 | 52 |
| 15 | GO:0060348 | bone development | 52/465 | 31426/668746 | 1.06E-08 | 1.04E-06 | 52 |
| 16 | GO:0001819 | positive regulation of cytokine production | 172/2346 | 31426/668746 | 1.27E-08 | 1.19E-06 | 172 |
| 17 | GO:0016072 | rRNA metabolic process | 47/406 | 31426/668746 | 1.81E-08 | 1.41E-06 | 47 |
| 18 | GO:2000379 | positive regulation of reactive oxygen species metabolic process | 34/253 | 31426/668746 | 4.49E-08 | 3.19E-06 | 34 |
| 19 | GO:0002526 | acute inflammatory response | 41/351 | 31426/668746 | 1.09E-07 | 7.53E-06 | 41 |
| 20 | GO:0030217 | T cell differentiation | 112/1431 | 31426/668746 | 1.76E-07 | 1.12E-05 | 112 |
| 21 | GO:0043405 | regulation of MAP kinase activity | 217/3240 | 31426/668746 | 2.16E-07 | 1.33E-05 | 217 |
| 22 | GO:0042254 | ribosome biogenesis | 48/465 | 31426/668746 | 4.17E-07 | 2.44E-05 | 48 |
| 23 | GO:0007369 | gastrulation | 74/861 | 31426/668746 | 7.05E-07 | 4.02E-05 | 74 |
| 24 | GO:0070661 | leukocyte proliferation | 112/1485 | 31426/668746 | 1.03E-06 | 5.63E-05 | 112 |
| 25 | GO:0048010 | vascular endothelial growth factor receptor signaling pathway | 21/136 | 31426/668746 | 1.67E-06 | 7.67E-05 | 21 |
| 26 | GO:0006260 | DNA replication | 107/1431 | 31426/668746 | 2.58E-06 | 0.000112 | 107 |
| 27 | GO:0071897 | DNA biosynthetic process | 48/496 | 31426/668746 | 2.58E-06 | 0.000112 | 48 |
| 28 | GO:0046651 | lymphocyte proliferation | 91/1176 | 31426/668746 | 3.61E-06 | 0.000151 | 91 |
| 29 | GO:0032943 | mononuclear cell proliferation | 91/1176 | 31426/668746 | 3.61E-06 | 0.000151 | 91 |
| 30 | GO:0030099 | myeloid cell differentiation | 191/2926 | 31426/668746 | 5.21E-06 | 0.000202 | 191 |
| 31 | GO:0007596 | blood coagulation | 112/1540 | 31426/668746 | 5.36E-06 | 0.000202 | 112 |
| 32 | GO:0050817 | coagulation | 112/1540 | 31426/668746 | 5.36E-06 | 0.000202 | 112 |
| 33 | GO:2000377 | regulation of reactive oxygen species metabolic process | 45/465 | 31426/668746 | 5.17E-06 | 0.000202 | 45 |
| 34 | GO:0071346 | cellular response to interferon-gamma | 37/351 | 31426/668746 | 5.05E-06 | 0.000202 | 37 |
| 35 | GO:0032963 | collagen metabolic process | 31/276 | 31426/668746 | 8E-06 | 0.000292 | 31 |
| 36 | GO:0050878 | regulation of body fluid levels | 225/3570 | 31426/668746 | 8.56E-06 | 0.000303 | 225 |
| 37 | GO:0050920 | regulation of chemotaxis | 65/780 | 31426/668746 | 8.54E-06 | 0.000303 | 65 |
| 38 | GO:0002697 | regulation of immune effector process | 98/1326 | 31426/668746 | 1.04E-05 | 0.000359 | 98 |
| 39 | GO:0048661 | positive regulation of smooth muscle cell proliferation | 40/406 | 31426/668746 | 1.09E-05 | 0.000371 | 40 |
| 40 | GO:0051480 | regulation of cytosolic calcium ion concentration | 164/2485 | 31426/668746 | 1.28E-05 | 0.000416 | 164 |
| 41 | GO:0007599 | hemostasis | 113/1596 | 31426/668746 | 1.56E-05 | 0.000499 | 113 |
| 42 | GO:0072593 | reactive oxygen species metabolic process | 59/703 | 31426/668746 | 1.75E-05 | 0.000545 | 59 |
| 43 | GO:0030168 | platelet activation | 52/595 | 31426/668746 | 1.82E-05 | 0.000559 | 52 |
| 44 | GO:0051052 | regulation of DNA metabolic process | 159/2415 | 31426/668746 | 1.93E-05 | 0.000583 | 159 |
| 45 | GO:0048872 | homeostasis of number of cells | 82/1081 | 31426/668746 | 2.11E-05 | 0.000617 | 82 |
| 46 | GO:0050918 | positive chemotaxis | 15/91 | 31426/668746 | 2.2E-05 | 0.000621 | 15 |
| 47 | GO:0051054 | positive regulation of DNA metabolic process | 79/1035 | 31426/668746 | 2.38E-05 | 0.000664 | 79 |
| 48 | GO:1903706 | regulation of hemopoiesis | 170/2628 | 31426/668746 | 2.62E-05 | 0.000689 | 170 |
| 49 | GO:0050729 | positive regulation of inflammatory response | 37/378 | 31426/668746 | 2.62E-05 | 0.000689 | 37 |
| 50 | GO:0071229 | cellular response to acid chemical | 115/1653 | 31426/668746 | 2.77E-05 | 0.000713 | 115 |
| 51 | GO:0033044 | regulation of chromosome organization | 125/1830 | 31426/668746 | 2.92E-05 | 0.000743 | 125 |
| 52 | GO:1902105 | regulation of leukocyte differentiation | 92/1275 | 31426/668746 | 4.54E-05 | 0.001118 | 92 |
| 53 | GO:0050707 | regulation of cytokine secretion | 40/435 | 31426/668746 | 5.22E-05 | 0.001257 | 40 |
| 54 | GO:0050900 | leukocyte migration | 188/3003 | 31426/668746 | 6.22E-05 | 0.001455 | 188 |
| 55 | GO:1904018 | positive regulation of vasculature development | 66/861 | 31426/668746 | 9.22E-05 | 0.002094 | 66 |
| 56 | GO:0001936 | regulation of endothelial cell proliferation | 30/300 | 31426/668746 | 9.77E-05 | 0.002197 | 30 |
| 57 | GO:0043122 | regulation of I-kappaB kinase/NF-kappaB signaling | 41/465 | 31426/668746 | 0.000107 | 0.002382 | 41 |
| 58 | GO:0001935 | endothelial cell proliferation | 37/406 | 31426/668746 | 0.000116 | 0.002565 | 37 |
| 59 | GO:0031570 | DNA integrity checkpoint | 49/595 | 31426/668746 | 0.000132 | 0.002792 | 49 |
| 60 | GO:0071219 | cellular response to molecule of bacterial origin | 78/1081 | 31426/668746 | 0.000161 | 0.00328 | 78 |
| 61 | GO:0071216 | cellular response to biotic stimulus | 78/1081 | 31426/668746 | 0.000161 | 0.00328 | 78 |
| 62 | GO:0071902 | positive regulation of protein serine/threonine kinase activity | 153/2415 | 31426/668746 | 0.000164 | 0.003299 | 153 |
| 63 | GO:0061448 | connective tissue development | 124/1891 | 31426/668746 | 0.000169 | 0.003384 | 124 |
| 64 | GO:0043200 | response to amino acid | 31/325 | 31426/668746 | 0.000178 | 0.003499 | 31 |
| 65 | GO:0038061 | NIK/NF-kappaB signaling | 31/325 | 31426/668746 | 0.000178 | 0.003499 | 31 |
| 66 | GO:0006953 | acute-phase response | 10/55 | 31426/668746 | 0.000219 | 0.004183 | 10 |
| 67 | GO:0000077 | DNA damage checkpoint | 38/435 | 31426/668746 | 0.000224 | 0.004186 | 38 |
| 68 | GO:0055074 | calcium ion homeostasis | 179/2926 | 31426/668746 | 0.000279 | 0.005018 | 179 |
| 69 | GO:0072503 | cellular divalent inorganic cation homeostasis | 179/2926 | 31426/668746 | 0.000279 | 0.005018 | 179 |
| 70 | GO:0001101 | response to acid chemical | 183/3003 | 31426/668746 | 0.00029 | 0.005177 | 183 |
| 71 | GO:2000573 | positive regulation of DNA biosynthetic process | 14/105 | 31426/668746 | 0.000414 | 0.006977 | 14 |
| 72 | GO:0060326 | cell chemotaxis | 139/2211 | 31426/668746 | 0.000425 | 0.007108 | 139 |
| 73 | GO:0045766 | positive regulation of angiogenesis | 49/630 | 31426/668746 | 0.000488 | 0.007983 | 49 |
| 74 | GO:0071887 | leukocyte apoptotic process | 28/300 | 31426/668746 | 0.000501 | 0.008025 | 28 |
| 75 | GO:0002761 | regulation of myeloid leukocyte differentiation | 28/300 | 31426/668746 | 0.000501 | 0.008025 | 28 |
| 76 | GO:0002822 | regulation of adaptive immune response based on somatic recombination of immune receptors built from immunoglobulin superfamily domains | 28/300 | 31426/668746 | 0.000501 | 0.008025 | 28 |
| 77 | GO:0071230 | cellular response to amino acid stimulus | 20/190 | 31426/668746 | 0.000688 | 0.010304 | 20 |
| 78 | GO:0002821 | positive regulation of adaptive immune response | 20/190 | 31426/668746 | 0.000688 | 0.010304 | 20 |
| 79 | GO:0010498 | proteasomal protein catabolic process | 67/946 | 31426/668746 | 0.000714 | 0.010579 | 67 |
| 80 | GO:0071222 | cellular response to lipopolysaccharide | 72/1035 | 31426/668746 | 0.00076 | 0.01118 | 72 |
| 81 | GO:0050663 | cytokine secretion | 40/496 | 31426/668746 | 0.000766 | 0.011198 | 40 |
| 82 | GO:0000075 | cell cycle checkpoint | 80/1176 | 31426/668746 | 0.000776 | 0.011274 | 80 |
| 83 | GO:0014065 | phosphatidylinositol 3-kinase signaling | 46/595 | 31426/668746 | 0.000802 | 0.011578 | 46 |
| 84 | GO:0001503 | ossification | 175/2926 | 31426/668746 | 0.00088 | 0.01248 | 175 |
| 85 | GO:0022617 | extracellular matrix disassembly | 10/66 | 31426/668746 | 0.000988 | 0.013695 | 10 |
| 86 | GO:0070663 | regulation of leukocyte proliferation | 56/780 | 31426/668746 | 0.001356 | 0.017724 | 56 |
| 87 | GO:0060759 | regulation of response to cytokine stimulus | 43/561 | 31426/668746 | 0.001363 | 0.017724 | 43 |
| 88 | GO:0048660 | regulation of smooth muscle cell proliferation | 63/903 | 31426/668746 | 0.001437 | 0.018477 | 63 |
| 89 | GO:0048659 | smooth muscle cell proliferation | 63/903 | 31426/668746 | 0.001437 | 0.018477 | 63 |
| 90 | GO:0018209 | peptidyl-serine modification | 81/1225 | 31426/668746 | 0.001589 | 0.019985 | 81 |
| 91 | GO:0001938 | positive regulation of endothelial cell proliferation | 19/190 | 31426/668746 | 0.001689 | 0.020698 | 19 |
| 92 | GO:0071900 | regulation of protein serine/threonine kinase activity | 365/6670 | 31426/668746 | 0.001828 | 0.022045 | 365 |
| 93 | GO:0002573 | myeloid leukocyte differentiation | 60/861 | 31426/668746 | 0.001875 | 0.022387 | 60 |
| 94 | GO:0033002 | muscle cell proliferation | 104/1653 | 31426/668746 | 0.001974 | 0.023447 | 104 |
| 95 | GO:1901990 | regulation of mitotic cell cycle phase transition | 164/2775 | 31426/668746 | 0.001989 | 0.023501 | 164 |
| 96 | GO:0051249 | regulation of lymphocyte activation | 134/2211 | 31426/668746 | 0.002027 | 0.02384 | 134 |
| 97 | GO:0043123 | positive regulation of I-kappaB kinase/NF-kappaB signaling | 20/210 | 31426/668746 | 0.00232 | 0.026741 | 20 |
| 98 | GO:0001933 | negative regulation of protein phosphorylation | 148/2485 | 31426/668746 | 0.002386 | 0.027369 | 148 |
| 99 | GO:0002703 | regulation of leukocyte mediated immunity | 29/351 | 31426/668746 | 0.002624 | 0.029518 | 29 |
| 100 | GO:0050867 | positive regulation of cell activation | 100/1596 | 31426/668746 | 0.002668 | 0.029852 | 100 |
| 101 | GO:0030595 | leukocyte chemotaxis | 69/1035 | 31426/668746 | 0.002731 | 0.029997 | 69 |
| 102 | GO:0016569 | covalent chromatin modification | 133/2211 | 31426/668746 | 0.002711 | 0.029997 | 133 |
| 103 | GO:0051656 | establishment of organelle localization | 74/1128 | 31426/668746 | 0.002953 | 0.03229 | 74 |
| 104 | GO:2000278 | regulation of DNA biosynthetic process | 17/171 | 31426/668746 | 0.003049 | 0.032732 | 17 |
| 105 | GO:0031663 | lipopolysaccharide-mediated signaling pathway | 12/105 | 31426/668746 | 0.003895 | 0.041052 | 12 |
| 106 | GO:0050863 | regulation of T cell activation | 87/1378 | 31426/668746 | 0.003913 | 0.041058 | 87 |
| 107 | GO:0002685 | regulation of leukocyte migration | 43/595 | 31426/668746 | 0.003992 | 0.04152 | 43 |
| 108 | GO:0032970 | regulation of actin filament-based process | 63/946 | 31426/668746 | 0.00411 | 0.042552 | 63 |
| 109 | GO:0051482 | positive regulation of cytosolic calcium ion concentration involved in phospholipase C-activating G protein-coupled signaling pathway | 7/45 | 31426/668746 | 0.004761 | 0.046812 | 7 |
| 110 | GO:0002696 | positive regulation of leukocyte activation | 95/1540 | 31426/668746 | 0.005077 | 0.049496 | 95 |
