# Supplementary material for: RNA-Seq-Based Breast Cancer Subtypes Classification Using Machine Learning Approaches
Source: Comput Intell Neurosci. 2020 Oct 29;2020:4737969. doi: 10.1155/2020/4737969 (PMC7644310; doi:10.1155/2020/4737969)
Supplement: Supplementary Materials — Figure S1: heatmap for Her2 and non Her2 groups. The left group 1 represents the Her2 group and the right group 2 denotes the non-Her2 group. Figure S2: heatmap for LumA and non-LumA groups. The left group 1 represents the LumA group and the right group 2 denotes the non-LumA group. Figure S3: heatmap for LumB and non-LumB groups. The left group 1 represents the LumB group and the right group 2 denotes the non-LumB group. Figure S4: heatmap for Normal-like and non-Normal-like groups. The left group 1 represents the Normal-like group and the right group 2 denotes the non-Normal-like group. S1 File: the detailed information of weighted DEGs for classification. S2 File: the detailed information of weighted DEGs for GO enrichment analysis. S3 File: the detailed enriched GO terms results for Basal-like subtype. S4 File: the detailed enriched GO terms results for Her2 subtype. S5 File: the detailed enriched GO terms results for LumA subtype. S6 File: the detailed enriched GO terms results for LumB subtype. S7 File: the detailed enriched GO terms results for Normal-like subtype. [file 4737969.f1.zip › supplementary materials/S4 File.docx]

**S4 File:** The detailed enriched GO terms results for Her2 subtype.

**Control group:**

| No. | ID | Description | GeneRatio | BgRatio | pvalue | p.adjust | count |
| --- | --- | --- | --- | --- | --- | --- | --- |
| 1 | GO:0048285 | organelle fission | 125/595 | 9396/115921 | 0 | 0 | 125 |
| 2 | GO:0007059 | chromosome segregation | 79/325 | 9396/115921 | 0 | 0 | 79 |
| 3 | GO:1901990 | regulation of mitotic cell cycle phase transition | 79/351 | 9396/115921 | 0 | 0 | 79 |
| 4 | GO:1901987 | regulation of cell cycle phase transition | 79/351 | 9396/115921 | 0 | 0 | 79 |
| 5 | GO:0033044 | regulation of chromosome organization | 75/253 | 9396/115921 | 0 | 0 | 75 |
| 6 | GO:0044843 | cell cycle G1/S phase transition | 50/190 | 9396/115921 | 5.8E-14 | 1.46E-11 | 50 |
| 7 | GO:0000082 | G1/S transition of mitotic cell cycle | 47/171 | 9396/115921 | 5.6E-14 | 1.46E-11 | 47 |
| 8 | GO:0000280 | nuclear division | 100/561 | 9396/115921 | 8.84E-14 | 1.95E-11 | 100 |
| 9 | GO:1901991 | negative regulation of mitotic cell cycle phase transition | 37/136 | 9396/115921 | 3.47E-11 | 6.13E-09 | 37 |
| 10 | GO:1901988 | negative regulation of cell cycle phase transition | 37/136 | 9396/115921 | 3.47E-11 | 6.13E-09 | 37 |
| 11 | GO:0098813 | nuclear chromosome segregation | 48/210 | 9396/115921 | 4.36E-11 | 6.99E-09 | 48 |
| 12 | GO:0010948 | negative regulation of cell cycle process | 60/300 | 9396/115921 | 6.66E-11 | 9.8E-09 | 60 |
| 13 | GO:0016570 | histone modification | 62/325 | 9396/115921 | 2.45E-10 | 3.09E-08 | 62 |
| 14 | GO:0016569 | covalent chromatin modification | 62/325 | 9396/115921 | 2.45E-10 | 3.09E-08 | 62 |
| 15 | GO:0051983 | regulation of chromosome segregation | 25/78 | 9396/115921 | 1.21E-09 | 1.37E-07 | 25 |
| 16 | GO:0000086 | G2/M transition of mitotic cell cycle | 37/153 | 9396/115921 | 1.32E-09 | 1.37E-07 | 37 |
| 17 | GO:0044839 | cell cycle G2/M phase transition | 37/153 | 9396/115921 | 1.32E-09 | 1.37E-07 | 37 |
| 18 | GO:0140014 | mitotic nuclear division | 77/465 | 9396/115921 | 1.89E-09 | 1.85E-07 | 77 |
| 19 | GO:0045930 | negative regulation of mitotic cell cycle | 49/253 | 9396/115921 | 1.04E-08 | 9.7E-07 | 49 |
| 20 | GO:0048708 | astrocyte differentiation | 12/21 | 9396/115921 | 1.17E-08 | 1.03E-06 | 12 |
| 21 | GO:1903046 | meiotic cell cycle process | 27/105 | 9396/115921 | 5.35E-08 | 4.5E-06 | 27 |
| 22 | GO:0009314 | response to radiation | 75/496 | 9396/115921 | 1.46E-07 | 1.17E-05 | 75 |
| 23 | GO:0071478 | cellular response to radiation | 20/66 | 9396/115921 | 1.52E-07 | 1.17E-05 | 20 |
| 24 | GO:0007088 | regulation of mitotic nuclear division | 49/276 | 9396/115921 | 1.77E-07 | 1.18E-05 | 49 |
| 25 | GO:0000070 | mitotic sister chromatid segregation | 33/153 | 9396/115921 | 1.87E-07 | 1.18E-05 | 33 |
| 26 | GO:0000819 | sister chromatid segregation | 33/153 | 9396/115921 | 1.87E-07 | 1.18E-05 | 33 |
| 27 | GO:0033045 | regulation of sister chromatid segregation | 18/55 | 9396/115921 | 1.72E-07 | 1.18E-05 | 18 |
| 28 | GO:0016579 | protein deubiquitination | 33/153 | 9396/115921 | 1.87E-07 | 1.18E-05 | 33 |
| 29 | GO:0010965 | regulation of mitotic sister chromatid separation | 16/45 | 9396/115921 | 2.25E-07 | 1.28E-05 | 16 |
| 30 | GO:1905818 | regulation of chromosome separation | 16/45 | 9396/115921 | 2.25E-07 | 1.28E-05 | 16 |
| 31 | GO:0051304 | chromosome separation | 16/45 | 9396/115921 | 2.25E-07 | 1.28E-05 | 16 |
| 32 | GO:1901655 | cellular response to ketone | 23/91 | 9396/115921 | 7.02E-07 | 3.87E-05 | 23 |
| 33 | GO:0051783 | regulation of nuclear division | 49/300 | 9396/115921 | 2.19E-06 | 0.000117 | 49 |
| 34 | GO:0010389 | regulation of G2/M transition of mitotic cell cycle | 22/91 | 9396/115921 | 2.71E-06 | 0.000137 | 22 |
| 35 | GO:1902749 | regulation of cell cycle G2/M phase transition | 22/91 | 9396/115921 | 2.71E-06 | 0.000137 | 22 |
| 36 | GO:0051302 | regulation of cell division | 20/78 | 9396/115921 | 2.87E-06 | 0.000141 | 20 |
| 37 | GO:0007093 | mitotic cell cycle checkpoint | 28/136 | 9396/115921 | 3.98E-06 | 0.00019 | 28 |
| 38 | GO:0000075 | cell cycle checkpoint | 30/153 | 9396/115921 | 5.22E-06 | 0.000236 | 30 |
| 39 | GO:0051321 | meiotic cell cycle | 30/153 | 9396/115921 | 5.22E-06 | 0.000236 | 30 |
| 40 | GO:0045931 | positive regulation of mitotic cell cycle | 32/171 | 9396/115921 | 7.2E-06 | 0.000318 | 32 |
| 41 | GO:2000045 | regulation of G1/S transition of mitotic cell cycle | 14/45 | 9396/115921 | 7.77E-06 | 0.000334 | 14 |
| 42 | GO:0031570 | DNA integrity checkpoint | 19/78 | 9396/115921 | 1.13E-05 | 0.000462 | 19 |
| 43 | GO:0140013 | meiotic nuclear division | 19/78 | 9396/115921 | 1.13E-05 | 0.000462 | 19 |
| 44 | GO:0000083 | regulation of transcription involved in G1/S transition of mitotic cell cycle | 9/21 | 9396/115921 | 1.79E-05 | 0.000703 | 9 |
| 45 | GO:0010972 | negative regulation of G2/M transition of mitotic cell cycle | 9/21 | 9396/115921 | 1.79E-05 | 0.000703 | 9 |
| 46 | GO:0032467 | positive regulation of cytokinesis | 5/6 | 9396/115921 | 1.96E-05 | 0.00075 | 5 |
| 47 | GO:0071383 | cellular response to steroid hormone stimulus | 43/276 | 9396/115921 | 2.86E-05 | 0.001075 | 43 |
| 48 | GO:1902750 | negative regulation of cell cycle G2/M phase transition | 10/28 | 9396/115921 | 4.07E-05 | 0.001467 | 10 |
| 49 | GO:0061982 | meiosis I cell cycle process | 10/28 | 9396/115921 | 4.07E-05 | 0.001467 | 10 |
| 50 | GO:0000076 | DNA replication checkpoint | 6/10 | 9396/115921 | 4.46E-05 | 0.001575 | 6 |
| 51 | GO:0048511 | rhythmic process | 50/351 | 9396/115921 | 7.52E-05 | 0.002604 | 50 |
| 52 | GO:0045787 | positive regulation of cell cycle | 79/630 | 9396/115921 | 8.27E-05 | 0.002808 | 79 |
| 53 | GO:0010212 | response to ionizing radiation | 21/105 | 9396/115921 | 9.44E-05 | 0.003144 | 21 |
| 54 | GO:0022412 | cellular process involved in reproduction in multicellular organism | 36/231 | 9396/115921 | 0.000121 | 0.003947 | 36 |
| 55 | GO:0006270 | DNA replication initiation | 8/21 | 9396/115921 | 0.000144 | 0.004622 | 8 |
| 56 | GO:1901992 | positive regulation of mitotic cell cycle phase transition | 15/66 | 9396/115921 | 0.000211 | 0.006541 | 15 |
| 57 | GO:1901989 | positive regulation of cell cycle phase transition | 15/66 | 9396/115921 | 0.000211 | 0.006541 | 15 |
| 58 | GO:0071453 | cellular response to oxygen levels | 35/231 | 9396/115921 | 0.000259 | 0.00787 | 35 |
| 59 | GO:0050673 | epithelial cell proliferation | 95/820 | 9396/115921 | 0.000312 | 0.009268 | 95 |
| 60 | GO:1903829 | positive regulation of cellular protein localization | 45/325 | 9396/115921 | 0.000315 | 0.009268 | 45 |
| 61 | GO:0071392 | cellular response to estradiol stimulus | 10/36 | 9396/115921 | 0.000432 | 0.012505 | 10 |
| 62 | GO:0006091 | generation of precursor metabolites and energy | 47/351 | 9396/115921 | 0.000502 | 0.014304 | 47 |
| 63 | GO:0090068 | positive regulation of cell cycle process | 44/325 | 9396/115921 | 0.000589 | 0.016498 | 44 |
| 64 | GO:0042176 | regulation of protein catabolic process | 25/153 | 9396/115921 | 0.000602 | 0.016612 | 25 |
| 65 | GO:0071479 | cellular response to ionizing radiation | 6/15 | 9396/115921 | 0.000745 | 0.020224 | 6 |
| 66 | GO:0051656 | establishment of organelle localization | 31/210 | 9396/115921 | 0.000873 | 0.02334 | 31 |
| 67 | GO:0048545 | response to steroid hormone | 104/946 | 9396/115921 | 0.001049 | 0.027633 | 104 |
| 68 | GO:0043010 | camera-type eye development | 40/300 | 9396/115921 | 0.001352 | 0.035101 | 40 |
| 69 | GO:0010639 | negative regulation of organelle organization | 26/171 | 9396/115921 | 0.001414 | 0.036163 | 26 |
| 70 | GO:0035690 | cellular response to drug | 66/561 | 9396/115921 | 0.001587 | 0.038462 | 66 |
| 71 | GO:0007292 | female gamete generation | 20/120 | 9396/115921 | 0.001575 | 0.038462 | 20 |
| 72 | GO:0150063 | visual system development | 45/351 | 9396/115921 | 0.001591 | 0.038462 | 45 |
| 73 | GO:0048880 | sensory system development | 45/351 | 9396/115921 | 0.001591 | 0.038462 | 45 |
| 74 | GO:0009755 | hormone-mediated signaling pathway | 30/210 | 9396/115921 | 0.001764 | 0.041508 | 30 |
| 75 | GO:0065004 | protein-DNA complex assembly | 30/210 | 9396/115921 | 0.001764 | 0.041508 | 30 |
| 76 | GO:0051781 | positive regulation of cell division | 9/36 | 9396/115921 | 0.001881 | 0.043675 | 9 |
| 77 | GO:0045137 | development of primary sexual characteristics | 56/465 | 9396/115921 | 0.002039 | 0.046728 | 56 |

**Experiment group:**

| No. | ID | Description | GeneRatio | BgRatio | pvalue | p.adjust | count |
| --- | --- | --- | --- | --- | --- | --- | --- |
| 1 | GO:0007204 | positive regulation of cytosolic calcium ion concentration | 71/741 | 5342/115921 | 8E-09 | 3.53E-06 | 71 |
| 2 | GO:0055074 | calcium ion homeostasis | 82/903 | 5342/115921 | 6.89E-09 | 3.53E-06 | 82 |
| 3 | GO:0072503 | cellular divalent inorganic cation homeostasis | 82/903 | 5342/115921 | 6.89E-09 | 3.53E-06 | 82 |
| 4 | GO:0050900 | leukocyte migration | 71/741 | 5342/115921 | 8E-09 | 3.53E-06 | 71 |
| 5 | GO:0042113 | B cell activation | 19/105 | 5342/115921 | 3.17E-07 | 0.000112 | 19 |
| 6 | GO:0030098 | lymphocyte differentiation | 47/465 | 5342/115921 | 5.48E-07 | 0.000161 | 47 |
| 7 | GO:0016055 | Wnt signaling pathway | 75/946 | 5342/115921 | 5.09E-06 | 0.000999 | 75 |
| 8 | GO:0198738 | cell-cell signaling by wnt | 75/946 | 5342/115921 | 5.09E-06 | 0.000999 | 75 |
| 9 | GO:0051188 | cofactor biosynthetic process | 20/136 | 5342/115921 | 4.62E-06 | 0.000999 | 20 |
| 10 | GO:0060326 | cell chemotaxis | 39/406 | 5342/115921 | 1.56E-05 | 0.002751 | 39 |
| 11 | GO:1903706 | regulation of hemopoiesis | 51/595 | 5342/115921 | 2.09E-05 | 0.003349 | 51 |
| 12 | GO:0006584 | catecholamine metabolic process | 5/10 | 5342/115921 | 4.3E-05 | 0.006326 | 5 |
| 13 | GO:0001936 | regulation of endothelial cell proliferation | 15/105 | 5342/115921 | 9.65E-05 | 0.013099 | 15 |
| 14 | GO:0009166 | nucleotide catabolic process | 10/55 | 5342/115921 | 0.000186 | 0.023502 | 10 |
| 15 | GO:0002703 | regulation of leukocyte mediated immunity | 11/66 | 5342/115921 | 0.000202 | 0.023822 | 11 |
| 16 | GO:1901617 | organic hydroxy compound biosynthetic process | 23/231 | 5342/115921 | 0.000477 | 0.044349 | 23 |
| 17 | GO:1903708 | positive regulation of hemopoiesis | 15/120 | 5342/115921 | 0.00043 | 0.044349 | 15 |
| 18 | GO:0001935 | endothelial cell proliferation | 15/120 | 5342/115921 | 0.00043 | 0.044349 | 15 |
| 19 | GO:0006732 | coenzyme metabolic process | 23/231 | 5342/115921 | 0.000477 | 0.044349 | 23 |

**Common:**

NA
