# Supplementary material for: RNA-Seq-Based Breast Cancer Subtypes Classification Using Machine Learning Approaches
Source: Comput Intell Neurosci. 2020 Oct 29;2020:4737969. doi: 10.1155/2020/4737969 (PMC7644310; doi:10.1155/2020/4737969)
Supplement: Supplementary Materials — Figure S1: heatmap for Her2 and non Her2 groups. The left group 1 represents the Her2 group and the right group 2 denotes the non-Her2 group. Figure S2: heatmap for LumA and non-LumA groups. The left group 1 represents the LumA group and the right group 2 denotes the non-LumA group. Figure S3: heatmap for LumB and non-LumB groups. The left group 1 represents the LumB group and the right group 2 denotes the non-LumB group. Figure S4: heatmap for Normal-like and non-Normal-like groups. The left group 1 represents the Normal-like group and the right group 2 denotes the non-Normal-like group. S1 File: the detailed information of weighted DEGs for classification. S2 File: the detailed information of weighted DEGs for GO enrichment analysis. S3 File: the detailed enriched GO terms results for Basal-like subtype. S4 File: the detailed enriched GO terms results for Her2 subtype. S5 File: the detailed enriched GO terms results for LumA subtype. S6 File: the detailed enriched GO terms results for LumB subtype. S7 File: the detailed enriched GO terms results for Normal-like subtype. [file 4737969.f1.zip › supplementary materials/S5 File.docx]

**S5 File:** The detailed enriched GO terms results for LumA subtype.

**Control group:**

| No. | ID | Description | GeneRatio | BgRatio | pvalue | p.adjust | count |
| --- | --- | --- | --- | --- | --- | --- | --- |
| 1 | GO:0048732 | gland development | 329/2016 | 21214/237016 | 0 | 0 | 329 |
| 2 | GO:0060562 | epithelial tube morphogenesis | 202/990 | 21214/237016 | 0 | 0 | 202 |
| 3 | GO:0006352 | DNA-templated transcription, initiation | 72/276 | 21214/237016 | 0 | 0 | 72 |
| 4 | GO:0002009 | morphogenesis of an epithelium | 264/1770 | 21214/237016 | 2.22E-16 | 1.03E-13 | 264 |
| 5 | GO:0022612 | gland morphogenesis | 62/231 | 21214/237016 | 2.33E-15 | 8.69E-13 | 62 |
| 6 | GO:0061180 | mammary gland epithelium development | 36/91 | 21214/237016 | 3.55E-15 | 1.1E-12 | 36 |
| 7 | GO:0007548 | sex differentiation | 128/703 | 21214/237016 | 1.22E-14 | 3.25E-12 | 128 |
| 8 | GO:0030522 | intracellular receptor signaling pathway | 103/528 | 21214/237016 | 5.28E-14 | 1.23E-11 | 103 |
| 9 | GO:0002064 | epithelial cell development | 69/300 | 21214/237016 | 2.62E-13 | 5.42E-11 | 69 |
| 10 | GO:0071222 | cellular response to lipopolysaccharide | 60/253 | 21214/237016 | 2.26E-12 | 3.5E-10 | 60 |
| 11 | GO:0071219 | cellular response to molecule of bacterial origin | 60/253 | 21214/237016 | 2.26E-12 | 3.5E-10 | 60 |
| 12 | GO:0071216 | cellular response to biotic stimulus | 60/253 | 21214/237016 | 2.26E-12 | 3.5E-10 | 60 |
| 13 | GO:0045137 | development of primary sexual characteristics | 92/496 | 21214/237016 | 2.03E-11 | 2.91E-09 | 92 |
| 14 | GO:0071383 | cellular response to steroid hormone stimulus | 107/630 | 21214/237016 | 1.23E-10 | 1.63E-08 | 107 |
| 15 | GO:0008406 | gonad development | 84/465 | 21214/237016 | 5.54E-10 | 6.88E-08 | 84 |
| 16 | GO:0043401 | steroid hormone mediated signaling pathway | 67/351 | 21214/237016 | 2.93E-09 | 3.21E-07 | 67 |
| 17 | GO:0001541 | ovarian follicle development | 11/15 | 21214/237016 | 2.86E-09 | 3.21E-07 | 11 |
| 18 | GO:0001655 | urogenital system development | 154/1081 | 21214/237016 | 8.34E-09 | 8.63E-07 | 154 |
| 19 | GO:0048863 | stem cell differentiation | 52/253 | 21214/237016 | 1.28E-08 | 1.26E-06 | 52 |
| 20 | GO:0048608 | reproductive structure development | 204/1540 | 21214/237016 | 1.5E-08 | 1.33E-06 | 204 |
| 21 | GO:0061458 | reproductive system development | 204/1540 | 21214/237016 | 1.5E-08 | 1.33E-06 | 204 |
| 22 | GO:0051052 | regulation of DNA metabolic process | 131/903 | 21214/237016 | 3.54E-08 | 3E-06 | 131 |
| 23 | GO:0045165 | cell fate commitment | 90/561 | 21214/237016 | 5.32E-08 | 4.31E-06 | 90 |
| 24 | GO:0007409 | axonogenesis | 63/351 | 21214/237016 | 9.1E-08 | 7.06E-06 | 63 |
| 25 | GO:1902105 | regulation of leukocyte differentiation | 83/528 | 21214/237016 | 4.03E-07 | 2.89E-05 | 83 |
| 26 | GO:0006338 | chromatin remodeling | 61/351 | 21214/237016 | 4.53E-07 | 3.13E-05 | 61 |
| 27 | GO:0060443 | mammary gland morphogenesis | 20/66 | 21214/237016 | 7.48E-07 | 4.65E-05 | 20 |
| 28 | GO:0051090 | regulation of DNA-binding transcription factor activity | 86/561 | 21214/237016 | 7.4E-07 | 4.65E-05 | 86 |
| 29 | GO:0070301 | cellular response to hydrogen peroxide | 18/55 | 21214/237016 | 7.48E-07 | 4.65E-05 | 18 |
| 30 | GO:0009755 | hormone-mediated signaling pathway | 78/496 | 21214/237016 | 8.64E-07 | 5.03E-05 | 78 |
| 31 | GO:0044786 | cell cycle DNA replication | 22/78 | 21214/237016 | 8.42E-07 | 5.03E-05 | 22 |
| 32 | GO:0060603 | mammary gland duct morphogenesis | 14/36 | 21214/237016 | 1.19E-06 | 6.7E-05 | 14 |
| 33 | GO:0046660 | female sex differentiation | 44/231 | 21214/237016 | 1.43E-06 | 7.83E-05 | 44 |
| 34 | GO:0001763 | morphogenesis of a branching structure | 81/528 | 21214/237016 | 1.47E-06 | 7.84E-05 | 81 |
| 35 | GO:0007411 | axon guidance | 33/153 | 21214/237016 | 1.74E-06 | 8.74E-05 | 33 |
| 36 | GO:0097485 | neuron projection guidance | 33/153 | 21214/237016 | 1.74E-06 | 8.74E-05 | 33 |
| 37 | GO:1902275 | regulation of chromatin organization | 28/120 | 21214/237016 | 2.03E-06 | 9.94E-05 | 28 |
| 38 | GO:0034502 | protein localization to chromosome | 30/136 | 21214/237016 | 3.09E-06 | 0.000147 | 30 |
| 39 | GO:0046545 | development of primary female sexual characteristics | 25/105 | 21214/237016 | 4.75E-06 | 0.000216 | 25 |
| 40 | GO:0002761 | regulation of myeloid leukocyte differentiation | 25/105 | 21214/237016 | 4.75E-06 | 0.000216 | 25 |
| 41 | GO:0071236 | cellular response to antibiotic | 32/153 | 21214/237016 | 4.91E-06 | 0.000218 | 32 |
| 42 | GO:0048754 | branching morphogenesis of an epithelial tube | 64/406 | 21214/237016 | 7.15E-06 | 0.00031 | 64 |
| 43 | GO:0071887 | leukocyte apoptotic process | 34/171 | 21214/237016 | 8.11E-06 | 0.000343 | 34 |
| 44 | GO:0006260 | DNA replication | 125/946 | 21214/237016 | 8.88E-06 | 0.000368 | 125 |
| 45 | GO:0046661 | male sex differentiation | 50/300 | 21214/237016 | 1.5E-05 | 0.000583 | 50 |
| 46 | GO:0060444 | branching involved in mammary gland duct morphogenesis | 11/28 | 21214/237016 | 1.49E-05 | 0.000583 | 11 |
| 47 | GO:0031663 | lipopolysaccharide-mediated signaling pathway | 8/15 | 21214/237016 | 1.48E-05 | 0.000583 | 8 |
| 48 | GO:0042093 | T-helper cell differentiation | 16/55 | 21214/237016 | 1.67E-05 | 0.000609 | 16 |
| 49 | GO:0002287 | alpha-beta T cell activation involved in immune response | 16/55 | 21214/237016 | 1.67E-05 | 0.000609 | 16 |
| 50 | GO:0002292 | T cell differentiation involved in immune response | 16/55 | 21214/237016 | 1.67E-05 | 0.000609 | 16 |
| 51 | GO:0071375 | cellular response to peptide hormone stimulus | 88/630 | 21214/237016 | 2.34E-05 | 0.000821 | 88 |
| 52 | GO:0034614 | cellular response to reactive oxygen species | 38/210 | 21214/237016 | 2.39E-05 | 0.000825 | 38 |
| 53 | GO:0051961 | negative regulation of nervous system development | 46/276 | 21214/237016 | 3.2E-05 | 0.001065 | 46 |
| 54 | GO:0002699 | positive regulation of immune effector process | 30/153 | 21214/237016 | 3.52E-05 | 0.00115 | 30 |
| 55 | GO:0035065 | regulation of histone acetylation | 9/21 | 21214/237016 | 3.97E-05 | 0.001276 | 9 |
| 56 | GO:2000756 | regulation of peptidyl-lysine acetylation | 12/36 | 21214/237016 | 4.23E-05 | 0.001313 | 12 |
| 57 | GO:1901983 | regulation of protein acetylation | 12/36 | 21214/237016 | 4.23E-05 | 0.001313 | 12 |
| 58 | GO:0070227 | lymphocyte apoptotic process | 17/66 | 21214/237016 | 5.21E-05 | 0.001566 | 17 |
| 59 | GO:0070664 | negative regulation of leukocyte proliferation | 17/66 | 21214/237016 | 5.21E-05 | 0.001566 | 17 |
| 60 | GO:2000106 | regulation of leukocyte apoptotic process | 25/120 | 21214/237016 | 5.41E-05 | 0.001601 | 25 |
| 61 | GO:0000082 | G1/S transition of mitotic cell cycle | 116/903 | 21214/237016 | 6.06E-05 | 0.001764 | 116 |
| 62 | GO:0048545 | response to steroid hormone | 219/1891 | 21214/237016 | 6.17E-05 | 0.001768 | 219 |
| 63 | GO:0006275 | regulation of DNA replication | 27/136 | 21214/237016 | 6.74E-05 | 0.001874 | 27 |
| 64 | GO:0006171 | cAMP biosynthetic process | 6/10 | 21214/237016 | 7.85E-05 | 0.002119 | 6 |
| 65 | GO:0035162 | embryonic hemopoiesis | 6/10 | 21214/237016 | 7.85E-05 | 0.002119 | 6 |
| 66 | GO:1902107 | positive regulation of leukocyte differentiation | 29/153 | 21214/237016 | 8.88E-05 | 0.002364 | 29 |
| 67 | GO:1903708 | positive regulation of hemopoiesis | 39/231 | 21214/237016 | 9.15E-05 | 0.002401 | 39 |
| 68 | GO:0048538 | thymus development | 10/28 | 21214/237016 | 9.49E-05 | 0.002421 | 10 |
| 69 | GO:0060688 | regulation of morphogenesis of a branching structure | 10/28 | 21214/237016 | 9.49E-05 | 0.002421 | 10 |
| 70 | GO:0001558 | regulation of cell growth | 63/435 | 21214/237016 | 0.000111 | 0.002748 | 63 |
| 71 | GO:0035690 | cellular response to drug | 110/861 | 21214/237016 | 0.000114 | 0.002791 | 110 |
| 72 | GO:0044843 | cell cycle G1/S phase transition | 119/946 | 21214/237016 | 0.000118 | 0.002858 | 119 |
| 73 | GO:0001657 | ureteric bud development | 24/120 | 21214/237016 | 0.000147 | 0.003475 | 24 |
| 74 | GO:0072163 | mesonephric epithelium development | 24/120 | 21214/237016 | 0.000147 | 0.003475 | 24 |
| 75 | GO:0070229 | negative regulation of lymphocyte apoptotic process | 7/15 | 21214/237016 | 0.000155 | 0.003601 | 7 |
| 76 | GO:0001701 | in utero embryonic development | 59/406 | 21214/237016 | 0.000162 | 0.003737 | 59 |
| 77 | GO:0048568 | embryonic organ development | 88/666 | 21214/237016 | 0.000168 | 0.003828 | 88 |
| 78 | GO:0070228 | regulation of lymphocyte apoptotic process | 11/36 | 21214/237016 | 0.000212 | 0.004708 | 11 |
| 79 | GO:0071900 | regulation of protein serine/threonine kinase activity | 317/2926 | 21214/237016 | 0.000264 | 0.005578 | 317 |
| 80 | GO:0010721 | negative regulation of cell development | 55/378 | 21214/237016 | 0.000255 | 0.005578 | 55 |
| 81 | GO:0032649 | regulation of interferon-gamma production | 14/55 | 21214/237016 | 0.000266 | 0.005578 | 14 |
| 82 | GO:0032609 | interferon-gamma production | 14/55 | 21214/237016 | 0.000266 | 0.005578 | 14 |
| 83 | GO:0006997 | nucleus organization | 14/55 | 21214/237016 | 0.000266 | 0.005578 | 14 |
| 84 | GO:0008156 | negative regulation of DNA replication | 8/21 | 21214/237016 | 0.000287 | 0.005945 | 8 |
| 85 | GO:0061138 | morphogenesis of a branching epithelium | 68/496 | 21214/237016 | 0.000309 | 0.006329 | 68 |
| 86 | GO:0007219 | Notch signaling pathway | 32/190 | 21214/237016 | 0.000385 | 0.007639 | 32 |
| 87 | GO:0060348 | bone development | 32/190 | 21214/237016 | 0.000385 | 0.007639 | 32 |
| 88 | GO:0031056 | regulation of histone modification | 19/91 | 21214/237016 | 0.000392 | 0.00769 | 19 |
| 89 | GO:0070663 | regulation of leukocyte proliferation | 54/378 | 21214/237016 | 0.000453 | 0.008692 | 54 |
| 90 | GO:0048562 | embryonic organ morphogenesis | 29/171 | 21214/237016 | 0.000626 | 0.011776 | 29 |
| 91 | GO:0023061 | signal release | 110/903 | 21214/237016 | 0.000666 | 0.012402 | 110 |
| 92 | GO:0034599 | cellular response to oxidative stress | 66/496 | 21214/237016 | 0.000832 | 0.015188 | 66 |
| 93 | GO:0097094 | craniofacial suture morphogenesis | 4/6 | 21214/237016 | 0.00083 | 0.015188 | 4 |
| 94 | GO:1901655 | cellular response to ketone | 20/105 | 21214/237016 | 0.000963 | 0.016606 | 20 |
| 95 | GO:2000107 | negative regulation of leukocyte apoptotic process | 13/55 | 21214/237016 | 0.000934 | 0.016606 | 13 |
| 96 | GO:0046854 | phosphatidylinositol phosphorylation | 20/105 | 21214/237016 | 0.000963 | 0.016606 | 20 |
| 97 | GO:0046638 | positive regulation of alpha-beta T cell differentiation | 10/36 | 21214/237016 | 0.000944 | 0.016606 | 10 |
| 98 | GO:0046834 | lipid phosphorylation | 20/105 | 21214/237016 | 0.000963 | 0.016606 | 20 |
| 99 | GO:0002285 | lymphocyte activation involved in immune response | 22/120 | 21214/237016 | 0.000943 | 0.016606 | 22 |
| 100 | GO:2000648 | positive regulation of stem cell proliferation | 5/10 | 21214/237016 | 0.000984 | 0.016817 | 5 |
| 101 | GO:0043583 | ear development | 26/153 | 21214/237016 | 0.001123 | 0.019012 | 26 |
| 102 | GO:1903429 | regulation of cell maturation | 6/15 | 21214/237016 | 0.001261 | 0.02097 | 6 |
| 103 | GO:0045576 | mast cell activation | 6/15 | 21214/237016 | 0.001261 | 0.02097 | 6 |
| 104 | GO:0030278 | regulation of ossification | 52/378 | 21214/237016 | 0.001339 | 0.021884 | 52 |
| 105 | GO:0045621 | positive regulation of lymphocyte differentiation | 16/78 | 21214/237016 | 0.00133 | 0.021884 | 16 |
| 106 | GO:0002573 | myeloid leukocyte differentiation | 46/325 | 21214/237016 | 0.001371 | 0.022204 | 46 |
| 107 | GO:0009615 | response to virus | 55/406 | 21214/237016 | 0.001418 | 0.022768 | 55 |
| 108 | GO:0042110 | T cell activation | 121/1035 | 21214/237016 | 0.001679 | 0.026736 | 121 |
| 109 | GO:0038034 | signal transduction in absence of ligand | 7/21 | 21214/237016 | 0.001726 | 0.027255 | 7 |
| 110 | GO:0030850 | prostate gland development | 14/66 | 21214/237016 | 0.00186 | 0.028869 | 14 |
| 111 | GO:1901796 | regulation of signal transduction by p53 class mediator | 14/66 | 21214/237016 | 0.00186 | 0.028869 | 14 |
| 112 | GO:0046546 | development of primary male sexual characteristics | 37/253 | 21214/237016 | 0.002149 | 0.03255 | 37 |
| 113 | GO:0048565 | digestive tract development | 32/210 | 21214/237016 | 0.00214 | 0.03255 | 32 |
| 114 | GO:0009416 | response to light stimulus | 37/253 | 21214/237016 | 0.002149 | 0.03255 | 37 |
| 115 | GO:0035148 | tube formation | 23/136 | 21214/237016 | 0.002233 | 0.033542 | 23 |
| 116 | GO:0001838 | embryonic epithelial tube formation | 19/105 | 21214/237016 | 0.002364 | 0.03524 | 19 |
| 117 | GO:0045684 | positive regulation of epidermis development | 8/28 | 21214/237016 | 0.002485 | 0.036735 | 8 |
| 118 | GO:0016570 | histone modification | 57/435 | 21214/237016 | 0.002512 | 0.036849 | 57 |
| 119 | GO:0007569 | cell aging | 17/91 | 21214/237016 | 0.002763 | 0.039596 | 17 |
| 120 | GO:0009411 | response to UV | 17/91 | 21214/237016 | 0.002763 | 0.039596 | 17 |
| 121 | GO:0051250 | negative regulation of lymphocyte activation | 17/91 | 21214/237016 | 0.002763 | 0.039596 | 17 |
| 122 | GO:0097191 | extrinsic apoptotic signaling pathway | 34/231 | 21214/237016 | 0.002842 | 0.040112 | 34 |
| 123 | GO:0018205 | peptidyl-lysine modification | 34/231 | 21214/237016 | 0.002842 | 0.040112 | 34 |
| 124 | GO:0046879 | hormone secretion | 81/666 | 21214/237016 | 0.003199 | 0.04414 | 81 |
| 125 | GO:0009914 | hormone transport | 81/666 | 21214/237016 | 0.003199 | 0.04414 | 81 |
| 126 | GO:0006310 | DNA recombination | 29/190 | 21214/237016 | 0.003262 | 0.044689 | 29 |
| 127 | GO:0060135 | maternal process involved in female pregnancy | 9/36 | 21214/237016 | 0.003702 | 0.049979 | 9 |
| 128 | GO:0002824 | positive regulation of adaptive immune response based on somatic recombination of immune receptors built from immunoglobulin superfamily domains | 9/36 | 21214/237016 | 0.003702 | 0.049979 | 9 |

**Experiment group:**

| NO. | ID | Description | GeneRatio | BgRatio | pvalue | p.adjust | count |
| --- | --- | --- | --- | --- | --- | --- | --- |
| 1 | GO:0001101 | response to acid chemical | 124/1431 | 6230/237016 | 0 | 0 | 124 |
| 2 | GO:0050673 | epithelial cell proliferation | 82/820 | 6230/237016 | 0 | 0 | 82 |
| 3 | GO:0050678 | regulation of epithelial cell proliferation | 62/561 | 6230/237016 | 0 | 0 | 62 |
| 4 | GO:0048871 | multicellular organismal homeostasis | 91/1378 | 6230/237016 | 4.22E-15 | 1.96E-12 | 91 |
| 5 | GO:0019216 | regulation of lipid metabolic process | 81/1225 | 6230/237016 | 1.21E-13 | 3.75E-11 | 81 |
| 6 | GO:0061448 | connective tissue development | 58/741 | 6230/237016 | 4.31E-13 | 1.15E-10 | 58 |
| 7 | GO:0070372 | regulation of ERK1 and ERK2 cascade | 73/1081 | 6230/237016 | 6.97E-13 | 1.53E-10 | 73 |
| 8 | GO:0070302 | regulation of stress-activated protein kinase signaling cascade | 26/171 | 6230/237016 | 7.4E-13 | 1.53E-10 | 26 |
| 9 | GO:0045785 | positive regulation of cell adhesion | 76/1176 | 6230/237016 | 2.04E-12 | 3.8E-10 | 76 |
| 10 | GO:0070371 | ERK1 and ERK2 cascade | 75/1176 | 6230/237016 | 5.3E-12 | 8.97E-10 | 75 |
| 11 | GO:0031589 | cell-substrate adhesion | 41/496 | 6230/237016 | 2.08E-10 | 3.22E-08 | 41 |
| 12 | GO:0010876 | lipid localization | 37/435 | 6230/237016 | 7.01E-10 | 1E-07 | 37 |
| 13 | GO:0032963 | collagen metabolic process | 24/210 | 6230/237016 | 2.25E-09 | 2.8E-07 | 24 |
| 14 | GO:0001503 | ossification | 72/1275 | 6230/237016 | 2.65E-09 | 3.09E-07 | 72 |
| 15 | GO:0043200 | response to amino acid | 21/171 | 6230/237016 | 6.1E-09 | 6.68E-07 | 21 |
| 16 | GO:1901652 | response to peptide | 83/1596 | 6230/237016 | 7.42E-09 | 7.68E-07 | 83 |
| 17 | GO:0031098 | stress-activated protein kinase signaling cascade | 28/300 | 6230/237016 | 1.06E-08 | 1.04E-06 | 28 |
| 18 | GO:0006979 | response to oxidative stress | 70/1275 | 6230/237016 | 1.33E-08 | 1.24E-06 | 70 |
| 19 | GO:0006641 | triglyceride metabolic process | 13/66 | 6230/237016 | 1.57E-08 | 1.27E-06 | 13 |
| 20 | GO:0006638 | neutral lipid metabolic process | 13/66 | 6230/237016 | 1.57E-08 | 1.27E-06 | 13 |
| 21 | GO:1903034 | regulation of response to wounding | 14/78 | 6230/237016 | 1.56E-08 | 1.27E-06 | 14 |
| 22 | GO:0006631 | fatty acid metabolic process | 34/435 | 6230/237016 | 2.72E-08 | 2.03E-06 | 34 |
| 23 | GO:0055074 | calcium ion homeostasis | 65/1176 | 6230/237016 | 3.34E-08 | 2.3E-06 | 65 |
| 24 | GO:0072503 | cellular divalent inorganic cation homeostasis | 65/1176 | 6230/237016 | 3.34E-08 | 2.3E-06 | 65 |
| 25 | GO:0060326 | cell chemotaxis | 39/561 | 6230/237016 | 6.42E-08 | 4.27E-06 | 39 |
| 26 | GO:0097756 | negative regulation of blood vessel diameter | 15/105 | 6230/237016 | 1.16E-07 | 7.22E-06 | 15 |
| 27 | GO:0014066 | regulation of phosphatidylinositol 3-kinase signaling | 18/153 | 6230/237016 | 1.39E-07 | 8.37E-06 | 18 |
| 28 | GO:0032102 | negative regulation of response to external stimulus | 26/300 | 6230/237016 | 1.53E-07 | 8.88E-06 | 26 |
| 29 | GO:0052547 | regulation of peptidase activity | 49/820 | 6230/237016 | 1.61E-07 | 9.09E-06 | 49 |
| 30 | GO:0014065 | phosphatidylinositol 3-kinase signaling | 21/210 | 6230/237016 | 2.19E-07 | 1.2E-05 | 21 |
| 31 | GO:0018108 | peptidyl-tyrosine phosphorylation | 51/903 | 6230/237016 | 4.96E-07 | 2.57E-05 | 51 |
| 32 | GO:0018212 | peptidyl-tyrosine modification | 51/903 | 6230/237016 | 4.96E-07 | 2.57E-05 | 51 |
| 33 | GO:0072593 | reactive oxygen species metabolic process | 31/435 | 6230/237016 | 8.02E-07 | 4.04E-05 | 31 |
| 34 | GO:0043062 | extracellular structure organization | 80/1711 | 6230/237016 | 9.74E-07 | 4.77E-05 | 80 |
| 35 | GO:0019915 | lipid storage | 10/55 | 6230/237016 | 1.55E-06 | 7.22E-05 | 10 |
| 36 | GO:0030595 | leukocyte chemotaxis | 24/300 | 6230/237016 | 1.86E-06 | 8.25E-05 | 24 |
| 37 | GO:0006869 | lipid transport | 24/300 | 6230/237016 | 1.86E-06 | 8.25E-05 | 24 |
| 38 | GO:0051216 | cartilage development | 31/465 | 6230/237016 | 3.19E-06 | 0.000138 | 31 |
| 39 | GO:0050680 | negative regulation of epithelial cell proliferation | 15/136 | 6230/237016 | 3.37E-06 | 0.00014 | 15 |
| 40 | GO:0050679 | positive regulation of epithelial cell proliferation | 15/136 | 6230/237016 | 3.37E-06 | 0.00014 | 15 |
| 41 | GO:0010810 | regulation of cell-substrate adhesion | 19/210 | 6230/237016 | 3.62E-06 | 0.000146 | 19 |
| 42 | GO:1901654 | response to ketone | 39/666 | 6230/237016 | 4.42E-06 | 0.000174 | 39 |
| 43 | GO:0051403 | stress-activated MAPK cascade | 21/253 | 6230/237016 | 4.49E-06 | 0.000174 | 21 |
| 44 | GO:0048771 | tissue remodeling | 12/91 | 6230/237016 | 4.96E-06 | 0.000188 | 12 |
| 45 | GO:0032612 | interleukin-1 production | 11/78 | 6230/237016 | 6.31E-06 | 0.000235 | 11 |
| 46 | GO:0043010 | camera-type eye development | 29/435 | 6230/237016 | 6.49E-06 | 0.000237 | 29 |
| 47 | GO:0033002 | muscle cell proliferation | 34/561 | 6230/237016 | 8.57E-06 | 0.000307 | 34 |
| 48 | GO:0048660 | regulation of smooth muscle cell proliferation | 26/378 | 6230/237016 | 1.1E-05 | 0.000367 | 26 |
| 49 | GO:0048659 | smooth muscle cell proliferation | 26/378 | 6230/237016 | 1.1E-05 | 0.000367 | 26 |
| 50 | GO:0051896 | regulation of protein kinase B signaling | 26/378 | 6230/237016 | 1.1E-05 | 0.000367 | 26 |
| 51 | GO:0051235 | maintenance of location | 26/378 | 6230/237016 | 1.1E-05 | 0.000367 | 26 |
| 52 | GO:1903037 | regulation of leukocyte cell-cell adhesion | 35/595 | 6230/237016 | 1.2E-05 | 0.00038 | 35 |
| 53 | GO:0007159 | leukocyte cell-cell adhesion | 35/595 | 6230/237016 | 1.2E-05 | 0.00038 | 35 |
| 54 | GO:0030324 | lung development | 18/210 | 6230/237016 | 1.35E-05 | 0.0004 | 18 |
| 55 | GO:0030323 | respiratory tube development | 18/210 | 6230/237016 | 1.35E-05 | 0.0004 | 18 |
| 56 | GO:0010952 | positive regulation of peptidase activity | 18/210 | 6230/237016 | 1.35E-05 | 0.0004 | 18 |
| 57 | GO:0009612 | response to mechanical stimulus | 16/171 | 6230/237016 | 1.35E-05 | 0.0004 | 16 |
| 58 | GO:0035296 | regulation of tube diameter | 19/231 | 6230/237016 | 1.42E-05 | 0.000407 | 19 |
| 59 | GO:0097746 | regulation of blood vessel diameter | 19/231 | 6230/237016 | 1.42E-05 | 0.000407 | 19 |
| 60 | GO:0150063 | visual system development | 32/528 | 6230/237016 | 1.55E-05 | 0.00043 | 32 |
| 61 | GO:0048880 | sensory system development | 32/528 | 6230/237016 | 1.55E-05 | 0.00043 | 32 |
| 62 | GO:0050730 | regulation of peptidyl-tyrosine phosphorylation | 29/465 | 6230/237016 | 2.24E-05 | 0.000615 | 29 |
| 63 | GO:0001667 | ameboidal-type cell migration | 37/666 | 6230/237016 | 2.38E-05 | 0.000643 | 37 |
| 64 | GO:0030900 | forebrain development | 57/1225 | 6230/237016 | 3.65E-05 | 0.000964 | 57 |
| 65 | GO:0043491 | protein kinase B signaling | 26/406 | 6230/237016 | 3.73E-05 | 0.000964 | 26 |
| 66 | GO:0019217 | regulation of fatty acid metabolic process | 7/36 | 6230/237016 | 3.69E-05 | 0.000964 | 7 |
| 67 | GO:0019229 | regulation of vasoconstriction | 10/78 | 6230/237016 | 3.85E-05 | 0.000984 | 10 |
| 68 | GO:0007204 | positive regulation of cytosolic calcium ion concentration | 45/903 | 6230/237016 | 4.84E-05 | 0.00117 | 45 |
| 69 | GO:0016055 | Wnt signaling pathway | 45/903 | 6230/237016 | 4.84E-05 | 0.00117 | 45 |
| 70 | GO:0198738 | cell-cell signaling by wnt | 45/903 | 6230/237016 | 4.84E-05 | 0.00117 | 45 |
| 71 | GO:0043409 | negative regulation of MAPK cascade | 17/210 | 6230/237016 | 4.79E-05 | 0.00117 | 17 |
| 72 | GO:1904018 | positive regulation of vasculature development | 19/253 | 6230/237016 | 4.97E-05 | 0.001171 | 19 |
| 73 | GO:0016042 | lipid catabolic process | 19/253 | 6230/237016 | 4.97E-05 | 0.001171 | 19 |
| 74 | GO:0007160 | cell-matrix adhesion | 15/171 | 6230/237016 | 5.26E-05 | 0.001224 | 15 |
| 75 | GO:0043405 | regulation of MAP kinase activity | 58/1275 | 6230/237016 | 5.79E-05 | 0.001331 | 58 |
| 76 | GO:0003018 | vascular process in circulatory system | 22/325 | 6230/237016 | 6.35E-05 | 0.001442 | 22 |
| 77 | GO:0003012 | muscle system process | 43/861 | 6230/237016 | 6.67E-05 | 0.001498 | 43 |
| 78 | GO:0051592 | response to calcium ion | 12/120 | 6230/237016 | 8.23E-05 | 0.001824 | 12 |
| 79 | GO:0000302 | response to reactive oxygen species | 34/630 | 6230/237016 | 8.66E-05 | 0.001898 | 34 |
| 80 | GO:0048145 | regulation of fibroblast proliferation | 11/105 | 6230/237016 | 0.000106 | 0.002265 | 11 |
| 81 | GO:0048144 | fibroblast proliferation | 11/105 | 6230/237016 | 0.000106 | 0.002265 | 11 |
| 82 | GO:0048146 | positive regulation of fibroblast proliferation | 10/91 | 6230/237016 | 0.000145 | 0.003061 | 10 |
| 83 | GO:0050880 | regulation of blood vessel size | 19/276 | 6230/237016 | 0.000156 | 0.003158 | 19 |
| 84 | GO:0035150 | regulation of tube size | 19/276 | 6230/237016 | 0.000156 | 0.003158 | 19 |
| 85 | GO:2000377 | regulation of reactive oxygen species metabolic process | 18/253 | 6230/237016 | 0.000153 | 0.003158 | 18 |
| 86 | GO:0043281 | regulation of cysteine-type endopeptidase activity involved in apoptotic process | 19/276 | 6230/237016 | 0.000156 | 0.003158 | 19 |
| 87 | GO:0032611 | interleukin-1 beta production | 7/45 | 6230/237016 | 0.000163 | 0.003269 | 7 |
| 88 | GO:0030049 | muscle filament sliding | 5/21 | 6230/237016 | 0.000179 | 0.003515 | 5 |
| 89 | GO:0033275 | actin-myosin filament sliding | 5/21 | 6230/237016 | 0.000179 | 0.003515 | 5 |
| 90 | GO:0051271 | negative regulation of cellular component movement | 22/351 | 6230/237016 | 0.000191 | 0.003709 | 22 |
| 91 | GO:0046677 | response to antibiotic | 46/990 | 6230/237016 | 0.000198 | 0.003807 | 46 |
| 92 | GO:0001894 | tissue homeostasis | 13/153 | 6230/237016 | 0.000222 | 0.004215 | 13 |
| 93 | GO:0010631 | epithelial cell migration | 24/406 | 6230/237016 | 0.000241 | 0.004444 | 24 |
| 94 | GO:0090132 | epithelium migration | 24/406 | 6230/237016 | 0.000241 | 0.004444 | 24 |
| 95 | GO:0090130 | tissue migration | 24/406 | 6230/237016 | 0.000241 | 0.004444 | 24 |
| 96 | GO:0032652 | regulation of interleukin-1 production | 8/66 | 6230/237016 | 0.000335 | 0.005952 | 8 |
| 97 | GO:0070252 | actin-mediated cell contraction | 8/66 | 6230/237016 | 0.000335 | 0.005952 | 8 |
| 98 | GO:0030048 | actin filament-based movement | 8/66 | 6230/237016 | 0.000335 | 0.005952 | 8 |
| 99 | GO:0050920 | regulation of chemotaxis | 18/276 | 6230/237016 | 0.000438 | 0.007631 | 18 |
| 100 | GO:0034330 | cell junction organization | 18/276 | 6230/237016 | 0.000438 | 0.007631 | 18 |
| 101 | GO:0006066 | alcohol metabolic process | 19/300 | 6230/237016 | 0.000443 | 0.00765 | 19 |
| 102 | GO:0040013 | negative regulation of locomotion | 20/325 | 6230/237016 | 0.00046 | 0.007783 | 20 |
| 103 | GO:0033273 | response to vitamin | 10/105 | 6230/237016 | 0.000466 | 0.007823 | 10 |
| 104 | GO:1904951 | positive regulation of establishment of protein localization | 43/946 | 6230/237016 | 0.000488 | 0.008042 | 43 |
| 105 | GO:0030111 | regulation of Wnt signaling pathway | 21/351 | 6230/237016 | 0.000486 | 0.008042 | 21 |
| 106 | GO:0002576 | platelet degranulation | 15/210 | 6230/237016 | 0.0005 | 0.008167 | 15 |
| 107 | GO:0030258 | lipid modification | 22/378 | 6230/237016 | 0.000525 | 0.008505 | 22 |
| 108 | GO:0022407 | regulation of cell-cell adhesion | 49/1128 | 6230/237016 | 0.000568 | 0.009125 | 49 |
| 109 | GO:0001952 | regulation of cell-matrix adhesion | 7/55 | 6230/237016 | 0.000581 | 0.009167 | 7 |
| 110 | GO:0030177 | positive regulation of Wnt signaling pathway | 7/55 | 6230/237016 | 0.000581 | 0.009167 | 7 |
| 111 | GO:0050867 | positive regulation of cell activation | 34/703 | 6230/237016 | 0.00063 | 0.009861 | 34 |
| 112 | GO:0106106 | cold-induced thermogenesis | 13/171 | 6230/237016 | 0.000646 | 0.009861 | 13 |
| 113 | GO:0120161 | regulation of cold-induced thermogenesis | 13/171 | 6230/237016 | 0.000646 | 0.009861 | 13 |
| 114 | GO:1990845 | adaptive thermogenesis | 13/171 | 6230/237016 | 0.000646 | 0.009861 | 13 |
| 115 | GO:0043551 | regulation of phosphatidylinositol 3-kinase activity | 9/91 | 6230/237016 | 0.000671 | 0.009929 | 9 |
| 116 | GO:0050900 | leukocyte migration | 41/903 | 6230/237016 | 0.000662 | 0.009929 | 41 |
| 117 | GO:0071230 | cellular response to amino acid stimulus | 9/91 | 6230/237016 | 0.000671 | 0.009929 | 9 |
| 118 | GO:0021782 | glial cell development | 9/91 | 6230/237016 | 0.000671 | 0.009929 | 9 |
| 119 | GO:0045992 | negative regulation of embryonic development | 5/28 | 6230/237016 | 0.000743 | 0.010728 | 5 |
| 120 | GO:0032570 | response to progesterone | 5/28 | 6230/237016 | 0.000743 | 0.010728 | 5 |
| 121 | GO:0035272 | exocrine system development | 5/28 | 6230/237016 | 0.000743 | 0.010728 | 5 |
| 122 | GO:0006936 | muscle contraction | 31/630 | 6230/237016 | 0.000806 | 0.011463 | 31 |
| 123 | GO:0050727 | regulation of inflammatory response | 35/741 | 6230/237016 | 0.000801 | 0.011463 | 35 |
| 124 | GO:0070542 | response to fatty acid | 11/136 | 6230/237016 | 0.000988 | 0.013948 | 11 |
| 125 | GO:0043407 | negative regulation of MAP kinase activity | 8/78 | 6230/237016 | 0.001038 | 0.014429 | 8 |
| 126 | GO:0010565 | regulation of cellular ketone metabolic process | 8/78 | 6230/237016 | 0.001038 | 0.014429 | 8 |
| 127 | GO:0043255 | regulation of carbohydrate biosynthetic process | 6/45 | 6230/237016 | 0.001112 | 0.015351 | 6 |
| 128 | GO:0046777 | protein autophosphorylation | 19/325 | 6230/237016 | 0.00115 | 0.015412 | 19 |
| 129 | GO:2000146 | negative regulation of cell motility | 18/300 | 6230/237016 | 0.001144 | 0.015412 | 18 |
| 130 | GO:0007178 | transmembrane receptor protein serine/threonine kinase signaling pathway | 33/703 | 6230/237016 | 0.001223 | 0.016156 | 33 |
| 131 | GO:0055123 | digestive system development | 16/253 | 6230/237016 | 0.001221 | 0.016156 | 16 |
| 132 | GO:0050863 | regulation of T cell activation | 29/595 | 6230/237016 | 0.001339 | 0.017324 | 29 |
| 133 | GO:1903522 | regulation of blood circulation | 22/406 | 6230/237016 | 0.001323 | 0.017324 | 22 |
| 134 | GO:0008544 | epidermis development | 29/595 | 6230/237016 | 0.001339 | 0.017324 | 29 |
| 135 | GO:0022409 | positive regulation of cell-cell adhesion | 24/465 | 6230/237016 | 0.001582 | 0.020323 | 24 |
| 136 | GO:0031349 | positive regulation of defense response | 30/630 | 6230/237016 | 0.001595 | 0.020347 | 30 |
| 137 | GO:0002218 | activation of innate immune response | 13/190 | 6230/237016 | 0.00169 | 0.021414 | 13 |
| 138 | GO:0043550 | regulation of lipid kinase activity | 9/105 | 6230/237016 | 0.001858 | 0.022924 | 9 |
| 139 | GO:0042698 | ovulation cycle | 9/105 | 6230/237016 | 0.001858 | 0.022924 | 9 |
| 140 | GO:1903725 | regulation of phospholipid metabolic process | 9/105 | 6230/237016 | 0.001858 | 0.022924 | 9 |
| 141 | GO:2000379 | positive regulation of reactive oxygen species metabolic process | 9/105 | 6230/237016 | 0.001858 | 0.022924 | 9 |
| 142 | GO:0021700 | developmental maturation | 35/780 | 6230/237016 | 0.001887 | 0.023127 | 35 |
| 143 | GO:0010749 | regulation of nitric oxide mediated signal transduction | 2/3 | 6230/237016 | 0.002036 | 0.024792 | 2 |
| 144 | GO:0032651 | regulation of interleukin-1 beta production | 5/36 | 6230/237016 | 0.002395 | 0.028599 | 5 |
| 145 | GO:0019395 | fatty acid oxidation | 5/36 | 6230/237016 | 0.002395 | 0.028599 | 5 |
| 146 | GO:0034440 | lipid oxidation | 5/36 | 6230/237016 | 0.002395 | 0.028599 | 5 |
| 147 | GO:0001890 | placenta development | 11/153 | 6230/237016 | 0.002532 | 0.030045 | 11 |
| 148 | GO:0050870 | positive regulation of T cell activation | 17/300 | 6230/237016 | 0.002797 | 0.03298 | 17 |
| 149 | GO:0010632 | regulation of epithelial cell migration | 16/276 | 6230/237016 | 0.002932 | 0.034352 | 16 |
| 150 | GO:0031570 | DNA integrity checkpoint | 15/253 | 6230/237016 | 0.003157 | 0.035359 | 15 |
| 151 | GO:0061614 | pri-miRNA transcription by RNA polymerase II | 6/55 | 6230/237016 | 0.00317 | 0.035359 | 6 |
| 152 | GO:1903524 | positive regulation of blood circulation | 6/55 | 6230/237016 | 0.00317 | 0.035359 | 6 |
| 153 | GO:0010717 | regulation of epithelial to mesenchymal transition | 6/55 | 6230/237016 | 0.00317 | 0.035359 | 6 |
| 154 | GO:0051341 | regulation of oxidoreductase activity | 6/55 | 6230/237016 | 0.00317 | 0.035359 | 6 |
| 155 | GO:0071214 | cellular response to abiotic stimulus | 15/253 | 6230/237016 | 0.003157 | 0.035359 | 15 |
| 156 | GO:0104004 | cellular response to environmental stimulus | 15/253 | 6230/237016 | 0.003157 | 0.035359 | 15 |
| 157 | GO:0072511 | divalent inorganic cation transport | 22/435 | 6230/237016 | 0.003073 | 0.035359 | 22 |
| 158 | GO:0008015 | blood circulation | 70/1891 | 6230/237016 | 0.003264 | 0.035704 | 70 |
| 159 | GO:0003013 | circulatory system process | 70/1891 | 6230/237016 | 0.003264 | 0.035704 | 70 |
| 160 | GO:0060401 | cytosolic calcium ion transport | 10/136 | 6230/237016 | 0.003315 | 0.035704 | 10 |
| 161 | GO:0043588 | skin development | 23/465 | 6230/237016 | 0.003302 | 0.035704 | 23 |
| 162 | GO:0051099 | positive regulation of binding | 10/136 | 6230/237016 | 0.003315 | 0.035704 | 10 |
| 163 | GO:0031348 | negative regulation of defense response | 10/136 | 6230/237016 | 0.003315 | 0.035704 | 10 |
| 164 | GO:0097305 | response to alcohol | 26/561 | 6230/237016 | 0.004426 | 0.047117 | 26 |
| 165 | GO:0051222 | positive regulation of protein transport | 26/561 | 6230/237016 | 0.004426 | 0.047117 | 26 |
| 166 | GO:0070482 | response to oxygen levels | 45/1128 | 6230/237016 | 0.004518 | 0.047795 | 45 |
| 167 | GO:0043280 | positive regulation of cysteine-type endopeptidase activity involved in apoptotic process | 9/120 | 6230/237016 | 0.004567 | 0.047795 | 9 |
| 168 | GO:0050728 | negative regulation of inflammatory response | 9/120 | 6230/237016 | 0.004567 | 0.047795 | 9 |

**Common:**

| No. | ID | Description | GeneRatio | BgRatio | pvalue | p.adjust | count |
| --- | --- | --- | --- | --- | --- | --- | --- |
| 1 | GO:0071229 | cellular response to acid chemical | 63/780 | 6230/237016 | 1.07E-14 | 3.97E-12 | 63 |
| 2 | GO:0010975 | regulation of neuron projection development | 51/741 | 6230/237016 | 9.62E-10 | 1.28E-07 | 51 |
| 3 | GO:0006644 | phospholipid metabolic process | 37/496 | 6230/237016 | 2.31E-08 | 1.8E-06 | 37 |
| 4 | GO:0046486 | glycerolipid metabolic process | 33/435 | 6230/237016 | 8.68E-08 | 5.58E-06 | 33 |
| 5 | GO:0042063 | gliogenesis | 36/561 | 6230/237016 | 1.32E-06 | 6.29E-05 | 36 |
| 6 | GO:1901653 | cellular response to peptide | 47/903 | 6230/237016 | 1.14E-05 | 0.000374 | 47 |
| 7 | GO:0009314 | response to radiation | 38/780 | 6230/237016 | 0.000275 | 0.005027 | 38 |
| 8 | GO:0061564 | axon development | 28/528 | 6230/237016 | 0.000453 | 0.007739 | 28 |
| 9 | GO:0051897 | positive regulation of protein kinase B signaling | 18/300 | 6230/237016 | 0.001144 | 0.015412 | 18 |
| 10 | GO:0060541 | respiratory system development | 19/325 | 6230/237016 | 0.00115 | 0.015412 | 19 |
