# Supplementary material for: RNA-Seq-Based Breast Cancer Subtypes Classification Using Machine Learning Approaches
Source: Comput Intell Neurosci. 2020 Oct 29;2020:4737969. doi: 10.1155/2020/4737969 (PMC7644310; doi:10.1155/2020/4737969)
Supplement: Supplementary Materials — Figure S1: heatmap for Her2 and non Her2 groups. The left group 1 represents the Her2 group and the right group 2 denotes the non-Her2 group. Figure S2: heatmap for LumA and non-LumA groups. The left group 1 represents the LumA group and the right group 2 denotes the non-LumA group. Figure S3: heatmap for LumB and non-LumB groups. The left group 1 represents the LumB group and the right group 2 denotes the non-LumB group. Figure S4: heatmap for Normal-like and non-Normal-like groups. The left group 1 represents the Normal-like group and the right group 2 denotes the non-Normal-like group. S1 File: the detailed information of weighted DEGs for classification. S2 File: the detailed information of weighted DEGs for GO enrichment analysis. S3 File: the detailed enriched GO terms results for Basal-like subtype. S4 File: the detailed enriched GO terms results for Her2 subtype. S5 File: the detailed enriched GO terms results for LumA subtype. S6 File: the detailed enriched GO terms results for LumB subtype. S7 File: the detailed enriched GO terms results for Normal-like subtype. [file 4737969.f1.zip › supplementary materials/S6 File.docx]

**S6 File:** The detailed enriched GO terms results for LumB subtype.

**Control group:**

| No. | ID | Description | GeneRatio | BgRatio | pvalue | p.adjust | count |
| --- | --- | --- | --- | --- | --- | --- | --- |
| 1 | GO:0048732 | gland development | 443/2346 | 17025/152076 | 0 | 0 | 443 |
| 2 | GO:0002064 | epithelial cell development | 119/528 | 17025/152076 | 8.29E-14 | 3.06E-11 | 119 |
| 3 | GO:0048863 | stem cell differentiation | 128/595 | 17025/152076 | 3.52E-13 | 1.08E-10 | 128 |
| 4 | GO:0016202 | regulation of striated muscle tissue development | 63/231 | 17025/152076 | 1.36E-11 | 2.5E-09 | 63 |
| 5 | GO:1901861 | regulation of muscle tissue development | 63/231 | 17025/152076 | 1.36E-11 | 2.5E-09 | 63 |
| 6 | GO:0048634 | regulation of muscle organ development | 63/231 | 17025/152076 | 1.36E-11 | 2.5E-09 | 63 |
| 7 | GO:0061180 | mammary gland epithelium development | 28/66 | 17025/152076 | 1.04E-10 | 1.48E-08 | 28 |
| 8 | GO:0045165 | cell fate commitment | 157/861 | 17025/152076 | 6.81E-10 | 8.38E-08 | 157 |
| 9 | GO:0010631 | epithelial cell migration | 107/528 | 17025/152076 | 1.03E-09 | 1.12E-07 | 107 |
| 10 | GO:0090132 | epithelium migration | 107/528 | 17025/152076 | 1.03E-09 | 1.12E-07 | 107 |
| 11 | GO:0048638 | regulation of developmental growth | 144/780 | 17025/152076 | 1.45E-09 | 1.41E-07 | 144 |
| 12 | GO:0030850 | prostate gland development | 35/105 | 17025/152076 | 1.45E-09 | 1.41E-07 | 35 |
| 13 | GO:0001667 | ameboidal-type cell migration | 155/861 | 17025/152076 | 2.17E-09 | 1.9E-07 | 155 |
| 14 | GO:0070374 | positive regulation of ERK1 and ERK2 cascade | 116/595 | 17025/152076 | 2.28E-09 | 1.92E-07 | 116 |
| 15 | GO:0070371 | ERK1 and ERK2 cascade | 205/1225 | 17025/152076 | 3.78E-09 | 3.03E-07 | 205 |
| 16 | GO:0033044 | regulation of chromosome organization | 115/595 | 17025/152076 | 4.44E-09 | 3.41E-07 | 115 |
| 17 | GO:0045787 | positive regulation of cell cycle | 232/1431 | 17025/152076 | 6.33E-09 | 4.55E-07 | 232 |
| 18 | GO:0035148 | tube formation | 40/136 | 17025/152076 | 6.42E-09 | 4.55E-07 | 40 |
| 19 | GO:0070372 | regulation of ERK1 and ERK2 cascade | 189/1128 | 17025/152076 | 1.35E-08 | 8.59E-07 | 189 |
| 20 | GO:0051098 | regulation of binding | 98/496 | 17025/152076 | 1.89E-08 | 1.12E-06 | 98 |
| 21 | GO:0048285 | organelle fission | 266/1711 | 17025/152076 | 2.68E-08 | 1.55E-06 | 266 |
| 22 | GO:0060444 | branching involved in mammary gland duct morphogenesis | 11/15 | 17025/152076 | 3.06E-08 | 1.66E-06 | 11 |
| 23 | GO:0060603 | mammary gland duct morphogenesis | 11/15 | 17025/152076 | 3.06E-08 | 1.66E-06 | 11 |
| 24 | GO:0018209 | peptidyl-serine modification | 97/496 | 17025/152076 | 3.74E-08 | 1.97E-06 | 97 |
| 25 | GO:1901988 | negative regulation of cell cycle phase transition | 83/406 | 17025/152076 | 4.71E-08 | 2.29E-06 | 83 |
| 26 | GO:0055024 | regulation of cardiac muscle tissue development | 41/153 | 17025/152076 | 7.66E-08 | 3.53E-06 | 41 |
| 27 | GO:0018105 | peptidyl-serine phosphorylation | 91/465 | 17025/152076 | 9.29E-08 | 4.18E-06 | 91 |
| 28 | GO:0090596 | sensory organ morphogenesis | 47/190 | 17025/152076 | 1.29E-07 | 5.65E-06 | 47 |
| 29 | GO:2000241 | regulation of reproductive process | 43/171 | 17025/152076 | 2.64E-07 | 1.13E-05 | 43 |
| 30 | GO:0060740 | prostate gland epithelium morphogenesis | 23/66 | 17025/152076 | 3.62E-07 | 1.45E-05 | 23 |
| 31 | GO:0060512 | prostate gland morphogenesis | 23/66 | 17025/152076 | 3.62E-07 | 1.45E-05 | 23 |
| 32 | GO:0051099 | positive regulation of binding | 28/91 | 17025/152076 | 4.03E-07 | 1.58E-05 | 28 |
| 33 | GO:0001655 | urogenital system development | 194/1225 | 17025/152076 | 5.34E-07 | 2.05E-05 | 194 |
| 34 | GO:0006352 | DNA-templated transcription, initiation | 36/136 | 17025/152076 | 6.35E-07 | 2.39E-05 | 36 |
| 35 | GO:0048708 | astrocyte differentiation | 25/78 | 17025/152076 | 7.05E-07 | 2.55E-05 | 25 |
| 36 | GO:0034502 | protein localization to chromosome | 25/78 | 17025/152076 | 7.05E-07 | 2.55E-05 | 25 |
| 37 | GO:0046620 | regulation of organ growth | 30/105 | 17025/152076 | 9.32E-07 | 3.31E-05 | 30 |
| 38 | GO:0051783 | regulation of nuclear division | 132/780 | 17025/152076 | 1.08E-06 | 3.75E-05 | 132 |
| 39 | GO:1901991 | negative regulation of mitotic cell cycle phase transition | 74/378 | 17025/152076 | 1.35E-06 | 4.61E-05 | 74 |
| 40 | GO:0060688 | regulation of morphogenesis of a branching structure | 22/66 | 17025/152076 | 1.53E-06 | 5.13E-05 | 22 |
| 41 | GO:0045841 | negative regulation of mitotic metaphase/anaphase transition | 24/78 | 17025/152076 | 2.64E-06 | 8.4E-05 | 24 |
| 42 | GO:1902100 | negative regulation of metaphase/anaphase transition of cell cycle | 24/78 | 17025/152076 | 2.64E-06 | 8.4E-05 | 24 |
| 43 | GO:0055021 | regulation of cardiac muscle tissue growth | 24/78 | 17025/152076 | 2.64E-06 | 8.4E-05 | 24 |
| 44 | GO:0060443 | mammary gland morphogenesis | 13/28 | 17025/152076 | 3.15E-06 | 9.84E-05 | 13 |
| 45 | GO:0035264 | multicellular organism growth | 31/120 | 17025/152076 | 6.34E-06 | 0.000186 | 31 |
| 46 | GO:0010948 | negative regulation of cell cycle process | 117/703 | 17025/152076 | 9.22E-06 | 0.000266 | 117 |
| 47 | GO:0110110 | positive regulation of animal organ morphogenesis | 33/136 | 17025/152076 | 1.36E-05 | 0.000381 | 33 |
| 48 | GO:0001933 | negative regulation of protein phosphorylation | 111/666 | 17025/152076 | 1.44E-05 | 0.00039 | 111 |
| 49 | GO:0042326 | negative regulation of phosphorylation | 111/666 | 17025/152076 | 1.44E-05 | 0.00039 | 111 |
| 50 | GO:0060420 | regulation of heart growth | 25/91 | 17025/152076 | 1.54E-05 | 0.000405 | 25 |
| 51 | GO:2001235 | positive regulation of apoptotic signaling pathway | 25/91 | 17025/152076 | 1.54E-05 | 0.000405 | 25 |
| 52 | GO:0046622 | positive regulation of organ growth | 16/45 | 17025/152076 | 1.59E-05 | 0.000412 | 16 |
| 53 | GO:0055017 | cardiac muscle tissue growth | 30/120 | 17025/152076 | 1.77E-05 | 0.000442 | 30 |
| 54 | GO:0042303 | molting cycle | 30/120 | 17025/152076 | 1.77E-05 | 0.000442 | 30 |
| 55 | GO:0042633 | hair cycle | 30/120 | 17025/152076 | 1.77E-05 | 0.000442 | 30 |
| 56 | GO:0090068 | positive regulation of cell cycle process | 131/820 | 17025/152076 | 2.18E-05 | 0.000528 | 131 |
| 57 | GO:0035690 | cellular response to drug | 131/820 | 17025/152076 | 2.18E-05 | 0.000528 | 131 |
| 58 | GO:0032355 | response to estradiol | 51/253 | 17025/152076 | 2.39E-05 | 0.00056 | 51 |
| 59 | GO:1902903 | regulation of supramolecular fiber organization | 41/190 | 17025/152076 | 2.83E-05 | 0.000653 | 41 |
| 60 | GO:0001657 | ureteric bud development | 35/153 | 17025/152076 | 2.95E-05 | 0.000663 | 35 |
| 61 | GO:0072163 | mesonephric epithelium development | 35/153 | 17025/152076 | 2.95E-05 | 0.000663 | 35 |
| 62 | GO:0072073 | kidney epithelium development | 54/276 | 17025/152076 | 3.35E-05 | 0.000744 | 54 |
| 63 | GO:0060419 | heart growth | 32/136 | 17025/152076 | 3.52E-05 | 0.000772 | 32 |
| 64 | GO:2000816 | negative regulation of mitotic sister chromatid separation | 24/91 | 17025/152076 | 4.67E-05 | 0.00099 | 24 |
| 65 | GO:1905819 | negative regulation of chromosome separation | 24/91 | 17025/152076 | 4.67E-05 | 0.00099 | 24 |
| 66 | GO:0051101 | regulation of DNA binding | 24/91 | 17025/152076 | 4.67E-05 | 0.00099 | 24 |
| 67 | GO:0008584 | male gonad development | 50/253 | 17025/152076 | 4.88E-05 | 0.000993 | 50 |
| 68 | GO:0046546 | development of primary male sexual characteristics | 50/253 | 17025/152076 | 4.88E-05 | 0.000993 | 50 |
| 69 | GO:0007059 | chromosome segregation | 157/1035 | 17025/152076 | 5.83E-05 | 0.001156 | 157 |
| 70 | GO:0051304 | chromosome separation | 37/171 | 17025/152076 | 6.35E-05 | 0.001208 | 37 |
| 71 | GO:0001701 | in utero embryonic development | 76/435 | 17025/152076 | 6.32E-05 | 0.001208 | 76 |
| 72 | GO:0050680 | negative regulation of epithelial cell proliferation | 37/171 | 17025/152076 | 6.35E-05 | 0.001208 | 37 |
| 73 | GO:0060043 | regulation of cardiac muscle cell proliferation | 17/55 | 17025/152076 | 6.87E-05 | 0.00128 | 17 |
| 74 | GO:1902905 | positive regulation of supramolecular fiber organization | 34/153 | 17025/152076 | 7.05E-05 | 0.001301 | 34 |
| 75 | GO:0071168 | protein localization to chromatin | 8/15 | 17025/152076 | 7.64E-05 | 0.001382 | 8 |
| 76 | GO:0010812 | negative regulation of cell-substrate adhesion | 8/15 | 17025/152076 | 7.64E-05 | 0.001382 | 8 |
| 77 | GO:0055023 | positive regulation of cardiac muscle tissue growth | 13/36 | 17025/152076 | 8.17E-05 | 0.001449 | 13 |
| 78 | GO:0060421 | positive regulation of heart growth | 13/36 | 17025/152076 | 8.17E-05 | 0.001449 | 13 |
| 79 | GO:1901987 | regulation of cell cycle phase transition | 162/1081 | 17025/152076 | 8.35E-05 | 0.001467 | 162 |
| 80 | GO:0010965 | regulation of mitotic sister chromatid separation | 31/136 | 17025/152076 | 8.73E-05 | 0.001477 | 31 |
| 81 | GO:1905818 | regulation of chromosome separation | 31/136 | 17025/152076 | 8.73E-05 | 0.001477 | 31 |
| 82 | GO:0006338 | chromatin remodeling | 31/136 | 17025/152076 | 8.73E-05 | 0.001477 | 31 |
| 83 | GO:1904062 | regulation of cation transmembrane transport | 31/136 | 17025/152076 | 8.73E-05 | 0.001477 | 31 |
| 84 | GO:1902692 | regulation of neuroblast proliferation | 5/6 | 17025/152076 | 9.56E-05 | 0.001604 | 5 |
| 85 | GO:0000280 | nuclear division | 234/1653 | 17025/152076 | 0.000116 | 0.001936 | 234 |
| 86 | GO:0001889 | liver development | 28/120 | 17025/152076 | 0.000122 | 0.001994 | 28 |
| 87 | GO:0061008 | hepaticobiliary system development | 28/120 | 17025/152076 | 0.000122 | 0.001994 | 28 |
| 88 | GO:0001942 | hair follicle development | 23/91 | 17025/152076 | 0.000134 | 0.002102 | 23 |
| 89 | GO:0022404 | molting cycle process | 23/91 | 17025/152076 | 0.000134 | 0.002102 | 23 |
| 90 | GO:0022405 | hair cycle process | 23/91 | 17025/152076 | 0.000134 | 0.002102 | 23 |
| 91 | GO:0071236 | cellular response to antibiotic | 23/91 | 17025/152076 | 0.000134 | 0.002102 | 23 |
| 92 | GO:0002285 | lymphocyte activation involved in immune response | 23/91 | 17025/152076 | 0.000134 | 0.002102 | 23 |
| 93 | GO:0043583 | ear development | 36/171 | 17025/152076 | 0.000142 | 0.002198 | 36 |
| 94 | GO:0043588 | skin development | 70/406 | 17025/152076 | 0.000179 | 0.002747 | 70 |
| 95 | GO:0002040 | sprouting angiogenesis | 25/105 | 17025/152076 | 0.000196 | 0.002965 | 25 |
| 96 | GO:0071392 | cellular response to estradiol stimulus | 9/21 | 17025/152076 | 0.000229 | 0.003403 | 9 |
| 97 | GO:1901655 | cellular response to ketone | 18/66 | 17025/152076 | 0.000252 | 0.003726 | 18 |
| 98 | GO:0034330 | cell junction organization | 41/210 | 17025/152076 | 0.000287 | 0.004036 | 41 |
| 99 | GO:0002067 | glandular epithelial cell differentiation | 27/120 | 17025/152076 | 0.0003 | 0.004185 | 27 |
| 100 | GO:0000075 | cell cycle checkpoint | 69/406 | 17025/152076 | 0.000302 | 0.004185 | 69 |
| 101 | GO:0016331 | morphogenesis of embryonic epithelium | 35/171 | 17025/152076 | 0.000306 | 0.004218 | 35 |
| 102 | GO:0046777 | protein autophosphorylation | 47/253 | 17025/152076 | 0.000359 | 0.004804 | 47 |
| 103 | GO:0003300 | cardiac muscle hypertrophy | 12/36 | 17025/152076 | 0.000362 | 0.004804 | 12 |
| 104 | GO:0014897 | striated muscle hypertrophy | 12/36 | 17025/152076 | 0.000362 | 0.004804 | 12 |
| 105 | GO:0043401 | steroid hormone mediated signaling pathway | 22/91 | 17025/152076 | 0.000367 | 0.004832 | 22 |
| 106 | GO:0048469 | cell maturation | 50/276 | 17025/152076 | 0.000439 | 0.005698 | 50 |
| 107 | GO:0033046 | negative regulation of sister chromatid segregation | 24/105 | 17025/152076 | 0.000495 | 0.006261 | 24 |
| 108 | GO:0051985 | negative regulation of chromosome segregation | 24/105 | 17025/152076 | 0.000495 | 0.006261 | 24 |
| 109 | GO:0045839 | negative regulation of mitotic nuclear division | 24/105 | 17025/152076 | 0.000495 | 0.006261 | 24 |
| 110 | GO:0098773 | skin epidermis development | 24/105 | 17025/152076 | 0.000495 | 0.006261 | 24 |
| 111 | GO:0014706 | striated muscle tissue development | 181/1275 | 17025/152076 | 0.000551 | 0.00691 | 181 |
| 112 | GO:0060045 | positive regulation of cardiac muscle cell proliferation | 10/28 | 17025/152076 | 0.000598 | 0.007399 | 10 |
| 113 | GO:2000243 | positive regulation of reproductive process | 10/28 | 17025/152076 | 0.000598 | 0.007399 | 10 |
| 114 | GO:0061900 | glial cell activation | 7/15 | 17025/152076 | 0.000625 | 0.007631 | 7 |
| 115 | GO:0033045 | regulation of sister chromatid segregation | 34/171 | 17025/152076 | 0.00064 | 0.007771 | 34 |
| 116 | GO:0048661 | positive regulation of smooth muscle cell proliferation | 26/120 | 17025/152076 | 0.000707 | 0.008527 | 26 |
| 117 | GO:0060537 | muscle tissue development | 193/1378 | 17025/152076 | 0.000722 | 0.008656 | 193 |
| 118 | GO:0010876 | lipid localization | 56/325 | 17025/152076 | 0.000746 | 0.008827 | 56 |
| 119 | GO:0051052 | regulation of DNA metabolic process | 56/325 | 17025/152076 | 0.000746 | 0.008827 | 56 |
| 120 | GO:0051785 | positive regulation of nuclear division | 17/66 | 17025/152076 | 0.000761 | 0.008888 | 17 |
| 121 | GO:0060038 | cardiac muscle cell proliferation | 17/66 | 17025/152076 | 0.000761 | 0.008888 | 17 |
| 122 | GO:1902750 | negative regulation of cell cycle G2/M phase transition | 15/55 | 17025/152076 | 0.000804 | 0.009212 | 15 |
| 123 | GO:1990868 | response to chemokine | 15/55 | 17025/152076 | 0.000804 | 0.009212 | 15 |
| 124 | GO:1990869 | cellular response to chemokine | 15/55 | 17025/152076 | 0.000804 | 0.009212 | 15 |
| 125 | GO:1901654 | response to ketone | 79/496 | 17025/152076 | 0.000886 | 0.01006 | 79 |
| 126 | GO:1901990 | regulation of mitotic cell cycle phase transition | 149/1035 | 17025/152076 | 0.000897 | 0.010093 | 149 |
| 127 | GO:0055025 | positive regulation of cardiac muscle tissue development | 13/45 | 17025/152076 | 0.000981 | 0.010834 | 13 |
| 128 | GO:0048013 | ephrin receptor signaling pathway | 13/45 | 17025/152076 | 0.000981 | 0.010834 | 13 |
| 129 | GO:0042472 | inner ear morphogenesis | 13/45 | 17025/152076 | 0.000981 | 0.010834 | 13 |
| 130 | GO:0046677 | response to antibiotic | 121/820 | 17025/152076 | 0.001059 | 0.011561 | 121 |
| 131 | GO:0045930 | negative regulation of mitotic cell cycle | 101/666 | 17025/152076 | 0.001073 | 0.011581 | 101 |
| 132 | GO:0060249 | anatomical structure homeostasis | 101/666 | 17025/152076 | 0.001073 | 0.011581 | 101 |
| 133 | GO:0048639 | positive regulation of developmental growth | 36/190 | 17025/152076 | 0.001135 | 0.01218 | 36 |
| 134 | GO:0014855 | striated muscle cell proliferation | 23/105 | 17025/152076 | 0.001191 | 0.012703 | 23 |
| 135 | GO:0051983 | regulation of chromosome segregation | 45/253 | 17025/152076 | 0.001205 | 0.012775 | 45 |
| 136 | GO:0010389 | regulation of G2/M transition of mitotic cell cycle | 48/276 | 17025/152076 | 0.001386 | 0.013954 | 48 |
| 137 | GO:0071772 | response to BMP | 48/276 | 17025/152076 | 0.001386 | 0.013954 | 48 |
| 138 | GO:0071773 | cellular response to BMP stimulus | 48/276 | 17025/152076 | 0.001386 | 0.013954 | 48 |
| 139 | GO:1902749 | regulation of cell cycle G2/M phase transition | 48/276 | 17025/152076 | 0.001386 | 0.013954 | 48 |
| 140 | GO:0150076 | neuroinflammatory response | 11/36 | 17025/152076 | 0.001429 | 0.013954 | 11 |
| 141 | GO:0010972 | negative regulation of G2/M transition of mitotic cell cycle | 11/36 | 17025/152076 | 0.001429 | 0.013954 | 11 |
| 142 | GO:0045843 | negative regulation of striated muscle tissue development | 11/36 | 17025/152076 | 0.001429 | 0.013954 | 11 |
| 143 | GO:0042093 | T-helper cell differentiation | 11/36 | 17025/152076 | 0.001429 | 0.013954 | 11 |
| 144 | GO:0048635 | negative regulation of muscle organ development | 11/36 | 17025/152076 | 0.001429 | 0.013954 | 11 |
| 145 | GO:1901862 | negative regulation of muscle tissue development | 11/36 | 17025/152076 | 0.001429 | 0.013954 | 11 |
| 146 | GO:0002287 | alpha-beta T cell activation involved in immune response | 11/36 | 17025/152076 | 0.001429 | 0.013954 | 11 |
| 147 | GO:0002292 | T cell differentiation involved in immune response | 11/36 | 17025/152076 | 0.001429 | 0.013954 | 11 |
| 148 | GO:0060736 | prostate gland growth | 3/3 | 17025/152076 | 0.001403 | 0.013954 | 3 |
| 149 | GO:0002244 | hematopoietic progenitor cell differentiation | 11/36 | 17025/152076 | 0.001429 | 0.013954 | 11 |
| 150 | GO:0030033 | microvillus assembly | 3/3 | 17025/152076 | 0.001403 | 0.013954 | 3 |
| 151 | GO:0050679 | positive regulation of epithelial cell proliferation | 62/378 | 17025/152076 | 0.001447 | 0.014047 | 62 |
| 152 | GO:0007088 | regulation of mitotic nuclear division | 95/630 | 17025/152076 | 0.001753 | 0.01667 | 95 |
| 153 | GO:0060218 | hematopoietic stem cell differentiation | 4/6 | 17025/152076 | 0.001953 | 0.018386 | 4 |
| 154 | GO:1901532 | regulation of hematopoietic progenitor cell differentiation | 4/6 | 17025/152076 | 0.001953 | 0.018386 | 4 |
| 155 | GO:0045598 | regulation of fat cell differentiation | 41/231 | 17025/152076 | 0.002004 | 0.01877 | 41 |
| 156 | GO:0002066 | columnar/cuboidal epithelial cell development | 18/78 | 17025/152076 | 0.002113 | 0.019399 | 18 |
| 157 | GO:0006939 | smooth muscle contraction | 18/78 | 17025/152076 | 0.002113 | 0.019399 | 18 |
| 158 | GO:2000736 | regulation of stem cell differentiation | 16/66 | 17025/152076 | 0.002134 | 0.01949 | 16 |
| 159 | GO:0009755 | hormone-mediated signaling pathway | 27/136 | 17025/152076 | 0.002223 | 0.020202 | 27 |
| 160 | GO:0007409 | axonogenesis | 81/528 | 17025/152076 | 0.002261 | 0.020451 | 81 |
| 161 | GO:0001894 | tissue homeostasis | 47/276 | 17025/152076 | 0.002381 | 0.021328 | 47 |
| 162 | GO:0000083 | regulation of transcription involved in G1/S transition of mitotic cell cycle | 14/55 | 17025/152076 | 0.002427 | 0.021634 | 14 |
| 163 | GO:0001838 | embryonic epithelial tube formation | 22/105 | 17025/152076 | 0.002721 | 0.023367 | 22 |
| 164 | GO:0072175 | epithelial tube formation | 22/105 | 17025/152076 | 0.002721 | 0.023367 | 22 |
| 165 | GO:1903429 | regulation of cell maturation | 5/10 | 17025/152076 | 0.002723 | 0.023367 | 5 |
| 166 | GO:0071897 | DNA biosynthetic process | 22/105 | 17025/152076 | 0.002721 | 0.023367 | 22 |
| 167 | GO:0048538 | thymus development | 5/10 | 17025/152076 | 0.002723 | 0.023367 | 5 |
| 168 | GO:0032873 | negative regulation of stress-activated MAPK cascade | 5/10 | 17025/152076 | 0.002723 | 0.023367 | 5 |
| 169 | GO:0070303 | negative regulation of stress-activated protein kinase signaling cascade | 5/10 | 17025/152076 | 0.002723 | 0.023367 | 5 |
| 170 | GO:0070098 | chemokine-mediated signaling pathway | 12/45 | 17025/152076 | 0.003194 | 0.026783 | 12 |
| 171 | GO:0014896 | muscle hypertrophy | 12/45 | 17025/152076 | 0.003194 | 0.026783 | 12 |
| 172 | GO:0051784 | negative regulation of nuclear division | 24/120 | 17025/152076 | 0.003417 | 0.028271 | 24 |
| 173 | GO:0110053 | regulation of actin filament organization | 24/120 | 17025/152076 | 0.003417 | 0.028271 | 24 |
| 174 | GO:0070555 | response to interleukin-1 | 40/231 | 17025/152076 | 0.003535 | 0.029118 | 40 |
| 175 | GO:0043409 | negative regulation of MAPK cascade | 37/210 | 17025/152076 | 0.003632 | 0.029786 | 37 |
| 176 | GO:0032956 | regulation of actin cytoskeleton organization | 43/253 | 17025/152076 | 0.003659 | 0.029867 | 43 |
| 177 | GO:0010165 | response to X-ray | 6/15 | 17025/152076 | 0.004008 | 0.032429 | 6 |
| 178 | GO:1905332 | positive regulation of morphogenesis of an epithelium | 6/15 | 17025/152076 | 0.004008 | 0.032429 | 6 |
| 179 | GO:0045444 | fat cell differentiation | 88/595 | 17025/152076 | 0.004297 | 0.034623 | 88 |
| 180 | GO:0051249 | regulation of lymphocyte activation | 127/903 | 17025/152076 | 0.004473 | 0.035869 | 127 |
| 181 | GO:0051495 | positive regulation of cytoskeleton organization | 31/171 | 17025/152076 | 0.004758 | 0.037675 | 31 |
| 182 | GO:0006940 | regulation of smooth muscle contraction | 10/36 | 17025/152076 | 0.005014 | 0.039195 | 10 |
| 183 | GO:0010675 | regulation of cellular carbohydrate metabolic process | 10/36 | 17025/152076 | 0.005014 | 0.039195 | 10 |
| 184 | GO:0061564 | axon development | 106/741 | 17025/152076 | 0.005321 | 0.041422 | 106 |
| 185 | GO:0014031 | mesenchymal cell development | 15/66 | 17025/152076 | 0.005552 | 0.04268 | 15 |
| 186 | GO:0045582 | positive regulation of T cell differentiation | 15/66 | 17025/152076 | 0.005552 | 0.04268 | 15 |
| 187 | GO:0000070 | mitotic sister chromatid segregation | 59/378 | 17025/152076 | 0.005605 | 0.042907 | 59 |
| 188 | GO:0031581 | hemidesmosome assembly | 7/21 | 17025/152076 | 0.00616 | 0.046769 | 7 |
| 189 | GO:0031069 | hair follicle morphogenesis | 7/21 | 17025/152076 | 0.00616 | 0.046769 | 7 |
| 190 | GO:0046661 | male sex differentiation | 55/351 | 17025/152076 | 0.006707 | 0.048633 | 55 |
| 191 | GO:0050878 | regulation of body fluid levels | 131/946 | 17025/152076 | 0.006591 | 0.048633 | 131 |
| 192 | GO:0007094 | mitotic spindle assembly checkpoint | 13/55 | 17025/152076 | 0.006722 | 0.048633 | 13 |
| 193 | GO:0031577 | spindle checkpoint | 13/55 | 17025/152076 | 0.006722 | 0.048633 | 13 |
| 194 | GO:0071173 | spindle assembly checkpoint | 13/55 | 17025/152076 | 0.006722 | 0.048633 | 13 |
| 195 | GO:0007411 | axon guidance | 45/276 | 17025/152076 | 0.006548 | 0.048633 | 45 |
| 196 | GO:0097485 | neuron projection guidance | 45/276 | 17025/152076 | 0.006548 | 0.048633 | 45 |
| 197 | GO:0045844 | positive regulation of striated muscle tissue development | 13/55 | 17025/152076 | 0.006722 | 0.048633 | 13 |
| 198 | GO:0048636 | positive regulation of muscle organ development | 13/55 | 17025/152076 | 0.006722 | 0.048633 | 13 |
| 199 | GO:1901863 | positive regulation of muscle tissue development | 13/55 | 17025/152076 | 0.006722 | 0.048633 | 13 |
| 200 | GO:0030010 | establishment of cell polarity | 13/55 | 17025/152076 | 0.006722 | 0.048633 | 13 |

**Experiment group:**

| No. | ID | Description | GeneRatio | BgRatio | pvalue | p.adjust | count |
| --- | --- | --- | --- | --- | --- | --- | --- |
| 1 | GO:0030198 | extracellular matrix organization | 91/1176 | 3512/152076 | 0 | 0 | 91 |
| 2 | GO:0060541 | respiratory system development | 44/378 | 3512/152076 | 0 | 0 | 44 |
| 3 | GO:0042063 | gliogenesis | 62/780 | 3512/152076 | 1.11E-16 | 5.12E-14 | 62 |
| 4 | GO:0001503 | ossification | 91/1596 | 3512/152076 | 1.09E-14 | 2.87E-12 | 91 |
| 5 | GO:0030324 | lung development | 32/253 | 3512/152076 | 1.02E-14 | 2.87E-12 | 32 |
| 6 | GO:0030323 | respiratory tube development | 32/253 | 3512/152076 | 1.02E-14 | 2.87E-12 | 32 |
| 7 | GO:0031668 | cellular response to extracellular stimulus | 28/300 | 3512/152076 | 6.44E-10 | 9.9E-08 | 28 |
| 8 | GO:0001649 | osteoblast differentiation | 39/561 | 3512/152076 | 2.03E-09 | 2.68E-07 | 39 |
| 9 | GO:0009612 | response to mechanical stimulus | 31/378 | 3512/152076 | 1.91E-09 | 2.68E-07 | 31 |
| 10 | GO:0090288 | negative regulation of cellular response to growth factor stimulus | 15/91 | 3512/152076 | 2.9E-09 | 3.56E-07 | 15 |
| 11 | GO:0043200 | response to amino acid | 19/153 | 3512/152076 | 3.34E-09 | 3.86E-07 | 19 |
| 12 | GO:0051090 | regulation of DNA-binding transcription factor activity | 41/630 | 3512/152076 | 5.32E-09 | 5.45E-07 | 41 |
| 13 | GO:0007369 | gastrulation | 24/253 | 3512/152076 | 7.55E-09 | 7.33E-07 | 24 |
| 14 | GO:0036293 | response to decreased oxygen levels | 43/703 | 3512/152076 | 1.39E-08 | 1.22E-06 | 43 |
| 15 | GO:0016049 | cell growth | 43/703 | 3512/152076 | 1.39E-08 | 1.22E-06 | 43 |
| 16 | GO:0045927 | positive regulation of growth | 33/465 | 3512/152076 | 2.07E-08 | 1.59E-06 | 33 |
| 17 | GO:0048771 | tissue remodeling | 16/120 | 3512/152076 | 2.04E-08 | 1.59E-06 | 16 |
| 18 | GO:0070482 | response to oxygen levels | 44/741 | 3512/152076 | 2.25E-08 | 1.63E-06 | 44 |
| 19 | GO:0001704 | formation of primary germ layer | 20/190 | 3512/152076 | 2.29E-08 | 1.63E-06 | 20 |
| 20 | GO:0001666 | response to hypoxia | 41/666 | 3512/152076 | 2.5E-08 | 1.65E-06 | 41 |
| 21 | GO:0010717 | regulation of epithelial to mesenchymal transition | 14/91 | 3512/152076 | 2.42E-08 | 1.65E-06 | 14 |
| 22 | GO:0090101 | negative regulation of transmembrane receptor protein serine/threonine kinase signaling pathway | 11/55 | 3512/152076 | 4.61E-08 | 2.93E-06 | 11 |
| 23 | GO:0097191 | extrinsic apoptotic signaling pathway | 25/300 | 3512/152076 | 4.91E-08 | 3.02E-06 | 25 |
| 24 | GO:1903708 | positive regulation of hemopoiesis | 26/325 | 3512/152076 | 6.07E-08 | 3.61E-06 | 26 |
| 25 | GO:0071496 | cellular response to external stimulus | 33/496 | 3512/152076 | 9.41E-08 | 5.26E-06 | 33 |
| 26 | GO:2001236 | regulation of extrinsic apoptotic signaling pathway | 21/231 | 3512/152076 | 1.31E-07 | 6.84E-06 | 21 |
| 27 | GO:2001237 | negative regulation of extrinsic apoptotic signaling pathway | 15/120 | 3512/152076 | 1.33E-07 | 6.84E-06 | 15 |
| 28 | GO:0001558 | regulation of cell growth | 32/496 | 3512/152076 | 2.89E-07 | 1.4E-05 | 32 |
| 29 | GO:0007219 | Notch signaling pathway | 17/171 | 3512/152076 | 5.7E-07 | 2.5E-05 | 17 |
| 30 | GO:0032680 | regulation of tumor necrosis factor production | 14/120 | 3512/152076 | 8.12E-07 | 3.26E-05 | 14 |
| 31 | GO:1903555 | regulation of tumor necrosis factor superfamily cytokine production | 14/120 | 3512/152076 | 8.12E-07 | 3.26E-05 | 14 |
| 32 | GO:0055074 | calcium ion homeostasis | 36/630 | 3512/152076 | 9.9E-07 | 3.81E-05 | 36 |
| 33 | GO:0072503 | cellular divalent inorganic cation homeostasis | 36/630 | 3512/152076 | 9.9E-07 | 3.81E-05 | 36 |
| 34 | GO:1904018 | positive regulation of vasculature development | 28/435 | 3512/152076 | 1.61E-06 | 5.95E-05 | 28 |
| 35 | GO:0072593 | reactive oxygen species metabolic process | 28/435 | 3512/152076 | 1.61E-06 | 5.95E-05 | 28 |
| 36 | GO:0033627 | cell adhesion mediated by integrin | 10/66 | 3512/152076 | 2.77E-06 | 9.63E-05 | 10 |
| 37 | GO:0032640 | tumor necrosis factor production | 14/136 | 3512/152076 | 3.66E-06 | 0.000118 | 14 |
| 38 | GO:0071706 | tumor necrosis factor superfamily cytokine production | 14/136 | 3512/152076 | 3.66E-06 | 0.000118 | 14 |
| 39 | GO:0048608 | reproductive structure development | 65/1540 | 3512/152076 | 3.99E-06 | 0.000125 | 65 |
| 40 | GO:0061458 | reproductive system development | 65/1540 | 3512/152076 | 3.99E-06 | 0.000125 | 65 |
| 41 | GO:0007584 | response to nutrient | 27/435 | 3512/152076 | 4.79E-06 | 0.00014 | 27 |
| 42 | GO:0001101 | response to acid chemical | 70/1711 | 3512/152076 | 4.97E-06 | 0.000143 | 70 |
| 43 | GO:0045621 | positive regulation of lymphocyte differentiation | 12/105 | 3512/152076 | 6.06E-06 | 0.000172 | 12 |
| 44 | GO:0046486 | glycerolipid metabolic process | 34/630 | 3512/152076 | 6.6E-06 | 0.000185 | 34 |
| 45 | GO:0010718 | positive regulation of epithelial to mesenchymal transition | 8/45 | 3512/152076 | 8.08E-06 | 0.000222 | 8 |
| 46 | GO:0051091 | positive regulation of DNA-binding transcription factor activity | 20/276 | 3512/152076 | 8.53E-06 | 0.000232 | 20 |
| 47 | GO:0045766 | positive regulation of angiogenesis | 23/351 | 3512/152076 | 9.86E-06 | 0.000256 | 23 |
| 48 | GO:1903706 | regulation of hemopoiesis | 43/903 | 3512/152076 | 1.02E-05 | 0.000257 | 43 |
| 49 | GO:0045017 | glycerolipid biosynthetic process | 13/136 | 3512/152076 | 1.8E-05 | 0.000443 | 13 |
| 50 | GO:0060191 | regulation of lipase activity | 12/120 | 3512/152076 | 2.38E-05 | 0.000578 | 12 |
| 51 | GO:0006979 | response to oxidative stress | 46/1035 | 3512/152076 | 2.82E-05 | 0.000672 | 46 |
| 52 | GO:0090287 | regulation of cellular response to growth factor stimulus | 20/300 | 3512/152076 | 2.84E-05 | 0.000672 | 20 |
| 53 | GO:0040013 | negative regulation of locomotion | 19/276 | 3512/152076 | 2.88E-05 | 0.000672 | 19 |
| 54 | GO:2000377 | regulation of reactive oxygen species metabolic process | 18/253 | 3512/152076 | 3E-05 | 0.00068 | 18 |
| 55 | GO:1905330 | regulation of morphogenesis of an epithelium | 16/210 | 3512/152076 | 3.58E-05 | 0.000795 | 16 |
| 56 | GO:0070849 | response to epidermal growth factor | 6/28 | 3512/152076 | 3.68E-05 | 0.000798 | 6 |
| 57 | GO:0022617 | extracellular matrix disassembly | 6/28 | 3512/152076 | 3.68E-05 | 0.000798 | 6 |
| 58 | GO:0010517 | regulation of phospholipase activity | 10/91 | 3512/152076 | 5E-05 | 0.001072 | 10 |
| 59 | GO:0031667 | response to nutrient levels | 45/1035 | 3512/152076 | 5.7E-05 | 0.001209 | 45 |
| 60 | GO:0030307 | positive regulation of cell growth | 13/153 | 3512/152076 | 6.21E-05 | 0.001301 | 13 |
| 61 | GO:0050900 | leukocyte migration | 54/1326 | 3512/152076 | 6.29E-05 | 0.001304 | 54 |
| 62 | GO:0008593 | regulation of Notch signaling pathway | 7/45 | 3512/152076 | 7.32E-05 | 0.001501 | 7 |
| 63 | GO:0007178 | transmembrane receptor protein serine/threonine kinase signaling pathway | 30/595 | 3512/152076 | 7.62E-05 | 0.001544 | 30 |
| 64 | GO:1903524 | positive regulation of blood circulation | 9/78 | 3512/152076 | 8.04E-05 | 0.001612 | 9 |
| 65 | GO:0046488 | phosphatidylinositol metabolic process | 21/351 | 3512/152076 | 8.66E-05 | 0.001672 | 21 |
| 66 | GO:0050920 | regulation of chemotaxis | 19/300 | 3512/152076 | 8.79E-05 | 0.001672 | 19 |
| 67 | GO:0090092 | regulation of transmembrane receptor protein serine/threonine kinase signaling pathway | 19/300 | 3512/152076 | 8.79E-05 | 0.001672 | 19 |
| 68 | GO:1903522 | regulation of blood circulation | 19/300 | 3512/152076 | 8.79E-05 | 0.001672 | 19 |
| 69 | GO:0032102 | negative regulation of response to external stimulus | 20/325 | 3512/152076 | 8.62E-05 | 0.001672 | 20 |
| 70 | GO:0060627 | regulation of vesicle-mediated transport | 31/630 | 3512/152076 | 9.08E-05 | 0.001709 | 31 |
| 71 | GO:0050766 | positive regulation of phagocytosis | 5/21 | 3512/152076 | 9.78E-05 | 0.001823 | 5 |
| 72 | GO:0072511 | divalent inorganic cation transport | 16/231 | 3512/152076 | 0.000111 | 0.002001 | 16 |
| 73 | GO:0010518 | positive regulation of phospholipase activity | 8/66 | 3512/152076 | 0.00014 | 0.002439 | 8 |
| 74 | GO:0060193 | positive regulation of lipase activity | 8/66 | 3512/152076 | 0.00014 | 0.002439 | 8 |
| 75 | GO:0003013 | circulatory system process | 48/1176 | 3512/152076 | 0.000147 | 0.002535 | 48 |
| 76 | GO:0051968 | positive regulation of synaptic transmission, glutamatergic | 3/6 | 3512/152076 | 0.000234 | 0.003882 | 3 |
| 77 | GO:0050727 | regulation of inflammatory response | 36/820 | 3512/152076 | 0.000246 | 0.003919 | 36 |
| 78 | GO:0051271 | negative regulation of cellular component movement | 19/325 | 3512/152076 | 0.000245 | 0.003919 | 19 |
| 79 | GO:0048146 | positive regulation of fibroblast proliferation | 9/91 | 3512/152076 | 0.000264 | 0.004168 | 9 |
| 80 | GO:0018108 | peptidyl-tyrosine phosphorylation | 53/1378 | 3512/152076 | 0.000291 | 0.004476 | 53 |
| 81 | GO:0018212 | peptidyl-tyrosine modification | 53/1378 | 3512/152076 | 0.000291 | 0.004476 | 53 |
| 82 | GO:0002697 | regulation of immune effector process | 25/496 | 3512/152076 | 0.000289 | 0.004476 | 25 |
| 83 | GO:0061448 | connective tissue development | 37/861 | 3512/152076 | 0.000306 | 0.004656 | 37 |
| 84 | GO:0030178 | negative regulation of Wnt signaling pathway | 16/253 | 3512/152076 | 0.00031 | 0.004656 | 16 |
| 85 | GO:0030514 | negative regulation of BMP signaling pathway | 4/15 | 3512/152076 | 0.000316 | 0.004702 | 4 |
| 86 | GO:0007160 | cell-matrix adhesion | 11/136 | 3512/152076 | 0.000341 | 0.005038 | 11 |
| 87 | GO:0007565 | female pregnancy | 15/231 | 3512/152076 | 0.000357 | 0.005228 | 15 |
| 88 | GO:0017145 | stem cell division | 5/28 | 3512/152076 | 0.000413 | 0.005775 | 5 |
| 89 | GO:0060412 | ventricular septum morphogenesis | 5/28 | 3512/152076 | 0.000413 | 0.005775 | 5 |
| 90 | GO:0032729 | positive regulation of interferon-gamma production | 5/28 | 3512/152076 | 0.000413 | 0.005775 | 5 |
| 91 | GO:0060071 | Wnt signaling pathway, planar cell polarity pathway | 5/28 | 3512/152076 | 0.000413 | 0.005775 | 5 |
| 92 | GO:0090175 | regulation of establishment of planar polarity | 5/28 | 3512/152076 | 0.000413 | 0.005775 | 5 |
| 93 | GO:0045619 | regulation of lymphocyte differentiation | 14/210 | 3512/152076 | 0.000425 | 0.0059 | 14 |
| 94 | GO:0072089 | stem cell proliferation | 8/78 | 3512/152076 | 0.000448 | 0.006173 | 8 |
| 95 | GO:0060759 | regulation of response to cytokine stimulus | 10/120 | 3512/152076 | 0.000495 | 0.006759 | 10 |
| 96 | GO:0032652 | regulation of interleukin-1 production | 6/45 | 3512/152076 | 0.000569 | 0.007657 | 6 |
| 97 | GO:0032612 | interleukin-1 production | 6/45 | 3512/152076 | 0.000569 | 0.007657 | 6 |
| 98 | GO:0031589 | cell-substrate adhesion | 21/406 | 3512/152076 | 0.000608 | 0.008129 | 21 |
| 99 | GO:0001837 | epithelial to mesenchymal transition | 12/171 | 3512/152076 | 0.000679 | 0.009019 | 12 |
| 100 | GO:0050807 | regulation of synapse organization | 9/105 | 3512/152076 | 0.000761 | 0.009953 | 9 |
| 101 | GO:0050803 | regulation of synapse structure or activity | 9/105 | 3512/152076 | 0.000761 | 0.009953 | 9 |
| 102 | GO:0046834 | lipid phosphorylation | 16/276 | 3512/152076 | 0.000797 | 0.010137 | 16 |
| 103 | GO:0002526 | acute inflammatory response | 16/276 | 3512/152076 | 0.000797 | 0.010137 | 16 |
| 104 | GO:0001818 | negative regulation of cytokine production | 16/276 | 3512/152076 | 0.000797 | 0.010137 | 16 |
| 105 | GO:0031670 | cellular response to nutrient | 7/66 | 3512/152076 | 0.000825 | 0.01015 | 7 |
| 106 | GO:0003281 | ventricular septum development | 7/66 | 3512/152076 | 0.000825 | 0.01015 | 7 |
| 107 | GO:0032649 | regulation of interferon-gamma production | 7/66 | 3512/152076 | 0.000825 | 0.01015 | 7 |
| 108 | GO:0021782 | glial cell development | 7/66 | 3512/152076 | 0.000825 | 0.01015 | 7 |
| 109 | GO:0032609 | interferon-gamma production | 7/66 | 3512/152076 | 0.000825 | 0.01015 | 7 |
| 110 | GO:0022409 | positive regulation of cell-cell adhesion | 34/820 | 3512/152076 | 0.00096 | 0.011727 | 34 |
| 111 | GO:0002685 | regulation of leukocyte migration | 14/231 | 3512/152076 | 0.001079 | 0.013015 | 14 |
| 112 | GO:2000146 | negative regulation of cell motility | 14/231 | 3512/152076 | 0.001079 | 0.013015 | 14 |
| 113 | GO:0032570 | response to progesterone | 4/21 | 3512/152076 | 0.001241 | 0.014769 | 4 |
| 114 | GO:0071364 | cellular response to epidermal growth factor stimulus | 4/21 | 3512/152076 | 0.001241 | 0.014769 | 4 |
| 115 | GO:0007249 | I-kappaB kinase/NF-kappaB signaling | 13/210 | 3512/152076 | 0.001323 | 0.015644 | 13 |
| 116 | GO:0001736 | establishment of planar polarity | 5/36 | 3512/152076 | 0.00136 | 0.015784 | 5 |
| 117 | GO:0007164 | establishment of tissue polarity | 5/36 | 3512/152076 | 0.00136 | 0.015784 | 5 |
| 118 | GO:0060021 | roof of mouth development | 5/36 | 3512/152076 | 0.00136 | 0.015784 | 5 |
| 119 | GO:0007204 | positive regulation of cytosolic calcium ion concentration | 22/465 | 3512/152076 | 0.001447 | 0.016586 | 22 |
| 120 | GO:0051897 | positive regulation of protein kinase B signaling | 23/496 | 3512/152076 | 0.001485 | 0.016917 | 23 |
| 121 | GO:1904953 | Wnt signaling pathway involved in midbrain dopaminergic neuron differentiation | 2/3 | 3512/152076 | 0.001575 | 0.01761 | 2 |
| 122 | GO:0007492 | endoderm development | 6/55 | 3512/152076 | 0.001664 | 0.018275 | 6 |
| 123 | GO:0032963 | collagen metabolic process | 6/55 | 3512/152076 | 0.001664 | 0.018275 | 6 |
| 124 | GO:0048872 | homeostasis of number of cells | 15/276 | 3512/152076 | 0.002143 | 0.023122 | 15 |
| 125 | GO:0003018 | vascular process in circulatory system | 11/171 | 3512/152076 | 0.002234 | 0.023418 | 11 |
| 126 | GO:0007519 | skeletal muscle tissue development | 11/171 | 3512/152076 | 0.002234 | 0.023418 | 11 |
| 127 | GO:0031669 | cellular response to nutrient levels | 11/171 | 3512/152076 | 0.002234 | 0.023418 | 11 |
| 128 | GO:0001764 | neuron migration | 7/78 | 3512/152076 | 0.002205 | 0.023418 | 7 |
| 129 | GO:0048568 | embryonic organ development | 39/1035 | 3512/152076 | 0.002403 | 0.024903 | 39 |
| 130 | GO:0008015 | blood circulation | 40/1081 | 3512/152076 | 0.002916 | 0.030052 | 40 |
| 131 | GO:0032642 | regulation of chemokine production | 8/105 | 3512/152076 | 0.003092 | 0.030897 | 8 |
| 132 | GO:0032602 | chemokine production | 8/105 | 3512/152076 | 0.003092 | 0.030897 | 8 |
| 133 | GO:0002687 | positive regulation of leukocyte migration | 10/153 | 3512/152076 | 0.003098 | 0.030897 | 10 |
| 134 | GO:0002262 | myeloid cell homeostasis | 10/153 | 3512/152076 | 0.003098 | 0.030897 | 10 |
| 135 | GO:0045807 | positive regulation of endocytosis | 8/105 | 3512/152076 | 0.003092 | 0.030897 | 8 |
| 136 | GO:0010863 | positive regulation of phospholipase C activity | 5/45 | 3512/152076 | 0.003715 | 0.035703 | 5 |
| 137 | GO:1900274 | regulation of phospholipase C activity | 5/45 | 3512/152076 | 0.003715 | 0.035703 | 5 |
| 138 | GO:0060425 | lung morphogenesis | 5/45 | 3512/152076 | 0.003715 | 0.035703 | 5 |
| 139 | GO:0042743 | hydrogen peroxide metabolic process | 5/45 | 3512/152076 | 0.003715 | 0.035703 | 5 |
| 140 | GO:0097756 | negative regulation of blood vessel diameter | 5/45 | 3512/152076 | 0.003715 | 0.035703 | 5 |
| 141 | GO:0010977 | negative regulation of neuron projection development | 5/45 | 3512/152076 | 0.003715 | 0.035703 | 5 |
| 142 | GO:0030336 | negative regulation of cell migration | 12/210 | 3512/152076 | 0.00382 | 0.036521 | 12 |
| 143 | GO:0030049 | muscle filament sliding | 6/66 | 3512/152076 | 0.004211 | 0.038842 | 6 |
| 144 | GO:0033275 | actin-myosin filament sliding | 6/66 | 3512/152076 | 0.004211 | 0.038842 | 6 |
| 145 | GO:0034612 | response to tumor necrosis factor | 16/325 | 3512/152076 | 0.004117 | 0.038842 | 16 |
| 146 | GO:0050806 | positive regulation of synaptic transmission | 6/66 | 3512/152076 | 0.004211 | 0.038842 | 6 |
| 147 | GO:0016525 | negative regulation of angiogenesis | 6/66 | 3512/152076 | 0.004211 | 0.038842 | 6 |
| 148 | GO:2000181 | negative regulation of blood vessel morphogenesis | 6/66 | 3512/152076 | 0.004211 | 0.038842 | 6 |
| 149 | GO:1904948 | midbrain dopaminergic neuron differentiation | 3/15 | 3512/152076 | 0.004547 | 0.040925 | 3 |
| 150 | GO:0021915 | neural tube development | 9/136 | 3512/152076 | 0.004507 | 0.040925 | 9 |
| 151 | GO:0051147 | regulation of muscle cell differentiation | 9/136 | 3512/152076 | 0.004507 | 0.040925 | 9 |
| 152 | GO:0062009 | secondary palate development | 3/15 | 3512/152076 | 0.004547 | 0.040925 | 3 |
| 153 | GO:0090050 | positive regulation of cell migration involved in sprouting angiogenesis | 3/15 | 3512/152076 | 0.004547 | 0.040925 | 3 |
| 154 | GO:0034341 | response to interferon-gamma | 11/190 | 3512/152076 | 0.004973 | 0.044109 | 11 |

**Common:**

| No. | ID | Description | GeneRatio | BgRatio | pvalue | p.adjust | count |
| --- | --- | --- | --- | --- | --- | --- | --- |
| 1 | GO:0002009 | morphogenesis of an epithelium | 109/1953 | 3512/152076 | 1.11E-16 | 5.12E-14 | 109 |
| 2 | GO:0050673 | epithelial cell proliferation | 85/1485 | 3512/152076 | 6.59E-14 | 1.52E-11 | 85 |
| 3 | GO:0032496 | response to lipopolysaccharide | 54/861 | 3512/152076 | 8.69E-11 | 1.6E-08 | 54 |
| 4 | GO:0002237 | response to molecule of bacterial origin | 54/861 | 3512/152076 | 8.69E-11 | 1.6E-08 | 54 |
| 5 | GO:0048545 | response to steroid hormone | 56/946 | 3512/152076 | 3.35E-10 | 5.62E-08 | 56 |
| 6 | GO:0061138 | morphogenesis of a branching epithelium | 37/528 | 3512/152076 | 4.19E-09 | 4.55E-07 | 37 |
| 7 | GO:0001763 | morphogenesis of a branching structure | 37/561 | 3512/152076 | 2.04E-08 | 1.59E-06 | 37 |
| 8 | GO:2000027 | regulation of animal organ morphogenesis | 32/465 | 3512/152076 | 6.81E-08 | 3.93E-06 | 32 |
| 9 | GO:0045765 | regulation of angiogenesis | 50/946 | 3512/152076 | 1E-07 | 5.44E-06 | 50 |
| 10 | GO:0030111 | regulation of Wnt signaling pathway | 31/465 | 3512/152076 | 2.17E-07 | 1.08E-05 | 31 |
| 11 | GO:0071222 | cellular response to lipopolysaccharide | 19/210 | 3512/152076 | 5.47E-07 | 2.46E-05 | 19 |
| 12 | GO:0071219 | cellular response to molecule of bacterial origin | 19/210 | 3512/152076 | 5.47E-07 | 2.46E-05 | 19 |
| 13 | GO:0071216 | cellular response to biotic stimulus | 19/210 | 3512/152076 | 5.47E-07 | 2.46E-05 | 19 |
| 14 | GO:0022612 | gland morphogenesis | 21/253 | 3512/152076 | 5.95E-07 | 2.55E-05 | 21 |
| 15 | GO:0060326 | cell chemotaxis | 51/1035 | 3512/152076 | 6.12E-07 | 2.57E-05 | 51 |
| 16 | GO:0045785 | positive regulation of cell adhesion | 64/1485 | 3512/152076 | 2.43E-06 | 8.78E-05 | 64 |
| 17 | GO:0030595 | leukocyte chemotaxis | 30/496 | 3512/152076 | 2.48E-06 | 8.78E-05 | 30 |
| 18 | GO:1901342 | regulation of vasculature development | 52/1128 | 3512/152076 | 3.26E-06 | 0.000111 | 52 |
| 19 | GO:0048754 | branching morphogenesis of an epithelial tube | 25/378 | 3512/152076 | 3.53E-06 | 0.000118 | 25 |
| 20 | GO:0001654 | eye development | 27/435 | 3512/152076 | 4.79E-06 | 0.00014 | 27 |
| 21 | GO:0150063 | visual system development | 27/435 | 3512/152076 | 4.79E-06 | 0.00014 | 27 |
| 22 | GO:0048880 | sensory system development | 27/435 | 3512/152076 | 4.79E-06 | 0.00014 | 27 |
| 23 | GO:0050678 | regulation of epithelial cell proliferation | 49/1081 | 3512/152076 | 9.54E-06 | 0.000252 | 49 |
| 24 | GO:1902107 | positive regulation of leukocyte differentiation | 17/210 | 3512/152076 | 9.42E-06 | 0.000252 | 17 |
| 25 | GO:0060562 | epithelial tube morphogenesis | 43/903 | 3512/152076 | 1.02E-05 | 0.000257 | 43 |
| 26 | GO:0007517 | muscle organ development | 56/1326 | 3512/152076 | 1.75E-05 | 0.000436 | 56 |
| 27 | GO:0016055 | Wnt signaling pathway | 43/946 | 3512/152076 | 3.02E-05 | 0.00068 | 43 |
| 28 | GO:0198738 | cell-cell signaling by wnt | 43/946 | 3512/152076 | 3.02E-05 | 0.00068 | 43 |
| 29 | GO:0071621 | granulocyte chemotaxis | 16/231 | 3512/152076 | 0.000111 | 0.002001 | 16 |
| 30 | GO:1902105 | regulation of leukocyte differentiation | 25/465 | 3512/152076 | 0.000109 | 0.002001 | 25 |
| 31 | GO:0048145 | regulation of fibroblast proliferation | 11/120 | 3512/152076 | 0.000114 | 0.002017 | 11 |
| 32 | GO:0048144 | fibroblast proliferation | 11/120 | 3512/152076 | 0.000114 | 0.002017 | 11 |
| 33 | GO:0019932 | second-messenger-mediated signaling | 29/595 | 3512/152076 | 0.000176 | 0.003003 | 29 |
| 34 | GO:0048565 | digestive tract development | 20/351 | 3512/152076 | 0.000239 | 0.003882 | 20 |
| 35 | GO:0021700 | developmental maturation | 31/666 | 3512/152076 | 0.00024 | 0.003882 | 31 |
| 36 | GO:0048762 | mesenchymal cell differentiation | 21/378 | 3512/152076 | 0.000239 | 0.003882 | 21 |
| 37 | GO:0008544 | epidermis development | 31/666 | 3512/152076 | 0.00024 | 0.003882 | 31 |
| 38 | GO:0010632 | regulation of epithelial cell migration | 20/351 | 3512/152076 | 0.000239 | 0.003882 | 20 |
| 39 | GO:0071383 | cellular response to steroid hormone stimulus | 16/253 | 3512/152076 | 0.00031 | 0.004656 | 16 |
| 40 | GO:0060485 | mesenchyme development | 28/595 | 3512/152076 | 0.000392 | 0.005697 | 28 |
| 41 | GO:0035265 | organ growth | 26/561 | 3512/152076 | 0.000771 | 0.010019 | 26 |
| 42 | GO:0055123 | digestive system development | 21/435 | 3512/152076 | 0.001435 | 0.016545 | 21 |
| 43 | GO:0043491 | protein kinase B signaling | 32/780 | 3512/152076 | 0.001566 | 0.01761 | 32 |
| 44 | GO:0071695 | anatomical structure maturation | 18/351 | 3512/152076 | 0.001575 | 0.01761 | 18 |
| 45 | GO:0090130 | tissue migration | 25/561 | 3512/152076 | 0.001639 | 0.018218 | 25 |
| 46 | GO:0002573 | myeloid leukocyte differentiation | 17/325 | 3512/152076 | 0.0017 | 0.018555 | 17 |
| 47 | GO:0051896 | regulation of protein kinase B signaling | 27/630 | 3512/152076 | 0.001909 | 0.020713 | 27 |
| 48 | GO:0030522 | intracellular receptor signaling pathway | 11/171 | 3512/152076 | 0.002234 | 0.023418 | 11 |
| 49 | GO:0033002 | muscle cell proliferation | 29/703 | 3512/152076 | 0.002324 | 0.024226 | 29 |
| 50 | GO:0045995 | regulation of embryonic development | 10/153 | 3512/152076 | 0.003098 | 0.030897 | 10 |
| 51 | GO:0048562 | embryonic organ morphogenesis | 18/378 | 3512/152076 | 0.003469 | 0.034411 | 18 |
| 52 | GO:0044706 | multi-multicellular organism process | 16/325 | 3512/152076 | 0.004117 | 0.038842 | 16 |
| 53 | GO:0048660 | regulation of smooth muscle cell proliferation | 15/300 | 3512/152076 | 0.004656 | 0.041499 | 15 |
| 54 | GO:0048659 | smooth muscle cell proliferation | 15/300 | 3512/152076 | 0.004656 | 0.041499 | 15 |
| 55 | GO:0002761 | regulation of myeloid leukocyte differentiation | 7/91 | 3512/152076 | 0.005223 | 0.046104 | 7 |
