# Supplementary material for: RNA-Seq-Based Breast Cancer Subtypes Classification Using Machine Learning Approaches
Source: Comput Intell Neurosci. 2020 Oct 29;2020:4737969. doi: 10.1155/2020/4737969 (PMC7644310; doi:10.1155/2020/4737969)
Supplement: Supplementary Materials — Figure S1: heatmap for Her2 and non Her2 groups. The left group 1 represents the Her2 group and the right group 2 denotes the non-Her2 group. Figure S2: heatmap for LumA and non-LumA groups. The left group 1 represents the LumA group and the right group 2 denotes the non-LumA group. Figure S3: heatmap for LumB and non-LumB groups. The left group 1 represents the LumB group and the right group 2 denotes the non-LumB group. Figure S4: heatmap for Normal-like and non-Normal-like groups. The left group 1 represents the Normal-like group and the right group 2 denotes the non-Normal-like group. S1 File: the detailed information of weighted DEGs for classification. S2 File: the detailed information of weighted DEGs for GO enrichment analysis. S3 File: the detailed enriched GO terms results for Basal-like subtype. S4 File: the detailed enriched GO terms results for Her2 subtype. S5 File: the detailed enriched GO terms results for LumA subtype. S6 File: the detailed enriched GO terms results for LumB subtype. S7 File: the detailed enriched GO terms results for Normal-like subtype. [file 4737969.f1.zip › supplementary materials/S7 File.docx]

**S7 File:** The detailed enriched GO terms results for Normal-like subtype.

**Control group:**

| No. | ID | Description | GeneRatio | BgRatio | pvalue | p.adjust | count |
| --- | --- | --- | --- | --- | --- | --- | --- |
| 1 | GO:0002009 | morphogenesis of an epithelium | 341/2278 | 18111/236328 | 0 | 0 | 341 |
| 2 | GO:1901342 | regulation of vasculature development | 279/1830 | 18111/236328 | 0 | 0 | 279 |
| 3 | GO:0043062 | extracellular structure organization | 238/1596 | 18111/236328 | 0 | 0 | 238 |
| 4 | GO:0045765 | regulation of angiogenesis | 218/1326 | 18111/236328 | 0 | 0 | 218 |
| 5 | GO:0042063 | gliogenesis | 142/820 | 18111/236328 | 0 | 0 | 142 |
| 6 | GO:0022612 | gland morphogenesis | 74/325 | 18111/236328 | 0 | 0 | 74 |
| 7 | GO:0048545 | response to steroid hormone | 168/1128 | 18111/236328 | 2.22E-16 | 6.49E-14 | 168 |
| 8 | GO:0001101 | response to acid chemical | 228/1711 | 18111/236328 | 4.44E-16 | 1.14E-13 | 228 |
| 9 | GO:0060541 | respiratory system development | 66/300 | 18111/236328 | 6.11E-15 | 1.39E-12 | 66 |
| 10 | GO:0032652 | regulation of interleukin-1 production | 28/66 | 18111/236328 | 1.07E-14 | 2.18E-12 | 28 |
| 11 | GO:0061448 | connective tissue development | 123/780 | 18111/236328 | 2.89E-14 | 4.92E-12 | 123 |
| 12 | GO:1904018 | positive regulation of vasculature development | 110/666 | 18111/236328 | 2.88E-14 | 4.92E-12 | 110 |
| 13 | GO:0048732 | gland development | 282/2346 | 18111/236328 | 7.29E-14 | 1.15E-11 | 282 |
| 14 | GO:0050707 | regulation of cytokine secretion | 67/325 | 18111/236328 | 1.04E-13 | 1.52E-11 | 67 |
| 15 | GO:0042110 | T cell activation | 295/2556 | 18111/236328 | 2.31E-12 | 3.15E-10 | 295 |
| 16 | GO:0032612 | interleukin-1 production | 30/91 | 18111/236328 | 3.11E-12 | 3.98E-10 | 30 |
| 17 | GO:0071346 | cellular response to interferon-gamma | 91/561 | 18111/236328 | 1.21E-11 | 1.46E-09 | 91 |
| 18 | GO:0048568 | embryonic organ development | 152/1128 | 18111/236328 | 1.28E-11 | 1.46E-09 | 152 |
| 19 | GO:0050663 | cytokine secretion | 83/496 | 18111/236328 | 1.88E-11 | 2.03E-09 | 83 |
| 20 | GO:0007162 | negative regulation of cell adhesion | 75/435 | 18111/236328 | 4.04E-11 | 4.14E-09 | 75 |
| 21 | GO:0002064 | epithelial cell development | 82/496 | 18111/236328 | 4.6E-11 | 4.48E-09 | 82 |
| 22 | GO:0045785 | positive regulation of cell adhesion | 262/2278 | 18111/236328 | 5.28E-11 | 4.71E-09 | 262 |
| 23 | GO:0050900 | leukocyte migration | 225/1891 | 18111/236328 | 5.67E-11 | 4.71E-09 | 225 |
| 24 | GO:0001763 | morphogenesis of a branching structure | 93/595 | 18111/236328 | 5.75E-11 | 4.71E-09 | 93 |
| 25 | GO:2000027 | regulation of animal organ morphogenesis | 93/595 | 18111/236328 | 5.75E-11 | 4.71E-09 | 93 |
| 26 | GO:0051249 | regulation of lymphocyte activation | 241/2080 | 18111/236328 | 1.53E-10 | 1.16E-08 | 241 |
| 27 | GO:0061138 | morphogenesis of a branching epithelium | 88/561 | 18111/236328 | 1.53E-10 | 1.16E-08 | 88 |
| 28 | GO:0034612 | response to tumor necrosis factor | 84/528 | 18111/236328 | 1.93E-10 | 1.41E-08 | 84 |
| 29 | GO:0050727 | regulation of inflammatory response | 199/1653 | 18111/236328 | 2.66E-10 | 1.88E-08 | 199 |
| 30 | GO:0090130 | tissue migration | 137/1035 | 18111/236328 | 4.12E-10 | 2.81E-08 | 137 |
| 31 | GO:0032496 | response to lipopolysaccharide | 187/1540 | 18111/236328 | 4.57E-10 | 2.92E-08 | 187 |
| 32 | GO:0002237 | response to molecule of bacterial origin | 187/1540 | 18111/236328 | 4.57E-10 | 2.92E-08 | 187 |
| 33 | GO:0032102 | negative regulation of response to external stimulus | 106/741 | 18111/236328 | 5.41E-10 | 3.36E-08 | 106 |
| 34 | GO:0050866 | negative regulation of cell activation | 50/253 | 18111/236328 | 5.88E-10 | 3.54E-08 | 50 |
| 35 | GO:0010632 | regulation of epithelial cell migration | 97/666 | 18111/236328 | 1.1E-09 | 6.42E-08 | 97 |
| 36 | GO:0034341 | response to interferon-gamma | 93/630 | 18111/236328 | 1.19E-09 | 6.78E-08 | 93 |
| 37 | GO:0048660 | regulation of smooth muscle cell proliferation | 78/496 | 18111/236328 | 1.42E-09 | 7.67E-08 | 78 |
| 38 | GO:0048659 | smooth muscle cell proliferation | 78/496 | 18111/236328 | 1.42E-09 | 7.67E-08 | 78 |
| 39 | GO:0048562 | embryonic organ morphogenesis | 61/351 | 18111/236328 | 1.8E-09 | 9.44E-08 | 61 |
| 40 | GO:0030324 | lung development | 46/231 | 18111/236328 | 2.16E-09 | 1.08E-07 | 46 |
| 41 | GO:0030323 | respiratory tube development | 46/231 | 18111/236328 | 2.16E-09 | 1.08E-07 | 46 |
| 42 | GO:0050708 | regulation of protein secretion | 138/1081 | 18111/236328 | 3.72E-09 | 1.81E-07 | 138 |
| 43 | GO:0046677 | response to antibiotic | 162/1326 | 18111/236328 | 4.11E-09 | 1.96E-07 | 162 |
| 44 | GO:0070661 | leukocyte proliferation | 147/1176 | 18111/236328 | 4.78E-09 | 2.22E-07 | 147 |
| 45 | GO:0030595 | leukocyte chemotaxis | 87/595 | 18111/236328 | 6.27E-09 | 2.85E-07 | 87 |
| 46 | GO:0060326 | cell chemotaxis | 181/1540 | 18111/236328 | 9.56E-09 | 4.25E-07 | 181 |
| 47 | GO:0071222 | cellular response to lipopolysaccharide | 79/528 | 18111/236328 | 1.09E-08 | 4.65E-07 | 79 |
| 48 | GO:0071219 | cellular response to molecule of bacterial origin | 79/528 | 18111/236328 | 1.09E-08 | 4.65E-07 | 79 |
| 49 | GO:0043491 | protein kinase B signaling | 122/946 | 18111/236328 | 1.6E-08 | 6.67E-07 | 122 |
| 50 | GO:0071229 | cellular response to acid chemical | 101/741 | 18111/236328 | 1.69E-08 | 6.93E-07 | 101 |
| 51 | GO:0097305 | response to alcohol | 121/946 | 18111/236328 | 2.88E-08 | 1.16E-06 | 121 |
| 52 | GO:0030217 | T cell differentiation | 116/903 | 18111/236328 | 4.34E-08 | 1.69E-06 | 116 |
| 53 | GO:0048863 | stem cell differentiation | 67/435 | 18111/236328 | 4.38E-08 | 1.69E-06 | 67 |
| 54 | GO:0045766 | positive regulation of angiogenesis | 70/465 | 18111/236328 | 5.59E-08 | 2.12E-06 | 70 |
| 55 | GO:0030010 | establishment of cell polarity | 20/66 | 18111/236328 | 6.14E-08 | 2.28E-06 | 20 |
| 56 | GO:0030098 | lymphocyte differentiation | 177/1540 | 18111/236328 | 6.43E-08 | 2.35E-06 | 177 |
| 57 | GO:0033002 | muscle cell proliferation | 111/861 | 18111/236328 | 6.91E-08 | 2.48E-06 | 111 |
| 58 | GO:0071216 | cellular response to biotic stimulus | 80/561 | 18111/236328 | 7.28E-08 | 2.53E-06 | 80 |
| 59 | GO:0042098 | T cell proliferation | 60/378 | 18111/236328 | 7.3E-08 | 2.53E-06 | 60 |
| 60 | GO:0060537 | muscle tissue development | 161/1378 | 18111/236328 | 8.72E-08 | 2.97E-06 | 161 |
| 61 | GO:0007596 | blood coagulation | 146/1225 | 18111/236328 | 1.04E-07 | 3.43E-06 | 146 |
| 62 | GO:0050817 | coagulation | 146/1225 | 18111/236328 | 1.04E-07 | 3.43E-06 | 146 |
| 63 | GO:0010631 | epithelial cell migration | 123/990 | 18111/236328 | 1.11E-07 | 3.56E-06 | 123 |
| 64 | GO:0090132 | epithelium migration | 123/990 | 18111/236328 | 1.11E-07 | 3.56E-06 | 123 |
| 65 | GO:1902105 | regulation of leukocyte differentiation | 106/820 | 18111/236328 | 1.17E-07 | 3.63E-06 | 106 |
| 66 | GO:0001701 | in utero embryonic development | 106/820 | 18111/236328 | 1.17E-07 | 3.63E-06 | 106 |
| 67 | GO:0030593 | neutrophil chemotaxis | 30/136 | 18111/236328 | 1.2E-07 | 3.68E-06 | 30 |
| 68 | GO:0007163 | establishment or maintenance of cell polarity | 37/190 | 18111/236328 | 1.37E-07 | 4.13E-06 | 37 |
| 69 | GO:0007599 | hemostasis | 150/1275 | 18111/236328 | 1.54E-07 | 4.51E-06 | 150 |
| 70 | GO:0071383 | cellular response to steroid hormone stimulus | 42/231 | 18111/236328 | 1.53E-07 | 4.51E-06 | 42 |
| 71 | GO:0022407 | regulation of cell-cell adhesion | 225/2080 | 18111/236328 | 1.57E-07 | 4.53E-06 | 225 |
| 72 | GO:0032651 | regulation of interleukin-1 beta production | 14/36 | 18111/236328 | 1.79E-07 | 5.1E-06 | 14 |
| 73 | GO:1902903 | regulation of supramolecular fiber organization | 65/435 | 18111/236328 | 2.12E-07 | 5.96E-06 | 65 |
| 74 | GO:0050673 | epithelial cell proliferation | 224/2080 | 18111/236328 | 2.33E-07 | 6.44E-06 | 224 |
| 75 | GO:0043407 | negative regulation of MAP kinase activity | 25/105 | 18111/236328 | 2.83E-07 | 7.71E-06 | 25 |
| 76 | GO:0048546 | digestive tract morphogenesis | 9/15 | 18111/236328 | 2.97E-07 | 8E-06 | 9 |
| 77 | GO:0002285 | lymphocyte activation involved in immune response | 29/136 | 18111/236328 | 4.14E-07 | 1.1E-05 | 29 |
| 78 | GO:0150063 | visual system development | 81/595 | 18111/236328 | 4.26E-07 | 1.1E-05 | 81 |
| 79 | GO:0048880 | sensory system development | 81/595 | 18111/236328 | 4.26E-07 | 1.1E-05 | 81 |
| 80 | GO:0045619 | regulation of lymphocyte differentiation | 49/300 | 18111/236328 | 4.56E-07 | 1.15E-05 | 49 |
| 81 | GO:0071356 | cellular response to tumor necrosis factor | 64/435 | 18111/236328 | 4.56E-07 | 1.15E-05 | 64 |
| 82 | GO:0002791 | regulation of peptide secretion | 138/1176 | 18111/236328 | 5.37E-07 | 1.34E-05 | 138 |
| 83 | GO:0009612 | response to mechanical stimulus | 70/496 | 18111/236328 | 6.65E-07 | 1.64E-05 | 70 |
| 84 | GO:0016055 | Wnt signaling pathway | 142/1225 | 18111/236328 | 7.4E-07 | 1.78E-05 | 142 |
| 85 | GO:0198738 | cell-cell signaling by wnt | 142/1225 | 18111/236328 | 7.4E-07 | 1.78E-05 | 142 |
| 86 | GO:0000302 | response to reactive oxygen species | 115/946 | 18111/236328 | 8.26E-07 | 1.94E-05 | 115 |
| 87 | GO:0051216 | cartilage development | 80/595 | 18111/236328 | 8.22E-07 | 1.94E-05 | 80 |
| 88 | GO:0001655 | urogenital system development | 128/1081 | 18111/236328 | 8.43E-07 | 1.96E-05 | 128 |
| 89 | GO:0006469 | negative regulation of protein kinase activity | 54/351 | 18111/236328 | 8.58E-07 | 1.97E-05 | 54 |
| 90 | GO:0007517 | muscle organ development | 161/1431 | 18111/236328 | 8.77E-07 | 2E-05 | 161 |
| 91 | GO:0036293 | response to decreased oxygen levels | 119/990 | 18111/236328 | 9.51E-07 | 2.14E-05 | 119 |
| 92 | GO:0002062 | chondrocyte differentiation | 40/231 | 18111/236328 | 1.1E-06 | 2.41E-05 | 40 |
| 93 | GO:0002040 | sprouting angiogenesis | 40/231 | 18111/236328 | 1.1E-06 | 2.41E-05 | 40 |
| 94 | GO:0002695 | negative regulation of leukocyte activation | 26/120 | 18111/236328 | 1.16E-06 | 2.53E-05 | 26 |
| 95 | GO:0070098 | chemokine-mediated signaling pathway | 20/78 | 18111/236328 | 1.22E-06 | 2.62E-05 | 20 |
| 96 | GO:0042088 | T-helper 1 type immune response | 13/36 | 18111/236328 | 1.34E-06 | 2.85E-05 | 13 |
| 97 | GO:0031100 | animal organ regeneration | 28/136 | 18111/236328 | 1.36E-06 | 2.88E-05 | 28 |
| 98 | GO:0007568 | aging | 114/946 | 18111/236328 | 1.4E-06 | 2.92E-05 | 114 |
| 99 | GO:0014706 | striated muscle tissue development | 150/1326 | 18111/236328 | 1.49E-06 | 3.08E-05 | 150 |
| 100 | GO:0001819 | positive regulation of cytokine production | 180/1653 | 18111/236328 | 1.67E-06 | 3.43E-05 | 180 |
| 101 | GO:0060562 | epithelial tube morphogenesis | 105/861 | 18111/236328 | 2.07E-06 | 4.2E-05 | 105 |
| 102 | GO:0001666 | response to hypoxia | 109/903 | 18111/236328 | 2.16E-06 | 4.34E-05 | 109 |
| 103 | GO:1903034 | regulation of response to wounding | 65/465 | 18111/236328 | 2.25E-06 | 4.46E-05 | 65 |
| 104 | GO:0110110 | positive regulation of animal organ morphogenesis | 32/171 | 18111/236328 | 2.28E-06 | 4.48E-05 | 32 |
| 105 | GO:0032611 | interleukin-1 beta production | 16/55 | 18111/236328 | 2.3E-06 | 4.49E-05 | 16 |
| 106 | GO:0032970 | regulation of actin filament-based process | 89/703 | 18111/236328 | 2.66E-06 | 5.14E-05 | 89 |
| 107 | GO:0070482 | response to oxygen levels | 130/1128 | 18111/236328 | 2.8E-06 | 5.36E-05 | 130 |
| 108 | GO:0071559 | response to transforming growth factor beta | 78/595 | 18111/236328 | 2.93E-06 | 5.5E-05 | 78 |
| 109 | GO:0032956 | regulation of actin cytoskeleton organization | 78/595 | 18111/236328 | 2.93E-06 | 5.5E-05 | 78 |
| 110 | GO:0043010 | camera-type eye development | 71/528 | 18111/236328 | 3.24E-06 | 6.03E-05 | 71 |
| 111 | GO:0031099 | regeneration | 58/406 | 18111/236328 | 3.87E-06 | 7.07E-05 | 58 |
| 112 | GO:0035265 | organ growth | 58/406 | 18111/236328 | 3.87E-06 | 7.07E-05 | 58 |
| 113 | GO:0045165 | cell fate commitment | 81/630 | 18111/236328 | 4.04E-06 | 7.32E-05 | 81 |
| 114 | GO:1990868 | response to chemokine | 21/91 | 18111/236328 | 4.18E-06 | 7.44E-05 | 21 |
| 115 | GO:1990869 | cellular response to chemokine | 21/91 | 18111/236328 | 4.18E-06 | 7.44E-05 | 21 |
| 116 | GO:0043200 | response to amino acid | 27/136 | 18111/236328 | 4.31E-06 | 7.6E-05 | 27 |
| 117 | GO:0045834 | positive regulation of lipid metabolic process | 41/253 | 18111/236328 | 4.56E-06 | 7.98E-05 | 41 |
| 118 | GO:0001667 | ameboidal-type cell migration | 162/1485 | 18111/236328 | 4.78E-06 | 8.3E-05 | 162 |
| 119 | GO:0022409 | positive regulation of cell-cell adhesion | 152/1378 | 18111/236328 | 5.09E-06 | 8.55E-05 | 152 |
| 120 | GO:0016525 | negative regulation of angiogenesis | 29/153 | 18111/236328 | 5.09E-06 | 8.55E-05 | 29 |
| 121 | GO:2000181 | negative regulation of blood vessel morphogenesis | 29/153 | 18111/236328 | 5.09E-06 | 8.55E-05 | 29 |
| 122 | GO:1903531 | negative regulation of secretion by cell | 29/153 | 18111/236328 | 5.09E-06 | 8.55E-05 | 29 |
| 123 | GO:0051897 | positive regulation of protein kinase B signaling | 77/595 | 18111/236328 | 5.41E-06 | 9E-05 | 77 |
| 124 | GO:0001822 | kidney development | 84/666 | 18111/236328 | 5.72E-06 | 9.43E-05 | 84 |
| 125 | GO:0043409 | negative regulation of MAPK cascade | 46/300 | 18111/236328 | 5.76E-06 | 9.43E-05 | 46 |
| 126 | GO:0001933 | negative regulation of protein phosphorylation | 124/1081 | 18111/236328 | 5.83E-06 | 9.47E-05 | 124 |
| 127 | GO:0070302 | regulation of stress-activated protein kinase signaling cascade | 70/528 | 18111/236328 | 6.17E-06 | 9.94E-05 | 70 |
| 128 | GO:0016049 | cell growth | 95/780 | 18111/236328 | 6.41E-06 | 0.000102 | 95 |
| 129 | GO:0003158 | endothelium development | 38/231 | 18111/236328 | 6.97E-06 | 0.000111 | 38 |
| 130 | GO:0051896 | regulation of protein kinase B signaling | 91/741 | 18111/236328 | 7.12E-06 | 0.000112 | 91 |
| 131 | GO:0071560 | cellular response to transforming growth factor beta stimulus | 73/561 | 18111/236328 | 7.67E-06 | 0.00012 | 73 |
| 132 | GO:0061041 | regulation of wound healing | 54/378 | 18111/236328 | 8.12E-06 | 0.000124 | 54 |
| 133 | GO:0010634 | positive regulation of epithelial cell migration | 54/378 | 18111/236328 | 8.12E-06 | 0.000124 | 54 |
| 134 | GO:0033673 | negative regulation of kinase activity | 54/378 | 18111/236328 | 8.12E-06 | 0.000124 | 54 |
| 135 | GO:0030111 | regulation of Wnt signaling pathway | 66/496 | 18111/236328 | 9.85E-06 | 0.000149 | 66 |
| 136 | GO:0055123 | digestive system development | 40/253 | 18111/236328 | 1.08E-05 | 0.000161 | 40 |
| 137 | GO:0110053 | regulation of actin filament organization | 40/253 | 18111/236328 | 1.08E-05 | 0.000161 | 40 |
| 138 | GO:0002825 | regulation of T-helper 1 type immune response | 9/21 | 18111/236328 | 1.14E-05 | 0.000169 | 9 |
| 139 | GO:0042129 | regulation of T cell proliferation | 42/276 | 18111/236328 | 1.71E-05 | 0.000252 | 42 |
| 140 | GO:0007015 | actin filament organization | 75/595 | 18111/236328 | 1.77E-05 | 0.000259 | 75 |
| 141 | GO:0031098 | stress-activated protein kinase signaling cascade | 89/741 | 18111/236328 | 2.07E-05 | 0.0003 | 89 |
| 142 | GO:0060560 | developmental growth involved in morphogenesis | 39/253 | 18111/236328 | 2.49E-05 | 0.000358 | 39 |
| 143 | GO:0007204 | positive regulation of cytosolic calcium ion concentration | 125/1128 | 18111/236328 | 2.65E-05 | 0.000374 | 125 |
| 144 | GO:0042326 | negative regulation of phosphorylation | 125/1128 | 18111/236328 | 2.65E-05 | 0.000374 | 125 |
| 145 | GO:0007389 | pattern specification process | 104/903 | 18111/236328 | 2.62E-05 | 0.000374 | 104 |
| 146 | GO:0050863 | regulation of T cell activation | 143/1326 | 18111/236328 | 2.87E-05 | 0.000402 | 143 |
| 147 | GO:0051781 | positive regulation of cell division | 16/66 | 18111/236328 | 2.94E-05 | 0.00041 | 16 |
| 148 | GO:0007249 | I-kappaB kinase/NF-kappaB signaling | 55/406 | 18111/236328 | 3.11E-05 | 0.00043 | 55 |
| 149 | GO:0034330 | cell junction organization | 61/465 | 18111/236328 | 3.17E-05 | 0.000433 | 61 |
| 150 | GO:0002460 | adaptive immune response based on somatic recombination of immune receptors built from immunoglobulin superfamily domains | 61/465 | 18111/236328 | 3.17E-05 | 0.000433 | 61 |
| 151 | GO:0002573 | myeloid leukocyte differentiation | 77/630 | 18111/236328 | 3.97E-05 | 0.000538 | 77 |
| 152 | GO:0060401 | cytosolic calcium ion transport | 27/153 | 18111/236328 | 3.99E-05 | 0.000538 | 27 |
| 153 | GO:0042303 | molting cycle | 21/105 | 18111/236328 | 4.25E-05 | 0.000565 | 21 |
| 154 | GO:0042633 | hair cycle | 21/105 | 18111/236328 | 4.25E-05 | 0.000565 | 21 |
| 155 | GO:0072593 | reactive oxygen species metabolic process | 70/561 | 18111/236328 | 4.52E-05 | 0.000597 | 70 |
| 156 | GO:0003179 | heart valve morphogenesis | 19/91 | 18111/236328 | 5.22E-05 | 0.000681 | 19 |
| 157 | GO:0003170 | heart valve development | 19/91 | 18111/236328 | 5.22E-05 | 0.000681 | 19 |
| 158 | GO:0018108 | peptidyl-tyrosine phosphorylation | 186/1830 | 18111/236328 | 6.36E-05 | 0.000819 | 186 |
| 159 | GO:0018212 | peptidyl-tyrosine modification | 186/1830 | 18111/236328 | 6.36E-05 | 0.000819 | 186 |
| 160 | GO:0050671 | positive regulation of lymphocyte proliferation | 51/378 | 18111/236328 | 6.51E-05 | 0.000828 | 51 |
| 161 | GO:0032946 | positive regulation of mononuclear cell proliferation | 51/378 | 18111/236328 | 6.51E-05 | 0.000828 | 51 |
| 162 | GO:0042102 | positive regulation of T cell proliferation | 33/210 | 18111/236328 | 6.81E-05 | 0.00086 | 33 |
| 163 | GO:0007178 | transmembrane receptor protein serine/threonine kinase signaling pathway | 94/820 | 18111/236328 | 7.27E-05 | 0.000913 | 94 |
| 164 | GO:0018105 | peptidyl-serine phosphorylation | 48/351 | 18111/236328 | 7.5E-05 | 0.000936 | 48 |
| 165 | GO:0070555 | response to interleukin-1 | 40/276 | 18111/236328 | 8.09E-05 | 0.001004 | 40 |
| 166 | GO:0048771 | tissue remodeling | 45/325 | 18111/236328 | 9.19E-05 | 0.001133 | 45 |
| 167 | GO:0050678 | regulation of epithelial cell proliferation | 174/1711 | 18111/236328 | 0.000104 | 0.00127 | 174 |
| 168 | GO:0042093 | T-helper cell differentiation | 12/45 | 18111/236328 | 0.000107 | 0.001285 | 12 |
| 169 | GO:0002287 | alpha-beta T cell activation involved in immune response | 12/45 | 18111/236328 | 0.000107 | 0.001285 | 12 |
| 170 | GO:0002292 | T cell differentiation involved in immune response | 12/45 | 18111/236328 | 0.000107 | 0.001285 | 12 |
| 171 | GO:0018209 | peptidyl-serine modification | 56/435 | 18111/236328 | 0.000107 | 0.001286 | 56 |
| 172 | GO:0032872 | regulation of stress-activated MAPK cascade | 62/496 | 18111/236328 | 0.000112 | 0.00132 | 62 |
| 173 | GO:0044706 | multi-multicellular organism process | 53/406 | 18111/236328 | 0.000113 | 0.00132 | 53 |
| 174 | GO:0048013 | ephrin receptor signaling pathway | 15/66 | 18111/236328 | 0.000114 | 0.00132 | 15 |
| 175 | GO:0001942 | hair follicle development | 15/66 | 18111/236328 | 0.000114 | 0.00132 | 15 |
| 176 | GO:0022404 | molting cycle process | 15/66 | 18111/236328 | 0.000114 | 0.00132 | 15 |
| 177 | GO:0022405 | hair cycle process | 15/66 | 18111/236328 | 0.000114 | 0.00132 | 15 |
| 178 | GO:0016202 | regulation of striated muscle tissue development | 42/300 | 18111/236328 | 0.00012 | 0.001353 | 42 |
| 179 | GO:1901861 | regulation of muscle tissue development | 42/300 | 18111/236328 | 0.00012 | 0.001353 | 42 |
| 180 | GO:0048634 | regulation of muscle organ development | 42/300 | 18111/236328 | 0.00012 | 0.001353 | 42 |
| 181 | GO:0007179 | transforming growth factor beta receptor signaling pathway | 37/253 | 18111/236328 | 0.000121 | 0.001353 | 37 |
| 182 | GO:0031348 | negative regulation of defense response | 37/253 | 18111/236328 | 0.000121 | 0.001353 | 37 |
| 183 | GO:0060348 | bone development | 37/253 | 18111/236328 | 0.000121 | 0.001353 | 37 |
| 184 | GO:0045580 | regulation of T cell differentiation | 30/190 | 18111/236328 | 0.00013 | 0.001444 | 30 |
| 185 | GO:0070663 | regulation of leukocyte proliferation | 78/666 | 18111/236328 | 0.000144 | 0.001588 | 78 |
| 186 | GO:0070372 | regulation of ERK1 and ERK2 cascade | 134/1275 | 18111/236328 | 0.000154 | 0.00169 | 134 |
| 187 | GO:0001649 | osteoblast differentiation | 71/595 | 18111/236328 | 0.000159 | 0.00174 | 71 |
| 188 | GO:0050710 | negative regulation of cytokine secretion | 9/28 | 18111/236328 | 0.000163 | 0.00177 | 9 |
| 189 | GO:0001937 | negative regulation of endothelial cell proliferation | 9/28 | 18111/236328 | 0.000163 | 0.00177 | 9 |
| 190 | GO:0043122 | regulation of I-kappaB kinase/NF-kappaB signaling | 39/276 | 18111/236328 | 0.000169 | 0.001819 | 39 |
| 191 | GO:0048754 | branching morphogenesis of an epithelial tube | 44/325 | 18111/236328 | 0.000182 | 0.001949 | 44 |
| 192 | GO:0070542 | response to fatty acid | 34/231 | 18111/236328 | 0.000196 | 0.002085 | 34 |
| 193 | GO:0051348 | negative regulation of transferase activity | 61/496 | 18111/236328 | 0.000197 | 0.002085 | 61 |
| 194 | GO:0070665 | positive regulation of leukocyte proliferation | 52/406 | 18111/236328 | 0.000209 | 0.002202 | 52 |
| 195 | GO:0050878 | regulation of body fluid levels | 221/2278 | 18111/236328 | 0.00022 | 0.00231 | 221 |
| 196 | GO:1901654 | response to ketone | 84/741 | 18111/236328 | 0.000241 | 0.002517 | 84 |
| 197 | GO:0098773 | skin epidermis development | 16/78 | 18111/236328 | 0.000245 | 0.002549 | 16 |
| 198 | GO:1902107 | positive regulation of leukocyte differentiation | 36/253 | 18111/236328 | 0.000255 | 0.002639 | 36 |
| 199 | GO:0043405 | regulation of MAP kinase activity | 137/1326 | 18111/236328 | 0.00027 | 0.002781 | 137 |
| 200 | GO:0006998 | nuclear envelope organization | 10/36 | 18111/236328 | 0.000276 | 0.002823 | 10 |
| 201 | GO:0051403 | stress-activated MAPK cascade | 80/703 | 18111/236328 | 0.000295 | 0.003006 | 80 |
| 202 | GO:0048565 | digestive tract development | 29/190 | 18111/236328 | 0.0003 | 0.003022 | 29 |
| 203 | GO:0031345 | negative regulation of cell projection organization | 29/190 | 18111/236328 | 0.0003 | 0.003022 | 29 |
| 204 | GO:0045471 | response to ethanol | 21/120 | 18111/236328 | 0.000306 | 0.003073 | 21 |
| 205 | GO:1904019 | epithelial cell apoptotic process | 31/210 | 18111/236328 | 0.000346 | 0.003431 | 31 |
| 206 | GO:0001818 | negative regulation of cytokine production | 54/435 | 18111/236328 | 0.000349 | 0.003431 | 54 |
| 207 | GO:0090287 | regulation of cellular response to growth factor stimulus | 54/435 | 18111/236328 | 0.000349 | 0.003431 | 54 |
| 208 | GO:0050680 | negative regulation of epithelial cell proliferation | 31/210 | 18111/236328 | 0.000346 | 0.003431 | 31 |
| 209 | GO:0014812 | muscle cell migration | 19/105 | 18111/236328 | 0.000375 | 0.003674 | 19 |
| 210 | GO:0060485 | mesenchyme development | 83/741 | 18111/236328 | 0.000379 | 0.003699 | 83 |
| 211 | GO:0070371 | ERK1 and ERK2 cascade | 150/1485 | 18111/236328 | 0.000391 | 0.003796 | 150 |
| 212 | GO:0061035 | regulation of cartilage development | 14/66 | 18111/236328 | 0.000409 | 0.003929 | 14 |
| 213 | GO:0030177 | positive regulation of Wnt signaling pathway | 14/66 | 18111/236328 | 0.000409 | 0.003929 | 14 |
| 214 | GO:0051048 | negative regulation of secretion | 33/231 | 18111/236328 | 0.000417 | 0.003993 | 33 |
| 215 | GO:0030856 | regulation of epithelial cell differentiation | 48/378 | 18111/236328 | 0.000431 | 0.004105 | 48 |
| 216 | GO:0090303 | positive regulation of wound healing | 11/45 | 18111/236328 | 0.000468 | 0.00441 | 11 |
| 217 | GO:1903036 | positive regulation of response to wounding | 11/45 | 18111/236328 | 0.000468 | 0.00441 | 11 |
| 218 | GO:0002827 | positive regulation of T-helper 1 type immune response | 5/10 | 18111/236328 | 0.000479 | 0.00446 | 5 |
| 219 | GO:0060231 | mesenchymal to epithelial transition | 5/10 | 18111/236328 | 0.000479 | 0.00446 | 5 |
| 220 | GO:0042554 | superoxide anion generation | 5/10 | 18111/236328 | 0.000479 | 0.00446 | 5 |
| 221 | GO:0051302 | regulation of cell division | 35/253 | 18111/236328 | 0.000523 | 0.004799 | 35 |
| 222 | GO:0031032 | actomyosin structure organization | 35/253 | 18111/236328 | 0.000523 | 0.004799 | 35 |
| 223 | GO:0007565 | female pregnancy | 35/253 | 18111/236328 | 0.000523 | 0.004799 | 35 |
| 224 | GO:0050870 | positive regulation of T cell activation | 97/903 | 18111/236328 | 0.000555 | 0.005072 | 97 |
| 225 | GO:0050730 | regulation of peptidyl-tyrosine phosphorylation | 75/666 | 18111/236328 | 0.000599 | 0.005451 | 75 |
| 226 | GO:0051051 | negative regulation of transport | 89/820 | 18111/236328 | 0.00066 | 0.005975 | 89 |
| 227 | GO:0050715 | positive regulation of cytokine secretion | 22/136 | 18111/236328 | 0.000686 | 0.006161 | 22 |
| 228 | GO:0060402 | calcium ion transport into cytosol | 22/136 | 18111/236328 | 0.000686 | 0.006161 | 22 |
| 229 | GO:1901343 | negative regulation of vasculature development | 30/210 | 18111/236328 | 0.00074 | 0.006617 | 30 |
| 230 | GO:0034694 | response to prostaglandin | 15/78 | 18111/236328 | 0.000775 | 0.006783 | 15 |
| 231 | GO:0048708 | astrocyte differentiation | 15/78 | 18111/236328 | 0.000775 | 0.006783 | 15 |
| 232 | GO:0045582 | positive regulation of T cell differentiation | 15/78 | 18111/236328 | 0.000775 | 0.006783 | 15 |
| 233 | GO:0070613 | regulation of protein processing | 15/78 | 18111/236328 | 0.000775 | 0.006783 | 15 |
| 234 | GO:1903317 | regulation of protein maturation | 15/78 | 18111/236328 | 0.000775 | 0.006783 | 15 |
| 235 | GO:0019216 | regulation of lipid metabolic process | 108/1035 | 18111/236328 | 0.000789 | 0.006827 | 108 |
| 236 | GO:0072080 | nephron tubule development | 20/120 | 18111/236328 | 0.0008 | 0.006827 | 20 |
| 237 | GO:0061326 | renal tubule development | 20/120 | 18111/236328 | 0.0008 | 0.006827 | 20 |
| 238 | GO:0035850 | epithelial cell differentiation involved in kidney development | 12/55 | 18111/236328 | 0.000794 | 0.006827 | 12 |
| 239 | GO:0061005 | cell differentiation involved in kidney development | 12/55 | 18111/236328 | 0.000794 | 0.006827 | 12 |
| 240 | GO:0050709 | negative regulation of protein secretion | 12/55 | 18111/236328 | 0.000794 | 0.006827 | 12 |
| 241 | GO:0090596 | sensory organ morphogenesis | 32/231 | 18111/236328 | 0.00086 | 0.007305 | 32 |
| 242 | GO:0002697 | regulation of immune effector process | 92/861 | 18111/236328 | 0.000888 | 0.007509 | 92 |
| 243 | GO:0045747 | positive regulation of Notch signaling pathway | 8/28 | 18111/236328 | 0.000913 | 0.007631 | 8 |
| 244 | GO:0010611 | regulation of cardiac muscle hypertrophy | 8/28 | 18111/236328 | 0.000913 | 0.007631 | 8 |
| 245 | GO:0014743 | regulation of muscle hypertrophy | 8/28 | 18111/236328 | 0.000913 | 0.007631 | 8 |
| 246 | GO:0042542 | response to hydrogen peroxide | 44/351 | 18111/236328 | 0.000943 | 0.007851 | 44 |
| 247 | GO:0097553 | calcium ion transmembrane import into cytosol | 18/105 | 18111/236328 | 0.001021 | 0.008459 | 18 |
| 248 | GO:0048738 | cardiac muscle tissue development | 64/561 | 18111/236328 | 0.001032 | 0.008519 | 64 |
| 249 | GO:0051251 | positive regulation of lymphocyte activation | 133/1326 | 18111/236328 | 0.001036 | 0.00852 | 133 |
| 250 | GO:0070997 | neuron death | 67/595 | 18111/236328 | 0.001124 | 0.0092 | 67 |
| 251 | GO:0001503 | ossification | 161/1653 | 18111/236328 | 0.001198 | 0.009766 | 161 |
| 252 | GO:0046651 | lymphocyte proliferation | 95/903 | 18111/236328 | 0.001207 | 0.009766 | 95 |
| 253 | GO:0032943 | mononuclear cell proliferation | 95/903 | 18111/236328 | 0.001207 | 0.009766 | 95 |
| 254 | GO:1903706 | regulation of hemopoiesis | 171/1770 | 18111/236328 | 0.001231 | 0.009918 | 171 |
| 255 | GO:0043552 | positive regulation of phosphatidylinositol 3-kinase activity | 9/36 | 18111/236328 | 0.001272 | 0.01017 | 9 |
| 256 | GO:0090218 | positive regulation of lipid kinase activity | 9/36 | 18111/236328 | 0.001272 | 0.01017 | 9 |
| 257 | GO:0045933 | positive regulation of muscle contraction | 13/66 | 18111/236328 | 0.001347 | 0.010565 | 13 |
| 258 | GO:0050830 | defense response to Gram-positive bacterium | 13/66 | 18111/236328 | 0.001347 | 0.010565 | 13 |
| 259 | GO:0002824 | positive regulation of adaptive immune response based on somatic recombination of immune receptors built from immunoglobulin superfamily domains | 13/66 | 18111/236328 | 0.001347 | 0.010565 | 13 |
| 260 | GO:0002821 | positive regulation of adaptive immune response | 13/66 | 18111/236328 | 0.001347 | 0.010565 | 13 |
| 261 | GO:0002792 | negative regulation of peptide secretion | 13/66 | 18111/236328 | 0.001347 | 0.010565 | 13 |
| 262 | GO:0050728 | negative regulation of inflammatory response | 25/171 | 18111/236328 | 0.001402 | 0.010915 | 25 |
| 263 | GO:0002822 | regulation of adaptive immune response based on somatic recombination of immune receptors built from immunoglobulin superfamily domains | 25/171 | 18111/236328 | 0.001402 | 0.010915 | 25 |
| 264 | GO:0110020 | regulation of actomyosin structure organization | 16/91 | 18111/236328 | 0.001439 | 0.011118 | 16 |
| 265 | GO:0031532 | actin cytoskeleton reorganization | 16/91 | 18111/236328 | 0.001439 | 0.011118 | 16 |
| 266 | GO:0003012 | muscle system process | 123/1225 | 18111/236328 | 0.001491 | 0.011475 | 123 |
| 267 | GO:0048469 | cell maturation | 29/210 | 18111/236328 | 0.001528 | 0.011673 | 29 |
| 268 | GO:0046890 | regulation of lipid biosynthetic process | 29/210 | 18111/236328 | 0.001528 | 0.011673 | 29 |
| 269 | GO:0045444 | fat cell differentiation | 57/496 | 18111/236328 | 0.001576 | 0.011995 | 57 |
| 270 | GO:0060419 | heart growth | 21/136 | 18111/236328 | 0.001638 | 0.012419 | 21 |
| 271 | GO:0051402 | neuron apoptotic process | 38/300 | 18111/236328 | 0.00166 | 0.012537 | 38 |
| 272 | GO:0071496 | cellular response to external stimulus | 83/780 | 18111/236328 | 0.001705 | 0.012835 | 83 |
| 273 | GO:0050670 | regulation of lymphocyte proliferation | 66/595 | 18111/236328 | 0.001763 | 0.013172 | 66 |
| 274 | GO:0032944 | regulation of mononuclear cell proliferation | 66/595 | 18111/236328 | 0.001763 | 0.013172 | 66 |
| 275 | GO:0034695 | response to prostaglandin E | 10/45 | 18111/236328 | 0.001834 | 0.013556 | 10 |
| 276 | GO:0060688 | regulation of morphogenesis of a branching structure | 10/45 | 18111/236328 | 0.001834 | 0.013556 | 10 |
| 277 | GO:0038061 | NIK/NF-kappaB signaling | 10/45 | 18111/236328 | 0.001834 | 0.013556 | 10 |
| 278 | GO:0006979 | response to oxidative stress | 185/1953 | 18111/236328 | 0.00192 | 0.01414 | 185 |
| 279 | GO:0071236 | cellular response to antibiotic | 48/406 | 18111/236328 | 0.001981 | 0.014484 | 48 |
| 280 | GO:0045621 | positive regulation of lymphocyte differentiation | 19/120 | 18111/236328 | 0.00198 | 0.014484 | 19 |
| 281 | GO:1903037 | regulation of leukocyte cell-cell adhesion | 122/1225 | 18111/236328 | 0.002051 | 0.01489 | 122 |
| 282 | GO:0051271 | negative regulation of cellular component movement | 79/741 | 18111/236328 | 0.002051 | 0.01489 | 79 |
| 283 | GO:0051250 | negative regulation of lymphocyte activation | 14/78 | 18111/236328 | 0.002273 | 0.016441 | 14 |
| 284 | GO:0001935 | endothelial cell proliferation | 45/378 | 18111/236328 | 0.002339 | 0.01686 | 45 |
| 285 | GO:0048639 | positive regulation of developmental growth | 35/276 | 18111/236328 | 0.002411 | 0.017315 | 35 |
| 286 | GO:0070167 | regulation of biomineral tissue development | 17/105 | 18111/236328 | 0.002612 | 0.018568 | 17 |
| 287 | GO:0021782 | glial cell development | 17/105 | 18111/236328 | 0.002612 | 0.018568 | 17 |
| 288 | GO:0055017 | cardiac muscle tissue growth | 17/105 | 18111/236328 | 0.002612 | 0.018568 | 17 |
| 289 | GO:0030850 | prostate gland development | 11/55 | 18111/236328 | 0.002711 | 0.019005 | 11 |
| 290 | GO:0035272 | exocrine system development | 11/55 | 18111/236328 | 0.002711 | 0.019005 | 11 |
| 291 | GO:0032729 | positive regulation of interferon-gamma production | 11/55 | 18111/236328 | 0.002711 | 0.019005 | 11 |
| 292 | GO:1901888 | regulation of cell junction assembly | 11/55 | 18111/236328 | 0.002711 | 0.019005 | 11 |
| 293 | GO:0002526 | acute inflammatory response | 42/351 | 18111/236328 | 0.002903 | 0.020279 | 42 |
| 294 | GO:0045446 | endothelial cell differentiation | 26/190 | 18111/236328 | 0.002934 | 0.020291 | 26 |
| 295 | GO:0045598 | regulation of fat cell differentiation | 26/190 | 18111/236328 | 0.002934 | 0.020291 | 26 |
| 296 | GO:0007519 | skeletal muscle tissue development | 26/190 | 18111/236328 | 0.002934 | 0.020291 | 26 |
| 297 | GO:0070301 | cellular response to hydrogen peroxide | 24/171 | 18111/236328 | 0.002982 | 0.020417 | 24 |
| 298 | GO:0071347 | cellular response to interleukin-1 | 24/171 | 18111/236328 | 0.002982 | 0.020417 | 24 |
| 299 | GO:0040013 | negative regulation of locomotion | 68/630 | 18111/236328 | 0.002964 | 0.020417 | 68 |
| 300 | GO:0048872 | homeostasis of number of cells | 78/741 | 18111/236328 | 0.003028 | 0.020621 | 78 |
| 301 | GO:0060191 | regulation of lipase activity | 28/210 | 18111/236328 | 0.003042 | 0.020621 | 28 |
| 302 | GO:0055024 | regulation of cardiac muscle tissue development | 28/210 | 18111/236328 | 0.003042 | 0.020621 | 28 |
| 303 | GO:0072009 | nephron epithelium development | 20/136 | 18111/236328 | 0.003716 | 0.024947 | 20 |
| 304 | GO:0002440 | production of molecular mediator of immune response | 32/253 | 18111/236328 | 0.003729 | 0.024947 | 32 |
| 305 | GO:0045862 | positive regulation of proteolysis | 32/253 | 18111/236328 | 0.003729 | 0.024947 | 32 |
| 306 | GO:0032649 | regulation of interferon-gamma production | 15/91 | 18111/236328 | 0.003801 | 0.025179 | 15 |
| 307 | GO:2000379 | positive regulation of reactive oxygen species metabolic process | 15/91 | 18111/236328 | 0.003801 | 0.025179 | 15 |
| 308 | GO:0090288 | negative regulation of cellular response to growth factor stimulus | 15/91 | 18111/236328 | 0.003801 | 0.025179 | 15 |
| 309 | GO:0048871 | multicellular organismal homeostasis | 147/1540 | 18111/236328 | 0.003919 | 0.025772 | 147 |
| 310 | GO:0050679 | positive regulation of epithelial cell proliferation | 64/595 | 18111/236328 | 0.004142 | 0.025772 | 64 |
| 311 | GO:0090257 | regulation of muscle system process | 55/496 | 18111/236328 | 0.004005 | 0.025772 | 55 |
| 312 | GO:0035909 | aorta morphogenesis | 12/66 | 18111/236328 | 0.00407 | 0.025772 | 12 |
| 313 | GO:0045927 | positive regulation of growth | 55/496 | 18111/236328 | 0.004005 | 0.025772 | 55 |
| 314 | GO:0061614 | pri-miRNA transcription by RNA polymerase II | 12/66 | 18111/236328 | 0.00407 | 0.025772 | 12 |
| 315 | GO:2000146 | negative regulation of cell motility | 64/595 | 18111/236328 | 0.004142 | 0.025772 | 64 |
| 316 | GO:0030857 | negative regulation of epithelial cell differentiation | 12/66 | 18111/236328 | 0.00407 | 0.025772 | 12 |
| 317 | GO:0097048 | dendritic cell apoptotic process | 5/15 | 18111/236328 | 0.004127 | 0.025772 | 5 |
| 318 | GO:2000668 | regulation of dendritic cell apoptotic process | 5/15 | 18111/236328 | 0.004127 | 0.025772 | 5 |
| 319 | GO:0001771 | immunological synapse formation | 5/15 | 18111/236328 | 0.004127 | 0.025772 | 5 |
| 320 | GO:0060740 | prostate gland epithelium morphogenesis | 6/21 | 18111/236328 | 0.004011 | 0.025772 | 6 |
| 321 | GO:0060512 | prostate gland morphogenesis | 6/21 | 18111/236328 | 0.004011 | 0.025772 | 6 |
| 322 | GO:0045622 | regulation of T-helper cell differentiation | 6/21 | 18111/236328 | 0.004011 | 0.025772 | 6 |
| 323 | GO:0042471 | ear morphogenesis | 12/66 | 18111/236328 | 0.00407 | 0.025772 | 12 |
| 324 | GO:0043280 | positive regulation of cysteine-type endopeptidase activity involved in apoptotic process | 12/66 | 18111/236328 | 0.00407 | 0.025772 | 12 |
| 325 | GO:0097366 | response to bronchodilator | 6/21 | 18111/236328 | 0.004011 | 0.025772 | 6 |
| 326 | GO:0031952 | regulation of protein autophosphorylation | 5/15 | 18111/236328 | 0.004127 | 0.025772 | 5 |
| 327 | GO:0016999 | antibiotic metabolic process | 12/66 | 18111/236328 | 0.00407 | 0.025772 | 12 |
| 328 | GO:0070664 | negative regulation of leukocyte proliferation | 6/21 | 18111/236328 | 0.004011 | 0.025772 | 6 |
| 329 | GO:0097028 | dendritic cell differentiation | 7/28 | 18111/236328 | 0.004357 | 0.026943 | 7 |
| 330 | GO:1901224 | positive regulation of NIK/NF-kappaB signaling | 7/28 | 18111/236328 | 0.004357 | 0.026943 | 7 |
| 331 | GO:0032103 | positive regulation of response to external stimulus | 107/1081 | 18111/236328 | 0.004383 | 0.027025 | 107 |
| 332 | GO:1903039 | positive regulation of leukocyte cell-cell adhesion | 99/990 | 18111/236328 | 0.004449 | 0.027265 | 99 |
| 333 | GO:0090066 | regulation of anatomical structure size | 99/990 | 18111/236328 | 0.004449 | 0.027265 | 99 |
| 334 | GO:0031663 | lipopolysaccharide-mediated signaling pathway | 18/120 | 18111/236328 | 0.004629 | 0.028287 | 18 |
| 335 | GO:0002683 | negative regulation of immune system process | 151/1596 | 18111/236328 | 0.004808 | 0.029293 | 151 |
| 336 | GO:0001936 | regulation of endothelial cell proliferation | 41/351 | 18111/236328 | 0.004909 | 0.029727 | 41 |
| 337 | GO:0007409 | axonogenesis | 70/666 | 18111/236328 | 0.004898 | 0.029727 | 70 |
| 338 | GO:0071731 | response to nitric oxide | 4/10 | 18111/236328 | 0.004968 | 0.029908 | 4 |
| 339 | GO:0014003 | oligodendrocyte development | 4/10 | 18111/236328 | 0.004968 | 0.029908 | 4 |
| 340 | GO:0061437 | renal system vasculature development | 8/36 | 18111/236328 | 0.005131 | 0.030444 | 8 |
| 341 | GO:0061440 | kidney vasculature development | 8/36 | 18111/236328 | 0.005131 | 0.030444 | 8 |
| 342 | GO:0045987 | positive regulation of smooth muscle contraction | 8/36 | 18111/236328 | 0.005131 | 0.030444 | 8 |
| 343 | GO:0014009 | glial cell proliferation | 8/36 | 18111/236328 | 0.005131 | 0.030444 | 8 |
| 344 | GO:0032663 | regulation of interleukin-2 production | 8/36 | 18111/236328 | 0.005131 | 0.030444 | 8 |
| 345 | GO:1903708 | positive regulation of hemopoiesis | 46/406 | 18111/236328 | 0.005369 | 0.031673 | 46 |
| 346 | GO:0043254 | regulation of protein complex assembly | 46/406 | 18111/236328 | 0.005369 | 0.031673 | 46 |
| 347 | GO:0048145 | regulation of fibroblast proliferation | 27/210 | 18111/236328 | 0.005835 | 0.033838 | 27 |
| 348 | GO:0048144 | fibroblast proliferation | 27/210 | 18111/236328 | 0.005835 | 0.033838 | 27 |
| 349 | GO:0002819 | regulation of adaptive immune response | 25/190 | 18111/236328 | 0.005799 | 0.033838 | 25 |
| 350 | GO:0071772 | response to BMP | 25/190 | 18111/236328 | 0.005799 | 0.033838 | 25 |
| 351 | GO:0071773 | cellular response to BMP stimulus | 25/190 | 18111/236328 | 0.005799 | 0.033838 | 25 |
| 352 | GO:0043123 | positive regulation of I-kappaB kinase/NF-kappaB signaling | 27/210 | 18111/236328 | 0.005835 | 0.033838 | 27 |
| 353 | GO:0032355 | response to estradiol | 29/231 | 18111/236328 | 0.006146 | 0.035032 | 29 |
| 354 | GO:0035270 | endocrine system development | 23/171 | 18111/236328 | 0.006071 | 0.035032 | 23 |
| 355 | GO:0006940 | regulation of smooth muscle contraction | 13/78 | 18111/236328 | 0.006161 | 0.035032 | 13 |
| 356 | GO:0030038 | contractile actin filament bundle assembly | 13/78 | 18111/236328 | 0.006161 | 0.035032 | 13 |
| 357 | GO:0043149 | stress fiber assembly | 13/78 | 18111/236328 | 0.006161 | 0.035032 | 13 |
| 358 | GO:0032231 | regulation of actin filament bundle assembly | 13/78 | 18111/236328 | 0.006161 | 0.035032 | 13 |
| 359 | GO:0035148 | tube formation | 13/78 | 18111/236328 | 0.006161 | 0.035032 | 13 |
| 360 | GO:0051224 | negative regulation of protein transport | 16/105 | 18111/236328 | 0.006278 | 0.035498 | 16 |
| 361 | GO:1904950 | negative regulation of establishment of protein localization | 16/105 | 18111/236328 | 0.006278 | 0.035498 | 16 |
| 362 | GO:1903727 | positive regulation of phospholipid metabolic process | 9/45 | 18111/236328 | 0.006411 | 0.036051 | 9 |
| 363 | GO:0032623 | interleukin-2 production | 9/45 | 18111/236328 | 0.006411 | 0.036051 | 9 |
| 364 | GO:0061564 | axon development | 94/946 | 18111/236328 | 0.006437 | 0.0361 | 94 |
| 365 | GO:0072511 | divalent inorganic cation transport | 51/465 | 18111/236328 | 0.006648 | 0.037179 | 51 |
| 366 | GO:0033273 | response to vitamin | 21/153 | 18111/236328 | 0.006741 | 0.037395 | 21 |
| 367 | GO:0006939 | smooth muscle contraction | 21/153 | 18111/236328 | 0.006741 | 0.037395 | 21 |
| 368 | GO:2001235 | positive regulation of apoptotic signaling pathway | 21/153 | 18111/236328 | 0.006741 | 0.037395 | 21 |
| 369 | GO:0097237 | cellular response to toxic substance | 79/780 | 18111/236328 | 0.00743 | 0.040625 | 79 |
| 370 | GO:0003007 | heart morphogenesis | 79/780 | 18111/236328 | 0.00743 | 0.040625 | 79 |
| 371 | GO:0045625 | regulation of T-helper 1 cell differentiation | 3/6 | 18111/236328 | 0.007542 | 0.040625 | 3 |
| 372 | GO:0043588 | skin development | 48/435 | 18111/236328 | 0.00739 | 0.040625 | 48 |
| 373 | GO:0060397 | JAK-STAT cascade involved in growth hormone signaling pathway | 3/6 | 18111/236328 | 0.007542 | 0.040625 | 3 |
| 374 | GO:0090231 | regulation of spindle checkpoint | 3/6 | 18111/236328 | 0.007542 | 0.040625 | 3 |
| 375 | GO:0034616 | response to laminar fluid shear stress | 3/6 | 18111/236328 | 0.007542 | 0.040625 | 3 |
| 376 | GO:0032930 | positive regulation of superoxide anion generation | 3/6 | 18111/236328 | 0.007542 | 0.040625 | 3 |
| 377 | GO:0032928 | regulation of superoxide anion generation | 3/6 | 18111/236328 | 0.007542 | 0.040625 | 3 |
| 378 | GO:0045624 | positive regulation of T-helper cell differentiation | 3/6 | 18111/236328 | 0.007542 | 0.040625 | 3 |
| 379 | GO:0032692 | negative regulation of interleukin-1 production | 3/6 | 18111/236328 | 0.007542 | 0.040625 | 3 |
| 380 | GO:0002576 | platelet degranulation | 33/276 | 18111/236328 | 0.007639 | 0.041042 | 33 |
| 381 | GO:0006801 | superoxide metabolic process | 10/55 | 18111/236328 | 0.008359 | 0.044442 | 10 |
| 382 | GO:0014910 | regulation of smooth muscle cell migration | 10/55 | 18111/236328 | 0.008359 | 0.044442 | 10 |
| 383 | GO:0017015 | regulation of transforming growth factor beta receptor signaling pathway | 10/55 | 18111/236328 | 0.008359 | 0.044442 | 10 |
| 384 | GO:1903844 | regulation of cellular response to transforming growth factor beta stimulus | 10/55 | 18111/236328 | 0.008359 | 0.044442 | 10 |
| 385 | GO:0021700 | developmental maturation | 45/406 | 18111/236328 | 0.008553 | 0.045357 | 45 |
| 386 | GO:0030336 | negative regulation of cell migration | 59/561 | 18111/236328 | 0.008962 | 0.047405 | 59 |

**Experiment group:**

| No. | ID | Description | GeneRatio | BgRatio | pvalue | p.adjust | count |
| --- | --- | --- | --- | --- | --- | --- | --- |
| 1 | GO:1903829 | positive regulation of cellular protein localization | 151/595 | 41977/236328 | 2.11E-06 | 0.002159 | 151 |
| 2 | GO:0008654 | phospholipid biosynthetic process | 61/190 | 41977/236328 | 1.25E-06 | 0.002159 | 61 |
| 3 | GO:0045017 | glycerolipid biosynthetic process | 64/210 | 41977/236328 | 4.96E-06 | 0.003385 | 64 |
| 4 | GO:0046488 | phosphatidylinositol metabolic process | 144/595 | 41977/236328 | 4.64E-05 | 0.012982 | 144 |
| 5 | GO:1905330 | regulation of morphogenesis of an epithelium | 61/210 | 41977/236328 | 4.05E-05 | 0.012982 | 61 |
| 6 | GO:0001657 | ureteric bud development | 32/91 | 41977/236328 | 5.56E-05 | 0.012982 | 32 |
| 7 | GO:0072163 | mesonephric epithelium development | 32/91 | 41977/236328 | 5.56E-05 | 0.012982 | 32 |
| 8 | GO:0065004 | protein-DNA complex assembly | 75/276 | 41977/236328 | 6.98E-05 | 0.012982 | 75 |
| 9 | GO:0006338 | chromatin remodeling | 47/153 | 41977/236328 | 6.67E-05 | 0.012982 | 47 |
| 10 | GO:0031497 | chromatin assembly | 39/120 | 41977/236328 | 6.97E-05 | 0.012982 | 39 |
| 11 | GO:0006333 | chromatin assembly or disassembly | 39/120 | 41977/236328 | 6.97E-05 | 0.012982 | 39 |
| 12 | GO:0071824 | protein-DNA complex subunit organization | 80/300 | 41977/236328 | 8.21E-05 | 0.014001 | 80 |
| 13 | GO:0034728 | nucleosome organization | 35/105 | 41977/236328 | 9.02E-05 | 0.01421 | 35 |
| 14 | GO:0090316 | positive regulation of intracellular protein transport | 46/153 | 41977/236328 | 0.000139 | 0.020356 | 46 |
| 15 | GO:0046777 | protein autophosphorylation | 114/465 | 41977/236328 | 0.000154 | 0.020977 | 114 |
| 16 | GO:0034508 | centromere complex assembly | 27/78 | 41977/236328 | 0.000275 | 0.028569 | 27 |
| 17 | GO:0031055 | chromatin remodeling at centromere | 21/55 | 41977/236328 | 0.000279 | 0.028569 | 21 |
| 18 | GO:0006336 | DNA replication-independent nucleosome assembly | 21/55 | 41977/236328 | 0.000279 | 0.028569 | 21 |
| 19 | GO:0034724 | DNA replication-independent nucleosome organization | 21/55 | 41977/236328 | 0.000279 | 0.028569 | 21 |
| 20 | GO:0033157 | regulation of intracellular protein transport | 63/231 | 41977/236328 | 0.000224 | 0.028569 | 63 |
| 21 | GO:0046854 | phosphatidylinositol phosphorylation | 94/378 | 41977/236328 | 0.000326 | 0.029132 | 94 |
| 22 | GO:0034080 | CENP-A containing nucleosome assembly | 18/45 | 41977/236328 | 0.000384 | 0.029132 | 18 |
| 23 | GO:0061641 | CENP-A containing chromatin organization | 18/45 | 41977/236328 | 0.000384 | 0.029132 | 18 |
| 24 | GO:0032388 | positive regulation of intracellular transport | 67/253 | 41977/236328 | 0.00036 | 0.029132 | 67 |
| 25 | GO:0043486 | histone exchange | 18/45 | 41977/236328 | 0.000384 | 0.029132 | 18 |
| 26 | GO:0043044 | ATP-dependent chromatin remodeling | 18/45 | 41977/236328 | 0.000384 | 0.029132 | 18 |
| 27 | GO:0009913 | epidermal cell differentiation | 66/253 | 41977/236328 | 0.000614 | 0.044862 | 66 |
| 28 | GO:0051701 | interaction with host | 61/231 | 41977/236328 | 0.00069 | 0.048714 | 61 |

**Common:**

| No. | ID | Description | GeneRatio | BgRatio | pvalue | p.adjust | count |
| --- | --- | --- | --- | --- | --- | --- | --- |
| 1 | GO:0072073 | kidney epithelium development | 67/253 | 41977/236328 | 0.00036 | 0.029132 | 67 |
